# iHyd-PseCp: Identify hydroxyproline and hydroxylysine in proteins by incorporating sequence-coupled effects into general PseAAC

**DOI:** 10.18632/oncotarget.10027

**Published:** 2016-06-14

**Authors:** Wang-Ren Qiu, Bi-Qian Sun, Xuan Xiao, Zhao-Chun Xu, Kuo-Chen Chou

**Affiliations:** ^1^ Computer Department, Jingdezhen Ceramic Institute, Jingdezhen, China; ^2^ Department of Computer Science and Bond Life Science Center, University of Missouri, Columbia, MO, USA; ^3^ Gordon Life Science Institute, Boston, MA, USA; ^4^ Center of Excellence in Genomic Medicine Research (CEGMR), King Abdulaziz University, Jeddah, Saudi Arabia; ^5^ Center of Bioinformatics, School of Life Science and Technology, University of Electronic Science and Technology of China, Chengdu, Sichuan, China

**Keywords:** PTMs, hydroxyproline, hydroxylysine, sequence-coupling model, general PseAAC

## Abstract

Protein hydroxylation is a posttranslational modification (PTM), in which a CH group in Pro (P) or Lys (K) residue has been converted into a COH group, or a hydroxyl group (−OH) is converted into an organic compound. Closely associated with cellular signaling activities, this type of PTM is also involved in some major diseases, such as stomach cancer and lung cancer. Therefore, from the angles of both basic research and drug development, we are facing a challenging problem: for an uncharacterized protein sequence containing many residues of P or K, which ones can be hydroxylated, and which ones cannot? With the explosive growth of protein sequences in the post-genomic age, the problem has become even more urgent. To address such a problem, we have developed a predictor called iHyd-PseCp by incorporating the sequence-coupled information into the general pseudo amino acid composition (PseAAC) and introducing the “Random Forest” algorithm to operate the calculation. Rigorous jackknife tests indicated that the new predictor remarkably outperformed the existing state-of-the-art prediction method for the same purpose. For the convenience of most experimental scientists, a user-friendly web-server for iHyd-PseCp has been established at http://www.jci-bioinfo.cn/iHyd-PseCp, by which users can easily obtain their desired results without the need to go through the complicated mathematical equations involved.

## INTRODUCTION

Protein post-translational modification (PTM or PTLM) is one of the most efficient biological mechanisms for expanding the genetic code and regulating cellular physiology. Hydroxylation is one type of PTM that can take place in proteins to hydroxylate proline and lysine. Hydroxyproline (HyP) is the key factor in stabilizing collagens [1, 2], whose instability or abnormal activity may cause stomach cancer [3] and lung cancer [4, 5]. Hydroxylysine (HyL) is also found in collagen, which may affect fibrillogenesis, cross-linking, and matrix mineralization [6]. Consequently, identifying the HyP and HyL sites in proteins is an indispensable step for decoding protein function. It is also crucially important for in-depth understanding the physiological roles of hydroxylation. Meanwhile, it can also provide useful information for developing drugs to treat various diseases associated with hydroxylation.

Although the information of HyP and HyL can be determined by means of large-scale mass spectrometry, it is time-consuming and expensive. Therefore, it is highly demanded to develop computational methods to deal with this problem. In a pioneer work, by incorporating dipeptide position-specific propensity into the general Chou's pseudo amino acid composition (PseAAC) [7] and using the discriminant function algorithm used by Chou et al. for identifying the HIV protease cleavage sites [8, 9], Xu et al. [10] proposed a predictor called iHyd-PseAAC to identify the HyP and HyL sites in proteins. Although these authors did make contribution in stimulating the development of this area, more work is definitely needed to improve the prediction quality. And the current study is to devote to do so by introducing the sequence-coupled approach.

According to the Chou's 5-step rule [7] and concurred by many investigators in a series of recent publications [11–23], for developing a new prediction method that can be widely used by broad users, we should consider the following five points: (1) the prediction method should be with a web-server accessible to public; (2) a compelling demonstration to show its prediction quality being improved over the existing counterparts; (3) a good benchmark dataset used to train or test the new model; (4) an elegant mathematical formulation to represent the statistical samples concerned; and (5) a powerful algorithm to operate the calculation. Below, let us address the above points one-by-one.

## RESULTS AND DISCUSSION

### A new predictor and its user guide

A powerful predictor, called iHyd-PseCp, has been developed for identifying the HyP and HyL sites in proteins. The new predictor is accessible to the public. Users can easily get their desired results by following the instructions below.

**Figure 1:**
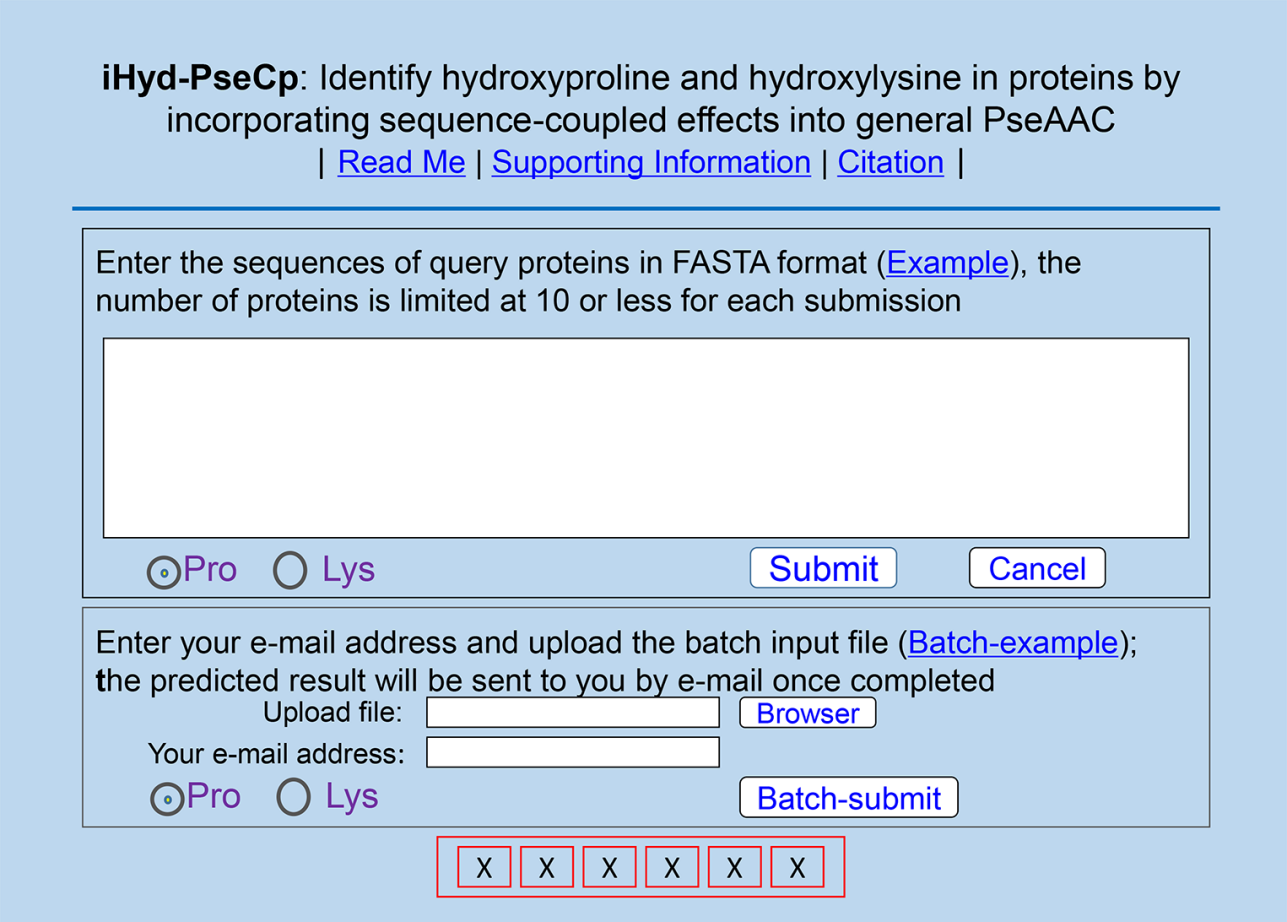
A semi-screenshot to show the top-page of the iHyd-PseCp web-server at http://www.jci-bioinfo.cn/iHyd-PseCp

1. Open the iHyd-PseCp web-server at http://www.jci-bioinfo.cn/iHyd-PseCp, your computer will be prompted with the web-server top-page shown on its screen (Figure 1).
2. In the input box (Figure 1), enter your query protein sequences. This can be done by either typing or copying/pasting manner. The entered query protein sequences should be in the FASTA format. If you are not familiar with FASTA, just click the Example button to see what it looks like.
3. If you wish to predict HyP sites, check on the Pro button; if you wish to predict the HyL sites, check on the Lys button.
4. Click the Submit button to see the predicted result. For example, if you use the sequences of the two query proteins in the Example window as the input and check the Pro button on, after clicking the Submit button, you will see the following predicted results: (a) The total number of Pro (P) in the 1st protein (P35248) is 41, of which those located at the sequence positions 47, 95, 149, 170 and 200 (highlighted with red) are of the hydroxylation site, but the remaining 36 sites are not. (b) The total number of Pro residues in the 2nd protein (Q4ZJN1) is 31, of which those at the sequence positions 31, 34, 40, 58, 61, 64, 76, 115, 151, 160 and 175 (highlighted with red) are of the hydroxylation site, but the remaining 20 sites are not. For the same input sequences, however, if you check on the Lys button, you will instead see the following outcomes after clicking the Submit button: (a) The total number of Lys (K) residues in the 1st protein (P35248) is 21, of which those located at the sequence positions 86 and 98 (highlighted in red) are of the hydroxylation site, but the remaining 19 sites are not. (b) The total number of Lys residues in the 2nd protein (Q4ZJN1) is 24, of which those at the sequence positions 73 and 127 (highlighted in red) are of the hydroxylation site, but the remaining 22 sites are not. It would take about 30 seconds before the aforementioned results shown on your screen. Of course, the more number of query protein sequences or the longer of the sequences concerned, the more time it is usually needed.
5. If you have many query protein sequences and need long computational time, you can also use the batch prediction mode. To do so, just use the Browse button to select the desired file (in FASTA format of course) and follow the online instructions.
6. To download the benchmark dataset used in this study, click the Supporting Information button on the top of Figure 1.
7. If you wish to find the papers closely related to the development of the current new prediction method, click Citation button.

## RESULTS AND ANALYSIS

The success rates achieved by the new predictor iHyd-PseCp via the rigorous jackknife test on the 164 hydroxyproline proteins are given in Table 1, where for facilitating comparison, the corresponding rates obtained by the predictor iHyd-PseAAC [10] are also listed. Also, the jackknife success rates by the new predictor iHyd-PseCp on the 33 hydroxylysine proteins are given in Table 2, along with the corresponding rates obtained by the predictor iHyd-PseAAC [10]. As we can see from Table 1, for predicting the HyP sites, the newly proposed method has remarkably outperformed the state-of-the-art method from all the four angles: overall accuracy Acc, stability MCC, sensitivity Sn, and specificity Sp. As for the prediction of the HyL sites, it can be observed from Table 2 that the new predictor iHyd-PseCp has significantly outperformed iHyd-PseAAC [10] in Acc and MCC. Although the rate of Sn by the new predictor is about 9% lower than that by iHyd-PseAAC, interestingly the rate of Sp by the new predictor is about 16% higher than that by iHyd-PseAAC.

**Table 1:** A comparison of the proposed predictor with the state-of-the-art method in identifying the HyP sites in proteins^a^

Predictor	Acc (%)^d^	MCC^d^	Sn (%)^d^	Sp (%)
iHyd-PseAAC^b^	80.57	0.51	80.66	80.54
iHyd-PseCp^c^	96.58	0.89	86.35	99.12

a The scores here were generated by the rigorous jackknife tests on the 164 hydroxyproline proteins as adopted by Xu et al. [10].

b The predictor developed by Xu et al. [10].

c The predictor proposed in this paper.

d See Eq.9 for the metrics definition.

**Table 2:** A comparison of the proposed predictor with the state-of-the-art method in identifying the HyL sites in proteins^a^

Predictor	Acc (%)^d^	MCC^d^	Sn (%)^d^	Sp (%)^d^
iHyd-PseAAC^b^	83.56	0.50	87.85	83.01
iHyd-PseCp^c^	97.08	0.86	78.77	99.80

a The scores here were generated by the rigorous jackknife tests on the 33 hydroxylysine proteins as adopted by Xu et al. [10].

b The predictor developed by Xu et al. [10].

c The predictor proposed in this paper.

d See Eq.9 for the metrics definition.

It is instructive to point out that, of the four metrics, the most important are the Acc and MCC [11, 12, 21, 22]: the former reflects the overall accuracy of a predictor; while the latter, its stability in practical applications. The metrics Sn and Sp are used to measure a predictor from two opposite angles. When, and only when, both Sn and Sp of the predictor A are higher than those of the predictor B, can we say A is better than B [19]. In other words, Sn and Sp are actually constrained with each other [24]. Therefore, it is meaningless to use only one of the two for comparing the quality of two predictors. A meaningful comparison in this regard should count the rates of both Sn and Sp, or even better count the rate of their combination, which is none but the score of MCC.

**Figure 2:**
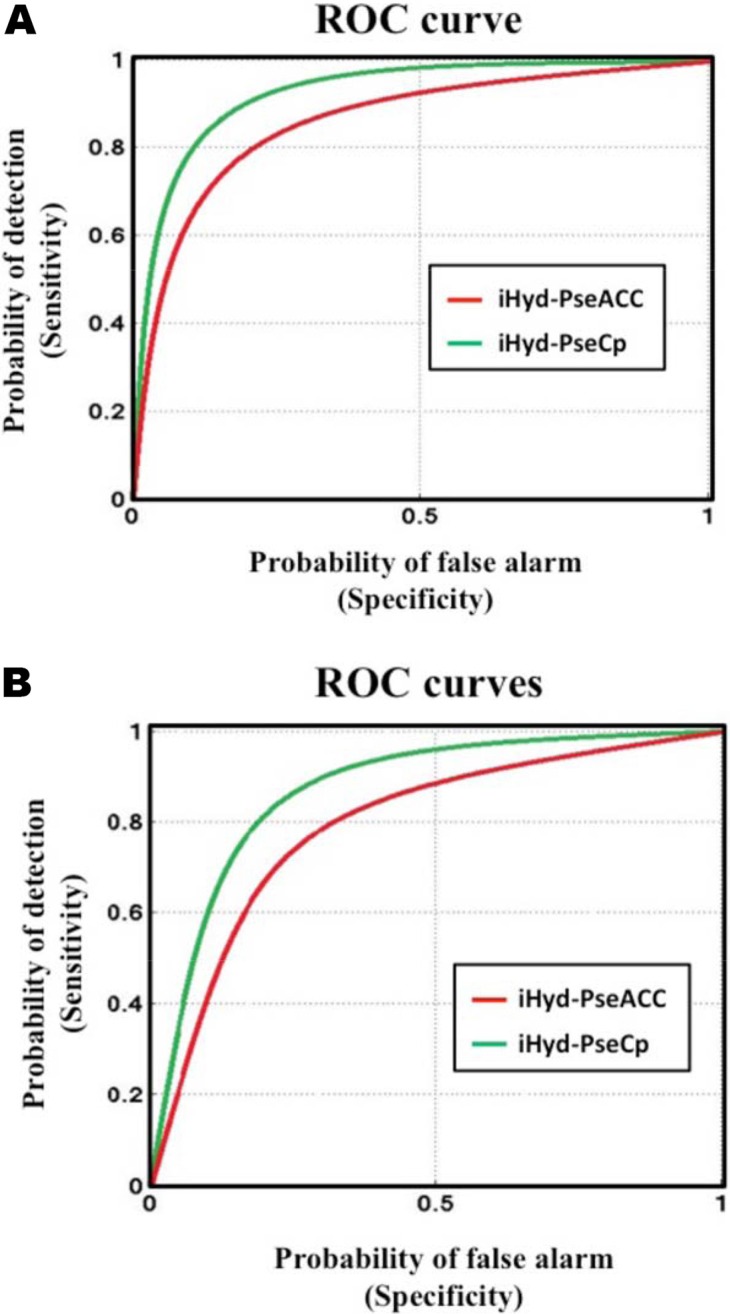
The intuitive graphs of ROC curves to show the performance of iHyd-PseAAC [10] and iHyd-PseCp proposed in this paper, respectively, for the case of (A) HyP and (B) HyL See the main text for further explanation.

Graphic analysis is a very useful vehicle to deal with complicated biological systems as demonstrated by a series of previous studies (see, e.g., [25–50]). To provide an intuitive comparison of the proposed method with the existing state-of-the art method [10] by using the graphic analysis, let us use the Receiver Operating Characteristic (ROC) graphs [51, 52] as given in Figure 2. In the figure, the green and red graphic lines are the ROC curves for iHyd-PseCp and iHyd-PseAAC [10], respectively, where panel (a) is for the case in predicting HyP sites in proteins, and panel (b) for the case of HyL. The area under the ROC curve is called AUC (area under the curve). The greater the AUC value is, the better the predictor will be [51, 52]. As we can see from Figure 2, the area under the green curve is remarkably greater than that under the red line for both the HyP and HyL cases, once again indicating that the proposed predictor is indeed much better than iHyd-PseAAC [10]. Accordingly, we anticipate that iHyd-PseCp will become a useful bioinformatics tool for identifying HyP and HyL sites in proteins, or at the very least, play a complementary role to the existing state-of-the art tool in this area.

Why could the proposed method be able to increase the prediction quality so substantially? This is due to the fact that the amino-acid-coupled effects around the hydroxylation sites have been taken into account via the conditional probability approach. Similar remarkable successes have also been observed in predicting beta-turns [53], alpha-turns [54], tight turns and their types in proteins [55], specificity of GalNAc-transferase [56], HIV protease cleavage sites [8, 24, 57], as well as signal peptide cleavage sites [58–60].

## MATERIALS AND METHODS

### Benchmark dataset

The benchmark dataset used in this study was derived from the same proteins as used by Xu et al. [10]. They consist of 164 hydroxyproline proteins and 33 hydroxylysine proteins. The former were used to construct the benchmark dataset for studying the HyP sites, while the latter used to construct the benchmark dataset for studying the HyL sites.

To make the description mathematically more rigorous and clear, the Chou's scheme [61] was adopted to formulate peptide samples, as done recently by many authors in studying the nitrotyrosine sites [62], methylation sites [63], protein-protein interaction [64], and protein-protein binding sites [65]. According to Chou's scheme, a potential hydroxylation site-containing peptide sample can be generally expressed by

$$P_{\xi }(⊛)=R_{-\xi }R_{-(\xi -1)}\cdots R_{-2}R_{-1}⊛R_{+1}R_{+2}\cdots R_{+(\xi -1)}R_{+\xi }$$ (1)

where the symbol ⊛ denotes the single amino acid code P or K, the subscript ξ is an integer, R_−ξ_ represents the ξ-th upstream amino acid residue from the center, the R_+ξ_ the ξ-th downstream amino acid residue, and so forth. The (2ξ + 1)-tuple peptide sample $P_{\xi }(⊛)$ can be further classified into the following two categories:

$$P_{\xi }(⊛)\in \{\begin{array}{l} P_{\xi }^{+}(⊛),\text{if its center is a hydroxylation site}\\ P_{\xi }^{-}(⊛),\text{other wise} \end{array}$$ (2)

where $P_{\xi }^{+}(⊛)$ denotes a true hydroxylation segment with P or K at its center, $P_{\xi }^{-}(⊛)$ a false hydroxylation segment with P or K at its center, and the symbol ∈ means “a member of” in the set theory.

In literature the benchmark dataset usually consists of a training dataset and a testing dataset: the former is used for training a model, while the latter for testing the model. But as pointed out in a comprehensive review [66], there is no need to artificially separate a benchmark dataset into the two parts if the prediction model is examined by the jackknife test or subsampling (K-fold) cross-validation since the outcome thus obtained is actually from a combination of many different independent dataset tests.

Thus, the benchmark dataset $S_{\hat{1}}(⊛)$ for the current study can be formulated as

$$\{\begin{array}{l} S_{\xi }\left(P\right)=S_{\xi }^{+}\left(P\right)\cup S_{\xi }^{-}\left(P\right),\text{ when }⊛=P\\ S_{\xi }\left(K\right)=S_{\xi }^{+}\left(K\right)\cup S_{\xi }^{-}\left(K\right),\text{ when }⊛=K \end{array}$$ (3)

where the positive subset $S_{\xi }^{+}(⊛)$ only contains the samples of true hydroxylation segments $P_{\xi }^{+}(⊛)$, and the negative subset $S_{\xi }^{-}(⊛)$ only contains the samples of false hydroxylation segments $P_{\xi }^{-}(⊛)$ (see Eq.2); while ⋃ represents the symbol for “union” in the set theory.

The detailed procedures in constructing the benchmark dataset $S_{\xi }\left(P\right)$ are as follows. (1) As done in [61], slide the (2ξ + 1)-tuple peptide window along each of the aforementioned 164 hydroxyproline protein sequences, and collected were only those peptide segments that have P (Pro) at the center. (2) If the upstream or downstream in a protein sequence was less than ξ or greater than *L* − ξ where *L* is the length of the protein sequence concerned, the lacking amino acid was filled with a dummy residue X. (3) The peptide segment samples thus obtained were put into the positive subset if their centers have been experimentally annotated as the hydroxylation sites; otherwise, into the negative subset. (4) The peptide samples thus obtained were subject to a screening procedure to window those that were identical to any other in a same subset; excluded from the benchmark dataset were also those that were self-conflict, namely, occurring in both hydroxylation group and non-hydroxylation group.

By following the same procedures but using the 33 hydroxylysine proteins and focusing on K (Lys), instead of the 164 hydroxyproline proteins and P (Pro), we obtained the benchmark dataset $S_{\xi }\left(K\right)$.

Because the length of peptide sample $P_{\xi }(⊛)$ is 2ξ + 1 (see Eq.1), the benchmark dataset with different ξ value will contain peptide segments with different number of amino acid residues, as illustrated below

$$\text{Length of peptide samples in }S_{\xi }(⊛)=\{\begin{array}{c} 13\text{ amino acid residues},\\ \begin{array}{c} \begin{array}{c} 14\text{ amino acid residues},\\ 17\text{ amino acid residues}, \end{array}\\ 19\text{ amino acid residues}, \end{array}\\ \begin{array}{l} 21\text{ amino acid residues},\\ \vdots \end{array} \end{array}\begin{array}{c} \text{if }\xi =6\\ \begin{array}{c} \begin{array}{c} \text{if }\xi =7\\ \text{if }\xi =8 \end{array}\\ \text{if }\xi =9 \end{array}\\ \begin{array}{l} \text{if }\xi =10\\ \vdots \end{array} \end{array}$$ (4)

But preliminary tests had indicated that it would be most promising when ξ = 10. Consequently, for further study below, instead of Eq.3, we shall consider

$$\{\begin{array}{l} S_{\xi =10}\left(P\right)=S_{\xi =10}^{+}\left(P\right)\cup S_{\xi =10}^{-}\left(P\right),\text{ when }⊛=P\\ S_{\xi =10}\left(K\right)=S_{\xi =10}^{+}\left(K\right)\cup S_{\xi =10}^{-}\left(K\right),\text{ when }⊛=K \end{array}$$ (5)

where the benchmark dataset $S_{\xi =10}\left(P\right)$ contains 4,356 (2ξ + 1) = 21-tuple peptide samples, of which 851 belong to the positive subset $S_{\xi =10}^{+}\left(P\right)$, and 3,505 to the negative subset $S_{\xi =10}^{-}\left(P\right)$; the benchmark dataset $S_{\xi =10}\left(K\right)$ contains 1,122 (2ξ + 1) = 21-tuple peptide samples, of which 142 belong to the positive subset $S_{\xi =10}^{+}\left(K\right)$, and 980 to the negative subset $S_{\xi =10}^{+}\left(K\right)$. For readers’ convenience, the detailed sequences of the aforementioned positive and negative samples in $S_{\xi =10}\left(P\right)$ and $S_{\xi =10}\left(K\right)$ are given in Online Supporting Information S1 and Online Supporting Information S2, respectively.

### Sequence-coupled information and general PseAAC

With the avalanche of biological sequence generated in the post-genomic age, one of the most important problems in computational biology is how to formulate a biological sequence with a discrete model or a vector, yet still considerably keep its sequence pattern or order information. This is because all the existing machine-learning algorithms can only handle vector but not sequence samples, as elaborated in [67].

To address this problem, the pseudo amino acid composition [68, 69] or PseAAC was proposed. Ever since the concept of pseudo amino acid composition or Chou's PseAAC [70–72] was proposed, it has rapidly penetrated into nearly all the areas of computational proteomics (see, e.g., [73–80] as well as a long list of references cited in [81, 82]) and many biomedicine and drug development areas [67, 83–86]. Because it has been widely and increasingly used, recently three powerful open access soft-wares, called ‘PseAAC-Builder’ [70], ‘propy’ [71], and ‘PseAAC-General’ [81], were established: the former two are for generating various modes of Chou's special PseAAC; while the 3rd one for those of Chou's general PseAAC [7], including not only all the special modes of feature vectors for proteins but also the higher level feature vectors such as “Functional Domain” mode (see Eqs. 9–10 of [7]), “Gene Ontology” mode (see Eqs. 11–12 of [7]), and “Sequential Evolution” or “PSSM” mode (see Eqs.13–14 of [7]). Inspired by the successes of using PseAAC to deal with protein/peptide sequences, three web-servers [87–89] were developed for generating various feature vectors for DNA/RNA sequences as well. Particularly, recently a powerful web-server called Pse-in-One [90] has been developed that can be used to generate any desired feature vectors for protein/peptide and DNA/RNA sequences according to the need of users’ studies.

According to the general PseAAC [7], the peptide sequence of Eq.1 can be formulated as

$$P_{\xi =10}(⊛)=P_{\xi =10}^{+}(⊛)-P_{\xi =10}^{-}(⊛)$$ (6)

where

$$P_{\xi =10}^{+}(⊛)=\left[\begin{array}{c} p_{-10}^{+}(R_{-10}|R_{-9})\\ p_{-9}^{+}(R_{-9}|R_{-8})\\ \vdots \\ p_{-2}^{+}(R_{-2}|R_{-1})\\ p_{-1}^{+}(R_{-1})\\ p_{+1}^{+}(R_{+1})\\ p_{+2}^{+}(R_{+2}|R_{+1})\\ \vdots \\ p_{+9}^{+}(R_{+9}|R_{+8})\\ p_{+10}^{+}(R_{+10}|R_{+9}) \end{array}\right]$$ (7)

and

$$P_{\xi =10}^{-}(⊛)=\left[\begin{array}{c} p_{-10}^{-}(R_{-10}|R_{-9})\\ p_{-9}^{-}(R_{-9}|R_{-8})\\ \vdots \\ p_{-2}^{-}(R_{-2}|R_{-1})\\ p_{-1}^{-}(R_{-1})\\ p_{+1}^{-}(R_{+1})\\ p_{+2}^{-}(R_{+2}|R_{+1})\\ \vdots \\ p_{+9}^{-}(R_{+9}|R_{+8})\\ p_{+10}^{-}(R_{+10}|R_{+9}) \end{array}\right]$$ (8)

In Eq.7 $p_{-10}^{+}(R_{-10}|R_{-9})$ is the conditional probability of amino acid R_−10_ occurring at the left 1st position (see Eq.1) given that its closest right neighbor is $p_{-9}^{+}(R_{-9}|R_{-8})$ is the conditional probability of amino acid R_9_ occurring at the left 2nd position given that its closest right neighbor is R_−8_, and so forth. Note that in Eq.7, only $p_{-1}^{+}(R_{-1})$ and $p_{+1}^{+}(R_{+1})$ are of non-conditional probability since the right neighbor of R_−1_ and the left neighbor of R_+1_ are always ⊛ (namely Pro for the case of HyP, or Lys for the case of HyL). All these probability values can be easily derived from the positive training subsets taken from Supporting Information S1 and S2, respectively, as done in [9]. Likewise, the components in Eq.8 are the same as those in Eq.7 except for that they are derived from the negative training subsets in Supporting Information S1 and S2, respectively.

### Random forests algorithm

The random forests (RF) algorithm is a powerful algorithm and has been widely used in many areas of computational biology (see, e.g. [13–15, 64, 65, 91–93]). The algorithm of random forest is based on the ensemble of a large number of decision trees, where each tree gives a classification and the forest chooses the final classification via the most votes (over all the trees in the forest). In the most commonly used type of random forests, split selection is performed based on the so-called decrease of Gini impurity. In this study, the random forest is used to rank the features using Gini importance that is implemented with the machine learning platform scikit-learn. The detailed procedures of RF and its formulation have been very clearly described in [94], and hence there is no need to repeat here.

For the current study, all the involved peptide samples were converted into a 20-D (dimensional) vector according to Eq.6, and then entered into the RF operation engine as the input. And the output would indicate whether the center residue ⊛ of the query peptide is a “hydroxylation site” or “non-hydroxylation site”. Note that, in using the current prediction method, one must observe the self-consistency principle: if the center residue of a query peptide is ⊛ = P, then the corresponding training data must be taken from $S_{\xi =10}(P)$; if the center residue of a query peptide is ⊛ = K, then the training data must be taken from $S_{\xi =10}(K)$.

The predictor established via the above procedures is called “iHyd-PseCp”, where “i” stands for “identify”, “Hyd” for “hydroxylation site”, “Pse” for “general PseAAC”, and “Cp” for “sequence coupled effect”.

As pointed out in the Introduction section, one of the keys in establishing a useful predictor is how to properly evaluate its anticipated success rates. To realize this, we need to consider the following two things: one is what metrics or scales should be used to quantitatively measure its prediction quality; the other is what validation method should be adopted to calculate or derive the metrics values. Below, let us address the two problems.

### A set of four metrics

The following four metrics are usually used in literature to measure the quality of binary classification: (1) overall accuracy or Acc; (2) Mathew's correlation coefficient or MCC; (3) sensitivity or Sn; and (4) specificity or Sp (see, e.g., [95]). Unfortunately, the conventional formulations for the four are not intuitive and that most experimental scientists feel difficult to understand them, particularly for the one of MCC. Interestingly, by using the Chou's symbols and derivation in studying signal peptides [96], the aforementioned four metrics can be easily converted into a set of following equations [97, 98]:

$$\{\begin{array}{ll} \mathrm{Sn}=1=\frac{N_{-}^{+}}{N^{+}} & 0\leq \mathrm{Sp}\leq 1\\ \mathrm{Sn}=1=\frac{N_{+}^{-}}{N^{-}} & 0\leq \mathrm{Sp}\leq 1\\ \mathrm{Acc}=\Lambda =1=\frac{N_{-}^{+}+N_{+}^{-}}{N^{+}+N^{-}} & 0\leq \mathrm{Acc}\leq 1\\ \mathrm{MCC}=\frac{1-\left(\frac{N_{-}^{+}+N_{+}^{-}}{N^{+}+N^{-}}\right)}{\sqrt{\left(1+\frac{N_{+}^{-}+N_{-}^{+}}{N^{+}}\right)\left(1+\frac{N_{-}^{+}+N_{+}^{-}}{N^{-}}\right)}} & -1\leq \mathrm{MCC}\leq 1 \end{array}$$ (9)

where *N*^+^ represents the total number of hydroxylation sites investigated whereas $N_{-}^{+}$ the number of true hydroxylation sites incorrectly predicted to be of non-hydroxylation site; *N*^−^ the total number of the non-hydroxylation sites investigated whereas $N_{+}^{-}$ the number of non-hydroxylation sites incorrectly predicted to be ofhydroxylation site.

According to Eq.9, it is crystal clear to see the following. When $N_{-}^{+}=0$ meaning none of the true hydroxylation sites are incorrectly predicted to be of non-hydroxylation site, we have the sensitivity Sn = 1. When $N_{-}^{+}=N^{+}$ meaning that all the hydroxylation sites are incorrectly predicted to be of non-hydroxylation site, we have the sensitivity Sn = 0. Likewise, when $N_{+}^{-}=0$ meaning none of the non-hydroxylation sites are incorrectly predicted to be of hydroxylation site, we have the specificity Sp = 1; whereas $N_{+}^{-}=N^{-}$ meaning that all the non-hydroxylation sites are incorrectly predicted to be of hydroxylation sites, we have the specificity Sp = 0. When $N_{-}^{+}=N_{+}^{-}=0$ meaning that none of hydroxylation sites in the positive dataset and none of the non-hydroxylation sites in the negative dataset are incorrectly predicted, we have the overall accuracy Acc = 1 and Mcc = 1; when $N_{-}^{+}=N^{+}$ and $N_{+}^{-}=N^{-}$ meaning that all the hydroxylation sites in the positive dataset and all thenon-hydroxylation sites in the negative dataset are incorrectly predicted, we have the overall accuracy Acc = 0 and Mcc = −1; whereas when $N_{-}^{+}=N^{+}/2$ and $N_{+}^{-}=N^{-}/2$ we have Acc = 0.5 and Mcc = 0 meaning no better than random guess. Therefore, using Eq.9 has made the meanings of sensitivity, specificity, overall accuracy, and Mathew's correlation coefficient much more intuitive and easier-to-understand, particularly for the meaning of MCC, as concurred recently by many investigators (see, e.g., [11, 12, 16, 18, 19, 21–23, 64, 65, 99–108]).

Note that, however, the set of equations defined in Eq.9 is valid only for the single-label systems. For the multi-label systems whose emergence has become more frequent in system biology [109–111] and system medicine [112], a completely different set of metrics are needed as elaborated in [113].

### Jackknife test

With a set of well-defined metrics to measuring the quality of a predictor, the next thing is what kind of validation method should be used to score these metrics. In predictive analytics, the following three cross-validation methods are often used: (1) independent dataset test, (2) subsampling (or K-fold cross-validation) test, and (3) jackknife test [114]. Of these three, however, the jackknife test is deemed the least arbitrary that can always yield a unique outcome for a given benchmark dataset as elucidated in [7]. Accordingly, the jackknife test has been widely recognized and increasingly used by investigators to examine the quality of various predictors (see, e.g., [73–76, 78–80, 115–123]). Therefore, the jackknife test was also adopted in this study to score the metrics of Eq.9. In the jackknife test, each of the samples in the benchmark dataset is singled out one-by-one and tested by the predictor trained with the remaining samples. During the jackknifing process, both the training dataset and testing dataset are literally open, and each sample is in turn moved between the two. The jackknife test can exclude the “memory” effect; it can also avoid the arbitrariness problem occurring in the independent dataset test and subsampling test as pointed out in [7] because the outcome obtained by the jackknife test is always unique for a given benchmark dataset.

## CONCLUSIONS

The iHyd-PseCp predictor is a new bioinformatics tool for identifying the hydroxylation sites in proteins. Compared with the existing state-of-the-art predictor in this area, its prediction quality is much better, with remarkably higher overall accuracy and stability. For the convenience of most experimental scientists, we have provided its web-server and a step-by-step guide, by which users can easily obtain their desired results without the need to go through the detailed mathematics. The reason of including them in this paper is for the integrity of the new prediction method, and that these techniques, such as incorporating the sequence-coupled approach into the general PseAAC, may be of use as well in developing other tools in computational biology.

We anticipate that iHyd-PseCp will become a very useful high throughput tool for both basic research and drug development in the areas relevant to the protein hydroxylation.

## ONLINE SUPPORTING INFORMATION

### Supporting Information S1

The benchmark dataset S(P) used to train and test the model for predicting the possibility, of hydroxylation at Pro site.

### Supporting Information S2

The benchmark dataset S(K) used to train and test the model for predicting the possibility of hydroxylation at Lys site.

## SUPPLEMENTARY MATERIALS






