# Supplementary material for: iHyd-PseCp: Identify hydroxyproline and hydroxylysine in proteins by incorporating sequence-coupled effects into general PseAAC
Source: Oncotarget. 2016 Jun 14;7(28):44310–21. doi: 10.18632/oncotarget.10027 (PMC5190098; doi:10.18632/oncotarget.10027)
Supplement: Supplementary file 1 [file oncotarget-07-44310-s001.pdf]

iHyd-PseCp: Identify hydroxyproline and hydroxylysine in proteins by incorporating sequence-coupled effects into general PseAAC

Supplementary Materials

Supporting Information S1: The benchmark dataset $\mathbb{S}$(P) used to train and test the model for predicting the possibility of hydroxylation at Pro site. It contains 851 positive samples and 3505 negative samples, which were extracted from the 164 hydroxyproline proteins. See the main text for further explanation.

Supporting Information S2: The benchmark dataset $\mathbb{S}$(K) used to train and test the model for predicting the possibility of hydroxylation at Lys site. It contains 142 positive samples and 980 negative samples, which were extracted from the 33 hydroxylysine proteins. See the main text for further explanation.
