# Supplementary material for: iHyd-PseCp: Identify hydroxyproline and hydroxylysine in proteins by incorporating sequence-coupled effects into general PseAAC
Source: Oncotarget. 2016 Jun 14;7(28):44310–21. doi: 10.18632/oncotarget.10027 (PMC5190098; doi:10.18632/oncotarget.10027)
Supplement: Supplementary file 2 [file oncotarget-07-44310-s002.docx]

**Supporting Information S1.** The benchmark dataset $\mathbb{S}(P)$used to train and test the model for predicting the possibility of hydroxylation at Pro site. It contains 851 positive samples and 3505 negative samples, which were extracted from the 164 hydroxyproline proteins. See the main text for further explanation.

(1) List of the 851 peptide samples in the positive subset $\mathbb{S}^{+}(P)$

| Sample # | Protein ID | Site | Sequences |
| --- | --- | --- | --- |
| 1  2  3  4  5  6  7  8  9  10  11  12  13  14  15  16  17  18  19  20  21  22  23  24  25  26  27  28  29  30  31  32  33  34  35  36  37  38  39  40  41  42  43  44  45  46  47  48  49  50  51  52  53  54  55  56  57  58  59  60  61  62  63  64  65  66  67  68  69  70  71  72  73  74  75  76  77  78  79  80  81  82  83  84  85  86  87  88  89  90  91  92  93  94  95  96  97  98  99  100  101  102  103  104  105  106  107  108  109  110  111  112  113  114  115  116  117  118  119  120  121  122  123  124  125  126  127  128  129  130  131  132  133  134  135  136  137  138  139  140  141  142  143  144  145  146  147  148  149  150  151  152  153  154  155  156  157  158  159  160  161  162  163  164  165  166  167  168  169  170  171  172  173  174  175  176  177  178  179  180  181  182  183  184  185  186  187  188  189  190  191  192  193  194  195  196  197  198  199  200  201  202  203  204  205  206  207  208  209  210  211  212  213  214  215  216  217  218  219  220  221  222  223  224  225  226  227  228  229  230  231  232  233  234  235  236  237  238  239  240  241  242  243  244  245  246  247  248  249  250  251  252  253  254  255  256  257  258  259  260  261  262  263  264  265  266  267  268  269  270  271  272  273  274  275  276  277  278  279  280  281  282  283  284  285  286  287  288  289  290  291  292  293  294  295  296  297  298  299  300  301  302  303  304  305  306  307  308  309  310  311  312  313  314  315  316  317  318  319  320  321  322  323  324  325  326  327  328  329  330  331  332  333  334  335  336  337  338  339  340  341  342  343  344  345  346  347  348  349  350  351  352  353  354  355  356  357  358  359  360  361  362  363  364  365  366  367  368  369  370  371  372  373  374  375  376  377  378  379  380  381  382  383  384  385  386  387  388  389  390  391  392  393  394  395  396  397  398  399  400  401  402  403  404  405  406  407  408  409  410  411  412  413  414  415  416  417  418  419  420  421  422  423  424  425  426  427  428  429  430  431  432  433  434  435  436  437  438  439  440  441  442  443  444  445  446  447  448  449  450  451  452  453  454  455  456  457  458  459  460  461  462  463  464  465  466  467  468  469  470  471  472  473  474  475  476  477  478  479  480  481  482  483  484  485  486  487  488  489  490  491  492  493  494  495  496  497  498  499  500  501  502  503  504  505  506  507  508  509  510  511  512  513  514  515  516  517  518  519  520  521  522  523  524  525  526  527  528  529  530  531  532  533  534  535  536  537  538  539  540  541  542  543  544  545  546  547  548  549  550  551  552  553  554  555  556  557  558  559  560  561  562  563  564  565  566  567  568  569  570  571  572  573  574  575  576  577  578  579  580  581  582  583  584  585  586  587  588  589  590  591  592  593  594  595  596  597  598  599  600  601  602  603  604  605  606  607  608  609  610  611  612  613  614  615  616  617  618  619  620  621  622  623  624  625  626  627  628  629  630  631  632  633  634  635  636  637  638  639  640  641  642  643  644  645  646  647  648  649  650  651  652  653  654  655  656  657  658  659  660  661  662  663  664  665  666  667  668  669  670  671  672  673  674  675  676  677  678  679  680  681  682  683  684  685  686  687  688  689  690  691  692  693  694  695  696  697  698  699  700  701  702  703  704  705  706  707  708  709  710  711  712  713  714  715  716  717  718  719  720  721  722  723  724  725  726  727  728  729  730  731  732  733  734  735  736  737  738  739  740  741  742  743  744  745  746  747  748  749  750  751  752  753  754  755  756  757  758  759  760  761  762  763  764  765  766  767  768  769  770  771  772  773  774  775  776  777  778  779  780  781  782  783  784  785  786  787  788  789  790  791  792  793  794  795  796  797  798  799  800  801  802  803  804  805  806  807  808  809  810  811  812  813  814  815  816  817  818  819  820  821  822  823  824  825  826  827  828  829  830  831  832  833  834  835  836  837  838  839  840  841  842  843  844  845  846  847  848  849  850  851 | A1X158  A1X158  A1X158  A1X158  A1X158  A1X158  A1X158  A1X158  A1X158  A1X158  A1X158  A1X158  A1X158  A1X158  A1X158  A1X158  A1X158  A1X158  A1X158  A1X158  A1X158  A1X158  A1X158  A1X158  A1X158  A6YR20  A6YR20  B2CS62  B2KPN7  B4YSU8  C3VVN6  C4PWC4  D2Y168  D2Y171  D2Y171  D2Y171  D2Y171  O82337  O82337  O82337  P00877  P00877  P01042  P01522  P01522  P01522  P01523  P01523  P01523  P02453  P02457  P02457  P02457  P02457  P02457  P02457  P02457  P02457  P02457  P02457  P02457  P02457  P02457  P02457  P02457  P02457  P02457  P02457  P02457  P02457  P02457  P02457  P02457  P02457  P02457  P02457  P02457  P02457  P02457  P02457  P02457  P02457  P02457  P02457  P02457  P02457  P02457  P02457  P02457  P02457  P02457  P02457  P02457  P02457  P02457  P02457  P02457  P02457  P02457  P02457  P02457  P02457  P02457  P02457  P02457  P02457  P02457  P02457  P02457  P02457  P02457  P02457  P02457  P02457  P02457  P02457  P02457  P02457  P02457  P02457  P02457  P02457  P02457  P02457  P02457  P02457  P02457  P02457  P02457  P02457  P02457  P02457  P02457  P02457  P02457  P02457  P02457  P02457  P02457  P02457  P02457  P02467  P02745  P02745  P02745  P02745  P02745  P02745  P02745  P02747  P02747  P02747  P02747  P02747  P02747  P02747  P02747  P02747  P02747  P02747  P02820  P02821  P05482  P05482  P05482  P05483  P05483  P05539  P05539  P05539  P05539  P05539  P05539  P05539  P05997  P05997  P05997  P05997  P05997  P05997  P05997  P07550  P07550  P08252  P08252  P08252  P08252  P08252  P08252  P08427  P08427  P08427  P08427  P08427  P08427  P08427  P08427  P08427  P08427  P08661  P08661  P08661  P08661  P08661  P0C1N1  P0C1N5  P0C1N5  P0C1W5  P0C1W5  P0C1W5  P0C1W6  P0C1W6  P0C1W6  P0C1W8  P0C1W8  P0C1X1  P0C1X1  P0C1X1  P0C1X1  P0C1X1  P0C1X1  P0C1X1  P0C2W2  P0C2W2  P0C2W2  P0C2W2  P0C2W2  P0C2W2  P0C2W2  P0C2W2  P0C2W8  P0C2W8  P0C2W8  P0C2W8  P0C2W8  P0C2W8  P0C2W8  P0C2W8  P0C2W8  P0C2W8  P0C2W8  P0C2W8  P0C2W8  P0C2W8  P0C2W8  P0C2W8  P0C2W8  P0C2W8  P0C2W8  P0C2W8  P0C2W8  P0C2W8  P0C2W8  P0C2W8  P0C2W8  P0C2W8  P0C2W8  P0C2W8  P0C2W8  P0C2W8  P0C2W8  P0C2W8  P0C2W8  P0C2W8  P0C2W8  P0C2W8  P0C2W8  P0C2W8  P0C349  P0C349  P0C351  P0C424  P0C8S5  P0C8S5  P0C8S5  P0C8V5  P0C8V5  P0C8V6  P0C8V6  P0C8V9  P0C8V9  P0C8W0  P0C8W0  P0C8W0  P0C8W0  P0CE29  P0CE30  P0CG45  P0CG45  P0CG45  P0CG45  P0CG45  P0CG45  P0CG45  P0CH13  P0CH13  P0CH16  P0CH16  P0CH18  P0CH18  P0CH18  P0CH18  P0CH20  P12108  P12108  P12111  P12111  P12111  P12111  P12111  P14618  P14618  P15472  P15472  P15502  P15502  P15502  P15502  P15502  P15502  P15502  P15502  P15502  P15502  P15502  P15502  P15502  P15502  P15502  P15502  P15502  P15502  P15502  P15502  P15502  P15502  P15502  P15502  P15502  P15502  P15502  P15502  P15502  P15502  P15502  P15502  P15502  P15502  P15502  P19999  P19999  P19999  P19999  P19999  P23805  P23805  P23805  P23805  P23805  P23805  P23805  P23805  P23805  P23805  P24091  P24091  P28880  P29602  P29602  P29602  P29602  P29602  P29602  P29602  P29602  P29602  P29602  P29602  P30754  P30754  P30754  P30754  P30754  P30754  P30754  P30754  P30754  P30754  P30754  P30754  P30754  P30754  P30754  P30754  P30754  P30754  P30754  P30754  P30754  P30754  P30754  P30754  P30754  P30754  P30754  P30754  P30754  P30754  P30754  P30754  P30754  P30754  P30754  P30754  P30754  P30754  P30754  P30754  P30754  P30754  P30754  P30754  P30754  P30754  P30754  P30754  P30754  P30754  P30754  P30754  P30754  P30754  P30754  P30754  P30754  P30754  P30754  P30754  P30754  P30754  P30754  P30754  P30754  P30754  P30754  P30754  P30754  P30754  P30754  P30754  P30754  P30754  P30754  P30754  P30754  P30754  P30754  P30754  P30754  P30754  P30754  P30754  P30754  P30754  P30754  P30754  P30754  P30754  P30754  P30754  P30754  P30754  P30754  P30754  P30754  P30754  P30754  P30754  P30754  P30754  P30754  P30754  P30754  P39056  P50982  P52285  P55963  P55963  P55963  P56529  P56529  P56529  P56633  P56710  P56710  P56711  P58782  P58782  P58782  P58782  P58782  P58786  P58787  P58804  P58804  P58808  P58841  P58843  P58846  P58913  P58913  P58914  P58914  P58915  P58915  P58915  P58916  P58922  P58923  P58923  P58924  P58925  P58925  P58928  P58928  P58928  P60245  P60245  P60245  P60274  P60513  P69746  P69746  P69747  P69765  P80760  P80760  P80760  P80760  P80762  P80762  P80762  P80762  P80762  P80774  P81727  P81755  P82439  P82439  P82439  P82439  P82439  P83184  P83184  P83184  P84349  P84698  P84698  P84698  P84895  P84900  P84935  P84946  P85009  P85011  P85012  P85012  P85016  P85016  P85016  P85016  P85017  P85020  P85020  P85020  P85022  P85065  P85153  P85153  P85153  P85153  P85153  P85153  P85153  P85153  P85153  P85153  P85154  P85154  P85154  P85154  P85154  P85154  P85154  P85154  P85154  P85154  P85154  P85154  P85154  P85154  P85154  P85154  P85154  P85154  P85154  P85154  P85154  P85886  P86256  P86289  P86289  P86289  P86289  P86289  P86290  P86290  P86290  P86500  P86500  Q02388  Q02388  Q02388  Q02388  Q02388  Q02388  Q02388  Q05707  Q05707  Q05707  Q05707  Q05707  Q05707  Q05707  Q05707  Q05707  Q05707  Q05707  Q05707  Q05707  Q05707  Q05707  Q05707  Q05707  Q05707  Q05707  Q05707  Q05707  Q05707  Q05707  Q05707  Q05707  Q05707  Q05707  Q05707  Q05707  Q05707  Q05707  Q05707  Q05707  Q05707  Q05707  Q05707  Q05707  Q05707  Q05707  Q0VKG8  Q0VTT8  Q15848  Q15848  Q15848  Q15848  Q15848  Q15848  Q15848  Q16665  Q16665  Q24940  Q24940  Q25460  Q25460  Q25460  Q25460  Q25460  Q25460  Q25460  Q25460  Q25460  Q25460  Q25460  Q25460  Q25460  Q25460  Q25460  Q25460  Q25460  Q25460  Q25460  Q25460  Q25460  Q25460  Q25460  Q25460  Q25460  Q25460  Q25460  Q25460  Q25460  Q25460  Q25460  Q25460  Q25460  Q25460  Q25460  Q25460  Q25460  Q25460  Q25460  Q25460  Q25460  Q25460  Q25460  Q25460  Q25460  Q25460  Q25460  Q25460  Q25460  Q25460  Q2I2Q5  Q3Y5Z3  Q3Y5Z3  Q3Y5Z3  Q3Y5Z3  Q4ZJN1  Q4ZJN1  Q4ZJN1  Q4ZJN1  Q4ZJN1  Q4ZJN1  Q4ZJN1  Q4ZJN1  Q4ZJN1  Q4ZJN1  Q4ZJN1  Q60994  Q60994  Q6PSU2  Q6PSU2  Q6PSU2  Q7M1I4  Q7M1I4  Q7M1I4  Q7M1I4  Q7M1I4  Q7M1I4  Q7M1I4  Q7M1I4  Q7M1I4  Q7M1I4  Q7M1I4  Q7M1I4  Q7M1I4  Q7XAD0  Q7XAD0  Q7XAD0  Q7XAD0  Q7XAD0  Q7XAD0  Q7XAD0  Q7XAD0  Q7XAD0  Q7XAD0  Q7XAD0  Q7XAD0  Q7XAD0  Q7Z091  Q7Z091  Q7Z092  Q7Z092  Q7Z092  Q7Z094  Q7Z094  Q7Z096  Q7Z096  Q7Z096  Q800F1  Q8LG54  Q8LG54  Q8LG54  Q8LG54  Q8LG54  Q93WP7  Q93WP7  Q93WP7  Q93WP7  Q93WP7  Q93WP7  Q93WP7  Q93WP7  Q93WP8  Q93WP8  Q93WP8  Q93WP8  Q93WP8  Q93WP8  Q941C7  Q941C7  Q9BPB1  Q9BPB2  Q9BPB2  Q9BPJ7  Q9C5S0  Q9C5S0  Q9C5S0  Q9C5S0  Q9C5S0  Q9LJD9  Q9LJD9  Q9LJD9  Q9LVC0  Q9LVC0  Q9LVC0  Q9LYF6  Q9LYF6  Q9LYF6  Q9M0S4  Q9M0S4  Q9M0S4  Q9M0S4  Q9M0S4  Q9SJY7  Q9SJY7  Q9SJY7  Q9SJY7  Q9SJY7  Q9STQ3  Q9STQ3  Q9STQ3  Q9U3Z3  Q9UKV8  Q9Y2N7  Q9ZT16  Q9ZT16  Q9ZT16  Q9ZT16  Q9ZT16  Q9ZT16  Q9ZT16  Q9ZT16  Q9ZT16 | 43  53  65  72  73  85  92  93  105  113  125  135  175  185  195  202  203  215  255  315  345  352  353  415  435  65  82  8  64  51  55  55  57  50  58  62  64  31  33  35  104  151  383  49  55  66  56  57  67  1163  179  182  185  194  197  200  215  230  236  245  251  269  278  281  287  296  302  317  323  332  335  362  365  377  383  392  398  401  416  425  428  440  449  464  470  479  485  497  503  512  518  524  533  536  545  554  560  572  581  584  590  593  611  629  635  641  647  653  659  671  680  692  704  707  713  719  728  737  746  761  767  776  788  794  797  806  812  830  839  911  917  965  986  1067  1109  1148  1154  1169  1172  1175  1178  442  39  45  54  57  73  79  85  36  39  42  45  54  63  81  93  96  99  105  60  9  6  7  17  4  7  602  839  1076  1118  1133  1139  1145  919  1156  290  293  296  611  617  382  395  67  69  71  72  74  75  30  33  36  42  54  57  63  67  70  76  43  58  69  78  81  64  55  65  55  70  74  59  75  94  5  11  40  55  60  61  69  70  74  6  12  15  30  294  474  480  567  128  173  182  185  212  215  242  248  251  266  290  292  299  314  320  323  335  362  530  611  617  710  716  758  764  767  812  815  863  869  887  890  959  962  965  998  1001  1004  8  18  3  13  10  24  28  57  65  57  65  55  67  53  68  93  97  56  56  2  3  7  8  13  15  21  4  14  8  18  6  13  20  29  5  158  178  2100  2206  2239  2316  2319  403  408  10  11  116  190  283  286  327  342  347  360  415  427  481  580  589  598  607  677  34  65  67  88  156  167  170  177  290  352  355  421  465  467  522  550  646  769  772  43  61  67  73  78  78  96  108  111  129  132  147  153  171  195  67  69  55  115  116  117  121  122  127  128  129  133  134  136  21  24  27  39  54  72  90  93  123  128  150  162  164  174  177  180  207  216  219  228  237  243  249  255  273  276  285  291  303  306  312  321  327  339  348  366  372  375  381  387  417  423  429  432  453  465  483  500  503  506  513  525  533  536  540  552  561  603  621  627  645  648  663  708  711  714  717  723  744  759  774  783  792  816  843  849  855  861  867  888  894  903  915  945  954  963  966  984  990  1011  1014  1017  1020  53  161  165  416  551  647  773  815  1010  1013  1016  1019  9  46  143  7  13  20  52  53  64  49  6  14  76  7  13  21  22  27  57  57  21  22  60  11  5  62  57  65  4  7  4  10  21  7  61  10  11  55  57  67  2  17  22  2  3  14  16  6  5  11  55  49  6  11  16  21  6  8  9  10  11  10  13  63  6  7  8  9  10  5  7  12  60  3  7  21  3  54  2  3  6  6  7  8  4  5  10  15  11  5  15  18  11  14  3  12  336  345  414  420  426  648  413  650  176  182  284  293  410  473  479  491  497  503  599  605  704  710  728  731  761  806  815  824  902  55  11  30  39  84  90  107  6  12  33  24  43  2167  2176  2185  2188  2664  2667  2673  1467  1470  1482  1497  1503  1517  1520  1532  1538  1544  1550  1556  1565  1568  1574  1577  1580  1595  1598  1643  1656  1659  1662  1665  1668  1674  1677  1680  1686  1689  1704  1715  1726  1729  1732  1735  1741  1747  1756  56  52  44  47  53  71  76  91  95  402  564  109  196  89  93  115  145  149  155  159  175  179  185  189  199  211  221  225  237  365  433  437  443  447  469  479  483  519  523  543  549  559  563  583  595  629  635  645  649  659  665  701  753  757  763  767  773  777  813  817  833  837  867  58  39  42  48  86  31  34  40  58  61  64  76  115  151  160  175  47  94  67  74  86  8  9  10  11  12  17  18  19  20  26  27  28  29  51  55  56  57  58  63  79  80  82  119  120  121  122  8  26  2  11  29  2  11  38  47  65  54  24  26  28  35  36  42  43  45  49  50  149  150  152  42  43  45  150  151  153  63  64  58  49  54  65  23  26  27  31  33  32  34  36  32  34  36  27  29  31  24  26  28  32  36  24  26  28  34  35  31  33  35  53  700  492  24  26  28  32  33  34  37  38  39 | PKPWTAWKVH**P**PAWTAWKAHP  PPAWTAWKAH**P**PAWTAWKATP  AWTAWKATPK**P**WTAWKAPPPA  TPKPWTAWKA**P**PPAWTAWKAT  PKPWTAWKAP**P**PAWTAWKATP  AWTAWKATPK**P**WTAWKAPPPT  TPKPWTAWKA**P**PPTWTAWKAT  PKPWTAWKAP**P**PTWTAWKATP  TWTAWKATPK**P**WTAWKAPPPA  PKPWTAWKAP**P**PAWTAWKATL  AWTAWKATLK**P**WTAWKATPKP  PWTAWKATPK**P**WTAWKATPKP  PWTAWKATPK**P**WTVWKATPKP  PWTVWKATPK**P**WTAWKATPKP  PWTAWKATPK**P**WTAWKAPPPA  TPKPWTAWKA**P**PPAWSAWKAT  PKPWTAWKAP**P**PAWSAWKATP  AWSAWKATPK**P**WTVWKATPKP  PWTVWKATPK**P**WTAWKAPPPA  AWSAWKATPK**P**WTAWKATPKP  PWTAWKATPK**P**WTAWKVPPPA  TPKPWTAWKV**P**PPAWTAWKAH  PKPWTAWKVP**P**PAWTAWKAHP  AWTAWKATPK**P**WTAWKATPKP  PWTVWKATPK**P**WTAWRATPPP  HNGCWCDQEA**P**HGNCCDTDGC  TDGCTAAWWC**P**GTKWDDWKTG  VRCCRVENKC**P**HTVCCDRSRC  RDWEYHAHPK**P**NSFWTLVVLT  CGGTGDSCNE**P**AGELCCRRLK  REVQECQVDT**P**GSSWGKCCMT  RDVQECQVVT**P**GSKWGRCCLN  NTCDSSNKCS**P**GFPGGPFGPS  IRQRDAADVK**P**VARTNEGPGR  VKPVARTNEG**P**GRDPAPCCQH  ARTNEGPGRD**P**APCCQHPIET  TNEGPGRDPA**P**CCQHPIETCC  LSLAGAQSLA**P**APAPTSDGTS  LAGAQSLAPA**P**APTSDGTSID  GAQSLAPAPA**P**TSDGTSIDQG  DNQYIAYVAY**P**IDLFEEGSVT  PPAYVKTFVG**P**PHGIQVERDK  LGMISLMKRP**P**GFSPFRSSRI  ELSLSTRCKS**P**GSSCSPTSYN  RCKSPGSSCS**P**TSYNCCRSCN  TSYNCCRSCN**P**YTKRCYGGYC  LFEKRRDCCT**P**PKKCKDRQCK  FEKRRDCCTP**P**KKCKDRQCKP  PKKCKDRQCK**P**QRCCAGRRGA  GLNGLPGPIG**P**PGPRGRTGDA  PMGPAGPRGL**P**GPPGAPGPQG  PAGPRGLPGP**P**GAPGPQGFQG  PRGLPGPPGA**P**GPQGFQGPPG  APGPQGFQGP**P**GEPGEPGASG  PQGFQGPPGE**P**GEPGASGPMG  FQGPPGEPGE**P**GASGPMGPRG  PMGPRGPAGP**P**GKNGDDGEAG  DDGEAGKPGR**P**GQRGPPGPQG  KPGRPGQRGP**P**GPQGARGLPG  PPGPQGARGL**P**GTAGLPGMKG  ARGLPGTAGL**P**GMKGHRGFSG  FSGLDGAKGQ**P**GPAGPKGEPG  QPGPAGPKGE**P**GSPGENGAPG  PAGPKGEPGS**P**GENGAPGQMG  EPGSPGENGA**P**GQMGPRGLPG  APGQMGPRGL**P**GERGRPGPSG  PRGLPGERGR**P**GPSGPAGARG  PAGARGNDGA**P**GAAGPPGPTG  NDGAPGAAGP**P**GPTGPAGPPG  PPGPTGPAGP**P**GFPGAAGAKG  PTGPAGPPGF**P**GAAGAKGETG  SEGPQGSRGE**P**GPPGPAGAAG  PQGSRGEPGP**P**GPAGAAGPAG  PAGAAGPAGN**P**GADGQPGAKG  PAGNPGADGQ**P**GAKGATGAPG  QPGAKGATGA**P**GIAGAPGFPG  ATGAPGIAGA**P**GFPGARGPSG  APGIAGAPGF**P**GARGPSGPQG  PSGPQGPSGA**P**GPKGNSGEPG  APGPKGNSGE**P**GAPGNKGDTG  PKGNSGEPGA**P**GNKGDTGAKG  NKGDTGAKGE**P**GPAGVQGPPG  EPGPAGVQGP**P**GPAGEEGKRG  EEGKRGARGE**P**GPAGLPGPAG  ARGEPGPAGL**P**GPAGERGAPG  LPGPAGERGA**P**GSRGFPGADG  ERGAPGSRGF**P**GADGIAGPKG  ADGIAGPKGP**P**GERGSPGAVG  PKGPPGERGS**P**GAVGPKGSPG  SPGAVGPKGS**P**GEAGRPGEAG  PKGSPGEAGR**P**GEAGLPGAKG  EAGRPGEAGL**P**GAKGLTGSPG  LPGAKGLTGS**P**GSPGPDGKTG  AKGLTGSPGS**P**GPDGKTGPPG  SPGPDGKTGP**P**GPAGQDGRPG  PPGPAGQDGR**P**GPAGPPGARG  QDGRPGPAGP**P**GARGQAGVMG  ARGQAGVMGF**P**GPKGAAGEPG  FPGPKGAAGE**P**GKPGERGAPG  PKGAAGEPGK**P**GERGAPGPPG  EPGKPGERGA**P**GPPGAVGAAG  KPGERGAPGP**P**GAVGAAGKDG  KDGEAGAQGP**P**GPTGPAGERG  ERGEQGPAGA**P**GFQGLPGPAG  PAGAPGFQGL**P**GPAGPPGEAG  FQGLPGPAGP**P**GEAGKPGEQG  PAGPPGEAGK**P**GEQGVPGNAG  EAGKPGEQGV**P**GNAGAPGPAG  EQGVPGNAGA**P**GPAGARGERG  PAGARGERGF**P**GERGVQGPPG  FPGERGVQGP**P**GPQGPRGANG  PQGPRGANGA**P**GNDGAKGDAG  NDGAKGDAGA**P**GAPGNEGPPG  AKGDAGAPGA**P**GNEGPPGLEG  APGAPGNEGP**P**GLEGMPGERG  NEGPPGLEGM**P**GERGAAGLPG  MPGERGAAGL**P**GAKGDRGDPG  LPGAKGDRGD**P**GPKGADGAPG  DPGPKGADGA**P**GKDGLRGLTG  LRGLTGPIGP**P**GPAGAPGDKG  PIGPPGPAGA**P**GDKGEAGPPG  APGDKGEAGP**P**GPAGPTGARG  PAGPTGARGA**P**GDRGEPGPPG  ARGAPGDRGE**P**GPPGPAGFAG  APGDRGEPGP**P**GPAGFAGPPG  PPGPAGFAGP**P**GADGQPGAKG  FAGPPGADGQ**P**GAKGETGDAG  DAGAKGDAGP**P**GPAGPTGAPG  PPGPAGPTGA**P**GPAGVGAPGP  GPAGRPGEPG**P**AGPPGPPGEK  GEPGPAGPPG**P**PGEKGSPGAD  GERGFPGLPG**P**SGEPGKQGPS  GASGERGPPG**P**MGPPGLAGPP  GPAGPAGPPG**P**AGARGPAGPQ  GFSGLQGPPG**P**PGAPGEQGPS  GKDGLNGLPG**P**IGPPGPRGRT  GLPGPIGPPG**P**RGRTGEVGPV  GEVGPVGPPG**P**PGPPGPPGPP  GPVGPPGPPG**P**PGPPGPPSGG  GPPGPPGPPG**P**PGPPSGGFDL  GPPGPPGPPG**P**PSGGFDLSFL  GFPGADGRVG**P**IGPAGNRGEP  PDGKKGEAGR**P**GRRGRPGLKG  EAGRPGRRGR**P**GLKGEQGEPG  RPGLKGEQGE**P**GAPGIRTGIQ  LKGEQGEPGA**P**GIRTGIQGLK  IQGLKGDQGE**P**GPSGNPGKVG  DQGEPGPSGN**P**GKVGYPGPSG  PSGNPGKVGY**P**GPSGPLGARG  GQANTGCYGI**P**GMPGLPGAPG  NTGCYGIPGM**P**GLPGAPGKDG  CYGIPGMPGL**P**GAPGKDGYDG  IPGMPGLPGA**P**GKDGYDGLPG  APGKDGYDGL**P**GPKGEPGIPA  LPGPKGEPGI**P**AIPGIRGPKG  PKGQKGEPGL**P**GHPGKNGPMG  HPGKNGPMGP**P**GMPGVPGPMG  KNGPMGPPGM**P**GVPGPMGIPG  PMGPPGMPGV**P**GPMGIPGEPG  MPGVPGPMGI**P**GEPGEEGRYK  RRYLDHWLGA**P**APYPDPLEPK  LYYLAPGLGA**P**APYPDPLEPK  TCCDRRDCCT**P**PKKCKDRRCK  CCDRRDCCTP**P**KKCKDRRCKP  PKKCKDRRCK**P**LKCCAACCKL  PTGPSKCCKS**P**GTPCSRGMRD  PSKCCKSPGT**P**CSRGMRDCCT  GPSGFQGLPG**P**PGPPGEGGKQ  GFPGAAGRVG**P**PGSNGNPGPA  GFTGLQGLPG**P**PGPSGDQGTS  GSNGIPGPIG**P**PGPRGRSGET  GRSGETGPAG**P**PGNPGPPGPP  GPAGPPGNPG**P**PGPPGPPGPG  GNPGPPGPPG**P**PGPGIDMSAF  GFPGSAGRVG**P**PGPAGAPGPA  GFTGLQGLPG**P**PGPNGEQGSA  FAGSPGARGF**P**GAPGLPGLKG  SPGARGFPGA**P**GLPGLKGHRG  ARGFPGAPGL**P**GLKGHRGHKG  PPGSIGIRGQ**P**GSMGLPGPKG  IRGQPGSMGL**P**GPKGSSGDPG  KENKLLCEDL**P**GTEDFVGHQG  EDFVGHQGTV**P**SDNIDSQGRN  GNCQSQCPGG**P**TPTPPTPPGG  CQSQCPGGPT**P**TPPTPPGGGD  SQCPGGPTPT**P**PTPPGGGDLG  QCPGGPTPTP**P**TPPGGGDLGS  PGGPTPTPPT**P**PGGGDLGSII  GGPTPTPPTP**P**GGGDLGSIIS  CNVTDVCAGS**P**GIPGAPGNHG  TDVCAGSPGI**P**GAPGNHGLPG  CAGSPGIPGA**P**GNHGLPGRDG  IPGAPGNHGL**P**GRDGRDGVKG  RDGRDGVKGD**P**GPPGPMGPPG  RDGVKGDPGP**P**GPMGPPGGMP  DPGPPGPMGP**P**GGMPGLPGRD  PGPMGPPGGM**P**GLPGRDGLPG  MGPPGGMPGL**P**GRDGLPGAPG  MPGLPGRDGL**P**GAPGAPGERG  ACSSPGLNGF**P**GKDGHDGAKG  HDGAKGEKGE**P**GQGLRGLQGP  GQGLRGLQGP**P**GKVGPAGPPG  PPGKVGPAGP**P**GNPGSKGATG  KVGPAGPPGN**P**GSKGATGPKG  CRLSCGLGCH**P**CCGGCCPHCG  PMFDAVRDCC**P**LPACPFGCNP  PLPACPFGCN**P**CCGGCCPNCG  RDVQDCQVST**P**GSKWGRCCLN  GRCCLNRVCG**P**MCCPASHCYC  LNRVCGPMCC**P**ASHCYCVYHR  SECIINTRDS**P**WGRCCRTRMC  RTRMCGSMCC**P**RNGCTCVYHW  HWRRGHGRSC**P**GGPCSRGHGR  NPVGCCCCGV**P**NAACPPCVCN  CCGVPNAACP**P**CVCNKTCGGC  GRNAVVHERA**P**ELVVTATTNC  TATTNCCGYN**P**MTICPPCMCT  CCGYNPMTIC**P**PCMCTYSCPP  CGYNPMTICP**P**CMCTYSCPPK  CPPCMCTYSC**P**PKRKPGRRND  PPCMCTYSCP**P**KRKPGRRNDD  CTYSCPPKRK**P**GRRNDDNRRG  AGTAGGATGA**P**GIAGAPGFPG  ATGAPGIAGA**P**GFPGARGAPG  APGIAGAPGF**P**GARGAPGPQG  APGPQGPSGA**P**GPKWQSRLHK  MTPALGVQGP**P**GPQGPRLIYT  RYYLPGSAGP**P**GATGFPGAAG  SAGPPGATGF**P**GAAGRSQMFW  ISYMAGVVGL**P**GQRRQGPLGV  DAGPAGPKGE**P**GSPGENGAPG  NDGATGAAGP**P**GPTGPAGPPG  PPGPTGPAGP**P**GFPGAVGAKG  PTGPAGPPGF**P**GAVGAKGEAG  SEGPQGVRGE**P**GPPGPAGAAG  PQGVRGEPGP**P**GPAGAAGPAG  QPGAKGANGA**P**GIAGAPGFPG  ANGAPGIAGA**P**GFPGARGPAG  APGIAGAPGF**P**GARGPAGPQG  PAGPQGPSGA**P**GPKGNSGEPG  SKGDAGAKGE**P**GPIGIQGPPG  GDAGAKGEPG**P**IGIQGPPGPA  EPGPIGIQGP**P**GPAGEEGKRG  EEGKRGARGE**P**GPTGLPGPPG  ARGEPGPTGL**P**GPPGERGGPG  EPGPTGLPGP**P**GERGGPGSRG  ERGGPGSRGF**P**GADGVAGPKG  SPGPAGPKGS**P**GEAGRPGEAG  FPGERGVQGP**P**GPAGPRGSNG  PRGLTGPIGP**P**GPAGAPGDKG  PIGPPGPAGA**P**GDKGEAGPSG  AKGARGSAGP**P**GATGFPGAAG  SAGPPGATGF**P**GAAGRVGPPG  PRGETGPAGR**P**GEVGPPGPPG  PAGRPGEVGP**P**GPPGPAGEKG  RPGEVGPPGP**P**GPAGEKGSPG  LPGQRGERGF**P**GLPGPSGEPG  QRGERGFPGL**P**GPSGEPGKQG  REGAPGAEGS**P**GRDGSPGPKG  AEGSPGRDGS**P**GPKGDRGETG  ETGPSGPPGA**P**GAPGAPGPVG  PSGPPGAPGA**P**GAPGPVGPAG  HRGFSGLQGP**P**GPPGSPGEQG  FSGLQGPPGP**P**GSPGEQGPSG  LQGPPGPPGS**P**GEQGPSGASG  APGKDGLNGL**P**GPPGPPGPRG  KDGLNGLPGP**P**GPPGPRGRTG  LNGLPGPPGP**P**GPRGRTGDAG  GHRRHGCCKG**P**KGCSSRECRP  PKGCSSRECR**P**QHCCCCHQPR  NWCCGPSQQS**P**GCCWNPACVK  APSACRLGCR**P**CCRRCCPRCG  TTYGIYDAKP**P**FSCAGLRGGC  AGLRGGCVLP**P**NLRPKFKEEK  GGCVLPPNLR**P**KFKEEKFKPR  KLNKRDECFS**P**GTFCGIKPGL  FSPGTFCGIK**P**GLCCSAWCYS  KLNKRDECYP**P**GTFCGIKPGL  YPPGTFCGIK**P**GLCCSERCFP  DCIEAGNYCG**P**TVMKICCGFC  VMKICCGFCS**P**FSKICMNYPQ  LRRLEKRGCD**P**TDGCQTTVCE  QTTVCETDTG**P**CCCKPNFTCQ  GTKSCSCSGQ**P**SDCPVVPCDS  CSCSGQPSDC**P**VVPCDSPQGS  DNEAECQINT**P**GSSWGKCCMT  DNEAECQINT**P**GSSWGKCCLT  KPPTCCPPLL**P**PCCTPPKKHC  PPTCCPPLLP**P**CCTPPKKHCP  CPPLLPPCCT**P**PKKHCPAPAC  PPLLPPCCTP**P**KKHCPAPACK  PCCTPPKKHC**P**APACKYKPCC  CTPPKKHCPA**P**ACKYKPCCKS  HCPAPACKYK**P**CCKSSKCCPK  EGGPICDDCI**P**GGENCDVFRP  PGGENCDVFR**P**YRCCSGYCIL  VREERVCCGY**P**MSCKSRACKP  PMSCKSRACK**P**SYCCCCYSPK  IFSGFFGSFI**P**CAHKGEPCTI  SFIPCAHKGE**P**CTICCRPLRC  KGEPCTICCR**P**LRCHEEKTPT  RPLRCHEEKT**P**TCVVCTPTKE  TPFNGYYGNF**P**TCSETGEDCS  PPGPPGKPGP**P**GHIQGVEGSA  ADFLCPTNCP**P**GPKGPQGLQG  QRGVKGSRGF**P**GEKGEVGEIG  KNGGFGRRGP**P**GAKGNKGGPG  AQGPAGPAGP**P**GLIGEQGISG  RRGKKGERGF**P**GYPGPKGNPG  KKGERGFPGY**P**GPKGNPGEPG  QLFEELRRLA**P**ITSDPTEATA  LRRLAPITSD**P**TEATAVGAVE  AACSGRGSRC**P**PQCCMGLRCG  ACSGRGSRCP**P**QCCMGLRCGR  AKAGAGLGGV**P**GVGGLGVSAG  PGVGGAFAGI**P**GVGPFGGPQP  VLPGVGGAGV**P**GVPGAIPGIG  GVGGAGVPGV**P**GAIPGIGGIA  GAAAGLVPGG**P**GFGPGVVGVP  GVVGVPGAGV**P**GVGVPGAGIP  PGAGVPGVGV**P**GAGIPVVPGA  GIPVVPGAGI**P**GAAVPGVVSP  PGFGVGVGGI**P**GVAGVPGVGG  VAGVPGVGGV**P**GVGGVPGVGI  VPGVPGTGGV**P**GVGTPAAAAA  RAAAGLGAGI**P**GLGVGVGVPG  IPGLGVGVGV**P**GLGVGAGVPG  VPGLGVGAGV**P**GLGVGAGVPG  VPGLGVGAGV**P**GFGAGADEGV  GLGALGGVGI**P**GGVVGAGPAA  SRPGGVPGAI**P**GGVPGGVFYP  ALGPGGKPLK**P**VPGGLAGAGL  GPGGKPLKPV**P**GGLAGAGLGA  GLGAFPAVTF**P**GALVPGGVAD  LPGVYPGGVL**P**GARFPGVGVL  GARFPGVGVL**P**GVPTGAGVKP  FPGVGVLPGV**P**TGAGVKPKAP  PGVPTGAGVK**P**KAPGVGGAFA  AGVPGVPGAI**P**GIGGIAGVGT  PGVGVPGAGI**P**VVPGAGIPGA  GVPGAGIPVV**P**GAGIPGAAVP  VGGIPGVAGV**P**GVGGVPGVGG  AGAGVLGGLV**P**GPQAAVPGVP  AGVLGGLVPG**P**QAAVPGVPGT  VGVAPGVGVA**P**GVGLAPGVGV  PGVGVAPGIG**P**GGVAAAAKSA  LPSTPSSPRV**P**GALAAAKAAK  VAARPGFGLS**P**IFPGGACLGK  RPGFGLSPIF**P**GGACLGKACG  IACGRDGRDG**P**KGEKGEPGQG  GQGLRGLQGP**P**GKLGPPGSVG  LQGPPGKLGP**P**GSVGAPGSQG  KLGPPGSVGA**P**GSQGPKGQKG  GSVGAPGSQG**P**KGQKGDRGDS  SPGPAGRAGR**P**GWVGPIGPKG  PKGDNGFVGE**P**GPKGDTGPRG  PKGDTGPRGP**P**GMPGPAGREG  DTGPRGPPGM**P**GPAGREGPSG  PSGKQGSMGP**P**GTPGPKGETG  KQGSMGPPGT**P**GPKGETGPKG  ETGPKGGVGA**P**GIQGFPGPSG  GVGAPGIQGF**P**GPSGLKGEKG  EKGAPGETGA**P**GRAGVTGPSG  PQGPSGARGP**P**GLKGDRGDPG  GNCQSQCPGG**P**TPPGGGDLGS  CQSQCPGGPT**P**PGGGDLGSII  STTRCRSSGS**P**CGVTSICCGR  VVAANATVSM**P**PPSSSPPSSV  VAANATVSMP**P**PSSSPPSSVM  AANATVSMPP**P**SSSPPSSVMP  TVSMPPPSSS**P**PSSVMPPPVM  VSMPPPSSSP**P**SSVMPPPVMP  PSSSPPSSVM**P**PPVMPPPSPS  SSSPPSSVMP**P**PVMPPPSPSS  SSPPSSVMPP**P**VMPPPSPSSP  SSVMPPPVMP**P**PSPSSPSPPP  SVMPPPVMPP**P**SPSSPSPPPM  MPPPVMPPPS**P**SSPSPPPMVP  QVGPIGPRGP**P**GPPGSPGQQG  PIGPRGPPGP**P**GSPGQQGYQG  PRGPPGPPGS**P**GQQGYQGLRG  QQGYQGLRGE**P**GDSGPMGPIG  PMGPIGKRGP**P**GPAGIAGKSG  KSGDDGRDGE**P**GPRGGIGPMG  PMGPRGAGGM**P**GMPGPKGHRG  PRGAGGMPGM**P**GPKGHRGFRG  KSGNQGPDGG**P**GPAGPSGPIG  GPDGGPGPAG**P**SGPIGPRGQT  ERGRDGKSGL**P**GLRGVDGLAG  LRGVDGLAGP**P**GPPGPIGSTG  GVDGLAGPPG**P**PGPIGSTGSP  PPGPIGSTGS**P**GFPGTPGSKG  PIGSTGSPGF**P**GTPGSKGDRG  STGSPGFPGT**P**GSKGDRGQSG  LQGPVGLSGQ**P**GVAGENGHPG  QPGVAGENGH**P**GMPGMDGANG  VAGENGHPGM**P**GMDGANGEPG  MPGMDGANGE**P**GASGESGLPG  EPGASGESGL**P**GPSGFPGPRG  ESGLPGPSGF**P**GPRGMPGTAG  PSGFPGPRGM**P**GTAGSPGQAG  PRGMPGTAGS**P**GQAGAKGDGG  DGGPTGEQGR**P**GAPGVKGSSG  PTGEQGRPGA**P**GVKGSSGPPG  APGVKGSSGP**P**GDVGAPGHAG  SSGPPGDVGA**P**GHAGEAGKRG  HAGEAGKRGS**P**GSPGPAGSPG  EAGKRGSPGS**P**GPAGSPGPQG  SPGSPGPAGS**P**GPQGDRGLPG  SPGPQGDRGL**P**GSRGLPGMTG  DRGLPGSRGL**P**GMTGASGAMG  MTGASGAMGI**P**GEKGPSGEPG  IPGEKGPSGE**P**GAKGPTGDTG  DTGRQGNQGT**P**GIAGLPGNPG  NQGTPGIAGL**P**GNPGSDGRPG  TPGIAGLPGN**P**GSDGRPGKDG  LPGNPGSDGR**P**GKDGRPGIRG  SDGRPGKDGR**P**GIRGKDGKQG  LAGLQGRAGP**P**GARGEPGKNG  RAGPPGARGE**P**GKNGAPGEPG  ARGEPGKNGA**P**GEPGAHGEQG  EPGKNGAPGE**P**GAHGEQGDAG  KDGETGAAGP**P**GAAGPTGARG  AAGPTGARGP**P**GPRGQQGFQG  FQGLAGAQGT**P**GEAGKTGERG  GERGAVGATG**P**SGPAGPGGER  GAVGATGPSG**P**AGPGGERGAP  GATGPSGPAG**P**GGERGAPGDR  PAGPGGERGA**P**GDRGNVGPRG  DRGNVGPRGM**P**GERGATGPAG  GMPGERGATG**P**AGPTGSPGVA  GERGATGPAG**P**TGSPGVAGAK  ATGPAGPTGS**P**GVAGAKGQGG  VAGAKGQGGP**P**GPAGLVGLPG  PPGPAGLVGL**P**GERGPKGVGG  ERGERGENGL**P**GPSGLAASKG  SKGERGDMGS**P**GERGSPGPAG  DMGSPGERGS**P**GPAGERGPAG  PAGSQGIQGQ**P**GPPGDAGPAG  SQGIQGQPGP**P**GDAGPAGTKG  PAGTKGDIGF**P**GERGTRGATG  ETGAQGEIGL**P**GSPGQPGLPG  AQGEIGLPGS**P**GQPGLPGPSG  EIGLPGSPGQ**P**GLPGPSGQPG  LPGSPGQPGL**P**GPSGQPGPSG  QPGLPGPSGQ**P**GPSGPAGTAG  KQGVKGARGS**P**GLVGKQGDRG  KQGDRGSDGE**P**GRDGTKGERG  TKGERGEDGP**P**GVSGPTGAPG  PPGVSGPTGA**P**GQQGERGMPG  APGQQGERGM**P**GMVGLRGETG  GQGMKGDGGP**P**GPSGDRGERG  PTGPSGQAGA**P**GQEGAPGKDG  QAGAPGQEGA**P**GKDGLPGLAG  QEGAPGKDGL**P**GLAGRPGERG  KDGLPGLAGR**P**GERGEPGVAG  LAGRPGERGE**P**GVAGRAGSQG  LAGLMGQRGL**P**GAAGPPGDRG  QRGLPGAAGP**P**GDRGERGEPG  PPGDRGERGE**P**GGQGVQGPVG  GQGVQGPVGA**P**GSQGPAGIMG  AKGDKGWTGL**P**GLQGLQGTPG  LPGLQGLQGT**P**GHSGESGPPG  TPGHSGESGP**P**GAPGPRGARG  HSGESGPPGA**P**GPRGARGEAG  EAGGRGSQGP**P**GKDGQPGPSG  SQGPPGKDGQ**P**GPSGRVGPRG  PSGDDGRSGP**P**GPPGPPGPPG  DDGRSGPPGP**P**GPPGPPGNSD  RSGPPGPPGP**P**GPPGNSDYGA  PPGPPGPPGP**P**GNSDYGAAGY  GPMGPIGKRG**P**PGPAGIAGKS  GLRGVDGLAG**P**PGPPGPIGST  VDGLAGPPGP**P**GPIGSTGSPG  GLAGLQGRAG**P**PGARGEPGKN  GVAGAKGQGG**P**PGPAGLVGLP  GSQGIQGQPG**P**PGDAGPAGTK  GTKGERGEDG**P**PGVSGPTGAP  GGQGMKGDGG**P**PGPSGDRGER  GPSGDDGRSG**P**PGPPGPPGPP  GDDGRSGPPG**P**PGPPGPPGNS  GRSGPPGPPG**P**PGPPGNSDYG  GPPGPPGPPG**P**PGNSDYGAAG  LQQLINGQGA**P**APYPDPLEPK  DLIALTARRD**P**CCYHPTCNMS  KIFNIKNDFT**P**EEEEQIRKEN  GCCGGCCGSY**P**NAACHPCSCK  CGSYPNAACH**P**CSCKDRPSYC  ACHPCSCKDR**P**SYCGQQGCYS  EVHRLLERRH**P**PCCMYGRCRR  VHRLLERRHP**P**CCMYGRCRRY  CCMYGRCRRY**P**GCSSASCCQG  KLSLSTRCRI**P**NQKCFQHLDD  SFCKSSKCFS**P**GTFCGIKPGL  FSPGTFCGIK**P**GLCCSVRCFS  DQTYCELYAF**P**SFGAAGFSPF  GCCGGCCGPY**P**NAACHPCGCK  CGPYPNAACH**P**CGCKVGRPPY  CHPCGCKVGR**P**PYCDRPSGGG  HPCGCKVGRP**P**YCDRPSGGGG  KVGRPPYCDR**P**SGGGGSPRDC  FMNVLRRSGC**P**WEPWCGGCWP  FMNVLRRFGC**P**WQPWCGGCWP  CNVTFKTCCG**P**PGDWQCVEAC  NVTFKTCCGP**P**GDWQCVEACP  GVCCGYKLCH**P**CCPHCLKYGC  CCSQDCLVCI**P**CCPNNPCCPI  WPCSQDDQSC**P**WCGFTCCLPN  RCCRTCFGCT**P**CCGGCCPTCG  KLDKKEACYA**P**GTFCGIKPGL  YAPGTFCGIK**P**GLCCSEFCLP  PSGPPKCCKP**P**GSPCRVSSYN  PPKCCKPPGS**P**CRVSSYNCCS  SSGPSLCCLS**P**GSSCSPTSYN  CCLSPGSSCS**P**TSYNCCRSCN  TSYNCCRSCN**P**YSRKCCKRSY  KGKCCKGKGA**P**CTRLMYDCCH  CKDGLTTCLA**P**SECCSEDCEG  SSCSGRDSRC**P**PVCCMGLMCS  SCSGRDSRCP**P**VCCMGLMCSR  DRGCTRTCGG**P**KCTGTCTCTN  EKRQRLCCGF**P**KSCRSRQCKP  PKSCRSRQCK**P**HRCCGRRGCC  IQSPVLWPAA**P**WLVPSQITTC  SQITTCCGYN**P**GTMCPSCMCT  CCGYNPGTMC**P**SCMCTNTCCT  SGYLCCPPGG**P**PCCLYGSCRP  GYLCCPPGGP**P**CCLYGSCRPF  CCLYGSCRPF**P**GCYNALCCRK  CSNPACRVNN**P**HVCCVHPNNV  KICDDDDCIK**P**YGFCSLPILK  NPVGCCCCGV**P**NAACHPCVCK  CCGVPNAACH**P**CVCKNTCCTN  TDKKRGCCSD**P**RCNYDHPEIC  LINTRCCPGK**P**CCRIGGIRCC  PKDYNNYDKP**P**VEKPPVYKPP  NYDKPPVEKP**P**VYKPPVEKPP  PVEKPPVYKP**P**VEKPPVYKPP  PVYKPPVEKP**P**VYKPPKYVPP  PLYMNNMYLP**P**VPPPPVVPTF  YMNNMYLPPV**P**PPPVVPTFFT  MNNMYLPPVP**P**PPVVPTFFTP  NNMYLPPVPP**P**PVVPTFFTPV  NMYLPPVPPP**P**VVPTFFTPVV  QQNQPPDFAN**P**FIIPQNAAAA  GGVCAYGESC**P**SSCNTCYSAQ  CEDGWCCTAA**P**LTGRRGTLPA  SYQYNNYQYS**P**PPPPKKKYYY  YQYNNYQYSP**P**PPPKKKYYYY  QYNNYQYSPP**P**PPKKKYYYYK  YNNYQYSPPP**P**PKKKYYYYKK  NNYQYSPPPP**P**KKKYYYYKKK  SPVEAYYAEV**P**SPAAQAPTAD  VEAYYAEVPS**P**AAQAPTADDA  AEVPSPAAQA**P**TADDATPAQA  RRYLYQWLGA**P**VPYPDPLEPR  TPCRTPCDDC**P**TRCPTTCANG  TPCDDCPTRC**P**TTCANGWECC  ANGWECCKGY**P**CVNKACSGCT  FPSFGPPRRP**P**GFSPFRRFPS  EKKRESPDRP**P**GFSPFRIYYI  RFPTFGPPVV**P**PGFTPFRQSS  RLLRWPPKKP**P**WRLLRWPPKK  DSCCGGCCSD**P**RCKHQCCQHK  DSCCGGCCSD**P**RCRYRCCRYR  CCYDDYCCRR**P**PCTLICCILT  CYDDYCCRRP**P**CTLICCILTC  WQPPCCGGCC**P**PQWCGPDCTS  QPPCCGGCCP**P**QWCGPDCTSP  GGCCPPQWCG**P**DCTSPCCCCP  PQWCGPDCTS**P**CCCCPSTCDP  KCCMRPICTC**P**CCIGPPGICC  WPSCCGGCCS**P**WNCIQLRACP  PWNCIQLRAC**P**CCPNNPCCPC  CIQLRACPCC**P**NNPCCPCARL  KCCMRPICMC**P**CCIGAGGAGI  WSLCTPGCTS**P**GGGSNCSFCC  PGVGGPEGGE**P**GGVGPIGPPG  EPGGVGPIGP**P**GERGAPGNRN  APGERGETGP**P**GPAGFAGPPG  PPGPAGFAGP**P**GADGQPGAKP  MCETEACVGP**P**GANGNPGPAG  CVGPPGANGN**P**GPAGPPGPAG  ANGNPGPAGP**P**GPAGKIVPMQ  CQGFTGLQGL**P**GPPGTSGDQG  FMCETEACVG**P**PGANGNPGPA  GFTGLQGLPG**P**PGTSGDQGAS  PAGPIGSAGP**P**GFPGAPGPKG  SAGPPGFPGA**P**GPKGEIGPVG  SKGESGSKGE**P**GSAGPQGPPG  EPGSAGPQGP**P**GPSGEEGKRG  PIGPAGARGE**P**GNIGFPGPKG  GKGEQGPAGP**P**GFQGLPGPSG  PAGPPGFQGL**P**GPSGTAGEAG  PSGTAGEAGK**P**GERGIPGEFG  EAGKPGERGI**P**GEFGLPGPAG  ERGIPGEFGL**P**GPAGPRGERG  NTGRDGARGA**P**GAVGAPGPAG  ARGAPGAVGA**P**GPAGATGDRG  PAGSRGDGGP**P**GATGFPGAAG  DGGPPGATGF**P**GAAGRTGPPG  PPGPAGITGP**P**GPPGAAGKEG  PAGITGPPGP**P**GAAGKEGLRG  RTGETGASGP**P**GFAGEKGSSG  LPGSRGERGL**P**GVAGAVGEPG  LPGVAGAVGE**P**GPLGIAGPPG  EPGPLGIAGP**P**GARGPPGAVG  PAGKHGNRGE**P**GPAGSVGPVG  LTARRTCCSR**P**TCRMEYPELC  DPCCGYRMCV**P**CCPVCMRYGC  PSGPQGPSGA**P**GPKGVQGPPG  APGPKGVQGP**P**GPQGPRGLTG  PTGARGSAGP**P**GATGFPGAAG  SAGPPGATGF**P**GAAGRGETGP  ETGPAGPAGP**P**GPAGARRAGA  EGNSGGSNGE**P**GSAGPPGPAG  SNGEPGSAGP**P**GPAGLRGLPG  ESGAVGPAGP**P**GSRRSGPPGA  QKTLECLENY**P**GQASQRAHYC  YCQQDATTNC**P**DTYYFGCCPG  ERGMAGPEGK**P**GLQGPRGPPG  KPGLQGPRGP**P**GPVGGHGDPG  PPGPVGGHGD**P**GPPGAPGLAG  PVGGHGDPGP**P**GAPGLAGPAG  LAGHKGEMGE**P**GVPGQSGAPG  HKGEMGEPGV**P**GQSGAPGKEG  EPGVPGQSGA**P**GKEGLIGPKG  NEVALGPAGP**P**GGPGLRGPKG  ALGPAGPPGG**P**GLRGPKGQQG  LRGPKGQQGE**P**GPKGPDGPRG  PDGPRGEIGL**P**GPQGPPGPQG  EIGLPGPQGP**P**GPQGPSGLSI  GPSGLSIQGM**P**GMPGEKGEKG  GLSIQGMPGM**P**GEKGEKGDTG  EKGEKGDTGL**P**GPQGIPGGVG  DTGLPGPQGI**P**GGVGSPGRDG  PQGIPGGVGS**P**GRDGSPGQRG  GVGSPGRDGS**P**GQRGLPGKDG  RDGSPGQRGL**P**GKDGSSGPPG  LPGKDGSSGP**P**GPPGPIGIPG  KDGSSGPPGP**P**GPIGIPGTPG  PPGPPGPIGI**P**GTPGVPGITG  PPGPIGIPGT**P**GVPGITGSMG  PIGIPGTPGV**P**GITGSMGPQG  SMGPQGALGP**P**GVPGAKGERG  PQGALGPPGV**P**GAKGERGERG  ARYTAILNQI**P**SHSSSIRTVQ  SSSIRTVQGP**P**GEPGRPGSPG  IRTVQGPPGE**P**GRPGSPGAPG  VQGPPGEPGR**P**GSPGAPGEQG  PPGEPGRPGS**P**GAPGEQGPPG  EPGRPGSPGA**P**GEQGPPGTPG  SPGAPGEQGP**P**GTPGFPGNAG  APGEQGPPGT**P**GFPGNAGVPG  EQGPPGTPGF**P**GNAGVPGTPG  TPGFPGNAGV**P**GTPGERGLTG  FPGNAGVPGT**P**GERGLTGIKG  LTGIKGEKGN**P**GVGTQGPRGP  GVGTQGPRGP**P**GPAGPSGESR  GPAGPSGESR**P**GSPGPPGSPG  GPSGESRPGS**P**GPPGSPGPRG  GESRPGSPGP**P**GSPGPRGPPG  RPGSPGPPGS**P**GPRGPPGHLG  PPGSPGPRGP**P**GHLGVPGPQG  PRGPPGHLGV**P**GPQGPSGQPG  VPGPQGPSGQ**P**GYCDPSSCSA  GNQEERRDRP**P**SWIPKKPIWS  SEEKKRFEPV**P**PGFTPFRQTT  GACTGWMAGI**P**GHPGHNGAPG  TGWMAGIPGH**P**GHNGAPGRDG  IPGHPGHNGA**P**GRDGRDGTPG  TPGEKGEKGD**P**GLIGPKGDIG  GEKGDPGLIG**P**KGDIGETGVP  GETGVPGAEG**P**RGFPGIQGRK  VPGAEGPRGF**P**GIQGRKGEPG  KEPDALTLLA**P**AAGDTIISLD  DTDLDLEMLA**P**YIPMDDDFQL  VPYEANNRAV**P**DKIDWRESGY  QFGLETESSY**P**YTAVEGQCRY  MTYPPTYKPK**P**SYPPTYKSKP  PTYKPKPSYP**P**TYKSKPTYKP  ITYPPTYKAK**P**SYPSSYKPKK  LTYPPTYKPK**P**SYPPTYKPKP  PTYKPKPSYP**P**TYKPKPSYPP  PSYPPTYKPK**P**SYPPSYKTKK  PTYKPKPSYP**P**SYKTKKTYPS  KTYPSSYKAK**P**SYPPTYKAKP  SSYKAKPSYP**P**TYKAKPSYPP  PSYPPTYKAK**P**SYPPTYKAKP  PTYKAKPSYP**P**TYKAKPSYPP  PTYKAKPSYP**P**TYKAKPTYKA  YKAKPTYKAK**P**TYPSTYKAKP  PTYPSTYKAK**P**SYPPTYKAKP  STYKAKPSYP**P**TYKAKPTYKA  YKAKPTYKAK**P**SYPPTYKAKP  STYKAKPSYP**P**TYKAKPSYPP  PTYPSTYKAK**P**SYPPSYKAKP  STYKAKPSYP**P**SYKAKPSYPP  PSYPPSYKAK**P**SYPPTYKAKP  PSYKAKPSYP**P**TYKAKPTYKA  PTYPSTYKAK**P**SYPASYKAKP  PSYPASYKAK**P**SYPPTYKSKS  ASYKAKPSYP**P**TYKSKSSYPS  LTYKPTYKPK**P**SYPPSYKPKT  PTYKPKPSYP**P**SYKPKTTYPP  PTYKPKISYP**P**TYKAKPSYPA  ISYPPTYKAK**P**SYPATYKAKP  PSYPATYKAK**P**SYPPTYKAKP  ATYKAKPSYP**P**TYKAKPSYPP  PTYKAKPSYP**P**TYKAKPSYKA  YKAKPSYKAK**P**TYPSTYKAKP  PTYKAKPSYP**P**TYKAKPTYPS  PSYPPTYKAK**P**TYPSTYKAKP  PTYPSTYKAK**P**SYPPTYKPKI  STYKAKPSYP**P**TYKPKISYPP  PTYKPKISYP**P**TYKAKPSYPP  ISYPPTYKAK**P**SYPPTYKAKP  PTNPSTYKAK**P**SYPPTYKAKP  YKAKPTYKAK**P**TYPPTYKAKP  PTYKAKPTYP**P**TYKAKPSYPP  PTYPPTYKAK**P**SYPPTYKPKP  PTYKAKPSYP**P**TYKPKPSYPP  PSYPPTYKPK**P**SYPPTYKSKS  PTYKPKPSYP**P**TYKSKSIYPS  LTYPPTYKPK**P**SYPPSYKPKI  PTYKPKPSYP**P**SYKPKITYPS  ITYPSTYKLK**P**SYPPTYKSKT  STYKLKPSYP**P**TYKSKTSYPP  SSYKAKTSYP**P**AYKPTNRYYR  LHAPRDECCE**P**QWCDGACDCC  GACAGWMAGI**P**GHPGHNGTPG  AGWMAGIPGH**P**GHNGTPGRDG  IPGHPGHNGT**P**GRDGRDGTPG  GETGITGIEG**P**RGFPGTPGRK  DTCRQGHSGI**P**GNPGHNGLPG  RQGHSGIPGN**P**GHNGLPGRDG  IPGNPGHNGL**P**GRDGRDGAKG  AKGDKGDAGE**P**GHPGGPGKDG  DKGDAGEPGH**P**GGPGKDGIRG  DAGEPGHPGG**P**GKDGIRGEKG  KDGIRGEKGE**P**GADGRVEAKG  SIGPTGEQGL**P**GETGPQGQKG  STGPLGPKGL**P**GPMGPIGKPG  LPGPMGPIGK**P**GPRGEAGPMG  EAGPMGPQGE**P**GVRGMRGWKG  GTCAGWMAGI**P**GHPGHNGTPG  GDVGMTGAEG**P**RGFPGTPGRK  EDSYGRDPYS**P**SQDPYSPSQD  PYSPSQDPYS**P**SQDPDRRDPY  QDPDRRDPYS**P**SPYDRRGAGS  YHNNHYSYSS**P**PPPPVVSSPP  HNNHYSYSSP**P**PPPVVSSPPP  NNHYSYSSPP**P**PPVVSSPPPP  NHYSYSSPPP**P**PVVSSPPPPY  HYSYSSPPPP**P**VVSSPPPPYY  SPPPPPVVSS**P**PPPYYYYSPP  PPPPPVVSSP**P**PPYYYYSPPP  PPPPVVSSPP**P**PYYYYSPPPP  PPPVVSSPPP**P**YYYYSPPPPV  SPPPPYYYYS**P**PPPVVPPPPS  PPPPYYYYSP**P**PPVVPPPPSY  PPPYYYYSPP**P**PVVPPPPSYY  PPYYYYSPPP**P**VVPPPPSYYY  KLESGNYGRT**P**YKTPPPPTSS  GNYGRTPYKT**P**PPPTSSSPTH  NYGRTPYKTP**P**PPTSSSPTHQ  YGRTPYKTPP**P**PTSSSPTHQE  GRTPYKTPPP**P**TSSSPTHQEI  KTPPPPTSSS**P**THQEIVNGRH  VNGRHDSVLP**P**PSPKTDPIIG  NGRHDSVLPP**P**SPKTDPIIGQ  RHDSVLPPPS**P**KTDPIIGQLT  VGGRHDYVAS**P**PPPKPQDEQR  GGRHDYVASP**P**PPKPQDEQRQ  GRHDYVASPP**P**PKPQDEQRQI  RHDYVASPPP**P**KPQDEQRQII  KCGGCKKDRK**P**CSYHADCCNC  CNCCLSGICA**P**STNWILPGCS  GNAKCFSPGG**P**SFCKANGKPC  GPSFCKANGK**P**CSYHADCCNC  CNCCLSGICK**P**STNVILPGCS  EDAKCFSPGG**P**SFCKADEKPC  GPSFCKADEK**P**CEYHADCCNC  SRAARVKNRG**P**SFCKADEKPC  GPSFCKADEK**P**CKYHADCCNC  CNCCLGGICK**P**STSWIGCSTN  EKKREAPERP**P**GFTPFRIYYI  LFTTSCLAQA**P**APSPTTTVTP  TTSCLAQAPA**P**SPTTTVTPPP  SCLAQAPAPS**P**TTTVTPPPVA  APSPTTTVTP**P**PVATPPPAAT  PSPTTTVTPP**P**VATPPPAATP  SGYGRGTNLP**P**PSPASSPPSK  GYGRGTNLPP**P**SPASSPPSKE  GRGTNLPPPS**P**ASSPPSKEVS  NLPPPSPASS**P**PSKEVSNSVS  LPPPSPASSP**P**SKEVSNSVSP  EKYWNRKPLS**P**PSPKPADGHR  KYWNRKPLSP**P**SPKPADGHRP  WNRKPLSPPS**P**KPADGHRPLQ  SGYGRGANLP**P**PSPASSPPSK  GYGRGANLPP**P**SPASSPPSKE  GRGANLPPPS**P**ASSPPSKEVS  ERYWNRKPLS**P**PSPKPADGQR  RYWNRKPLSP**P**SPKPADGQRP  WNRKPLSPPS**P**KPADGQRPLH  SANPKHDPGV**P**PSATGQRVVG  ANPKHDPGVP**P**SATGQRVVGR  CSDDWQYCES**P**TDCCSWDCDV  DAEKQQKRLC**P**DYTDPCSHAH  QKRLCPDYTD**P**CSHAHECCSW  NAGFCRFGCT**P**CCYYCCPTCG  VLIAGVTGQA**P**TSPPTATPAP  AGVTGQAPTS**P**PTATPAPPTP  GVTGQAPTSP**P**TATPAPPTPT  QAPTSPPTAT**P**APPTPTTPPP  PTSPPTATPA**P**PTPTTPPPAA  VGNVAAQTEA**P**APSPTSDAAM  NVAAQTEAPA**P**SPTSDAAMFV  AAQTEAPAPS**P**TSDAAMFVPA  VHQTVAAVDA**P**APSPTSDASS  QTVAAVDAPA**P**SPTSDASSFI  VAAVDAPAPS**P**TSDASSFIPT  SVVASAQSEA**P**APSPTSGSSA  VASAQSEAPA**P**SPTSGSSAIS  SAQSEAPAPS**P**TSGSSAISAS  LIASSAIAQA**P**GPAPTRSPLP  ASSAIAQAPG**P**APTRSPLPSP  SAIAQAPGPA**P**TRSPLPSPAQ  QAPGPAPTRS**P**LPSPAQPPRT  PAPTRSPLPS**P**AQPPRTAAPT  FLATSCLAQA**P**APAPTTVTPP  ATSCLAQAPA**P**APTTVTPPPT  SCLAQAPAPA**P**TTVTPPPTAL  PAPAPTTVTP**P**PTALPPVTAE  APAPTTVTPP**P**TALPPVTAET  AVQQAAAVEA**P**APSPTSDASL  QQAAAVEAPA**P**SPTSDASLAI  AAAVEAPAPS**P**TSDASLAIPA  NECCWGGCGH**P**CRHPGKRSKL  ACIKLEKDYQ**P**GITFIVVQKR  ADALDLEMLA**P**YISMDDDFQL  LFATSALAQA**P**APTPTATPPP  ATSALAQAPA**P**TPTATPPPAT  SALAQAPAPT**P**TATPPPATPP  QAPAPTPTAT**P**PPATPPPVAT  APAPTPTATP**P**PATPPPVATP  PAPTPTATPP**P**ATPPPVATPP  TPTATPPPAT**P**PPVATPPPVA  PTATPPPATP**P**PVATPPPVAT  TATPPPATPP**P**VATPPPVATP |

(2) List of the 3,505 peptide samples in the negative subset $\mathbb{S}^{-}$(P)

| Sample # | Protein ID | Site | Sequences |
| --- | --- | --- | --- |
| 1  2  3  4  5  6  7  8  9  10  11  12  13  14  15  16  17  18  19  20  21  22  23  24  25  26  27  28  29  30  31  32  33  34  35  36  37  38  39  40  41  42  43  44  45  46  47  48  49  50  51  52  53  54  55  56  57  58  59  60  61  62  63  64  65  66  67  68  69  70  71  72  73  74  75  76  77  78  79  80  81  82  83  84  85  86  87  88  89  90  91  92  93  94  95  96  97  98  99  100  101  102  103  104  105  106  107  108  109  110  111  112  113  114  115  116  117  118  119  120  121  122  123  124  125  126  127  128  129  130  131  132  133  134  135  136  137  138  139  140  141  142  143  144  145  146  147  148  149  150  151  152  153  154  155  156  157  158  159  160  161  162  163  164  165  166  167  168  169  170  171  172  173  174  175  176  177  178  179  180  181  182  183  184  185  186  187  188  189  190  191  192  193  194  195  196  197  198  199  200  201  202  203  204  205  206  207  208  209  210  211  212  213  214  215  216  217  218  219  220  221  222  223  224  225  226  227  228  229  230  231  232  233  234  235  236  237  238  239  240  241  242  243  244  245  246  247  248  249  250  251  252  253  254  255  256  257  258  259  260  261  262  263  264  265  266  267  268  269  270  271  272  273  274  275  276  277  278  279  280  281  282  283  284  285  286  287  288  289  290  291  292  293  294  295  296  297  298  299  300  301  302  303  304  305  306  307  308  309  310  311  312  313  314  315  316  317  318  319  320  321  322  323  324  325  326  327  328  329  330  331  332  333  334  335  336  337  338  339  340  341  342  343  344  345  346  347  348  349  350  351  352  353  354  355  356  357  358  359  360  361  362  363  364  365  366  367  368  369  370  371  372  373  374  375  376  377  378  379  380  381  382  383  384  385  386  387  388  389  390  391  392  393  394  395  396  397  398  399  400  401  402  403  404  405  406  407  408  409  410  411  412  413  414  415  416  417  418  419  420  421  422  423  424  425  426  427  428  429  430  431  432  433  434  435  436  437  438  439  440  441  442  443  444  445  446  447  448  449  450  451  452  453  454  455  456  457  458  459  460  461  462  463  464  465  466  467  468  469  470  471  472  473  474  475  476  477  478  479  480  481  482  483  484  485  486  487  488  489  490  491  492  493  494  495  496  497  498  499  500  501  502  503  504  505  506  507  508  509  510  511  512  513  514  515  516  517  518  519  520  521  522  523  524  525  526  527  528  529  530  531  532  533  534  535  536  537  538  539  540  541  542  543  544  545  546  547  548  549  550  551  552  553  554  555  556  557  558  559  560  561  562  563  564  565  566  567  568  569  570  571  572  573  574  575  576  577  578  579  580  581  582  583  584  585  586  587  588  589  590  591  592  593  594  595  596  597  598  599  600  601  602  603  604  605  606  607  608  609  610  611  612  613  614  615  616  617  618  619  620  621  622  623  624  625  626  627  628  629  630  631  632  633  634  635  636  637  638  639  640  641  642  643  644  645  646  647  648  649  650  651  652  653  654  655  656  657  658  659  660  661  662  663  664  665  666  667  668  669  670  671  672  673  674  675  676  677  678  679  680  681  682  683  684  685  686  687  688  689  690  691  692  693  694  695  696  697  698  699  700  701  702  703  704  705  706  707  708  709  710  711  712  713  714  715  716  717  718  719  720  721  722  723  724  725  726  727  728  729  730  731  732  733  734  735  736  737  738  739  740  741  742  743  744  745  746  747  748  749  750  751  752  753  754  755  756  757  758  759  760  761  762  763  764  765  766  767  768  769  770  771  772  773  774  775  776  777  778  779  780  781  782  783  784  785  786  787  788  789  790  791  792  793  794  795  796  797  798  799  800  801  802  803  804  805  806  807  808  809  810  811  812  813  814  815  816  817  818  819  820  821  822  823  824  825  826  827  828  829  830  831  832  833  834  835  836  837  838  839  840  841  842  843  844  845  846  847  848  849  850  851  852  853  854  855  856  857  858  859  860  861  862  863  864  865  866  867  868  869  870  871  872  873  874  875  876  877  878  879  880  881  882  883  884  885  886  887  888  889  890  891  892  893  894  895  896  897  898  899  900  901  902  903  904  905  906  907  908  909  910  911  912  913  914  915  916  917  918  919  920  921  922  923  924  925  926  927  928  929  930  931  932  933  934  935  936  937  938  939  940  941  942  943  944  945  946  947  948  949  950  951  952  953  954  955  956  957  958  959  960  961  962  963  964  965  966  967  968  969  970  971  972  973  974  975  976  977  978  979  980  981  982  983  984  985  986  987  988  989  990  991  992  993  994  995  996  997  998  999  1000  1001  1002  1003  1004  1005  1006  1007  1008  1009  1010  1011  1012  1013  1014  1015  1016  1017  1018  1019  1020  1021  1022  1023  1024  1025  1026  1027  1028  1029  1030  1031  1032  1033  1034  1035  1036  1037  1038  1039  1040  1041  1042  1043  1044  1045  1046  1047  1048  1049  1050  1051  1052  1053  1054  1055  1056  1057  1058  1059  1060  1061  1062  1063  1064  1065  1066  1067  1068  1069  1070  1071  1072  1073  1074  1075  1076  1077  1078  1079  1080  1081  1082  1083  1084  1085  1086  1087  1088  1089  1090  1091  1092  1093  1094  1095  1096  1097  1098  1099  1100  1101  1102  1103  1104  1105  1106  1107  1108  1109  1110  1111  1112  1113  1114  1115  1116  1117  1118  1119  1120  1121  1122  1123  1124  1125  1126  1127  1128  1129  1130  1131  1132  1133  1134  1135  1136  1137  1138  1139  1140  1141  1142  1143  1144  1145  1146  1147  1148  1149  1150  1151  1152  1153  1154  1155  1156  1157  1158  1159  1160  1161  1162  1163  1164  1165  1166  1167  1168  1169  1170  1171  1172  1173  1174  1175  1176  1177  1178  1179  1180  1181  1182  1183  1184  1185  1186  1187  1188  1189  1190  1191  1192  1193  1194  1195  1196  1197  1198  1199  1200  1201  1202  1203  1204  1205  1206  1207  1208  1209  1210  1211  1212  1213  1214  1215  1216  1217  1218  1219  1220  1221  1222  1223  1224  1225  1226  1227  1228  1229  1230  1231  1232  1233  1234  1235  1236  1237  1238  1239  1240  1241  1242  1243  1244  1245  1246  1247  1248  1249  1250  1251  1252  1253  1254  1255  1256  1257  1258  1259  1260  1261  1262  1263  1264  1265  1266  1267  1268  1269  1270  1271  1272  1273  1274  1275  1276  1277  1278  1279  1280  1281  1282  1283  1284  1285  1286  1287  1288  1289  1290  1291  1292  1293  1294  1295  1296  1297  1298  1299  1300  1301  1302  1303  1304  1305  1306  1307  1308  1309  1310  1311  1312  1313  1314  1315  1316  1317  1318  1319  1320  1321  1322  1323  1324  1325  1326  1327  1328  1329  1330  1331  1332  1333  1334  1335  1336  1337  1338  1339  1340  1341  1342  1343  1344  1345  1346  1347  1348  1349  1350  1351  1352  1353  1354  1355  1356  1357  1358  1359  1360  1361  1362  1363  1364  1365  1366  1367  1368  1369  1370  1371  1372  1373  1374  1375  1376  1377  1378  1379  1380  1381  1382  1383  1384  1385  1386  1387  1388  1389  1390  1391  1392  1393  1394  1395  1396  1397  1398  1399  1400  1401  1402  1403  1404  1405  1406  1407  1408  1409  1410  1411  1412  1413  1414  1415  1416  1417  1418  1419  1420  1421  1422  1423  1424  1425  1426  1427  1428  1429  1430  1431  1432  1433  1434  1435  1436  1437  1438  1439  1440  1441  1442  1443  1444  1445  1446  1447  1448  1449  1450  1451  1452  1453  1454  1455  1456  1457  1458  1459  1460  1461  1462  1463  1464  1465  1466  1467  1468  1469  1470  1471  1472  1473  1474  1475  1476  1477  1478  1479  1480  1481  1482  1483  1484  1485  1486  1487  1488  1489  1490  1491  1492  1493  1494  1495  1496  1497  1498  1499  1500  1501  1502  1503  1504  1505  1506  1507  1508  1509  1510  1511  1512  1513  1514  1515  1516  1517  1518  1519  1520  1521  1522  1523  1524  1525  1526  1527  1528  1529  1530  1531  1532  1533  1534  1535  1536  1537  1538  1539  1540  1541  1542  1543  1544  1545  1546  1547  1548  1549  1550  1551  1552  1553  1554  1555  1556  1557  1558  1559  1560  1561  1562  1563  1564  1565  1566  1567  1568  1569  1570  1571  1572  1573  1574  1575  1576  1577  1578  1579  1580  1581  1582  1583  1584  1585  1586  1587  1588  1589  1590  1591  1592  1593  1594  1595  1596  1597  1598  1599  1600  1601  1602  1603  1604  1605  1606  1607  1608  1609  1610  1611  1612  1613  1614  1615  1616  1617  1618  1619  1620  1621  1622  1623  1624  1625  1626  1627  1628  1629  1630  1631  1632  1633  1634  1635  1636  1637  1638  1639  1640  1641  1642  1643  1644  1645  1646  1647  1648  1649  1650  1651  1652  1653  1654  1655  1656  1657  1658  1659  1660  1661  1662  1663  1664  1665  1666  1667  1668  1669  1670  1671  1672  1673  1674  1675  1676  1677  1678  1679  1680  1681  1682  1683  1684  1685  1686  1687  1688  1689  1690  1691  1692  1693  1694  1695  1696  1697  1698  1699  1700  1701  1702  1703  1704  1705  1706  1707  1708  1709  1710  1711  1712  1713  1714  1715  1716  1717  1718  1719  1720  1721  1722  1723  1724  1725  1726  1727  1728  1729  1730  1731  1732  1733  1734  1735  1736  1737  1738  1739  1740  1741  1742  1743  1744  1745  1746  1747  1748  1749  1750  1751  1752  1753  1754  1755  1756  1757  1758  1759  1760  1761  1762  1763  1764  1765  1766  1767  1768  1769  1770  1771  1772  1773  1774  1775  1776  1777  1778  1779  1780  1781  1782  1783  1784  1785  1786  1787  1788  1789  1790  1791  1792  1793  1794  1795  1796  1797  1798  1799  1800  1801  1802  1803  1804  1805  1806  1807  1808  1809  1810  1811  1812  1813  1814  1815  1816  1817  1818  1819  1820  1821  1822  1823  1824  1825  1826  1827  1828  1829  1830  1831  1832  1833  1834  1835  1836  1837  1838  1839  1840  1841  1842  1843  1844  1845  1846  1847  1848  1849  1850  1851  1852  1853  1854  1855  1856  1857  1858  1859  1860  1861  1862  1863  1864  1865  1866  1867  1868  1869  1870  1871  1872  1873  1874  1875  1876  1877  1878  1879  1880  1881  1882  1883  1884  1885  1886  1887  1888  1889  1890  1891  1892  1893  1894  1895  1896  1897  1898  1899  1900  1901  1902  1903  1904  1905  1906  1907  1908  1909  1910  1911  1912  1913  1914  1915  1916  1917  1918  1919  1920  1921  1922  1923  1924  1925  1926  1927  1928  1929  1930  1931  1932  1933  1934  1935  1936  1937  1938  1939  1940  1941  1942  1943  1944  1945  1946  1947  1948  1949  1950  1951  1952  1953  1954  1955  1956  1957  1958  1959  1960  1961  1962  1963  1964  1965  1966  1967  1968  1969  1970  1971  1972  1973  1974  1975  1976  1977  1978  1979  1980  1981  1982  1983  1984  1985  1986  1987  1988  1989  1990  1991  1992  1993  1994  1995  1996  1997  1998  1999  2000  2001  2002  2003  2004  2005  2006  2007  2008  2009  2010  2011  2012  2013  2014  2015  2016  2017  2018  2019  2020  2021  2022  2023  2024  2025  2026  2027  2028  2029  2030  2031  2032  2033  2034  2035  2036  2037  2038  2039  2040  2041  2042  2043  2044  2045  2046  2047  2048  2049  2050  2051  2052  2053  2054  2055  2056  2057  2058  2059  2060  2061  2062  2063  2064  2065  2066  2067  2068  2069  2070  2071  2072  2073  2074  2075  2076  2077  2078  2079  2080  2081  2082  2083  2084  2085  2086  2087  2088  2089  2090  2091  2092  2093  2094  2095  2096  2097  2098  2099  2100  2101  2102  2103  2104  2105  2106  2107  2108  2109  2110  2111  2112  2113  2114  2115  2116  2117  2118  2119  2120  2121  2122  2123  2124  2125  2126  2127  2128  2129  2130  2131  2132  2133  2134  2135  2136  2137  2138  2139  2140  2141  2142  2143  2144  2145  2146  2147  2148  2149  2150  2151  2152  2153  2154  2155  2156  2157  2158  2159  2160  2161  2162  2163  2164  2165  2166  2167  2168  2169  2170  2171  2172  2173  2174  2175  2176  2177  2178  2179  2180  2181  2182  2183  2184  2185  2186  2187  2188  2189  2190  2191  2192  2193  2194  2195  2196  2197  2198  2199  2200  2201  2202  2203  2204  2205  2206  2207  2208  2209  2210  2211  2212  2213  2214  2215  2216  2217  2218  2219  2220  2221  2222  2223  2224  2225  2226  2227  2228  2229  2230  2231  2232  2233  2234  2235  2236  2237  2238  2239  2240  2241  2242  2243  2244  2245  2246  2247  2248  2249  2250  2251  2252  2253  2254  2255  2256  2257  2258  2259  2260  2261  2262  2263  2264  2265  2266  2267  2268  2269  2270  2271  2272  2273  2274  2275  2276  2277  2278  2279  2280  2281  2282  2283  2284  2285  2286  2287  2288  2289  2290  2291  2292  2293  2294  2295  2296  2297  2298  2299  2300  2301  2302  2303  2304  2305  2306  2307  2308  2309  2310  2311  2312  2313  2314  2315  2316  2317  2318  2319  2320  2321  2322  2323  2324  2325  2326  2327  2328  2329  2330  2331  2332  2333  2334  2335  2336  2337  2338  2339  2340  2341  2342  2343  2344  2345  2346  2347  2348  2349  2350  2351  2352  2353  2354  2355  2356  2357  2358  2359  2360  2361  2362  2363  2364  2365  2366  2367  2368  2369  2370  2371  2372  2373  2374  2375  2376  2377  2378  2379  2380  2381  2382  2383  2384  2385  2386  2387  2388  2389  2390  2391  2392  2393  2394  2395  2396  2397  2398  2399  2400  2401  2402  2403  2404  2405  2406  2407  2408  2409  2410  2411  2412  2413  2414  2415  2416  2417  2418  2419  2420  2421  2422  2423  2424  2425  2426  2427  2428  2429  2430  2431  2432  2433  2434  2435  2436  2437  2438  2439  2440  2441  2442  2443  2444  2445  2446  2447  2448  2449  2450  2451  2452  2453  2454  2455  2456  2457  2458  2459  2460  2461  2462  2463  2464  2465  2466  2467  2468  2469  2470  2471  2472  2473  2474  2475  2476  2477  2478  2479  2480  2481  2482  2483  2484  2485  2486  2487  2488  2489  2490  2491  2492  2493  2494  2495  2496  2497  2498  2499  2500  2501  2502  2503  2504  2505  2506  2507  2508  2509  2510  2511  2512  2513  2514  2515  2516  2517  2518  2519  2520  2521  2522  2523  2524  2525  2526  2527  2528  2529  2530  2531  2532  2533  2534  2535  2536  2537  2538  2539  2540  2541  2542  2543  2544  2545  2546  2547  2548  2549  2550  2551  2552  2553  2554  2555  2556  2557  2558  2559  2560  2561  2562  2563  2564  2565  2566  2567  2568  2569  2570  2571  2572  2573  2574  2575  2576  2577  2578  2579  2580  2581  2582  2583  2584  2585  2586  2587  2588  2589  2590  2591  2592  2593  2594  2595  2596  2597  2598  2599  2600  2601  2602  2603  2604  2605  2606  2607  2608  2609  2610  2611  2612  2613  2614  2615  2616  2617  2618  2619  2620  2621  2622  2623  2624  2625  2626  2627  2628  2629  2630  2631  2632  2633  2634  2635  2636  2637  2638  2639  2640  2641  2642  2643  2644  2645  2646  2647  2648  2649  2650  2651  2652  2653  2654  2655  2656  2657  2658  2659  2660  2661  2662  2663  2664  2665  2666  2667  2668  2669  2670  2671  2672  2673  2674  2675  2676  2677  2678  2679  2680  2681  2682  2683  2684  2685  2686  2687  2688  2689  2690  2691  2692  2693  2694  2695  2696  2697  2698  2699  2700  2701  2702  2703  2704  2705  2706  2707  2708  2709  2710  2711  2712  2713  2714  2715  2716  2717  2718  2719  2720  2721  2722  2723  2724  2725  2726  2727  2728  2729  2730  2731  2732  2733  2734  2735  2736  2737  2738  2739  2740  2741  2742  2743  2744  2745  2746  2747  2748  2749  2750  2751  2752  2753  2754  2755  2756  2757  2758  2759  2760  2761  2762  2763  2764  2765  2766  2767  2768  2769  2770  2771  2772  2773  2774  2775  2776  2777  2778  2779  2780  2781  2782  2783  2784  2785  2786  2787  2788  2789  2790  2791  2792  2793  2794  2795  2796  2797  2798  2799  2800  2801  2802  2803  2804  2805  2806  2807  2808  2809  2810  2811  2812  2813  2814  2815  2816  2817  2818  2819  2820  2821  2822  2823  2824  2825  2826  2827  2828  2829  2830  2831  2832  2833  2834  2835  2836  2837  2838  2839  2840  2841  2842  2843  2844  2845  2846  2847  2848  2849  2850  2851  2852  2853  2854  2855  2856  2857  2858  2859  2860  2861  2862  2863  2864  2865  2866  2867  2868  2869  2870  2871  2872  2873  2874  2875  2876  2877  2878  2879  2880  2881  2882  2883  2884  2885  2886  2887  2888  2889  2890  2891  2892  2893  2894  2895  2896  2897  2898  2899  2900  2901  2902  2903  2904  2905  2906  2907  2908  2909  2910  2911  2912  2913  2914  2915  2916  2917  2918  2919  2920  2921  2922  2923  2924  2925  2926  2927  2928  2929  2930  2931  2932  2933  2934  2935  2936  2937  2938  2939  2940  2941  2942  2943  2944  2945  2946  2947  2948  2949  2950  2951  2952  2953  2954  2955  2956  2957  2958  2959  2960  2961  2962  2963  2964  2965  2966  2967  2968  2969  2970  2971  2972  2973  2974  2975  2976  2977  2978  2979  2980  2981  2982  2983  2984  2985  2986  2987  2988  2989  2990  2991  2992  2993  2994  2995  2996  2997  2998  2999  3000  3001  3002  3003  3004  3005  3006  3007  3008  3009  3010  3011  3012  3013  3014  3015  3016  3017  3018  3019  3020  3021  3022  3023  3024  3025  3026  3027  3028  3029  3030  3031  3032  3033  3034  3035  3036  3037  3038  3039  3040  3041  3042  3043  3044  3045  3046  3047  3048  3049  3050  3051  3052  3053  3054  3055  3056  3057  3058  3059  3060  3061  3062  3063  3064  3065  3066  3067  3068  3069  3070  3071  3072  3073  3074  3075  3076  3077  3078  3079  3080  3081  3082  3083  3084  3085  3086  3087  3088  3089  3090  3091  3092  3093  3094  3095  3096  3097  3098  3099  3100  3101  3102  3103  3104  3105  3106  3107  3108  3109  3110  3111  3112  3113  3114  3115  3116  3117  3118  3119  3120  3121  3122  3123  3124  3125  3126  3127  3128  3129  3130  3131  3132  3133  3134  3135  3136  3137  3138  3139  3140  3141  3142  3143  3144  3145  3146  3147  3148  3149  3150  3151  3152  3153  3154  3155  3156  3157  3158  3159  3160  3161  3162  3163  3164  3165  3166  3167  3168  3169  3170  3171  3172  3173  3174  3175  3176  3177  3178  3179  3180  3181  3182  3183  3184  3185  3186  3187  3188  3189  3190  3191  3192  3193  3194  3195  3196  3197  3198  3199  3200  3201  3202  3203  3204  3205  3206  3207  3208  3209  3210  3211  3212  3213  3214  3215  3216  3217  3218  3219  3220  3221  3222  3223  3224  3225  3226  3227  3228  3229  3230  3231  3232  3233  3234  3235  3236  3237  3238  3239  3240  3241  3242  3243  3244  3245  3246  3247  3248  3249  3250  3251  3252  3253  3254  3255  3256  3257  3258  3259  3260  3261  3262  3263  3264  3265  3266  3267  3268  3269  3270  3271  3272  3273  3274  3275  3276  3277  3278  3279  3280  3281  3282  3283  3284  3285  3286  3287  3288  3289  3290  3291  3292  3293  3294  3295  3296  3297  3298  3299  3300  3301  3302  3303  3304  3305  3306  3307  3308  3309  3310  3311  3312  3313  3314  3315  3316  3317  3318  3319  3320  3321  3322  3323  3324  3325  3326  3327  3328  3329  3330  3331  3332  3333  3334  3335  3336  3337  3338  3339  3340  3341  3342  3343  3344  3345  3346  3347  3348  3349  3350  3351  3352  3353  3354  3355  3356  3357  3358  3359  3360  3361  3362  3363  3364  3365  3366  3367  3368  3369  3370  3371  3372  3373  3374  3375  3376  3377  3378  3379  3380  3381  3382  3383  3384  3385  3386  3387  3388  3389  3390  3391  3392  3393  3394  3395  3396  3397  3398  3399  3400  3401  3402  3403  3404  3405  3406  3407  3408  3409  3410  3411  3412  3413  3414  3415  3416  3417  3418  3419  3420  3421  3422  3423  3424  3425  3426  3427  3428  3429  3430  3431  3432  3433  3434  3435  3436  3437  3438  3439  3440  3441  3442  3443  3444  3445  3446  3447  3448  3449  3450  3451  3452  3453  3454  3455  3456  3457  3458  3459  3460  3461  3462  3463  3464  3465  3466  3467  3468  3469  3470  3471  3472  3473  3474  3475  3476  3477  3478  3479  3480  3481  3482  3483  3484  3485  3486  3487  3488  3489  3490  3491  3492  3493  3494  3495  3496  3497  3498  3499  3500  3501  3502  3503  3504  3505 | A1X158  A1X158  A1X158  A1X158  A1X158  A1X158  A1X158  A1X158  A1X158  A1X158  A1X158  A1X158  A1X158  A1X158  A1X158  A1X158  A1X158  A1X158  A1X158  A1X158  A1X158  A1X158  A1X158  A1X158  A1X158  A1X158  A1X158  A1X158  A1X158  A1X158  A1X158  A1X158  A1X158  A1X158  A1X158  A1X158  A1X158  A1X158  A1X158  A1X158  A1X158  A6YR20  A6YR20  B2CS62  B2KPN7  B2KPN7  B4YSU8  B4YSU8  C3VVN6  C3VVN6  C3VVN6  C4PWC4  C4PWC4  C4PWC4  C4PWC4  D2Y168  D2Y168  D2Y168  D2Y168  D2Y171  D5KR58  D5KR58  D5KR58  O82337  P00057  P00057  P00057  P00057  P00057  P00057  P00057  P00877  P00877  P00877  P00877  P00877  P00877  P00877  P00877  P00877  P00877  P00877  P00877  P00877  P00877  P00877  P00877  P00877  P00877  P00877  P01042  P01042  P01042  P01042  P01042  P01042  P01042  P01042  P01042  P01042  P01042  P01042  P01042  P01042  P01042  P01042  P01042  P01042  P01042  P01042  P01042  P01042  P01042  P01042  P01042  P01042  P01042  P01042  P01042  P01042  P01042  P01042  P01042  P01042  P01042  P01042  P01042  P01042  P01523  P01523  P01523  P01523  P01523  P02453  P02453  P02453  P02453  P02453  P02453  P02453  P02453  P02453  P02453  P02453  P02453  P02453  P02453  P02453  P02453  P02453  P02453  P02453  P02453  P02453  P02453  P02453  P02453  P02453  P02453  P02453  P02453  P02453  P02453  P02453  P02453  P02453  P02453  P02453  P02453  P02453  P02453  P02453  P02453  P02453  P02453  P02453  P02453  P02453  P02453  P02453  P02453  P02453  P02453  P02453  P02453  P02453  P02453  P02453  P02453  P02453  P02453  P02453  P02453  P02453  P02453  P02453  P02453  P02453  P02453  P02453  P02453  P02453  P02453  P02453  P02453  P02453  P02453  P02453  P02453  P02453  P02453  P02453  P02453  P02453  P02453  P02453  P02453  P02453  P02453  P02453  P02453  P02453  P02453  P02453  P02453  P02453  P02453  P02453  P02453  P02453  P02453  P02453  P02453  P02453  P02453  P02453  P02453  P02453  P02453  P02453  P02453  P02453  P02453  P02453  P02453  P02453  P02453  P02453  P02453  P02453  P02453  P02453  P02453  P02453  P02453  P02453  P02453  P02453  P02453  P02453  P02453  P02453  P02453  P02453  P02453  P02453  P02453  P02453  P02453  P02453  P02453  P02453  P02453  P02453  P02453  P02453  P02453  P02453  P02453  P02453  P02453  P02453  P02453  P02453  P02453  P02453  P02453  P02453  P02453  P02453  P02453  P02453  P02453  P02453  P02453  P02453  P02453  P02453  P02453  P02453  P02453  P02453  P02453  P02453  P02453  P02453  P02453  P02453  P02453  P02453  P02453  P02453  P02453  P02453  P02453  P02453  P02453  P02453  P02453  P02453  P02453  P02453  P02453  P02453  P02453  P02453  P02453  P02453  P02453  P02453  P02453  P02453  P02453  P02453  P02453  P02453  P02453  P02453  P02453  P02453  P02453  P02453  P02453  P02453  P02453  P02453  P02453  P02453  P02453  P02453  P02453  P02453  P02453  P02453  P02453  P02453  P02453  P02453  P02453  P02453  P02453  P02453  P02453  P02453  P02453  P02453  P02453  P02453  P02453  P02453  P02453  P02453  P02453  P02453  P02453  P02453  P02453  P02453  P02453  P02453  P02453  P02453  P02453  P02453  P02453  P02453  P02453  P02453  P02453  P02453  P02453  P02453  P02453  P02453  P02453  P02453  P02453  P02453  P02453  P02453  P02453  P02453  P02453  P02453  P02453  P02453  P02453  P02453  P02453  P02453  P02453  P02457  P02457  P02457  P02457  P02457  P02457  P02457  P02457  P02457  P02457  P02457  P02457  P02457  P02457  P02457  P02457  P02457  P02457  P02457  P02457  P02457  P02457  P02457  P02457  P02457  P02457  P02457  P02457  P02457  P02457  P02457  P02457  P02457  P02457  P02457  P02457  P02457  P02457  P02457  P02457  P02457  P02457  P02457  P02457  P02457  P02457  P02457  P02457  P02457  P02457  P02457  P02457  P02457  P02457  P02457  P02457  P02457  P02457  P02457  P02457  P02457  P02457  P02457  P02457  P02457  P02457  P02457  P02457  P02457  P02457  P02457  P02457  P02457  P02457  P02457  P02457  P02457  P02457  P02457  P02457  P02457  P02457  P02457  P02457  P02457  P02457  P02457  P02457  P02457  P02457  P02457  P02457  P02457  P02457  P02457  P02457  P02457  P02457  P02457  P02457  P02457  P02457  P02457  P02457  P02457  P02457  P02457  P02457  P02457  P02457  P02457  P02457  P02457  P02457  P02457  P02457  P02457  P02457  P02457  P02457  P02457  P02457  P02457  P02457  P02457  P02457  P02457  P02457  P02457  P02457  P02457  P02457  P02457  P02457  P02457  P02457  P02457  P02457  P02457  P02457  P02457  P02457  P02457  P02457  P02457  P02457  P02457  P02457  P02457  P02457  P02467  P02467  P02467  P02467  P02467  P02467  P02467  P02467  P02467  P02467  P02467  P02467  P02467  P02467  P02467  P02467  P02467  P02467  P02467  P02467  P02467  P02467  P02467  P02467  P02467  P02467  P02467  P02467  P02467  P02467  P02467  P02467  P02467  P02467  P02467  P02467  P02467  P02467  P02467  P02467  P02467  P02467  P02467  P02467  P02467  P02467  P02467  P02467  P02467  P02467  P02467  P02467  P02467  P02467  P02467  P02467  P02467  P02467  P02467  P02467  P02467  P02467  P02467  P02467  P02467  P02467  P02467  P02467  P02467  P02467  P02467  P02467  P02467  P02467  P02467  P02467  P02467  P02467  P02467  P02467  P02467  P02467  P02467  P02467  P02467  P02467  P02467  P02467  P02467  P02467  P02467  P02467  P02467  P02467  P02467  P02467  P02467  P02467  P02467  P02467  P02467  P02467  P02467  P02467  P02467  P02467  P02467  P02467  P02467  P02467  P02467  P02467  P02467  P02467  P02467  P02467  P02467  P02467  P02467  P02467  P02467  P02467  P02467  P02467  P02467  P02467  P02467  P02467  P02467  P02467  P02467  P02467  P02467  P02467  P02467  P02467  P02467  P02467  P02467  P02467  P02467  P02467  P02467  P02467  P02467  P02467  P02467  P02467  P02467  P02467  P02467  P02467  P02467  P02467  P02467  P02467  P02467  P02467  P02467  P02467  P02467  P02467  P02467  P02467  P02467  P02467  P02467  P02467  P02467  P02467  P02467  P02467  P02467  P02467  P02467  P02467  P02467  P02467  P02467  P02467  P02467  P02467  P02467  P02467  P02467  P02467  P02467  P02467  P02467  P02467  P02467  P02467  P02467  P02467  P02467  P02467  P02467  P02467  P02467  P02467  P02467  P02467  P02467  P02467  P02467  P02467  P02467  P02467  P02467  P02467  P02467  P02467  P02467  P02467  P02467  P02467  P02467  P02467  P02467  P02467  P02467  P02467  P02467  P02467  P02467  P02467  P02467  P02467  P02467  P02467  P02467  P02467  P02467  P02467  P02467  P02467  P02467  P02467  P02745  P02745  P02745  P02745  P02745  P02745  P02745  P02745  P02745  P02745  P02745  P02745  P02745  P02745  P02745  P02747  P02747  P02747  P02747  P02747  P02747  P02747  P02747  P02747  P02747  P02747  P02747  P02747  P02747  P02747  P02747  P02747  P02747  P02747  P02820  P02820  P02820  P02820  P02820  P02820  P02820  P02820  P02821  P02821  P02821  P02821  P02821  P02821  P05539  P05539  P05539  P05539  P05539  P05539  P05539  P05539  P05539  P05539  P05539  P05539  P05539  P05539  P05539  P05539  P05539  P05539  P05539  P05539  P05539  P05539  P05539  P05539  P05539  P05539  P05539  P05539  P05539  P05539  P05539  P05539  P05539  P05539  P05539  P05539  P05539  P05539  P05539  P05539  P05539  P05539  P05539  P05539  P05539  P05539  P05539  P05539  P05539  P05539  P05539  P05539  P05539  P05539  P05539  P05539  P05539  P05539  P05539  P05539  P05539  P05539  P05539  P05539  P05539  P05539  P05539  P05539  P05539  P05539  P05539  P05539  P05539  P05539  P05539  P05539  P05539  P05539  P05539  P05539  P05539  P05539  P05539  P05539  P05539  P05539  P05539  P05539  P05539  P05539  P05539  P05539  P05539  P05539  P05539  P05539  P05539  P05539  P05539  P05539  P05539  P05539  P05539  P05539  P05539  P05539  P05539  P05539  P05539  P05539  P05539  P05539  P05539  P05539  P05539  P05539  P05539  P05539  P05539  P05539  P05539  P05539  P05539  P05539  P05539  P05539  P05539  P05539  P05539  P05539  P05539  P05539  P05539  P05539  P05539  P05539  P05539  P05539  P05539  P05539  P05539  P05539  P05539  P05539  P05539  P05539  P05539  P05539  P05539  P05539  P05539  P05539  P05539  P05539  P05539  P05539  P05539  P05539  P05539  P05539  P05539  P05539  P05539  P05539  P05539  P05539  P05539  P05539  P05539  P05539  P05539  P05539  P05539  P05539  P05539  P05539  P05539  P05539  P05539  P05539  P05539  P05539  P05539  P05539  P05539  P05539  P05539  P05539  P05539  P05539  P05539  P05539  P05539  P05539  P05539  P05539  P05539  P05539  P05539  P05539  P05539  P05539  P05539  P05539  P05539  P05539  P05539  P05539  P05539  P05539  P05539  P05539  P05539  P05539  P05539  P05539  P05539  P05539  P05539  P05539  P05539  P05539  P05539  P05539  P05539  P05539  P05539  P05539  P05539  P05539  P05539  P05539  P05539  P05539  P05539  P05539  P05539  P05539  P05539  P05539  P05539  P05997  P05997  P05997  P05997  P05997  P05997  P05997  P05997  P05997  P05997  P05997  P05997  P05997  P05997  P05997  P05997  P05997  P05997  P05997  P05997  P05997  P05997  P05997  P05997  P05997  P05997  P05997  P05997  P05997  P05997  P05997  P05997  P05997  P05997  P05997  P05997  P05997  P05997  P05997  P05997  P05997  P05997  P05997  P05997  P05997  P05997  P05997  P05997  P05997  P05997  P05997  P05997  P05997  P05997  P05997  P05997  P05997  P05997  P05997  P05997  P05997  P05997  P05997  P05997  P05997  P05997  P05997  P05997  P05997  P05997  P05997  P05997  P05997  P05997  P05997  P05997  P05997  P05997  P05997  P05997  P05997  P05997  P05997  P05997  P05997  P05997  P05997  P05997  P05997  P05997  P05997  P05997  P05997  P05997  P05997  P05997  P05997  P05997  P05997  P05997  P05997  P05997  P05997  P05997  P05997  P05997  P05997  P05997  P05997  P05997  P05997  P05997  P05997  P05997  P05997  P05997  P05997  P05997  P05997  P05997  P05997  P05997  P05997  P05997  P05997  P05997  P05997  P05997  P05997  P05997  P05997  P05997  P05997  P05997  P05997  P05997  P05997  P05997  P05997  P05997  P05997  P05997  P05997  P05997  P05997  P05997  P05997  P05997  P05997  P05997  P05997  P05997  P05997  P05997  P05997  P05997  P05997  P05997  P05997  P05997  P05997  P05997  P05997  P05997  P05997  P05997  P05997  P05997  P05997  P05997  P05997  P05997  P05997  P05997  P05997  P05997  P05997  P05997  P05997  P05997  P05997  P05997  P05997  P05997  P05997  P05997  P05997  P05997  P05997  P05997  P05997  P05997  P05997  P05997  P05997  P05997  P05997  P05997  P05997  P05997  P05997  P05997  P05997  P05997  P05997  P05997  P05997  P05997  P05997  P05997  P05997  P05997  P05997  P05997  P05997  P05997  P05997  P05997  P05997  P05997  P05997  P05997  P05997  P05997  P05997  P05997  P05997  P05997  P05997  P05997  P05997  P05997  P05997  P05997  P05997  P05997  P05997  P05997  P05997  P05997  P05997  P05997  P05997  P05997  P05997  P05997  P05997  P05997  P05997  P05997  P05997  P05997  P05997  P05997  P05997  P05997  P05997  P05997  P05997  P05997  P05997  P05997  P05997  P07550  P07550  P07550  P07550  P07550  P07550  P07550  P07550  P07550  P07550  P08252  P08252  P08252  P08252  P08252  P08252  P08252  P08252  P08252  P08252  P08252  P08252  P08252  P08252  P08252  P08252  P08252  P08252  P08252  P08252  P08427  P08427  P08427  P08427  P08427  P08427  P08427  P08427  P08427  P08427  P08427  P08427  P08427  P08661  P08661  P08661  P08661  P08661  P08661  P08661  P08661  P08661  P08661  P0C1N1  P0C1N1  P0C1N1  P0C1N1  P0C1N1  P0C1N1  P0C1N5  P0C1N5  P0C1N5  P0C1N5  P0C1W5  P0C1W8  P0C1X1  P0C2W2  P0C2W2  P0C2W2  P0C2W2  P0C2W2  P0C2W2  P0C2W2  P0C2W2  P0C2W2  P0C2W2  P0C2W2  P0C2W2  P0C2W2  P0C2W2  P0C2W2  P0C2W2  P0C2W2  P0C2W2  P0C2W2  P0C2W2  P0C2W2  P0C2W2  P0C2W2  P0C2W2  P0C2W2  P0C2W2  P0C2W2  P0C2W2  P0C2W2  P0C2W2  P0C2W2  P0C2W2  P0C2W8  P0C2W8  P0C2W8  P0C2W8  P0C2W8  P0C2W8  P0C2W8  P0C2W8  P0C2W8  P0C2W8  P0C2W8  P0C2W8  P0C2W8  P0C2W8  P0C2W8  P0C2W8  P0C2W8  P0C2W8  P0C2W8  P0C2W8  P0C2W8  P0C2W8  P0C2W8  P0C2W8  P0C2W8  P0C2W8  P0C2W8  P0C2W8  P0C2W8  P0C2W8  P0C2W8  P0C2W8  P0C2W8  P0C2W8  P0C2W8  P0C2W8  P0C2W8  P0C2W8  P0C2W8  P0C2W8  P0C2W8  P0C2W8  P0C2W8  P0C2W8  P0C2W8  P0C2W8  P0C2W8  P0C2W8  P0C2W8  P0C2W8  P0C2W8  P0C2W8  P0C2W8  P0C2W8  P0C2W8  P0C2W8  P0C2W8  P0C2W8  P0C2W8  P0C2W8  P0C2W8  P0C2W8  P0C2W8  P0C2W8  P0C2W8  P0C2W8  P0C2W8  P0C2W8  P0C2W8  P0C2W8  P0C2W8  P0C2W8  P0C2W8  P0C2W8  P0C2W8  P0C2W8  P0C2W8  P0C2W8  P0C2W8  P0C2W8  P0C2W8  P0C2W8  P0C2W8  P0C2W8  P0C2W8  P0C2W8  P0C2W8  P0C2W8  P0C2W8  P0C2W8  P0C2W8  P0C2W8  P0C351  P0C424  P0C7W7  P0C8S5  P0C8S5  P0C8V5  P0C8V5  P0C8V6  P0C8V6  P0C8V6  P0C8V9  P0C8W0  P0C8W0  P0C8W0  P0C8W0  P0C8W0  P0CE29  P0CE29  P0CE30  P12108  P12108  P12108  P12108  P12108  P12108  P12108  P12108  P12108  P12108  P12108  P12108  P12108  P12108  P12108  P12108  P12108  P12108  P12108  P12108  P12108  P12108  P12108  P12108  P12108  P12108  P12108  P12108  P12108  P12108  P12108  P12108  P12108  P12108  P12108  P12108  P12108  P12108  P12108  P12108  P12108  P12108  P12108  P12108  P12108  P12108  P12108  P12108  P12108  P12108  P12108  P12108  P12108  P12108  P12108  P12108  P12108  P12108  P12108  P12108  P12108  P12108  P12108  P12108  P12108  P12108  P12108  P12108  P12108  P12108  P12108  P12108  P12108  P12108  P12108  P12108  P12108  P12108  P12108  P12108  P12108  P12108  P12108  P12108  P12108  P12108  P12108  P12108  P12108  P12108  P12108  P12108  P12108  P12108  P12108  P12108  P12108  P12108  P12108  P12108  P12108  P12108  P12108  P12108  P12108  P12108  P12108  P12108  P12108  P12108  P12108  P12108  P12108  P12108  P12108  P12108  P12108  P12108  P12108  P12108  P12108  P12108  P12108  P12108  P12108  P12108  P12108  P12108  P12108  P12111  P12111  P12111  P12111  P12111  P12111  P12111  P12111  P12111  P12111  P12111  P12111  P12111  P12111  P12111  P12111  P12111  P12111  P12111  P12111  P12111  P12111  P12111  P12111  P12111  P12111  P12111  P12111  P12111  P12111  P12111  P12111  P12111  P12111  P12111  P12111  P12111  P12111  P12111  P12111  P12111  P12111  P12111  P12111  P12111  P12111  P12111  P12111  P12111  P12111  P12111  P12111  P12111  P12111  P12111  P12111  P12111  P12111  P12111  P12111  P12111  P12111  P12111  P12111  P12111  P12111  P12111  P12111  P12111  P12111  P12111  P12111  P12111  P12111  P12111  P12111  P12111  P12111  P12111  P12111  P12111  P12111  P12111  P12111  P12111  P12111  P12111  P12111  P12111  P12111  P12111  P12111  P12111  P12111  P12111  P12111  P12111  P12111  P12111  P12111  P12111  P12111  P12111  P12111  P12111  P12111  P12111  P12111  P12111  P12111  P12111  P12111  P12111  P12111  P12111  P12111  P12111  P12111  P12111  P12111  P12111  P12111  P12111  P12111  P12111  P12111  P12111  P12111  P12111  P12111  P12111  P12111  P12111  P12111  P12111  P12111  P12111  P12111  P12111  P12111  P12111  P12111  P12111  P12111  P12111  P12111  P12111  P12111  P12111  P12111  P12111  P12111  P12111  P12111  P12111  P12111  P12111  P12111  P12111  P12111  P12111  P12111  P12111  P12111  P12111  P12111  P12111  P12111  P12111  P12111  P12111  P12111  P12111  P12111  P12111  P12111  P12111  P12111  P12111  P12111  P12111  P12111  P12111  P12111  P12111  P12111  P12111  P12111  P12111  P12111  P12111  P12111  P12111  P12111  P12111  P12111  P12111  P12111  P12111  P12111  P12111  P12111  P12111  P12111  P14618  P14618  P14618  P14618  P14618  P14618  P14618  P14618  P14618  P14618  P14618  P14618  P14618  P14618  P14618  P14618  P14618  P14618  P14618  P14618  P14618  P14618  P15472  P15502  P15502  P15502  P15502  P15502  P15502  P15502  P15502  P15502  P15502  P15502  P15502  P15502  P15502  P15502  P15502  P15502  P15502  P15502  P15502  P15502  P15502  P15502  P15502  P15502  P15502  P15502  P15502  P15502  P15502  P15502  P15502  P15502  P15502  P15502  P15502  P15502  P15502  P15502  P15502  P15502  P15502  P15502  P15502  P15502  P15502  P15502  P15502  P15502  P15502  P15502  P15502  P15502  P15502  P15502  P15502  P15502  P15502  P15502  P15502  P15502  P15502  P15502  P15502  P15502  P19999  P19999  P19999  P19999  P19999  P19999  P19999  P19999  P23805  P23805  P23805  P23805  P23805  P23805  P23805  P23805  P23805  P23805  P23805  P23805  P23805  P23805  P23805  P23805  P23805  P23805  P23805  P23805  P23805  P23805  P23805  P23805  P23805  P23805  P23805  P23805  P23805  P23805  P23805  P23805  P24091  P24091  P24091  P24091  P29602  P29602  P29602  P29602  P29602  P30754  P30754  P30754  P30754  P30754  P30754  P30754  P30754  P30754  P30754  P30754  P30754  P30754  P30754  P30754  P30754  P30754  P30754  P30754  P30754  P30754  P30754  P30754  P30754  P30754  P30754  P30754  P30754  P30754  P30754  P30754  P30754  P30754  P30754  P30754  P30754  P30754  P30754  P30754  P30754  P30754  P30754  P30754  P30754  P30754  P30754  P30754  P30754  P30754  P30754  P30754  P30754  P30754  P30754  P30754  P30754  P30754  P30754  P30754  P39056  P39056  P39056  P39056  P39056  P50982  P50982  P52285  P52285  P52285  P52285  P52285  P52285  P52285  P52285  P52285  P52285  P56529  P56529  P56529  P56711  P58782  P58786  P58786  P58786  P58786  P58787  P58787  P58787  P58787  P58804  P58808  P58808  P58808  P58808  P58841  P58843  P58846  P58846  P58846  P58846  P58846  P58913  P58914  P58924  P58925  P58925  P58925  P58925  P58928  P60245  P60274  P60513  P69747  P69765  P69765  P69765  P80760  P80760  P80760  P80760  P80760  P80762  P80762  P80774  P80774  P80774  P81755  P81755  P81755  P84349  P84349  P84349  P84349  P84349  P84349  P84349  P84895  P84895  P84900  P84900  P84900  P84935  P84935  P84946  P85017  P85017  P85022  P85065  P85153  P85153  P85153  P85153  P85153  P85153  P85153  P85153  P85153  P85153  P85153  P85153  P85153  P85153  P85153  P85153  P85153  P85153  P85153  P85153  P85153  P85153  P85153  P85153  P85153  P85153  P85153  P85153  P85153  P85153  P85153  P85153  P85153  P85153  P85153  P85153  P85153  P85153  P85153  P85153  P85153  P85153  P85153  P85154  P85154  P85154  P85154  P85154  P85154  P85154  P85154  P85154  P85154  P85154  P85154  P85154  P85154  P85154  P85154  P85154  P85154  P85154  P85154  P85154  P85154  P85154  P85154  P85154  P85154  P85154  P85154  P85154  P85154  P85154  P85154  P85154  P85154  P85154  P85154  P85154  P85154  P85154  P85154  P85154  P85154  P85154  P85154  P85154  P85154  P85154  P85154  P85154  P85154  P85154  P85154  P85154  P85154  P85154  P85154  P85154  P85154  P85154  P85154  P85154  P85154  P85154  P85154  P85154  P85154  P85154  P85154  P85154  P85154  P85154  P85154  P85154  P85154  P85154  P85154  P85154  P85154  P85154  P85154  P85154  P85154  P85154  P85154  P85154  P85154  P85154  P85154  P85154  P85154  P85154  P85154  P85154  P85154  P85154  P85154  P85154  P85154  P85154  P85154  P85154  P85154  P85154  P85154  P85154  P85154  P85154  P85154  P85154  P85154  P85154  P85154  P85154  P85154  P85154  P85154  P85154  P85154  P85154  P85154  P85154  P85154  P85154  P85154  P85154  P85154  P85154  P85154  P85154  P85154  P85154  P85154  P85154  P85154  P85154  P85154  P85154  P85154  P85154  P85154  P85154  P85154  P85154  P85154  P85154  P85154  P85154  P85154  P85154  P85154  P85154  P85154  P85154  P85154  P85154  P85154  P85154  P85154  P85154  P85154  P85154  P85154  P85154  P85154  P85154  P85154  P85154  P85154  P85154  P85154  P85154  P85154  P85154  P85154  P85154  P85154  P85154  P85154  P85154  P85886  P86256  P86289  P86289  P86289  P86289  P86289  P86289  P86289  P86289  P86289  P86289  P86289  P86289  P86289  P86289  P86289  P86290  P86290  P86290  P86290  P86290  P86500  P86500  P86500  Q02388  Q02388  Q02388  Q02388  Q02388  Q02388  Q02388  Q02388  Q02388  Q02388  Q02388  Q02388  Q02388  Q02388  Q02388  Q02388  Q02388  Q02388  Q02388  Q02388  Q02388  Q02388  Q02388  Q02388  Q02388  Q02388  Q02388  Q02388  Q02388  Q02388  Q02388  Q02388  Q02388  Q02388  Q02388  Q02388  Q02388  Q02388  Q02388  Q02388  Q02388  Q02388  Q02388  Q02388  Q02388  Q02388  Q02388  Q02388  Q02388  Q02388  Q02388  Q02388  Q02388  Q02388  Q02388  Q02388  Q02388  Q02388  Q02388  Q02388  Q02388  Q02388  Q02388  Q02388  Q02388  Q02388  Q02388  Q02388  Q02388  Q02388  Q02388  Q02388  Q02388  Q02388  Q02388  Q02388  Q02388  Q02388  Q02388  Q02388  Q02388  Q02388  Q02388  Q02388  Q02388  Q02388  Q02388  Q02388  Q02388  Q02388  Q02388  Q02388  Q02388  Q02388  Q02388  Q02388  Q02388  Q02388  Q02388  Q02388  Q02388  Q02388  Q02388  Q02388  Q02388  Q02388  Q02388  Q02388  Q02388  Q02388  Q02388  Q02388  Q02388  Q02388  Q02388  Q02388  Q02388  Q02388  Q02388  Q02388  Q02388  Q02388  Q02388  Q02388  Q02388  Q02388  Q02388  Q02388  Q02388  Q02388  Q02388  Q02388  Q02388  Q02388  Q02388  Q02388  Q02388  Q02388  Q02388  Q02388  Q02388  Q02388  Q02388  Q02388  Q02388  Q02388  Q02388  Q02388  Q02388  Q02388  Q02388  Q02388  Q02388  Q02388  Q02388  Q02388  Q02388  Q02388  Q02388  Q02388  Q02388  Q02388  Q02388  Q02388  Q02388  Q02388  Q02388  Q02388  Q02388  Q02388  Q02388  Q02388  Q02388  Q02388  Q02388  Q02388  Q02388  Q02388  Q02388  Q02388  Q02388  Q02388  Q02388  Q02388  Q02388  Q02388  Q02388  Q02388  Q02388  Q02388  Q02388  Q02388  Q02388  Q02388  Q02388  Q02388  Q02388  Q02388  Q02388  Q02388  Q02388  Q02388  Q02388  Q02388  Q02388  Q02388  Q02388  Q02388  Q02388  Q02388  Q02388  Q02388  Q02388  Q02388  Q02388  Q02388  Q02388  Q02388  Q02388  Q02388  Q02388  Q02388  Q02388  Q02388  Q02388  Q02388  Q02388  Q02388  Q02388  Q02388  Q02388  Q02388  Q02388  Q02388  Q02388  Q02388  Q02388  Q02388  Q02388  Q02388  Q02388  Q02388  Q02388  Q02388  Q02388  Q02388  Q02388  Q02388  Q02388  Q02388  Q02388  Q02388  Q02388  Q02388  Q02388  Q02388  Q02388  Q02388  Q02388  Q02388  Q02388  Q02388  Q02388  Q02388  Q02388  Q02388  Q02388  Q02388  Q02388  Q02388  Q02388  Q02388  Q02388  Q02388  Q02388  Q02388  Q02388  Q02388  Q02388  Q02388  Q02388  Q02388  Q02388  Q02388  Q02388  Q02388  Q02388  Q02388  Q02388  Q02388  Q02388  Q02388  Q02388  Q02388  Q02388  Q02388  Q02388  Q02388  Q02388  Q02388  Q02388  Q02388  Q02388  Q02388  Q02388  Q02388  Q02388  Q02388  Q02388  Q02388  Q02388  Q02388  Q02388  Q02388  Q02388  Q02388  Q02388  Q02388  Q02388  Q02388  Q02388  Q02388  Q02388  Q02388  Q02388  Q02388  Q02388  Q02388  Q02388  Q02388  Q02388  Q02388  Q02388  Q02388  Q02388  Q02388  Q02388  Q02388  Q02388  Q02388  Q02388  Q02388  Q02388  Q02388  Q02388  Q02388  Q02388  Q02388  Q02388  Q02388  Q02388  Q02388  Q02388  Q02388  Q02388  Q02388  Q02388  Q02388  Q02388  Q02388  Q02388  Q02388  Q02388  Q02388  Q02388  Q02388  Q02388  Q02388  Q02388  Q02388  Q02388  Q02388  Q02388  Q02388  Q02388  Q02388  Q02388  Q02388  Q02388  Q02388  Q02388  Q02388  Q02388  Q02388  Q02388  Q02388  Q02388  Q02388  Q02388  Q02388  Q02388  Q02388  Q02388  Q02388  Q02388  Q02388  Q02388  Q02388  Q02388  Q02388  Q02388  Q02388  Q02388  Q02388  Q02388  Q02388  Q02388  Q02388  Q02388  Q02388  Q02388  Q02388  Q02388  Q02388  Q02388  Q02388  Q02388  Q02388  Q02388  Q05707  Q05707  Q05707  Q05707  Q05707  Q05707  Q05707  Q05707  Q05707  Q05707  Q05707  Q05707  Q05707  Q05707  Q05707  Q05707  Q05707  Q05707  Q05707  Q05707  Q05707  Q05707  Q05707  Q05707  Q05707  Q05707  Q05707  Q05707  Q05707  Q05707  Q05707  Q05707  Q05707  Q05707  Q05707  Q05707  Q05707  Q05707  Q05707  Q05707  Q05707  Q05707  Q05707  Q05707  Q05707  Q05707  Q05707  Q05707  Q05707  Q05707  Q05707  Q05707  Q05707  Q05707  Q05707  Q05707  Q05707  Q05707  Q05707  Q05707  Q05707  Q05707  Q05707  Q05707  Q05707  Q05707  Q05707  Q05707  Q05707  Q05707  Q05707  Q05707  Q05707  Q05707  Q05707  Q05707  Q05707  Q05707  Q05707  Q05707  Q05707  Q05707  Q05707  Q05707  Q05707  Q05707  Q05707  Q05707  Q05707  Q05707  Q05707  Q05707  Q05707  Q05707  Q05707  Q05707  Q05707  Q05707  Q05707  Q05707  Q05707  Q05707  Q05707  Q05707  Q05707  Q05707  Q05707  Q05707  Q05707  Q05707  Q05707  Q05707  Q05707  Q05707  Q05707  Q05707  Q05707  Q0VKG8  Q0VKG8  Q0VTT8  Q0VTT8  Q0VTT8  Q15848  Q15848  Q15848  Q15848  Q15848  Q15848  Q15848  Q15848  Q15848  Q15848  Q16665  Q16665  Q16665  Q16665  Q16665  Q16665  Q16665  Q16665  Q16665  Q16665  Q16665  Q16665  Q16665  Q16665  Q16665  Q16665  Q16665  Q16665  Q16665  Q16665  Q16665  Q16665  Q16665  Q16665  Q16665  Q16665  Q16665  Q16665  Q16665  Q16665  Q16665  Q16665  Q16665  Q16665  Q16665  Q16665  Q16665  Q16665  Q16665  Q16665  Q16665  Q16665  Q16665  Q16665  Q16665  Q16665  Q16665  Q16665  Q24940  Q24940  Q24940  Q24940  Q24940  Q24940  Q25460  Q25460  Q25460  Q25460  Q25460  Q25460  Q25460  Q25460  Q25460  Q25460  Q25460  Q25460  Q25460  Q25460  Q25460  Q25460  Q25460  Q25460  Q25460  Q25460  Q25460  Q25460  Q25460  Q25460  Q25460  Q25460  Q25460  Q25460  Q25460  Q25460  Q25460  Q25460  Q25460  Q25460  Q25460  Q25460  Q25460  Q25460  Q25460  Q25460  Q25460  Q25460  Q25460  Q25460  Q25460  Q25460  Q25460  Q25460  Q25460  Q25460  Q25460  Q25460  Q25460  Q25460  Q25460  Q25460  Q25460  Q25460  Q25460  Q25460  Q25460  Q25460  Q25460  Q25460  Q25460  Q25460  Q25460  Q25460  Q25460  Q25460  Q25460  Q25460  Q25460  Q25460  Q25460  Q25460  Q25460  Q25460  Q25460  Q25460  Q25460  Q25460  Q25460  Q25460  Q25460  Q25460  Q25460  Q25460  Q25460  Q25460  Q25460  Q25460  Q25460  Q2I2Q5  Q2I2Q5  Q2I2Q5  Q2I2Q5  Q3Y5Z3  Q3Y5Z3  Q3Y5Z3  Q3Y5Z3  Q3Y5Z3  Q3Y5Z3  Q3Y5Z3  Q3Y5Z3  Q3Y5Z3  Q3Y5Z3  Q3Y5Z3  Q3Y5Z3  Q3Y5Z3  Q4ZJN1  Q4ZJN1  Q4ZJN1  Q4ZJN1  Q4ZJN1  Q4ZJN1  Q4ZJN1  Q4ZJN1  Q4ZJN1  Q4ZJN1  Q4ZJN1  Q4ZJN1  Q4ZJN1  Q4ZJN1  Q4ZJN1  Q4ZJN1  Q4ZJN1  Q4ZJN1  Q4ZJN1  Q4ZJN1  Q60994  Q60994  Q60994  Q60994  Q60994  Q60994  Q60994  Q60994  Q60994  Q60994  Q60994  Q60994  Q60994  Q6PSU2  Q6PSU2  Q6PSU2  Q6PSU2  Q6PSU2  Q6PSU2  Q6PSU2  Q6PSU2  Q7XAD0  Q7XAD0  Q7XAD0  Q7XAD0  Q7XAD0  Q7XAD0  Q7XAD0  Q7Z091  Q7Z092  Q7Z094  Q800F1  Q800F1  Q800F1  Q8LG54  Q8LG54  Q8LG54  Q8LG54  Q8LG54  Q8LG54  Q8LG54  Q8LG54  Q8LG54  Q8LG54  Q8LG54  Q8LG54  Q8LG54  Q8LG54  Q8LG54  Q8LG54  Q8LG54  Q8LG54  Q8LG54  Q8LG54  Q8LG54  Q8LG54  Q8LG54  Q8LG54  Q8LG54  Q8LG54  Q93WP7  Q93WP7  Q93WP7  Q93WP7  Q93WP7  Q93WP7  Q93WP7  Q93WP7  Q93WP8  Q93WP8  Q93WP8  Q93WP8  Q93WP8  Q93WP8  Q93WP8  Q941C7  Q941C7  Q941C7  Q941C7  Q9BPB1  Q9BPB2  Q9BPG6  Q9BPG6  Q9BPJ7  Q9BPJ7  Q9BPJ7  Q9BPJ7  Q9BPJ7  Q9C5S0  Q9C5S0  Q9C5S0  Q9C5S0  Q9C5S0  Q9C5S0  Q9C5S0  Q9C5S0  Q9C5S0  Q9C5S0  Q9C5S0  Q9C5S0  Q9C5S0  Q9C5S0  Q9C5S0  Q9C5S0  Q9C5S0  Q9C5S0  Q9C5S0  Q9C5S0  Q9C5S0  Q9C5S0  Q9C5S0  Q9C5S0  Q9C5S0  Q9C5S0  Q9C5S0  Q9C5S0  Q9C5S0  Q9C5S0  Q9C5S0  Q9C5S0  Q9C5S0  Q9C5S0  Q9C5S0  Q9C5S0  Q9C5S0  Q9C5S0  Q9C5S0  Q9C5S0  Q9C5S0  Q9C5S0  Q9C5S0  Q9C5S0  Q9C5S0  Q9C5S0  Q9C5S0  Q9C5S0  Q9C5S0  Q9C5S0  Q9C5S0  Q9C5S0  Q9C5S0  Q9C5S0  Q9C5S0  Q9C5S0  Q9C5S0  Q9C5S0  Q9C5S0  Q9C5S0  Q9C5S0  Q9LJD9  Q9LVC0  Q9M0S4  Q9M0S4  Q9M0S4  Q9M0S4  Q9M0S4  Q9M0S4  Q9M0S4  Q9M0S4  Q9M0S4  Q9M0S4  Q9M0S4  Q9M0S4  Q9M0S4  Q9M0S4  Q9M0S4  Q9M0S4  Q9M0S4  Q9M0S4  Q9M0S4  Q9M0S4  Q9M0S4  Q9M0S4  Q9M0S4  Q9SJY7  Q9SJY7  Q9SJY7  Q9SJY7  Q9SJY7  Q9SJY7  Q9SJY7  Q9SJY7  Q9SJY7  Q9SJY7  Q9SJY7  Q9SJY7  Q9SJY7  Q9SJY7  Q9SJY7  Q9SJY7  Q9SJY7  Q9SJY7  Q9SJY7  Q9SJY7  Q9SJY7  Q9SJY7  Q9SJY7  Q9STQ3  Q9U3Z3  Q9U3Z3  Q9UKV8  Q9UKV8  Q9UKV8  Q9UKV8  Q9UKV8  Q9UKV8  Q9UKV8  Q9UKV8  Q9UKV8  Q9UKV8  Q9UKV8  Q9UKV8  Q9UKV8  Q9UKV8  Q9UKV8  Q9UKV8  Q9UKV8  Q9UKV8  Q9UKV8  Q9UKV8  Q9UKV8  Q9UKV8  Q9UKV8  Q9UKV8  Q9UKV8  Q9UKV8  Q9UKV8  Q9UKV8  Q9UKV8  Q9UKV8  Q9UKV8  Q9UKV8  Q9UKV8  Q9UKV8  Q9UKV8  Q9UKV8  Q9UKV8  Q9UKV8  Q9UKV8  Q9UKV8  Q9UKV8  Q9UKV8  Q9UKV8  Q9UKV8  Q9UKV8  Q9UKV8  Q9UKV8  Q9UKV8  Q9UKV8  Q9UKV8  Q9UKV8  Q9UKV8  Q9UKV8  Q9UKV8  Q9UKV8  Q9UKV8  Q9UKV8  Q9Y2N7  Q9Y2N7  Q9Y2N7  Q9Y2N7  Q9Y2N7  Q9Y2N7  Q9Y2N7  Q9Y2N7  Q9Y2N7  Q9Y2N7  Q9Y2N7  Q9Y2N7  Q9Y2N7  Q9Y2N7  Q9Y2N7  Q9Y2N7  Q9Y2N7  Q9Y2N7  Q9Y2N7  Q9Y2N7  Q9Y2N7  Q9Y2N7  Q9Y2N7  Q9Y2N7  Q9Y2N7  Q9Y2N7  Q9Y2N7  Q9Y2N7  Q9Y2N7  Q9Y2N7  Q9Y2N7  Q9Y2N7  Q9Y2N7  Q9Y2N7  Q9Y2N7  Q9Y2N7  Q9Y2N7  Q9Y2N7  Q9Y2N7  Q9Y2N7  Q9Y2N7  Q9Y2N7  Q9Y2N7  Q9Y2N7  Q9Y2N7  Q9Y2N7  Q9Y2N7  Q9Y2N7  Q9Y2N7  Q9Y2N7  Q9Y2N7  Q9Y2N7  Q9Y2N7  Q9Y2N7  Q9Y2N7  Q9Y2N7  Q9Y2N7  Q9Y2N7  Q9Y2N7  Q9Y2N7  Q9Y2N7  Q9ZT16  Q9ZT16  Q9ZT16  Q9ZT16  Q9ZT16  Q9ZT16  Q9ZT16  Q9ZT16  Q9ZT16  Q9ZT16  Q9ZT16  Q9ZT16  Q9ZT16  Q9ZT16  Q9ZT16  Q9ZT16  Q9ZT16  Q9ZT16  Q9ZT16  Q9ZT16  Q9ZT16  Q9ZT16  Q9ZT16  Q9ZT16  Q9ZT16  Q9ZT16  Q9ZT16 | 24  25  33  35  44  54  63  74  94  103  114  133  143  173  183  193  204  213  253  313  343  354  413  433  443  444  445  461  464  467  472  475  478  479  496  497  500  503  506  557  558  15  34  27  17  62  16  69  2  15  93  2  15  70  74  23  42  46  60  69  28  48  62  63  5  6  35  76  81  88  93  3  27  44  49  50  89  91  141  142  152  176  210  263  372  376  388  410  415  453  84  130  134  148  154  158  216  252  259  260  268  272  276  359  366  372  382  387  404  405  411  534  545  547  549  554  574  575  578  580  589  595  602  607  612  615  618  627  16  21  27  31  45  36  37  55  57  82  87  93  96  102  113  119  122  125  128  129  135  138  141  143  144  146  147  149  150  152  153  161  177  179  182  185  189  191  192  195  197  203  204  207  210  215  218  221  222  224  225  237  240  245  246  248  255  261  281  284  288  291  297  302  306  312  315  317  332  333  335  338  341  342  345  356  359  365  372  374  375  377  383  387  393  402  408  411  416  419  422  425  426  428  435  438  450  452  458  459  461  474  476  480  482  483  489  495  503  506  513  515  518  522  528  534  543  546  548  554  555  557  564  566  567  569  570  582  584  591  600  602  603  608  620  621  623  626  635  639  645  647  650  651  657  663  669  671  681  689  690  692  695  702  714  717  723  729  738  740  749  756  767  770  771  773  777  785  788  791  798  804  806  807  809  815  816  822  828  839  840  842  845  848  849  851  858  860  869  870  876  884  885  887  893  894  896  897  899  908  914  918  923  924  926  927  929  936  941  945  948  950  963  972  975  977  981  986  995  996  998  1001  1002  1007  1008  1017  1023  1029  1040  1043  1044  1047  1050  1053  1055  1058  1070  1073  1076  1079  1085  1088  1091  1118  1119  1121  1122  1125  1130  1136  1139  1142  1143  1149  1158  1160  1164  1166  1175  1178  1179  1181  1182  1184  1185  1187  1188  1190  1191  1201  1203  1204  1247  1254  1277  1301  1304  1317  1344  1437  1443  1449  1459  48  50  75  80  86  89  95  98  106  112  119  121  122  128  136  137  139  140  142  143  151  167  169  172  175  181  205  208  211  214  227  235  238  271  274  304  307  322  325  328  331  346  355  364  367  406  409  412  418  442  448  451  466  472  493  496  508  547  556  559  574  592  610  613  616  625  637  661  679  682  685  712  739  757  760  775  778  781  829  832  835  841  847  849  873  874  876  883  885  886  888  896  902  906  909  914  915  918  924  929  933  936  938  989  990  995  996  1005  1011  1019  1028  1041  1043  1046  1058  1061  1064  1065  1073  1076  1106  1107  1110  1113  1118  1127  1130  1131  1146  1151  1152  1163  1166  1167  1170  1173  1176  1179  1189  1191  1192  1235  1242  1289  1305  1332  1425  1431  1437  1447  35  41  47  48  50  51  59  60  62  63  65  66  68  69  81  89  91  97  100  101  106  107  109  110  116  119  122  127  130  133  134  136  137  146  149  152  160  167  170  172  173  191  194  200  203  209  212  218  227  229  241  244  250  256  259  262  269  274  277  278  281  287  296  302  305  307  311  313  314  316  319  329  331  344  349  350  352  353  355  368  373  374  376  383  389  400  409  415  422  425  430  434  445  452  458  460  463  467  470  481  485  487  496  497  511  514  515  521  523  526  533  545  547  550  557  565  568  574  577  578  580  587  592  596  598  601  605  614  623  641  647  649  661  664  667  674  680  682  685  691  692  698  706  709  715  721  727  728  730  736  739  745  746  752  760  761  763  769  770  772  773  775  781  790  802  803  811  820  821  824  826  833  839  848  857  859  865  866  871  874  878  880  884  893  898  899  905  914  917  919  926  928  934  938  944  946  949  952  958  964  967  974  983  995  1006  1007  1012  1015  1018  1019  1021  1024  1025  1034  1036  1039  1051  1054  1055  1057  1058  1060  1061  1063  1064  1066  1085  1089  1111  1118  1130  1141  1171  1181  1297  1301  1307  1323  4  29  75  87  90  97  106  113  115  124  125  143  156  221  243  5  9  23  56  60  66  71  78  84  89  92  101  108  131  132  134  147  163  244  4  25  62  64  66  69  78  99  4  11  13  15  18  27  7  33  40  49  52  53  55  58  64  77  79  86  89  92  94  95  97  98  100  101  103  104  134  137  140  143  144  146  150  152  159  162  165  170  173  176  177  179  183  192  201  203  210  213  216  225  237  246  252  254  257  261  269  282  284  287  288  290  293  297  303  311  317  327  330  333  335  342  348  357  363  366  368  371  372  374  380  383  390  401  407  410  414  416  429  434  437  438  444  450  458  462  467  473  480  483  486  489  498  503  509  513  519  521  522  524  531  537  539  546  558  564  575  576  578  581  591  593  600  603  605  606  618  624  629  636  642  650  654  657  662  668  671  672  677  678  684  695  707  711  722  725  726  728  740  741  743  753  761  762  764  777  795  797  800  804  806  809  815  824  825  831  840  846  848  851  852  854  860  870  876  881  885  891  896  902  903  905  918  932  936  942  950  951  953  956  957  962  966  972  977  978  999  1002  1004  1005  1008  1010  1013  1028  1031  1034  1043  1046  1056  1074  1077  1079  1088  1091  1094  1097  1098  1100  1103  1113  1115  1119  1121  1130  1134  1137  1140  1142  1143  1146  1148  1165  1167  1204  1211  1223  1234  1258  1260  1264  1300  1393  1399  1405  1415  10  56  58  85  88  89  94  100  118  122  131  133  136  137  142  148  152  154  157  164  167  170  173  175  176  179  182  184  190  212  217  220  223  235  238  239  242  245  247  250  256  259  260  263  269  275  284  310  317  325  328  334  337  341  349  353  362  365  367  370  374  380  383  391  397  400  409  410  412  416  419  428  433  437  442  443  448  449  452  454  460  467  470  473  481  485  487  493  496  497  505  509  514  515  517  524  530  536  538  547  553  560  563  566  569  578  583  589  593  599  601  602  619  626  629  638  644  652  655  658  659  670  671  673  680  682  683  685  686  692  698  701  706  709  716  722  728  739  742  746  748  752  757  758  764  773  775  800  802  805  806  808  811  818  820  826  827  833  841  850  853  857  863  866  875  877  884  886  889  893  904  905  911  920  922  926  928  931  935  940  941  947  958  961  962  965  971  977  979  982  983  985  998  1007  1010  1012  1016  1021  1030  1031  1033  1036  1037  1042  1046  1048  1051  1058  1073  1075  1079  1085  1088  1090  1094  1103  1108  1111  1112  1121  1123  1126  1154  1157  1159  1169  1171  1174  1177  1178  1180  1183  1190  1192  1195  1198  1199  1210  1213  1214  1217  1219  1220  1222  1223  1225  1226  1245  1247  1249  1258  1266  1285  1292  1315  1341  1345  1355  1359  1380  1473  1479  1495  4  14  20  88  138  168  211  288  323  330  36  56  64  120  154  157  171  177  182  185  194  205  218  225  239  242  244  256  267  308  56  59  62  79  82  91  97  100  165  168  195  213  216  30  37  68  74  77  89  143  161  209  231  16  21  27  31  45  49  21  45  57  60  15  10  23  21  23  26  32  62  110  129  199  219  235  245  249  250  281  286  293  296  299  330  337  345  415  416  430  441  442  468  473  509  523  546  548  17  19  22  196  205  256  259  262  275  278  298  301  316  322  343  346  353  355  358  440  442  443  529  532  535  542  589  596  601  607  610  680  682  685  689  691  698  709  736  737  739  748  754  766  769  776  781  785  788  790  803  821  826  835  836  838  841  842  847  848  871  880  883  884  910  913  916  919  925  928  931  979  982  983  989  1000  1003  1006  1015  1018  1019  1021  1022  1024  1025  1027  1028  1030  1031  1041  1043  1044  9  4  5  9  23  34  43  34  56  75  76  9  15  19  29  73  15  40  71  6  26  27  30  32  35  36  38  39  42  54  57  60  66  69  71  74  75  78  92  102  105  107  108  111  113  116  118  119  122  128  134  136  140  142  145  148  149  151  152  155  157  173  177  180  183  196  202  210  223  225  226  228  235  240  246  262  267  270  271  274  288  294  301  310  313  322  325  327  328  337  340  346  364  370  372  373  382  393  397  405  411  412  415  417  421  430  435  442  460  462  475  477  484  490  492  493  495  505  508  555  556  558  559  561  562  564  565  573  576  580  594  612  615  617  618  621  624  627  640  641  644  650  653  655  659  664  672  674  8  21  87  143  161  192  223  273  290  347  357  403  417  431  432  495  503  517  550  568  605  612  618  623  631  656  687  744  793  806  809  815  828  832  853  870  897  959  970  980  990  1000  1007  1021  1023  1045  1078  1104  1106  1133  1151  1162  1182  1189  1218  1222  1224  1226  1246  1280  1304  1327  1337  1355  1366  1385  1398  1407  1426  1427  1428  1449  1470  1486  1498  1513  1541  1590  1603  1615  1620  1622  1623  1628  1629  1630  1633  1687  1733  1744  1826  1883  1894  1897  1908  1922  1942  2005  2033  2045  2051  2055  2064  2070  2075  2076  2088  2124  2133  2141  2157  2160  2169  2178  2182  2188  2191  2205  2215  2218  2232  2235  2238  2250  2260  2268  2275  2278  2280  2289  2321  2325  2328  2336  2348  2349  2358  2361  2363  2366  2388  2393  2397  2400  2440  2519  2524  2540  2569  2595  2604  2653  2655  2671  2681  2682  2731  2733  2748  2790  2808  2817  2830  2844  2859  2866  2870  2876  2882  2889  2896  2903  2907  2912  2914  2917  2922  2932  2933  2938  2944  2949  2954  2955  2962  2967  2970  2972  2977  2982  2987  3014  3015  3017  3081  3082  3083  3084  3086  3103  3115  3132  3164  3169  4  38  39  53  102  107  117  212  219  302  323  338  340  371  446  449  457  471  477  517  529  531  24  9  11  23  26  30  38  44  58  62  83  93  130  132  138  142  147  151  161  180  194  198  200  203  207  211  214  220  227  232  245  252  275  301  324  331  337  365  370  387  395  405  433  439  472  475  486  504  510  516  528  534  540  546  623  630  637  640  643  662  685  720  729  730  755  763  5  50  60  66  137  155  203  237  5  15  42  48  59  66  69  71  83  86  98  104  107  113  119  128  134  140  155  165  179  185  188  194  204  249  285  321  336  344  352  360  64  70  251  262  16  19  42  74  132  5  14  17  20  23  44  47  56  74  80  83  95  119  125  131  134  167  200  239  245  266  284  308  314  344  353  401  404  452  458  464  467  521  554  566  581  605  629  635  653  680  719  725  728  779  803  818  830  833  836  893  911  920  962  968  983  992  998  1001  11  13  15  27  48  51  58  40  42  44  65  67  82  83  94  110  128  16  27  31  38  5  28  35  40  60  28  35  40  60  32  5  18  24  31  14  14  21  27  31  45  49  75  3  77  16  21  27  45  6  12  9  13  62  10  23  46  5  10  15  20  25  5  14  4  5  14  5  18  31  25  49  62  64  66  69  78  2  7  50  53  58  3  7  2  6  16  6  9  8  11  18  24  46  48  49  66  86  88  102  122  222  231  258  279  294  311  327  351  355  361  371  380  393  422  425  428  434  440  446  447  511  512  521  544  569  594  628  651  662  665  668  11  13  19  22  23  28  29  32  37  38  41  44  49  55  58  59  71  74  82  89  92  95  113  116  122  125  131  140  149  151  163  166  169  175  179  184  190  194  196  199  202  209  214  217  218  221  236  245  247  251  253  269  271  289  292  295  304  313  316  317  323  329  340  341  349  355  362  365  370  374  377  382  388  392  398  400  403  416  418  421  425  439  443  445  454  455  469  472  481  505  508  514  515  523  526  532  538  545  551  556  559  563  572  607  619  625  628  632  640  643  649  667  673  676  679  685  688  691  692  694  703  718  719  721  727  730  742  748  760  773  778  779  782  784  790  791  797  817  823  829  830  836  842  851  856  857  863  872  877  884  886  889  892  904  910  916  919  922  937  941  953  965  970  973  976  979  982  989  997  1009  1012  1013  1015  1016  1018  1019  1021  1022  1024  62  2  26  32  38  41  44  50  68  71  72  74  83  100  103  106  109  11  14  21  29  32  5  52  81  19  67  88  133  138  141  180  190  209  223  227  228  235  243  258  268  275  277  286  299  331  351  367  375  387  405  423  428  439  456  457  463  476  496  501  504  507  510  520  530  532  554  581  595  598  602  620  622  637  644  645  657  675  685  688  710  725  745  763  764  773  775  816  823  855  863  864  867  868  889  891  902  914  926  939  944  955  958  978  991  995  1000  1003  1007  1020  1030  1038  1046  1049  1059  1085  1088  1102  1104  1107  1125  1129  1148  1151  1158  1168  1176  1197  1205  1220  1242  1244  1246  1248  1252  1259  1270  1271  1274  1277  1283  1285  1288  1289  1301  1307  1310  1316  1319  1322  1328  1331  1333  1337  1342  1345  1349  1352  1361  1364  1370  1372  1375  1376  1378  1381  1385  1387  1390  1391  1394  1410  1411  1413  1420  1423  1426  1429  1432  1434  1437  1440  1441  1455  1458  1461  1464  1469  1472  1473  1478  1485  1487  1499  1500  1502  1509  1515  1520  1523  1524  1526  1536  1539  1542  1547  1552  1561  1564  1576  1577  1582  1585  1587  1591  1596  1605  1606  1611  1612  1618  1621  1623  1624  1626  1629  1644  1645  1648  1651  1663  1668  1678  1687  1692  1699  1701  1702  1704  1705  1713  1721  1729  1732  1736  1739  1742  1753  1754  1756  1760  1765  1774  1775  1787  1792  1795  1805  1811  1820  1822  1825  1826  1829  1832  1838  1847  1850  1873  1880  1885  1888  1889  1892  1894  1897  1898  1903  1906  1911  1927  1936  1940  1972  1974  1980  1989  1990  1995  1999  2011  2013  2016  2017  2026  2027  2029  2036  2039  2042  2045  2057  2077  2078  2081  2084  2086  2087  2089  2090  2092  2099  2101  2109  2110  2119  2127  2134  2143  2145  2152  2155  2163  2172  2175  2178  2187  2191  2196  2199  2202  2209  2214  2215  2222  2229  2231  2232  2234  2240  2244  2247  2256  2259  2274  2277  2280  2283  2285  2288  2292  2294  2298  2306  2315  2326  2335  2337  2350  2353  2362  2364  2371  2376  2379  2381  2382  2384  2385  2394  2397  2400  2406  2411  2418  2420  2429  2430  2436  2438  2441  2442  2444  2445  2450  2451  2463  2468  2470  2477  2486  2491  2497  2498  2528  2529  2531  2543  2552  2559  2568  2577  2585  2589  2595  2598  2601  2607  2621  2648  2649  2652  2681  2691  2693  2706  2712  2714  2723  2724  2726  2727  2732  2735  2747  2748  2756  2759  2762  2771  2773  2776  2815  2817  2832  2846  2847  2866  2869  2875  2879  2906  2930  2931  14  15  32  33  53  66  91  110  126  128  141  144  154  206  229  251  277  278  309  355  356  377  389  395  444  467  478  506  525  537  538  557  584  589  595  614  625  646  660  685  720  726  776  787  797  800  810  823  828  832  851  852  855  865  869  871  979  997  1009  1012  1013  1016  1017  1020  1021  1081  1135  1184  1218  1253  1260  1273  1279  1283  1296  1299  1302  1315  1347  1406  1412  1436  1445  1448  1463  1466  1475  1484  1487  1490  1499  1502  1505  1508  1534  1564  1567  1570  1588  1594  1655  1673  1711  1714  1717  1720  1731  1737  1740  1749  1752  1761  1772  1774  1777  1781  1794  55  60  50  53  57  14  25  30  32  62  86  104  126  129  155  45  47  138  208  215  216  226  228  230  236  267  332  363  394  429  437  439  452  454  459  461  468  480  482  492  499  501  511  513  516  549  567  582  590  595  610  642  644  658  669  685  688  702  708  741  775  793  805  100  166  238  264  320  326  5  9  17  20  21  25  34  39  40  50  60  72  73  77  82  83  87  92  99  103  108  109  118  123  128  129  133  138  139  143  148  153  158  168  178  188  198  205  214  224  231  240  267  273  279  364  436  446  472  482  492  497  502  503  507  513  517  522  527  532  533  537  542  552  562  582  589  598  628  638  648  653  685  691  694  740  747  756  766  771  776  786  791  811  816  821  826  836  846  847  856  866  871  16  27  40  51  14  24  25  27  57  66  71  90  93  99  121  124  150  91  97  102  108  120  132  135  144  147  153  156  162  168  171  197  200  215  216  217  220  14  29  33  34  35  65  79  98  101  107  129  132  158  44  64  71  78  83  88  149  157  78  86  98  107  108  124  141  33  36  36  50  53  58  34  40  41  42  46  48  52  53  54  58  60  64  65  69  71  76  80  81  83  86  90  95  97  103  104  105  13  41  60  89  112  146  154  160  13  41  91  113  147  155  161  25  52  56  60  26  26  31  34  16  21  27  31  45  34  36  39  40  41  45  46  47  51  52  53  58  59  60  65  66  67  70  71  72  73  77  78  79  82  83  84  87  88  89  93  94  95  96  100  101  102  105  106  107  111  112  113  115  119  120  124  126  128  132  135  137  139  143  144  146  151  153  160  162  164  45  45  34  39  40  45  47  51  53  55  57  61  65  68  69  73  75  80  81  83  84  89  93  97  102  33  39  40  46  48  52  53  55  59  61  63  67  71  75  76  81  83  85  91  95  97  99  106  44  23  57  7  11  12  14  15  16  17  25  26  27  29  50  63  67  92  105  107  120  152  155  170  176  191  208  229  249  288  295  323  326  340  373  392  415  416  430  458  484  489  503  523  527  557  580  584  585  590  601  602  609  622  661  731  743  762  800  802  42  84  135  147  159  200  201  206  210  214  215  227  229  234  235  266  329  363  369  375  377  384  386  394  395  406  412  420  431  434  440  443  449  453  509  515  520  522  532  533  537  541  547  554  559  564  591  592  596  598  607  618  620  626  629  636  641  646  655  658  661  43  44  45  49  50  51  55  57  60  61  62  66  68  72  73  77  79  83  87  88  90  93  97  101  103  108  110 | LIGSASAVYH**P**PSWTAWIAPK  IGSASAVYHP**P**SWTAWIAPKP  HPPSWTAWIA**P**KPWTAWKVHP  PSWTAWIAPK**P**WTAWKVHPPA  KPWTAWKVHP**P**AWTAWKAHPP  PAWTAWKAHP**P**AWTAWKATPK  PPAWTAWKAT**P**KPWTAWKAPP  KPWTAWKAPP**P**AWTAWKATPK  KPWTAWKAPP**P**TWTAWKATPK  PPTWTAWKAT**P**KPWTAWKAPP  KPWTAWKAPP**P**AWTAWKATLK  LKPWTAWKAT**P**KPWTAWKATP  PKPWTAWKAT**P**KPWTAWKATP  PKPWTAWKAT**P**KPWTVWKATP  PKPWTVWKAT**P**KPWTAWKATP  PKPWTAWKAT**P**KPWTAWKAPP  KPWTAWKAPP**P**AWSAWKATPK  PPAWSAWKAT**P**KPWTVWKATP  PKPWTVWKAT**P**KPWTAWKAPP  PPAWSAWKAT**P**KPWTAWKATP  PKPWTAWKAT**P**KPWTAWKVPP  KPWTAWKVPP**P**AWTAWKAHPP  PPAWTAWKAT**P**KPWTAWKATP  PKPWTVWKAT**P**KPWTAWRATP  PKPWTAWRAT**P**PPTWTAWHGH  KPWTAWRATP**P**PTWTAWHGHG  PWTAWRATPP**P**TWTAWHGHGY  HGHGYGGYGK**P**GKPGKPGSKG  GYGGYGKPGK**P**GKPGSKGPRG  GYGKPGKPGK**P**GSKGPRGPAG  GKPGKPGSKG**P**RGPAGPPGAT  GKPGSKGPRG**P**AGPPGATGKT  GSKGPRGPAG**P**PGATGKTGRT  SKGPRGPAGP**P**GATGKTGRTG  GRTGATGKRG**P**PGYPGKPGVP  RTGATGKRGP**P**GYPGKPGVPG  ATGKRGPPGY**P**GKPGVPGRNG  KRGPPGYPGK**P**GVPGRNGYVH  PPGYPGKPGV**P**GRNGYVHIVF  HGHGWTAWTA**P**PAYGGYAPPA  GHGWTAWTAP**P**AYGGYAPPAT  CVLVVLLLLL**P**YGDLITNNYI  YIRGAARKVT**P**WRRNLKTRDV  RCSCKLIRTR**P**LMYHVCVCCV  VLLCIFLVLF**P**MATLQLDGDQ  KKRDWEYHAH**P**KPNSFWTLVV  VLLLALLLLL**P**LSTAQDAEGS  RLKCVNSRCC**P**TTDGCCGDTT  LLVVALKPMM**P**KLAVVLLVLL  AVVLLVLLIL**P**LSYFDAAGGQ  HWRRGHGCSC**P**GGPCSCGHGR  LLMMELKPMM**P**KLEMMLLVLL  EMMLLVLLIL**P**LSSFSAAGEQ  GRCCLNRVCG**P**MCCPASHCYC  LNRVCGPMCC**P**ASHCYCIYHR  LTSFIETEAG**P**VNEAGVERLF  LFRALVGRGC**P**ADCPNTCDSS  LVGRGCPADC**P**NTCDSSNKCS  DSSNKCSPGF**P**GGPFGPSCKN  GRDPAPCCQH**P**IETCCRRRRC  QFTAADDMEY**P**KWLRGLSTDS  SERGCWLCLG**P**NACCRGSVCH  CRGSVCHDYC**P**RRPCYDHCVS  VALVLTYLIH**P**LDASSSYSFF  PPAFSAASFA**P**PGVKAGEKIF  PAFSAASFAP**P**GVKAGEKIFK  VEKGAGHKQG**P**NLNGLFGRQS  ENTLYDYLLN**P**KKYIPGTKMV  DYLLNPKKYI**P**GTKMVFPGLK  KYIPGTKMVF**P**GLKKPQDRAD  TKMVFPGLKK**P**QDRADLIAYL  KTETQPVMMV**P**QTETKAGAGF  VKDYRLTYYT**P**DYVVRDTDIL  TDILAAFRMT**P**QLGVPPEECG  AFRMTPQLGV**P**PEECGAAVAA  FRMTPQLGVP**P**EECGAAVAAE  RYKGRCYDIE**P**VPGEDNQYIA  KGRCYDIEPV**P**GEDNQYIAYV  RALRLEDLRI**P**PAYVKTFVGP  ALRLEDLRIP**P**AYVKTFVGPP  PAYVKTFVGP**P**HGIQVERDKL  GRGLLGCTIK**P**KLGLSAKNYG  TKDDENVNSQ**P**FMRWRDRFLF  RAVCAKELGV**P**IIMHDYLTGG  IYFTQDWCSM**P**GVMPVASGGI  QDWCSMPGVM**P**VASGGIHVWH  ASGGIHVWHM**P**ALVEIFGDDA  LQFGGGTLGH**P**WGNAPGAAAN  GTLGHPWGNA**P**GAAANRVALE  DVIRSACKWS**P**ELAAACEVWK  FKYEIKEGDC**P**VQSGKTWQDC  SVATQTCQIT**P**AEGPVVTAQY  QTCQITPAEG**P**VVTAQYDCLG  AQYDCLGCVH**P**ISTQSPDLEP  GCVHPISTQS**P**DLEPILRHGI  PISTQSPDLE**P**ILRHGIQYFN  CSKENFLFLT**P**DCKSLWNGDT  ASFSQNCDIY**P**GKDFVQPPTK  DIYPGKDFVQ**P**PTKICVGCPR  IYPGKDFVQP**P**TKICVGCPRD  QPPTKICVGC**P**RDIPTNSPEL  KICVGCPRDI**P**TNSPELEETL  GCPRDIPTNS**P**ELEETLTHTI  LDCNAEVYVV**P**WEKKIYPTVN  YVVPWEKKIY**P**TVNCQPLGMI  KKIYPTVNCQ**P**LGMISLMKRP  PLGMISLMKR**P**PGFSPFRSSR  SLMKRPPGFS**P**FRSSRIGEIK  GEIKEETTVS**P**PHTSMAPAQD  EIKEETTVSP**P**HTSMAPAQDE  TVSPPHTSMA**P**AQDEERDSGK  LASSSEDSTT**P**SAQTQEKTEG  SAQTQEKTEG**P**TPIPSLAKPG  QTQEKTEGPT**P**IPSLAKPGVT  QEKTEGPTPI**P**SLAKPGVTVT  GPTPIPSLAK**P**GVTVTFSDFQ  QDSDLIATMM**P**PISPAPIQSD  DSDLIATMMP**P**ISPAPIQSDD  LIATMMPPIS**P**APIQSDDDWI  ATMMPPISPA**P**IQSDDDWIPD  APIQSDDDWI**P**DIQIDPNGLS  DDWIPDIQID**P**NGLSFNPISD  QIDPNGLSFN**P**ISDFPDTTSP  GLSFNPISDF**P**DTTSPKCPGR  PISDFPDTTS**P**KCPGRPWKSV  DFPDTTSPKC**P**GRPWKSVSEI  DTTSPKCPGR**P**WKSVSEINPT  RPWKSVSEIN**P**TTQMKESYYF  VLLTICLLLF**P**LTALPMDGDE  CLLLFPLTAL**P**MDGDEPANRP  LTALPMDGDE**P**ANRPVERMQD  PMDGDEPANR**P**VERMQDNISS  MQDNISSEQY**P**LFEKRRDCCT  GQEEGQEEDI**P**PVTCVQNGLR  QEEGQEEDIP**P**VTCVQNGLRY  LRYHDRDVWK**P**VPCQICVCDN  YHDRDVWKPV**P**CQICVCDNGN  DVICDELKDC**P**NAKVPTDECC  ELKDCPNAKV**P**TDECCPVCPE  NAKVPTDECC**P**VCPEGQESPT  VPTDECCPVC**P**EGQESPTDQE  CPVCPEGQES**P**TDQETTGVEG  TDQETTGVEG**P**KGDTGPRGPR  GVEGPKGDTG**P**RGPRGPAGPP  GPKGDTGPRG**P**RGPAGPPGRD  GDTGPRGPRG**P**AGPPGRDGIP  GPRGPRGPAG**P**PGRDGIPGQP  PRGPRGPAGP**P**GRDGIPGQPG  PAGPPGRDGI**P**GQPGLPGPPG  PPGRDGIPGQ**P**GLPGPPGPPG  RDGIPGQPGL**P**GPPGPPGPPG  GIPGQPGLPG**P**PGPPGPPGPP  IPGQPGLPGP**P**GPPGPPGPPG  GQPGLPGPPG**P**PGPPGPPGLG  QPGLPGPPGP**P**GPPGPPGLGG  GLPGPPGPPG**P**PGPPGLGGNF  LPGPPGPPGP**P**GPPGLGGNFA  GPPGPPGPPG**P**PGLGGNFAPQ  PPGPPGPPGP**P**GLGGNFAPQL  GPPGLGGNFA**P**QLSYGYDEKS  YDEKSTGISV**P**GPMGPSGPRG  EKSTGISVPG**P**MGPSGPRGLP  TGISVPGPMG**P**SGPRGLPGPP  SVPGPMGPSG**P**RGLPGPPGAP  PMGPSGPRGL**P**GPPGAPGPQG  GPSGPRGLPG**P**PGAPGPQGFQ  PSGPRGLPGP**P**GAPGPQGFQG  PRGLPGPPGA**P**GPQGFQGPPG  GLPGPPGAPG**P**QGFQGPPGEP  GAPGPQGFQG**P**PGEPGEPGAS  APGPQGFQGP**P**GEPGEPGASG  PQGFQGPPGE**P**GEPGASGPMG  FQGPPGEPGE**P**GASGPMGPRG  GEPGEPGASG**P**MGPRGPPGPP  GEPGASGPMG**P**RGPPGPPGKN  GASGPMGPRG**P**PGPPGKNGDD  ASGPMGPRGP**P**GPPGKNGDDG  GPMGPRGPPG**P**PGKNGDDGEA  PMGPRGPPGP**P**GKNGDDGEAG  KNGDDGEAGK**P**GRPGERGPPG  DDGEAGKPGR**P**GERGPPGPQG  GKPGRPGERG**P**PGPQGARGLP  KPGRPGERGP**P**GPQGARGLPG  GRPGERGPPG**P**QGARGLPGTA  PPGPQGARGL**P**GTAGLPGMKG  ARGLPGTAGL**P**GMKGHRGFSG  GLDGAKGDAG**P**AGPKGEPGSP  GAKGDAGPAG**P**KGEPGSPGEN  DAGPAGPKGE**P**GSPGENGAPG  PAGPKGEPGS**P**GENGAPGQMG  EPGSPGENGA**P**GQMGPRGLPG  GENGAPGQMG**P**RGLPGERGRP  APGQMGPRGL**P**GERGRPGAPG  PRGLPGERGR**P**GAPGPAGARG  LPGERGRPGA**P**GPAGARGNDG  GERGRPGAPG**P**AGARGNDGAT  GNDGATGAAG**P**PGPTGPAGPP  NDGATGAAGP**P**GPTGPAGPPG  GATGAAGPPG**P**TGPAGPPGFP  GAAGPPGPTG**P**AGPPGFPGAV  GPPGPTGPAG**P**PGFPGAVGAK  PPGPTGPAGP**P**GFPGAVGAKG  PTGPAGPPGF**P**GAVGAKGEGG  GAVGAKGEGG**P**QGPRGSEGPQ  GAKGEGGPQG**P**RGSEGPQGVR  GPQGPRGSEG**P**QGVRGEPGPP  SEGPQGVRGE**P**GPPGPAGAAG  GPQGVRGEPG**P**PGPAGAAGPA  PQGVRGEPGP**P**GPAGAAGPAG  GVRGEPGPPG**P**AGAAGPAGNP  GPPGPAGAAG**P**AGNPGADGQP  PAGAAGPAGN**P**GADGQPGAKG  PAGNPGADGQ**P**GAKGANGAPG  QPGAKGANGA**P**GIAGAPGFPG  ANGAPGIAGA**P**GFPGARGPSG  APGIAGAPGF**P**GARGPSGPQG  GAPGFPGARG**P**SGPQGPSGPP  GFPGARGPSG**P**QGPSGPPGPK  GARGPSGPQG**P**SGPPGPKGNS  GPSGPQGPSG**P**PGPKGNSGEP  PSGPQGPSGP**P**GPKGNSGEPG  GPQGPSGPPG**P**KGNSGEPGAP  PPGPKGNSGE**P**GAPGSKGDTG  PKGNSGEPGA**P**GSKGDTGAKG  SKGDTGAKGE**P**GPTGIQGPPG  GDTGAKGEPG**P**TGIQGPPGPA  GEPGPTGIQG**P**PGPAGEEGKR  EPGPTGIQGP**P**GPAGEEGKRG  GPTGIQGPPG**P**AGEEGKRGAR  EEGKRGARGE**P**GPAGLPGPPG  GKRGARGEPG**P**AGLPGPPGER  ARGEPGPAGL**P**GPPGERGGPG  GEPGPAGLPG**P**PGERGGPGSR  EPGPAGLPGP**P**GERGGPGSRG  LPGPPGERGG**P**GSRGFPGADG  ERGGPGSRGF**P**GADGVAGPKG  GFPGADGVAG**P**KGPAGERGAP  GADGVAGPKG**P**AGERGAPGPA  PKGPAGERGA**P**GPAGPKGSPG  GPAGERGAPG**P**AGPKGSPGEA  GERGAPGPAG**P**KGSPGEAGRP  APGPAGPKGS**P**GEAGRPGEAG  PKGSPGEAGR**P**GEAGLPGAKG  EAGRPGEAGL**P**GAKGLTGSPG  LPGAKGLTGS**P**GSPGPDGKTG  AKGLTGSPGS**P**GPDGKTGPPG  GLTGSPGSPG**P**DGKTGPPGPA  GSPGPDGKTG**P**PGPAGQDGRP  SPGPDGKTGP**P**GPAGQDGRPG  GPDGKTGPPG**P**AGQDGRPGPP  PPGPAGQDGR**P**GPPGPPGARG  GPAGQDGRPG**P**PGPPGARGQA  PAGQDGRPGP**P**GPPGARGQAG  GQDGRPGPPG**P**PGARGQAGVM  QDGRPGPPGP**P**GARGQAGVMG  ARGQAGVMGF**P**GPKGAAGEPG  GQAGVMGFPG**P**KGAAGEPGKA  FPGPKGAAGE**P**GKAGERGVPG  EPGKAGERGV**P**GPPGAVGPAG  GKAGERGVPG**P**PGAVGPAGKD  KAGERGVPGP**P**GAVGPAGKDG  GVPGPPGAVG**P**AGKDGEAGAQ  GKDGEAGAQG**P**PGPAGPAGER  KDGEAGAQGP**P**GPAGPAGERG  GEAGAQGPPG**P**AGPAGERGEQ  GAQGPPGPAG**P**AGERGEQGPA  GPAGERGEQG**P**AGSPGFQGLP  ERGEQGPAGS**P**GFQGLPGPAG  PAGSPGFQGL**P**GPAGPPGEAG  GSPGFQGLPG**P**AGPPGEAGKP  GFQGLPGPAG**P**PGEAGKPGEQ  FQGLPGPAGP**P**GEAGKPGEQG  PAGPPGEAGK**P**GEQGVPGDLG  EAGKPGEQGV**P**GDLGAPGPSG  EQGVPGDLGA**P**GPSGARGERG  GVPGDLGAPG**P**SGARGERGFP  PSGARGERGF**P**GERGVQGPPG  GFPGERGVQG**P**PGPAGPRGAN  FPGERGVQGP**P**GPAGPRGANG  GERGVQGPPG**P**AGPRGANGAP  GVQGPPGPAG**P**RGANGAPGND  PAGPRGANGA**P**GNDGAKGDAG  NDGAKGDAGA**P**GAPGSQGAPG  AKGDAGAPGA**P**GSQGAPGLQG  APGAPGSQGA**P**GLQGMPGERG  SQGAPGLQGM**P**GERGAAGLPG  MPGERGAAGL**P**GPKGDRGDAG  GERGAAGLPG**P**KGDRGDAGPK  GPKGDRGDAG**P**KGADGAPGKD  DAGPKGADGA**P**GKDGVRGLTG  GKDGVRGLTG**P**IGPPGPAGAP  GVRGLTGPIG**P**PGPAGAPGDK  VRGLTGPIGP**P**GPAGAPGDKG  GLTGPIGPPG**P**AGAPGDKGEA  PIGPPGPAGA**P**GDKGEAGPSG  GAPGDKGEAG**P**SGPAGPTGAR  GDKGEAGPSG**P**AGPTGARGAP  GEAGPSGPAG**P**TGARGAPGDR  PAGPTGARGA**P**GDRGEPGPPG  ARGAPGDRGE**P**GPPGPAGFAG  GAPGDRGEPG**P**PGPAGFAGPP  APGDRGEPGP**P**GPAGFAGPPG  GDRGEPGPPG**P**AGFAGPPGAD  GPPGPAGFAG**P**PGADGQPGAK  PPGPAGFAGP**P**GADGQPGAKG  FAGPPGADGQ**P**GAKGEPGDAG  ADGQPGAKGE**P**GDAGAKGDAG  GDAGAKGDAG**P**PGPAGPAGPP  DAGAKGDAGP**P**GPAGPAGPPG  GAKGDAGPPG**P**AGPAGPPGPI  GDAGPPGPAG**P**AGPPGPIGNV  GPPGPAGPAG**P**PGPIGNVGAP  PPGPAGPAGP**P**GPIGNVGAPG  GPAGPAGPPG**P**IGNVGAPGPK  PPGPIGNVGA**P**GPKGARGSAG  GPIGNVGAPG**P**KGARGSAGPP  GPKGARGSAG**P**PGATGFPGAA  PKGARGSAGP**P**GATGFPGAAG  SAGPPGATGF**P**GAAGRVGPPG  GFPGAAGRVG**P**PGPSGNAGPP  FPGAAGRVGP**P**GPSGNAGPPG  GAAGRVGPPG**P**SGNAGPPGPP  GPPGPSGNAG**P**PGPPGPAGKE  PPGPSGNAGP**P**GPPGPAGKEG  GPSGNAGPPG**P**PGPAGKEGSK  PSGNAGPPGP**P**GPAGKEGSKG  GNAGPPGPPG**P**AGKEGSKGPR  GPAGKEGSKG**P**RGETGPAGRP  GSKGPRGETG**P**AGRPGEVGPP  PRGETGPAGR**P**GEVGPPGPPG  GPAGRPGEVG**P**PGPPGPAGEK  PAGRPGEVGP**P**GPPGPAGEKG  GRPGEVGPPG**P**PGPAGEKGAP  RPGEVGPPGP**P**GPAGEKGAPG  GEVGPPGPPG**P**AGEKGAPGAD  PPGPAGEKGA**P**GADGPAGAPG  GEKGAPGADG**P**AGAPGTPGPQ  APGADGPAGA**P**GTPGPQGIAG  ADGPAGAPGT**P**GPQGIAGQRG  GPAGAPGTPG**P**QGIAGQRGVV  IAGQRGVVGL**P**GQRGERGFPG  LPGQRGERGF**P**GLPGPSGEPG  QRGERGFPGL**P**GPSGEPGKQG  GERGFPGLPG**P**SGEPGKQGPS  FPGLPGPSGE**P**GKQGPSGASG  GPSGEPGKQG**P**SGASGERGPP  GPSGASGERG**P**PGPMGPPGLA  PSGASGERGP**P**GPMGPPGLAG  GASGERGPPG**P**MGPPGLAGPP  GERGPPGPMG**P**PGLAGPPGES  ERGPPGPMGP**P**GLAGPPGESG  GPMGPPGLAG**P**PGESGREGAP  PMGPPGLAGP**P**GESGREGAPG  PPGESGREGA**P**GAEGSPGRDG  REGAPGAEGS**P**GRDGSPGAKG  AEGSPGRDGS**P**GAKGDRGETG  GAKGDRGETG**P**AGPPGAPGAP  GDRGETGPAG**P**PGAPGAPGAP  DRGETGPAGP**P**GAPGAPGAPG  ETGPAGPPGA**P**GAPGAPGPVG  PAGPPGAPGA**P**GAPGPVGPAG  PPGAPGAPGA**P**GPVGPAGKSG  GAPGAPGAPG**P**VGPAGKSGDR  GAPGAPGPVG**P**AGKSGDRGET  GKSGDRGETG**P**AGPAGPIGPV  GDRGETGPAG**P**AGPIGPVGAR  GETGPAGPAG**P**IGPVGARGPA  GPAGPAGPIG**P**VGARGPAGPQ  GPIGPVGARG**P**AGPQGPRGDK  GPVGARGPAG**P**QGPRGDKGET  GARGPAGPQG**P**RGDKGETGEQ  GHRGFSGLQG**P**PGPPGSPGEQ  HRGFSGLQGP**P**GPPGSPGEQG  GFSGLQGPPG**P**PGSPGEQGPS  FSGLQGPPGP**P**GSPGEQGPSG  LQGPPGPPGS**P**GEQGPSGASG  GPPGSPGEQG**P**SGASGPAGPR  GEQGPSGASG**P**AGPRGPPGSA  GPSGASGPAG**P**RGPPGSAGSP  GASGPAGPRG**P**PGSAGSPGKD  ASGPAGPRGP**P**GSAGSPGKDG  PRGPPGSAGS**P**GKDGLNGLPG  SPGKDGLNGL**P**GPIGPPGPRG  GKDGLNGLPG**P**IGPPGPRGRT  LNGLPGPIGP**P**GPRGRTGDAG  GLPGPIGPPG**P**RGRTGDAGPA  GPRGRTGDAG**P**AGPPGPPGPP  GRTGDAGPAG**P**PGPPGPPGPP  RTGDAGPAGP**P**GPPGPPGPPG  GDAGPAGPPG**P**PGPPGPPGPP  DAGPAGPPGP**P**GPPGPPGPPS  GPAGPPGPPG**P**PGPPGPPSGG  PAGPPGPPGP**P**GPPGPPSGGY  GPPGPPGPPG**P**PGPPSGGYDL  PPGPPGPPGP**P**GPPSGGYDLS  GPPGPPGPPG**P**PSGGYDLSFL  PPGPPGPPGP**P**SGGYDLSFLP  PSGGYDLSFL**P**QPPQEKAHDG  GGYDLSFLPQ**P**PQEKAHDGGR  GYDLSFLPQP**P**QEKAHDGGRY  LSQQIENIRS**P**EGSRKNPART  IRSPEGSRKN**P**ARTCRDLKMC  DWKSGEYWID**P**NQGCNLDAIK  NMETGETCVY**P**TQPSVAQKNW  TGETCVYPTQ**P**SVAQKNWYIS  AQKNWYISKN**P**KEKRHVWYGE  QFEYGGQGSD**P**ADVAIQLTFL  EYKTTKTSRL**P**IIDVAPLDVG  TSRLPIIDVA**P**LDVGAPDQEF  IDVAPLDVGA**P**DQEFGFDVGP  PDQEFGFDVG**P**ACFLLFCAPG  LTYNDKDVWK**P**EPCQICVCDS  YNDKDVWKPE**P**CQICVCDSGN  EVICEDTSDC**P**NAEIPFGECC  DTSDCPNAEI**P**FGECCPICPD  NAEIPFGECC**P**ICPDVDASPV  IPFGECCPIC**P**DVDASPVYPE  CPICPDVDAS**P**VYPESAGVEG  CPDVDASPVY**P**ESAGVEGPKG  VYPESAGVEG**P**KGDTGPRGDR  GVEGPKGDTG**P**RGDRGLPGPP  DTGPRGDRGL**P**GPPGRDGIPG  GPRGDRGLPG**P**PGRDGIPGQP  PRGDRGLPGP**P**GRDGIPGQPG  LPGPPGRDGI**P**GQPGLPGPPG  GIPGQPGLPG**P**PGPPGPPGLG  IPGQPGLPGP**P**GPPGPPGLGG  GQPGLPGPPG**P**PGPPGLGGNF  QPGLPGPPGP**P**GPPGLGGNFA  GLPGPPGPPG**P**PGLGGNFAPQ  LPGPPGPPGP**P**GLGGNFAPQM  GPPGLGGNFA**P**QMSYGYDEKS  YDEKSAGVAV**P**GPMGPAGPRG  EKSAGVAVPG**P**MGPAGPRGLP  AGVAVPGPMG**P**AGPRGLPGPP  AVPGPMGPAG**P**RGLPGPPGAP  GPAGPRGLPG**P**PGAPGPQGFQ  GEPGEPGASG**P**MGPRGPAGPP  GEPGASGPMG**P**RGPAGPPGKN  GASGPMGPRG**P**AGPPGKNGDD  GPMGPRGPAG**P**PGKNGDDGEA  KNGDDGEAGK**P**GRPGQRGPPG  GKPGRPGQRG**P**PGPQGARGLP  GRPGQRGPPG**P**QGARGLPGTA  GLDGAKGQPG**P**AGPKGEPGSP  GAKGQPGPAG**P**KGEPGSPGEN  GLPGERGRPG**P**SGPAGARGND  GERGRPGPSG**P**AGARGNDGAP  GNDGAPGAAG**P**PGPTGPAGPP  GAPGAAGPPG**P**TGPAGPPGFP  GAAGPPGPTG**P**AGPPGFPGAA  GPPGPTGPAG**P**PGFPGAAGAK  GAAGAKGETG**P**QGARGSEGPQ  GPQGARGSEG**P**QGSRGEPGPP  GPQGSRGEPG**P**PGPAGAAGPA  GSRGEPGPPG**P**AGAAGPAGNP  GAPGFPGARG**P**SGPQGPSGAP  GFPGARGPSG**P**QGPSGAPGPK  GARGPSGPQG**P**SGAPGPKGNS  GPQGPSGAPG**P**KGNSGEPGAP  GDTGAKGEPG**P**AGVQGPPGPA  GEPGPAGVQG**P**PGPAGEEGKR  GPAGVQGPPG**P**AGEEGKRGAR  GKRGARGEPG**P**AGLPGPAGER  GEPGPAGLPG**P**AGERGAPGSR  GFPGADGIAG**P**KGPPGERGSP  GADGIAGPKG**P**PGERGSPGAV  GERGSPGAVG**P**KGSPGEAGRP  GPDGKTGPPG**P**AGQDGRPGPA  GPAGQDGRPG**P**AGPPGARGQA  GQDGRPGPAG**P**PGARGQAGVM  GQAGVMGFPG**P**KGAAGEPGKP  GKPGERGAPG**P**PGAVGAAGKD  GKDGEAGAQG**P**PGPTGPAGER  GEAGAQGPPG**P**TGPAGERGEQ  GAQGPPGPTG**P**AGERGEQGPA  GPAGERGEQG**P**AGAPGFQGLP  GAPGFQGLPG**P**AGPPGEAGKP  GVPGNAGAPG**P**AGARGERGFP  GFPGERGVQG**P**PGPQGPRGAN  GERGVQGPPG**P**QGPRGANGAP  GVQGPPGPQG**P**RGANGAPGND  GAPGAPGNEG**P**PGLEGMPGER  GAKGDRGDPG**P**KGADGAPGKD  GKDGLRGLTG**P**IGPPGPAGAP  GLRGLTGPIG**P**PGPAGAPGDK  GAPGDKGEAG**P**PGPAGPTGAR  GDKGEAGPPG**P**AGPTGARGAP  GEAGPPGPAG**P**TGARGAPGDR  GDAGAKGDAG**P**PGPAGPTGAP  GAKGDAGPPG**P**AGPTGAPGPA  GDAGPPGPAG**P**TGAPGPAGVG  GPAGPTGAPG**P**AGVGAPGPKG  GAPGPAGVGA**P**GPKGARGSAG  PGPAGVGAPG**P**KGARGSAGPP  GFPGAAGRVG**P**PGPSGNIGLP  FPGAAGRVGP**P**GPSGNIGLPG  GAAGRVGPPG**P**SGNIGLPGPP  PPGPSGNIGL**P**GPPGPAGKGS  GPSGNIGLPG**P**PGPAGKGSKG  PSGNIGLPGP**P**GPAGKGSKGP  GNIGLPGPPG**P**AGKGSKGPRG  PGPAGKGSKG**P**RGETGPAGRP  GSKGPRGETG**P**AGRPGEPGPA  PRGETGPAGR**P**GEPGPAGPPG  ETGPAGRPGE**P**GPAGPPGPPG  GRPGEPGPAG**P**PGPPGEKGSP  RPGEPGPAGP**P**GPPGEKGSPG  EPGPAGPPGP**P**GEKGSPGADG  PPGPPGEKGS**P**GADGPIGAPG  GEKGSPGADG**P**IGAPGTPGPQ  SPGADGPIGA**P**GTPGPQGIAG  ADGPIGAPGT**P**GPQGIAGQRG  GPIGAPGTPG**P**QGIAGQRGVV  GERGPPGPMG**P**PGLAGPPGEA  ERGPPGPMGP**P**GLAGPPGEAG  GPMGPPGLAG**P**PGEAGREGAP  PMGPPGLAGP**P**GEAGREGAPG  PPGEAGREGA**P**GAEGAPGRDG  REGAPGAEGA**P**GRDGAAGPKG  GAPGRDGAAG**P**KGDRGETGPA  GPKGDRGETG**P**AGPPGAPGAP  PPGAPGAPGA**P**GPVGPAGKNG  GAPGAPGAPG**P**VGPAGKNGDR  GAPGAPGPVG**P**AGKNGDRGET  GKNGDRGETG**P**AGPAGPPGPA  GDRGETGPAG**P**AGPPGPAGAR  GETGPAGPAG**P**PGPAGARGPA  ETGPAGPAGP**P**GPAGARGPAG  GPPGPAGARG**P**AGPQGPRGDK  GPAGARGPAG**P**QGPRGDKGET  GHRGFSGLQG**P**PGPPGAPGEQ  HRGFSGLQGP**P**GPPGAPGEQG  FSGLQGPPGP**P**GAPGEQGPSG  LQGPPGPPGA**P**GEQGPSGASG  GPPGAPGEQG**P**SGASGPAGPR  GPSGASGPAG**P**RGPPGSAGAA  GASGPAGPRG**P**PGSAGAAGKD  ASGPAGPRGP**P**GSAGAAGKDG  AAGKDGLNGL**P**GPIGPPGPRG  GLNGLPGPIG**P**PGPRGRTGEV  LNGLPGPIGP**P**GPRGRTGEVG  GPRGRTGEVG**P**VGPPGPPGPP  GRTGEVGPVG**P**PGPPGPPGPP  RTGEVGPVGP**P**GPPGPPGPPG  EVGPVGPPGP**P**GPPGPPGPPS  PVGPPGPPGP**P**GPPGPPSGGF  PPGPPGPPGP**P**GPPSGGFDLS  PPGPPGPPGP**P**SGGFDLSFLP  PSGGFDLSFL**P**QPPQEKAHDG  GGFDLSFLPQ**P**PQEKAHDGGR  GFDLSFLPQP**P**QEKAHDGGRY  LSQQIENIRS**P**EGTRKNPART  IRSPEGTRKN**P**ARTCRDLKMC  NMETGETCVY**P**TQATIAQKNW  AQKNWYLSKN**P**KEKKHVWFGE  QFEYGGEGSN**P**ADVAIQLTFL  EYKTTKTSRL**P**IIDLAPMDVG  TSRLPIIDLA**P**MDVGAPDQEF  IDLAPMDVGA**P**DQEFGIDIGP  PDQEFGIDIG**P**VCFLLFCVPG  VSEASAGRKG**P**RGDKGPQGER  GRKGPRGDKG**P**QGERGPPGPP  GDKGPQGERG**P**PGPPGRDGED  DKGPQGERGP**P**GPPGRDGEDG  GPQGERGPPG**P**PGRDGEDGPP  PQGERGPPGP**P**GRDGEDGPPG  GPPGRDGEDG**P**PGPPGPPGPP  PPGRDGEDGP**P**GPPGPPGPPG  GRDGEDGPPG**P**PGPPGPPGLG  RDGEDGPPGP**P**GPPGPPGLGG  GEDGPPGPPG**P**PGPPGLGGNF  EDGPPGPPGP**P**GPPGLGGNFA  GPPGPPGPPG**P**PGLGGNFAAQ  PPGPPGPPGP**P**GLGGNFAAQY  LGGNFAAQYD**P**SKAADFGPGP  YDPSKAADFG**P**GPMGLMGPRG  PSKAADFGPG**P**MGLMGPRGPP  FGPGPMGLMG**P**RGPPGASGPP  GPMGLMGPRG**P**PGASGPPGPP  PMGLMGPRGP**P**GASGPPGPPG  GPRGPPGASG**P**PGPPGFQGVP  PRGPPGASGP**P**GPPGFQGVPG  GPPGASGPPG**P**PGFQGVPGEP  PPGASGPPGP**P**GFQGVPGEPG  PPGPPGFQGV**P**GEPGEPGQTG  PPGFQGVPGE**P**GEPGQTGPQG  FQGVPGEPGE**P**GQTGPQGPRG  GEPGEPGQTG**P**QGPRGPPGPP  GEPGQTGPQG**P**RGPPGPPGKA  GQTGPQGPRG**P**PGPPGKAGED  QTGPQGPRGP**P**GPPGKAGEDG  GPQGPRGPPG**P**PGKAGEDGHP  PQGPRGPPGP**P**GKAGEDGHPG  PPGKAGEDGH**P**GKPGRPGERG  KAGEDGHPGK**P**GRPGERGVAG  EDGHPGKPGR**P**GERGVAGPQG  GRPGERGVAG**P**QGARGFPGTP  VAGPQGARGF**P**GTPGPPGFKG  PQGARGFPGT**P**GPPGFKGIRG  GARGFPGTPG**P**PGFKGIRGHN  ARGFPGTPGP**P**GFKGIRGHNG  HNGLDGLTGQ**P**GAPGTKGEPG  LDGLTGQPGA**P**GTKGEPGAPG  QPGAPGTKGE**P**GAPGENGTPG  APGTKGEPGA**P**GENGTPGQPG  EPGAPGENGT**P**GQPGARGLPG  APGENGTPGQ**P**GARGLPGERG  TPGQPGARGL**P**GERGRIGAPG  LPGERGRIGA**P**GPAGARGSDG  GERGRIGAPG**P**AGARGSDGSA  GARGSDGSAG**P**TGPAGEIGPA  GSDGSAGPTG**P**AGEIGPAGNE  GPTGPAGEIG**P**AGNEGPTGPA  GEIGPAGNEG**P**TGPAGPRGEI  GPAGNEGPTG**P**AGPRGEIGLP  GNEGPTGPAG**P**RGEIGLPGSS  PAGPRGEIGL**P**GSSGPVGPPG  GEIGLPGSSG**P**VGPPGNPGAN  GLPGSSGPVG**P**PGNPGANGLP  LPGSSGPVGP**P**GNPGANGLPG  SSGPVGPPGN**P**GANGLPGAKG  PPGNPGANGL**P**GAKGAAGLPG  LPGAKGAAGL**P**GVAGAPGLPG  AAGLPGVAGA**P**GLPGPRGIPG  LPGVAGAPGL**P**GPRGIPGPPG  GVAGAPGLPG**P**RGIPGPPGPA  APGLPGPRGI**P**GPPGPAGPSG  GLPGPRGIPG**P**PGPAGPSGAR  LPGPRGIPGP**P**GPAGPSGARG  GPRGIPGPPG**P**AGPSGARGLV  GIPGPPGPAG**P**SGARGLVGEP  PSGARGLVGE**P**GPAGAKGESG  GARGLVGEPG**P**AGAKGESGNK  AKGESGNKGE**P**GAAGPPGPPG  GNKGEPGAAG**P**PGPPGPSGEE  NKGEPGAAGP**P**GPPGPSGEEG  GEPGAAGPPG**P**PGPSGEEGKR  EPGAAGPPGP**P**GPSGEEGKRG  GAAGPPGPPG**P**SGEEGKRGSN  EEGKRGSNGE**P**GSAGPPGPAG  GSNGEPGSAG**P**PGPAGLRGEP  SNGEPGSAGP**P**GPAGLRGEPG  GEPGSAGPPG**P**AGLRGEPGSR  PPGPAGLRGE**P**GSRGLPGADG  LRGEPGSRGL**P**GADGRAGVMG  GADGRAGVMG**P**AGNRGASGPV  GPAGNRGASG**P**VGAKGPNGDA  GASGPVGAKG**P**NGDAGRPGEP  AKGPNGDAGR**P**GEPGLMGPRG  PNGDAGRPGE**P**GLMGPRGFPG  GRPGEPGLMG**P**RGFPGADGRV  EPGLMGPRGF**P**GADGRVGPIG  GADGRVGPIG**P**AGNRGEPGNI  PIGPAGNRGE**P**GNIGFPGPKG  NRGEPGNIGF**P**GPKGPTGEPG  GEPGNIGFPG**P**KGPTGEPGKP  GNIGFPGPKG**P**TGEPGKPGEK  FPGPKGPTGE**P**GKPGEKGNVG  PKGPTGEPGK**P**GEKGNVGLAG  GEKGNVGLAG**P**RGAPGPEGNN  NVGLAGPRGA**P**GPEGNNGAQG  GLAGPRGAPG**P**EGNNGAQGPP  GPEGNNGAQG**P**PGVTGNQGAK  PEGNNGAQGP**P**GVTGNQGAKG  GNQGAKGETG**P**AGPPGFQGLP  GAKGETGPAG**P**PGFQGLPGPS  AKGETGPAGP**P**GFQGLPGPSG  PAGPPGFQGL**P**GPSGPAGEAG  GPPGFQGLPG**P**SGPAGEAGKP  GFQGLPGPSG**P**AGEAGKPGER  PSGPAGEAGK**P**GERGLHGEFG  ERGLHGEFGV**P**GPAGPRGERG  GLHGEFGVPG**P**AGPRGERGLP  GEFGVPGPAG**P**RGERGLPGES  PAGPRGERGL**P**GESGAVGPAG  GLPGESGAVG**P**AGPIGSRGPS  GESGAVGPAG**P**IGSRGPSGPP  GPAGPIGSRG**P**SGPPGPDGNK  GPIGSRGPSG**P**PGPDGNKGEP  PIGSRGPSGP**P**GPDGNKGEPG  GSRGPSGPPG**P**DGNKGEPGNV  PPGPDGNKGE**P**GNVGPAGAPG  GNKGEPGNVG**P**AGAPGPAGPG  EPGNVGPAGA**P**GPAGPGGIPG  GNVGPAGAPG**P**AGPGGIPGER  GPAGAPGPAG**P**GGIPGERGVA  APGPAGPGGI**P**GERGVAGVPG  IPGERGVAGV**P**GGKGEKGAPG  VPGGKGEKGA**P**GLRGDTGATG  ATGRDGARGL**P**GAIGAPGPAG  ARGLPGAIGA**P**GPAGGAGDRG  GLPGAIGAPG**P**AGGAGDRGEG  GGAGDRGEGG**P**AGPAGPAGAR  GDRGEGGPAG**P**AGPAGARGIP  GEGGPAGPAG**P**AGARGIPGER  PAGPAGARGI**P**GERGEPGPVG  ARGIPGERGE**P**GPVGPSGFAG  GIPGERGEPG**P**VGPSGFAGPP  GERGEPGPVG**P**SGFAGPPGAA  GPVGPSGFAG**P**PGAAGQPGAK  PVGPSGFAGP**P**GAAGQPGAKG  FAGPPGAAGQ**P**GAKGERGPKG  GQPGAKGERG**P**KGPKGETGPT  GAKGERGPKG**P**KGETGPTGAI  GPKGPKGETG**P**TGAIGPIGAS  GETGPTGAIG**P**IGASGPPGPV  GAIGPIGASG**P**PGPVGAAGPA  AIGPIGASGP**P**GPVGAAGPAG  GPIGASGPPG**P**VGAAGPAGPR  GPPGPVGAAG**P**AGPRGDAGPP  GPVGAAGPAG**P**RGDAGPPGMT  GPAGPRGDAG**P**PGMTGFPGAA  PAGPRGDAGP**P**GMTGFPGAAG  DAGPPGMTGF**P**GAAGRVGPPG  GFPGAAGRVG**P**PGPAGITGPP  FPGAAGRVGP**P**GPAGITGPPG  GAAGRVGPPG**P**AGITGPPGPP  GPPGPAGITG**P**PGPPGPAGKD  PPGPAGITGP**P**GPPGPAGKDG  GPAGITGPPG**P**PGPAGKDGPR  PAGITGPPGP**P**GPAGKDGPRG  GITGPPGPPG**P**AGKDGPRGLR  GPPGPAGKDG**P**RGLRGDVGPV  GPRGLRGDVG**P**VGRTGEQGIA  GRTGEQGIAG**P**PGFAGEKGPS  RTGEQGIAGP**P**GFAGEKGPSG  GPPGFAGEKG**P**SGEAGAAGPP  GPSGEAGAAG**P**PGTPGPQGIL  PSGEAGAAGP**P**GTPGPQGILG  EAGAAGPPGT**P**GPQGILGAPG  GAAGPPGTPG**P**QGILGAPGIL  TPGPQGILGA**P**GILGLPGSRG  ILGAPGILGL**P**GSRGERGLPG  LPGSRGERGL**P**GIAGATGEPG  LPGIAGATGE**P**GPLGVSGPPG  GIAGATGEPG**P**LGVSGPPGAR  GEPGPLGVSG**P**PGARGPSGPV  EPGPLGVSGP**P**GARGPSGPVG  GVSGPPGARG**P**SGPVGSPGPN  GPPGARGPSG**P**VGSPGPNGAP  ARGPSGPVGS**P**GPNGAPGEAG  GPSGPVGSPG**P**NGAPGEAGRD  PVGSPGPNGA**P**GEAGRDGNPG  APGEAGRDGN**P**GNDGPPGRDG  GRDGNPGNDG**P**PGRDGAPGFK  RDGNPGNDGP**P**GRDGAPGFKG  NDGPPGRDGA**P**GFKGERGAPG  APGFKGERGA**P**GNPGPSGALG  FKGERGAPGN**P**GPSGALGAPG  GERGAPGNPG**P**SGALGAPGPH  NPGPSGALGA**P**GPHGQVGPSG  GPSGALGAPG**P**HGQVGPSGKP  GAPGPHGQVG**P**SGKPGNRGDP  PHGQVGPSGK**P**GNRGDPGPVG  PSGKPGNRGD**P**GPVGPVGPAG  GKPGNRGDPG**P**VGPVGPAGAF  GNRGDPGPVG**P**VGPAGAFGPR  GDPGPVGPVG**P**AGAFGPRGLA  GPVGPAGAFG**P**RGLAGPQGPR  GAFGPRGLAG**P**QGPRGEKGEP  GPRGLAGPQG**P**RGEKGEPGDK  PQGPRGEKGE**P**GDKGHRGLPG  EPGDKGHRGL**P**GLKGHNGLQG  LKGHNGLQGL**P**GLAGQHGDQG  GLAGQHGDQG**P**PGNNGPAGPR  LAGQHGDQGP**P**GNNGPAGPRG  GDQGPPGNNG**P**AGPRGPPGPS  GPPGNNGPAG**P**RGPPGPSGPP  GNNGPAGPRG**P**PGPSGPPGKD  NNGPAGPRGP**P**GPSGPPGKDG  GPAGPRGPPG**P**SGPPGKDGRN  GPRGPPGPSG**P**PGKDGRNGLP  PRGPPGPSGP**P**GKDGRNGLPG  PPGKDGRNGL**P**GPIGPAGVRG  GKDGRNGLPG**P**IGPAGVRGSH  GRNGLPGPIG**P**AGVRGSHGSQ  GVRGSHGSQG**P**AGPPGPPGPP  GSHGSQGPAG**P**PGPPGPPGPP  SHGSQGPAGP**P**GPPGPPGPPG  GSQGPAGPPG**P**PGPPGPPGPN  SQGPAGPPGP**P**GPPGPPGPNG  GPAGPPGPPG**P**PGPPGPNGGG  PAGPPGPPGP**P**GPPGPNGGGY  GPPGPPGPPG**P**PGPNGGGYEV  PPGPPGPPGP**P**GPNGGGYEVG  GPPGPPGPPG**P**NGGGYEVGFD  FDAEYYRADQ**P**SLRPKDYEVD  YYRADQPSLR**P**KDYEVDATLK  LNNQIETLLT**P**EGSKKNPART  LLTPEGSKKN**P**ARTCRDLRLS  RTCRDLRLSH**P**EWSSGFYWID  EWSSGFYWID**P**NQGCTADAIR  TCIHASLEDI**P**TKTWYVSKNP  PTKTWYVSKN**P**KDKKHIWFGE  KTIIEYRTNK**P**SRLPILDIAP  EYRTNKPSRL**P**ILDIAPLDIG  PSRLPILDIA**P**LDIGGADQEF  ADQEFGLHIG**P**VCFKKFCVPG  WGRPGEMMEG**P**RGWLVLCVLA  SMVTEDLCRA**P**DGKKGEAGRP  GLKGDQGEPG**P**SGNPGKVGYP  GNPGKVGYPG**P**SGPLGARGIP  GKVGYPGPSG**P**LGARGIPGIK  PSGPLGARGI**P**GIKGTKGSPG  IPGIKGTKGS**P**GNIKDQPRPA  KGSPGNIKDQ**P**RPAFSAIRRN  SPGNIKDQPR**P**AFSAIRRNPP  RPAFSAIRRN**P**PMGGNVVIFD  PAFSAIRRNP**P**MGGNVVIFDT  FDTVITNQEE**P**YQNHSGRFVC  NHSGRFVCTV**P**GYYYFTFQVL  QGDQVWVEKD**P**KKGHIYQGSE  DSVFSGFLIF**P**SAASPFILFG  SPGVDMMDVG**P**SSLPHLGLKL  DMMDVGPSSL**P**HLGLKLLLLL  LKLLLLLLLL**P**LRGQANTGCY  GKDGYDGLPG**P**KGEPGIPAIP  YDGLPGPKGE**P**GIPAIPGIRG  PKGEPGIPAI**P**GIRGPKGQKG  GIPAIPGIRG**P**KGQKGEPGLP  IRGPKGQKGE**P**GLPGHPGKNG  QKGEPGLPGH**P**GKNGPMGPPG  GLPGHPGKNG**P**MGPPGMPGVP  GHPGKNGPMG**P**PGMPGVPGPM  GPPGMPGVPG**P**MGIPGEPGEE  VPGPMGIPGE**P**GEEGRYKQKF  VFTVTRQTHQ**P**PAPNSLIRFN  FTVTRQTHQP**P**APNSLIRFNA  VTRQTHQPPA**P**NSLIRFNAVL  LIRFNAVLTN**P**QGDYDTSTGK  TSTGKFTCKV**P**GLYYFVYHAS  DSVFSGFLLF**P**DDPFLLFGSF  LLMPTRMMRT**P**MLLALLALAT  LCLAGRADAK**P**GDAESGKGAA  YLDHWLGAPA**P**YPDPLEPKRE  DHWLGAPAPY**P**DPLEPKREVC  WLGAPAPYPD**P**LEPKREVCEL  APAPYPDPLE**P**KREVCELNPD  EPKREVCELN**P**DCDELADHIG  FQEAYRRFYG**P**VVPGYFRRYA  GLGPALYYLA**P**GLGAPAPYPD  YLAPGLGAPA**P**YPDPLEPKRE  APGLGAPAPY**P**DPLEPKREIC  GLGAPAPYPD**P**LEPKREICEL  APAPYPDPLE**P**KREICELNPD  EPKREICELN**P**DCDELADHIG  LRIMMIRLGA**P**QSLVLLTLLI  CQGQDARKLG**P**KGQKGEPGDI  KLGPKGQKGE**P**GDIKDIIGPK  EPGDIKDIIG**P**KGPPGPQGPA  DIKDIIGPKG**P**PGPQGPAGEQ  IKDIIGPKGP**P**GPQGPAGEQG  DIIGPKGPPG**P**QGPAGEQGPR  GPKGPPGPQG**P**AGEQGPRGDR  GPQGPAGEQG**P**RGDRGDKGER  DRGDKGERGA**P**GPRGRDGEPG  GDKGERGAPG**P**RGRDGEPGTP  APGPRGRDGE**P**GTPGNPGPPG  PRGRDGEPGT**P**GNPGPPGPPG  RDGEPGTPGN**P**GPPGPPGPPG  GEPGTPGNPG**P**PGPPGPPGPP  EPGTPGNPGP**P**GPPGPPGPPG  GTPGNPGPPG**P**PGPPGPPGLG  TPGNPGPPGP**P**GPPGPPGLGG  GNPGPPGPPG**P**PGPPGLGGGN  NPGPPGPPGP**P**GPPGLGGGNF  GPPGPPGPPG**P**PGLGGGNFAA  PPGPPGPPGP**P**GLGGGNFAAQ  GGAQMGVMQG**P**MGPMGPRGPP  QMGVMQGPMG**P**MGPRGPPGPA  VMQGPMGPMG**P**RGPPGPAGAP  GPMGPMGPRG**P**PGPAGAPGPQ  PMGPMGPRGP**P**GPAGAPGPQG  GPMGPRGPPG**P**AGAPGPQGFQ  PRGPPGPAGA**P**GPQGFQGNPG  GPPGPAGAPG**P**QGFQGNPGEP  APGPQGFQGN**P**GEPGEPGVSG  PQGFQGNPGE**P**GEPGVSGPIG  FQGNPGEPGE**P**GVSGPIGPRG  GEPGEPGVSG**P**IGPRGPPGPA  GEPGVSGPIG**P**RGPPGPAGKP  GVSGPIGPRG**P**PGPAGKPGDD  VSGPIGPRGP**P**GPAGKPGDDG  GPIGPRGPPG**P**AGKPGDDGEA  PRGPPGPAGK**P**GDDGEAGKPG  KPGDDGEAGK**P**GKAGERGLPG  KPGKAGERGL**P**GPQGARGFPG  GKAGERGLPG**P**QGARGFPGTP  LPGPQGARGF**P**GTPGLPGVKG  PQGARGFPGT**P**GLPGVKGHRG  ARGFPGTPGL**P**GVKGHRGYPG  LPGVKGHRGY**P**GLDGAKGEAG  LDGAKGEAGA**P**GVKGESGSPG  APGVKGESGS**P**GENGSPGPMG  ESGSPGENGS**P**GPMGPRGLPG  GSPGENGSPG**P**MGPRGLPGER  GENGSPGPMG**P**RGLPGERGRT  SPGPMGPRGL**P**GERGRTGPAG  GLPGERGRTG**P**AGAAGARGND  AAGARGNDGQ**P**GPAGPPGPVG  GARGNDGQPG**P**AGPPGPVGPA  GNDGQPGPAG**P**PGPVGPAGGP  NDGQPGPAGP**P**GPVGPAGGPG  GQPGPAGPPG**P**VGPAGGPGFL  GPAGPPGPVG**P**AGGPGFLGAP  PPGPVGPAGG**P**GFLGAPGAKG  PAGGPGFLGA**P**GAKGEAGPTG  GAPGAKGEAG**P**TGARGPEGAQ  GEAGPTGARG**P**EGAQGSRGEP  PEGAQGSRGE**P**GNPGSPGPAG  AQGSRGEPGN**P**GSPGPAGASG  SRGEPGNPGS**P**GPAGASGNPG  GEPGNPGSPG**P**AGASGNPGTD  SPGPAGASGN**P**GTDGIPGAKG  ASGNPGTDGI**P**GAKGSAGAPG  IPGAKGSAGA**P**GIAGAPGFPG  SAGAPGIAGA**P**GFPGPRGPPG  APGIAGAPGF**P**GPRGPPGPQG  GIAGAPGFPG**P**RGPPGPQGAT  GAPGFPGPRG**P**PGPQGATGPL  APGFPGPRGP**P**GPQGATGPLG  GFPGPRGPPG**P**QGATGPLGPK  GPPGPQGATG**P**LGPKGQTGEP  GPQGATGPLG**P**KGQTGEPGIA  PLGPKGQTGE**P**GIAGFKGEQG  GIAGFKGEQG**P**KGETGPAGPQ  GEQGPKGETG**P**AGPQGAPGPA  GPKGETGPAG**P**QGAPGPAGEE  ETGPAGPQGA**P**GPAGEEGKRG  GPAGPQGAPG**P**AGEEGKRGAR  EEGKRGARGE**P**GGAGPIGPPG  GARGEPGGAG**P**IGPPGERGAP  GEPGGAGPIG**P**PGERGAPGNR  EPGGAGPIGP**P**GERGAPGNRG  PIGPPGERGA**P**GNRGFPGQDG  ERGAPGNRGF**P**GQDGLAGPKG  GFPGQDGLAG**P**KGAPGERGPS  QDGLAGPKGA**P**GERGPSGLAG  GPKGAPGERG**P**SGLAGPKGAN  GERGPSGLAG**P**KGANGDPGRP  LAGPKGANGD**P**GRPGEPGLPG  PKGANGDPGR**P**GEPGLPGARG  ANGDPGRPGE**P**GLPGARGLTG  DPGRPGEPGL**P**GARGLTGRPG  LPGARGLTGR**P**GDAGPQGKVG  GLTGRPGDAG**P**QGKVGPSGAP  GDAGPQGKVG**P**SGAPGEDGRP  PQGKVGPSGA**P**GEDGRPGPPG  PSGAPGEDGR**P**GPPGPQGARG  GAPGEDGRPG**P**PGPQGARGQP  APGEDGRPGP**P**GPQGARGQPG  GEDGRPGPPG**P**QGARGQPGVM  PPGPQGARGQ**P**GVMGFPGPKG  ARGQPGVMGF**P**GPKGANGEPG  GQPGVMGFPG**P**KGANGEPGKA  FPGPKGANGE**P**GKAGEKGLAG  KAGEKGLAGA**P**GLRGLPGKDG  LAGAPGLRGL**P**GKDGETGAAG  GKDGETGAAG**P**PGPSGPAGER  KDGETGAAGP**P**GPSGPAGERG  GETGAAGPPG**P**SGPAGERGEQ  GAAGPPGPSG**P**AGERGEQGAP  PAGERGEQGA**P**GPSGFQGLPG  GERGEQGAPG**P**SGFQGLPGPP  APGPSGFQGL**P**GPPGPPGEGG  PSGFQGLPGP**P**GPPGEGGKQG  GFQGLPGPPG**P**PGEGGKQGDQ  FQGLPGPPGP**P**GEGGKQGDQG  EGGKQGDQGI**P**GEAGAPGLVG  DQGIPGEAGA**P**GLVGPRGERG  GEAGAPGLVG**P**RGERGFPGER  LVGPRGERGF**P**GERGSPGAQG  ERGFPGERGS**P**GAQGLQGPRG  GSPGAQGLQG**P**RGLPGTPGTD  AQGLQGPRGL**P**GTPGTDGPKG  LQGPRGLPGT**P**GTDGPKGAAG  GLPGTPGTDG**P**KGAAGPDGPP  GTDGPKGAAG**P**DGPPGAQGPP  GPKGAAGPDG**P**PGAQGPPGLQ  PKGAAGPDGP**P**GAQGPPGLQG  GPDGPPGAQG**P**PGLQGMPGER  PDGPPGAQGP**P**GLQGMPGERG  AQGPPGLQGM**P**GERGAAGIAG  GERGAAGIAG**P**KGDRGDVGEK  GDRGDVGEKG**P**EGAPGKDGGR  DVGEKGPEGA**P**GKDGGRGLTG  GKDGGRGLTG**P**IGPPGPAGAN  GGRGLTGPIG**P**PGPAGANGEK  GRGLTGPIGP**P**GPAGANGEKG  GLTGPIGPPG**P**AGANGEKGEV  GANGEKGEVG**P**PGPSGSTGAR  ANGEKGEVGP**P**GPSGSTGARG  GEKGEVGPPG**P**SGSTGARGAP  PSGSTGARGA**P**GERGETGPPG  GAPGERGETG**P**PGPAGFAGPP  APGERGETGP**P**GPAGFAGPPG  GERGETGPPG**P**AGFAGPPGAD  FAGPPGADGQ**P**GAKGDQGEAG  EAGQKGDAGA**P**GPQGPSGAPG  GQKGDAGAPG**P**QGPSGAPGPQ  GDAGAPGPQG**P**SGAPGPQGPT  APGPQGPSGA**P**GPQGPTGVTG  GPQGPSGAPG**P**QGPTGVTGPK  GPSGAPGPQG**P**TGVTGPKGAR  GPQGPTGVTG**P**KGARGAQGPP  GPKGARGAQG**P**PGATGFPGAA  PKGARGAQGP**P**GATGFPGAAG  AQGPPGATGF**P**GAAGRVGPPG  FPGAAGRVGP**P**GSNGNPGPAG  RVGPPGSNGN**P**GPAGPPGPAG  GPPGSNGNPG**P**AGPPGPAGKD  GSNGNPGPAG**P**PGPAGKDGPK  SNGNPGPAGP**P**GPAGKDGPKG  GNPGPAGPPG**P**AGKDGPKGAR  GPPGPAGKDG**P**KGARGDTGAP  PKGARGDTGA**P**GRAGDPGLQG  DTGAPGRAGD**P**GLQGPAGAPG  GRAGDPGLQG**P**AGAPGEKGEP  DPGLQGPAGA**P**GEKGEPGDDG  PAGAPGEKGE**P**GDDGPSGSDG  GEKGEPGDDG**P**SGSDGPPGPQ  GDDGPSGSDG**P**PGPQGLAGQR  DDGPSGSDGP**P**GPQGLAGQRG  GPSGSDGPPG**P**QGLAGQRGIV  LAGQRGIVGL**P**GQRGERGFPG  GERGFPGLPG**P**SGEPGKQGAP  FPGLPGPSGE**P**GKQGAPGASG  PSGEPGKQGA**P**GASGDRGPPG  GAPGASGDRG**P**PGPVGPPGLT  APGASGDRGP**P**GPVGPPGLTG  GASGDRGPPG**P**VGPPGLTGPA  GDRGPPGPVG**P**PGLTGPAGEP  DRGPPGPVGP**P**GLTGPAGEPG  GPVGPPGLTG**P**AGEPGREGSP  PPGLTGPAGE**P**GREGSPGADG  PAGEPGREGS**P**GADGPPGRDG  GREGSPGADG**P**PGRDGAAGVK  REGSPGADGP**P**GRDGAAGVKG  DRGETGALGA**P**GAPGPPGSPG  ETGALGAPGA**P**GPPGSPGPAG  GALGAPGAPG**P**PGSPGPAGPT  ALGAPGAPGP**P**GSPGPAGPTG  APGAPGPPGS**P**GPAGPTGKQG  GAPGPPGSPG**P**AGPTGKQGDR  GPPGSPGPAG**P**TGKQGDRGEA  GDRGEAGAQG**P**MGPSGPAGAR  GEAGAQGPMG**P**SGPAGARGIA  GAQGPMGPSG**P**AGARGIAGPQ  GPAGARGIAG**P**QGPRGDKGEA  GARGIAGPQG**P**RGDKGEAGEP  PRGDKGEAGE**P**GERGLKGHRG  HRGFTGLQGL**P**GPPGPSGDQG  FTGLQGLPGP**P**GPSGDQGTSG  GLQGLPGPPG**P**SGDQGTSGPA  GPSGDQGTSG**P**AGPSGPRGPP  GDQGTSGPAG**P**SGPRGPPGPV  GTSGPAGPSG**P**RGPPGPVGPS  GPAGPSGPRG**P**PGPVGPSGKD  PAGPSGPRGP**P**GPVGPSGKDG  GPSGPRGPPG**P**VGPSGKDGSN  GPRGPPGPVG**P**SGKDGSNGIP  PSGKDGSNGI**P**GPIGPPGPRG  GKDGSNGIPG**P**IGPPGPRGRS  SNGIPGPIGP**P**GPRGRSGETG  GIPGPIGPPG**P**RGRSGETGPA  GPRGRSGETG**P**AGPPGNPGPP  RSGETGPAGP**P**GNPGPPGPPG  ETGPAGPPGN**P**GPPGPPGPPG  PAGPPGNPGP**P**GPPGPPGPGI  GPPGNPGPPG**P**PGPPGPGIDM  PPGNPGPPGP**P**GPPGPGIDMS  NPGPPGPPGP**P**GPGIDMSAFA  GPPGPPGPPG**P**GIDMSAFAGL  FAGLGQREKG**P**DPLQYMRADE  GLGQREKGPD**P**LQYMRADEAD  LNNQIESIRS**P**DGSRKNPART  IRSPDGSRKN**P**ARTCQDLKLC  RTCQDLKLCH**P**EWKSGDYWID  EWKSGDYWID**P**NQGCTLDAMK  NMETGESCVY**P**NPATVPRKNW  ETGESCVYPN**P**ATVPRKNWWS  SCVYPNPATV**P**RKNWWSSKSK  HFSYGDGNLA**P**NTANVQMTFL  EYRSQKTSRL**P**IVDIAPMDIG  TSRLPIVDIA**P**MDIGGPDQEF  VDIAPMDIGG**P**DQEFGVDIGP  PDQEFGVDIG**P**VCFLLFCVPG  MMMANWAEAR**P**LLILIVLLGQ  QMYLNRDIWK**P**APCQICVCDN  YLNRDIWKPA**P**CQICVCDNGA  ECQDVLDCAD**P**VTPPGECCPV  DVLDCADPVT**P**PGECCPVCSQ  VLDCADPVTP**P**GECCPVCSQT  DPVTPPGECC**P**VCSQTPGGGN  GECCPVCSQT**P**GGGNTNFGRG  GRGRKGQKGE**P**GLVPVVTGIR  KGQKGEPGLV**P**VVTGIRGRPG  VPVVTGIRGR**P**GPAGPPGSQG  VVTGIRGRPG**P**AGPPGSQGPR  GIRGRPGPAG**P**PGSQGPRGER  IRGRPGPAGP**P**GSQGPRGERG  GPAGPPGSQG**P**RGERGPKGRP  GSQGPRGERG**P**KGRPGPRGPQ  PRGERGPKGR**P**GPRGPQGIDG  GERGPKGRPG**P**RGPQGIDGEP  GPKGRPGPRG**P**QGIDGEPGVP  PRGPQGIDGE**P**GVPGQPGAPG  PQGIDGEPGV**P**GQPGAPGPPG  IDGEPGVPGQ**P**GAPGPPGHPS  EPGVPGQPGA**P**GPPGHPSHPG  GVPGQPGAPG**P**PGHPSHPGPD  VPGQPGAPGP**P**GHPSHPGPDG  QPGAPGPPGH**P**SHPGPDGLSR  APGPPGHPSH**P**GPDGLSRPFS  GPPGHPSHPG**P**DGLSRPFSAQ  SHPGPDGLSR**P**FSAQMAGLDE  SGLGSQVGLM**P**GSVGPVGPRG  QVGLMPGSVG**P**VGPRGPQGLQ  LMPGSVGPVG**P**RGPQGLQGQQ  GSVGPVGPRG**P**QGLQGQQGGA  GLQGQQGGAG**P**TGPPGEPGDP  GQQGGAGPTG**P**PGEPGDPGPM  QQGGAGPTGP**P**GEPGDPGPMG  GAGPTGPPGE**P**GDPGPMGPIG  PTGPPGEPGD**P**GPMGPIGSRG  GPPGEPGDPG**P**MGPIGSRGPE  GEPGDPGPMG**P**IGSRGPEGPP  GPMGPIGSRG**P**EGPPGKPGED  GPIGSRGPEG**P**PGKPGEDGEP  PIGSRGPEGP**P**GKPGEDGEPG  SRGPEGPPGK**P**GEDGEPGRNG  PPGKPGEDGE**P**GRNGNPGEVG  EDGEPGRNGN**P**GEVGFAGSPG  NPGEVGFAGS**P**GARGFPGAPG  GHRGHKGLEG**P**KGEVGAPGSK  LEGPKGEVGA**P**GSKGEAGPTG  GAPGSKGEAG**P**TGPMGAMGPL  GSKGEAGPTG**P**MGAMGPLGPR  GPTGPMGAMG**P**LGPRGMPGER  GPMGAMGPLG**P**RGMPGERGRL  AMGPLGPRGM**P**GERGRLGPQG  GMPGERGRLG**P**QGAPGQRGAH  ERGRLGPQGA**P**GQRGAHGMPG  APGQRGAHGM**P**GKPGPMGPLG  QRGAHGMPGK**P**GPMGPLGIPG  GAHGMPGKPG**P**MGPLGIPGSS  GMPGKPGPMG**P**LGIPGSSGFP  KPGPMGPLGI**P**GSSGFPGNPG  PLGIPGSSGF**P**GNPGMKGEAG  IPGSSGFPGN**P**GMKGEAGPTG  GNPGMKGEAG**P**TGARGPEGPQ  GEAGPTGARG**P**EGPQGQRGET  GPTGARGPEG**P**QGQRGETGPP  GPQGQRGETG**P**PGPVGSPGLP  PQGQRGETGP**P**GPVGSPGLPG  GQRGETGPPG**P**VGSPGLPGAI  ETGPPGPVGS**P**GLPGAIGTDG  PPGPVGSPGL**P**GAIGTDGTPG  LPGAIGTDGT**P**GAKGPTGSPG  GTDGTPGAKG**P**TGSPGTSGPP  TPGAKGPTGS**P**GTSGPPGSAG  GPTGSPGTSG**P**PGSAGPPGSP  PTGSPGTSGP**P**GSAGPPGSPG  GTSGPPGSAG**P**PGSPGPQGST  TSGPPGSAGP**P**GSPGPQGSTG  PPGSAGPPGS**P**GPQGSTGPQG  GSAGPPGSPG**P**QGSTGPQGIR  GSPGPQGSTG**P**QGIRGQPGDP  STGPQGIRGQ**P**GDPGVPGFKG  PQGIRGQPGD**P**GVPGFKGEAG  IRGQPGDPGV**P**GFKGEAGPKG  GVPGFKGEAG**P**KGEPGPHGIQ  FKGEAGPKGE**P**GPHGIQGPIG  GEAGPKGEPG**P**HGIQGPIGPP  GEPGPHGIQG**P**IGPPGEEGKR  GPHGIQGPIG**P**PGEEGKRGPR  PHGIQGPIGP**P**GEEGKRGPRG  GPPGEEGKRG**P**RGDPGTVGPP  EEGKRGPRGD**P**GTVGPPGPVG  GPRGDPGTVG**P**PGPVGERGAP  PRGDPGTVGP**P**GPVGERGAPG  GDPGTVGPPG**P**VGERGAPGNR  PPGPVGERGA**P**GNRGFPGSDG  ERGAPGNRGF**P**GSDGLPGPKG  NRGFPGSDGL**P**GPKGAQGERG  GFPGSDGLPG**P**KGAQGERGPV  GPKGAQGERG**P**VGSSGPKGSQ  GERGPVGSSG**P**KGSQGDPGRP  SSGPKGSQGD**P**GRPGEPGLPG  PKGSQGDPGR**P**GEPGLPGARG  SQGDPGRPGE**P**GLPGARGLTG  DPGRPGEPGL**P**GARGLTGNPG  LPGARGLTGN**P**GVQGPEGKLG  GLTGNPGVQG**P**EGKLGPLGAP  GVQGPEGKLG**P**LGAPGEDGRP  PEGKLGPLGA**P**GEDGRPGPPG  PLGAPGEDGR**P**GPPGSIGIRG  GAPGEDGRPG**P**PGSIGIRGQP  APGEDGRPGP**P**GSIGIRGQPG  GQPGSMGLPG**P**KGSSGDPGKP  LPGPKGSSGD**P**GKPGEAGNAG  PKGSSGDPGK**P**GEAGNAGVPG  KPGEAGNAGV**P**GQRGAPGKDG  NAGVPGQRGA**P**GKDGEVGPSG  GAPGKDGEVG**P**SGPVGPPGLA  GKDGEVGPSG**P**VGPPGLAGER  GEVGPSGPVG**P**PGLAGERGEQ  EVGPSGPVGP**P**GLAGERGEQG  GLAGERGEQG**P**PGPTGFQGLP  LAGERGEQGP**P**GPTGFQGLPG  GERGEQGPPG**P**TGFQGLPGPP  PPGPTGFQGL**P**GPPGPPGEGG  GPTGFQGLPG**P**PGPPGEGGKP  PTGFQGLPGP**P**GPPGEGGKPG  GFQGLPGPPG**P**PGEGGKPGDQ  FQGLPGPPGP**P**GEGGKPGDQG  PPGPPGEGGK**P**GDQGVPGDPG  EGGKPGDQGV**P**GDPGAVGPLG  KPGDQGVPGD**P**GAVGPLGPRG  GVPGDPGAVG**P**LGPRGERGNP  GDPGAVGPLG**P**RGERGNPGER  PLGPRGERGN**P**GERGEPGITG  ERGNPGERGE**P**GITGLPGEKG  ERGEPGITGL**P**GEKGMAGGHG  GEKGMAGGHG**P**DGPKGSPGPS  GMAGGHGPDG**P**KGSPGPSGTP  GHGPDGPKGS**P**GPSGTPGDTG  GPDGPKGSPG**P**SGTPGDTGPP  PKGSPGPSGT**P**GDTGPPGLQG  GPSGTPGDTG**P**PGLQGMPGER  PSGTPGDTGP**P**GLQGMPGERG  DTGPPGLQGM**P**GERGIAGTPG  MPGERGIAGT**P**GPKGDRGGIG  GERGIAGTPG**P**KGDRGGIGEK  TAGNDGARGL**P**GPLGPPGPAG  GNDGARGLPG**P**LGPPGPAGPT  GARGLPGPLG**P**PGPAGPTGEK  ARGLPGPLGP**P**GPAGPTGEKG  GLPGPLGPPG**P**AGPTGEKGEP  GPLGPPGPAG**P**TGEKGEPGPR  PAGPTGEKGE**P**GPRGLVGPPG  GPTGEKGEPG**P**RGLVGPPGSR  GEPGPRGLVG**P**PGSRGNPGSR  EPGPRGLVGP**P**GSRGNPGSRG  LVGPPGSRGN**P**GSRGENGPTG  GNPGSRGENG**P**TGAVGFAGPQ  GPTGAVGFAG**P**QGPDGQPGVK  GAVGFAGPQG**P**DGQPGVKGEP  FAGPQGPDGQ**P**GVKGEPGEPG  PDGQPGVKGE**P**GEPGQKGDAG  QPGVKGEPGE**P**GQKGDAGSPG  EPGQKGDAGS**P**GPQGLAGSPG  GQKGDAGSPG**P**QGLAGSPGPH  SPGPQGLAGS**P**GPHGPNGVPG  GPQGLAGSPG**P**HGPNGVPGLK  GLAGSPGPHG**P**NGVPGLKGGR  SPGPHGPNGV**P**GLKGGRGTQG  GLKGGRGTQG**P**PGATGFPGSA  LKGGRGTQGP**P**GATGFPGSAG  TQGPPGATGF**P**GSAGRVGPPG  FPGSAGRVGP**P**GPAGAPGPAG  GSAGRVGPPG**P**AGAPGPAGPL  RVGPPGPAGA**P**GPAGPLGEPG  GPPGPAGAPG**P**AGPLGEPGKE  GPAGAPGPAG**P**LGEPGKEGPP  APGPAGPLGE**P**GKEGPPGLRG  GPLGEPGKEG**P**PGLRGDPGSH  PLGEPGKEGP**P**GLRGDPGSHG  KEGPPGLRGD**P**GSHGRVGDRG  GSHGRVGDRG**P**AGPPGGPGDK  GRVGDRGPAG**P**PGGPGDKGDP  RVGDRGPAGP**P**GGPGDKGDPG  DRGPAGPPGG**P**GDKGDPGEDG  PPGGPGDKGD**P**GEDGQPGPDG  DKGDPGEDGQ**P**GPDGPPGPAG  GDPGEDGQPG**P**DGPPGPAGTT  GEDGQPGPDG**P**PGPAGTTGQR  EDGQPGPDGP**P**GPAGTTGQRG  GQPGPDGPPG**P**AGTTGQRGIV  TTGQRGIVGM**P**GQRGERGMPG  MPGQRGERGM**P**GLPGPAGTPG  QRGERGMPGL**P**GPAGTPGKVG  GERGMPGLPG**P**AGTPGKVGPT  MPGLPGPAGT**P**GKVGPTGATG  GPAGTPGKVG**P**TGATGDKGPP  GPTGATGDKG**P**PGPVGPPGSN  PTGATGDKGP**P**GPVGPPGSNG  GATGDKGPPG**P**VGPPGSNGPV  GDKGPPGPVG**P**PGSNGPVGEP  DKGPPGPVGP**P**GSNGPVGEPG  GPVGPPGSNG**P**VGEPGPEGPA  PPGSNGPVGE**P**GPEGPAGNDG  GSNGPVGEPG**P**EGPAGNDGTP  GPVGEPGPEG**P**AGNDGTPGRD  PEGPAGNDGT**P**GRDGAVGERG  AVGERGDRGD**P**GPAGLPGSQG  GERGDRGDPG**P**AGLPGSQGAP  DRGDPGPAGL**P**GSQGAPGTPG  PAGLPGSQGA**P**GTPGPVGAPG  LPGSQGAPGT**P**GPVGAPGDAG  GSQGAPGTPG**P**VGAPGDAGQR  APGTPGPVGA**P**GDAGQRGDPG  APGDAGQRGD**P**GSRGPIGPPG  GQRGDPGSRG**P**IGPPGRAGKR  GDPGSRGPIG**P**PGRAGKRGLP  DPGSRGPIGP**P**GRAGKRGLPG  PPGRAGKRGL**P**GPQGPRGDKG  GRAGKRGLPG**P**QGPRGDKGDH  GKRGLPGPQG**P**RGDKGDHGDR  HRGFTGLQGL**P**GPPGPNGEQG  FTGLQGLPGP**P**GPNGEQGSAG  GLQGLPGPPG**P**NGEQGSAGIP  PNGEQGSAGI**P**GPFGPRGPPG  GEQGSAGIPG**P**FGPRGPPGPV  GSAGIPGPFG**P**RGPPGPVGPS  GIPGPFGPRG**P**PGPVGPSGKE  IPGPFGPRGP**P**GPVGPSGKEG  GPFGPRGPPG**P**VGPSGKEGNP  GPRGPPGPVG**P**SGKEGNPGPL  PVGPSGKEGN**P**GPLGPIGPPG  GPSGKEGNPG**P**LGPIGPPGVR  GKEGNPGPLG**P**IGPPGVRGSV  GNPGPLGPIG**P**PGVRGSVGEA  NPGPLGPIGP**P**GVRGSVGEAG  GVRGSVGEAG**P**EGPPGEPGPP  GSVGEAGPEG**P**PGEPGPPGPP  SVGEAGPEGP**P**GEPGPPGPPG  EAGPEGPPGE**P**GPPGPPGPPG  GPEGPPGEPG**P**PGPPGPPGHL  PEGPPGEPGP**P**GPPGPPGHLT  GPPGEPGPPG**P**PGPPGHLTAA  PPGEPGPPGP**P**GPPGHLTAAL  GEPGPPGPPG**P**PGHLTAALGD  EPGPPGPPGP**P**GHLTAALGDI  DIMGHYDESM**P**DPLPEFTEDQ  MGHYDESMPD**P**LPEFTEDQAA  HYDESMPDPL**P**EFTEDQAAPD  LPEFTEDQAA**P**DDKNKTDPGV  AAPDDKNKTD**P**GVHATLKSLS  LSSQIETMRS**P**DGSKKHPART  MRSPDGSKKH**P**ARTCDDLKLC  AKQSGEYWID**P**NQGSVEDAIK  ETGETCISAN**P**SSVPRKTWWA  TCISANPSSV**P**RKTWWASKSP  PRKTWWASKS**P**DNKPVWYGLD  WWASKSPDNK**P**VWYGLDMNRG  SQFAYGDHQS**P**NTAITQMTFL  EYRTQNVARL**P**IIDLAPVDVG  VARLPIIDLA**P**VDVGGTDQEF  TDQEFGVEIG**P**VCFVVFCVPG  GNGPQGMMGQ**P**GNGSAFLLAP  PGNGSAFLLA**P**NGSHAPDHDV  FLLAPNGSHA**P**DHDVTQERDE  ADLVMGLAVV**P**FGAAHILMKM  AVDRYFAITS**P**FKYQSLLTKN  WIVSGLTSFL**P**IQMHWYRATH  IASSIVSFYV**P**LVIMVFVYSR  IMGTFTLCWL**P**FFIVNIVHVI  WIGYVNSGFN**P**LIYCRSPDFR  GFNPLIYCRS**P**DFRIAFQELL  CGSQAGGARC**P**SGLCCSKFGW  WCGNTNDYCG**P**GNCQSQCPGG  CGPGNCQSQC**P**GGPTPTPPTP  NAFINAARSF**P**GFGTSGDTTA  HETTGGWATA**P**DGPYAWGYCW  TGGWATAPDG**P**YAWGYCWLRE  GYCWLREQGS**P**GDYCTPSGQW  EQGSPGDYCT**P**SGQWPCAPGR  GDYCTPSGQW**P**CAPGRKYFGR  CTPSGQWPCA**P**GRKYFGRGPI  APGRKYFGRG**P**IQISHNYNYG  IQISHNYNYG**P**CGRAIGVDLL  RAIGVDLLNN**P**DLVATDPVIS  LNNPDLVATD**P**VISFKSALWF  FKSALWFWMT**P**QSPKPSCHDV  ALWFWMTPQS**P**KPSCHDVIIG  WFWMTPQSPK**P**SCHDVIIGRW  CHDVIIGRWQ**P**SAGDRAANRL  SAGDRAANRL**P**GFGVITNIIN  RRYCSILGVS**P**GDNLDCGNQR  GRDGVKGDPG**P**PGPMGPPGGM  GVKGDPGPPG**P**MGPPGGMPGL  GDPGPPGPMG**P**PGGMPGLPGR  LPGRDGLPGA**P**GAPGERGDKG  RDGLPGAPGA**P**GERGDKGEPG  APGERGDKGE**P**GERGLPGFPA  DKGEPGERGL**P**GFPAYLDEEL  EPGERGLPGF**P**AYLDEELQTE  CTRAGGNIAV**P**RTPEENEAIA  AGGNIAVPRT**P**EENEAIASIA  VYLGMIEDQT**P**GDFHYLDGAS  GASVNYTNWY**P**GEPRGQGKEK  VNYTNWYPGE**P**RGQGKEKCVE  TLTEGAQSSC**P**VIACSSPGLN  SSCPVIACSS**P**GLNGFPGKDG  PGQGLRGLQG**P**PGKVGPAGPP  GLQGPPGKVG**P**AGPPGNPGSK  GPPGKVGPAG**P**PGNPGSKGAT  GNPGSKGATG**P**KGDRGESVEF  KYFMSSVRRM**P**LNRAKALCSE  CSELQGTVAT**P**RNAEENRAIQ  VRYTNWNEGE**P**NNVGSGENCV  LLTNGKWNDV**P**CSDSFLVVCE  ILLTICLLLF**P**LTAVPLDGDQ  CLLLFPLTAV**P**LDGDQPADRP  LTAVPLDGDQ**P**ADRPAERMQD  PLDGDQPADR**P**AERMQDDISS  MQDDISSEHH**P**FFDPVKRCCR  ISSEHHPFFD**P**VKRCCRLSCG  CLLLFALTAV**P**LDGDQPADRP  MQDDISSERH**P**MFDAVRDCCP  FDAVRDCCPL**P**ACPFGCNPCC  VRDCCPLPAC**P**FGCNPCCGGC  EMMLLVLLIF**P**LSYFIAAGGQ  CCCGVPNAAC**P**PCVCNKTCGG  VVLATTVVSI**P**SDRASDGRNA  APGFPGARGA**P**GPQGPSGAPG  GFPGARGAPG**P**QGPSGAPGPK  GARGAPGPQG**P**SGAPGPKWQS  GPQGPSGAPG**P**KWQSRLHKKQ  HMYVQWIYYY**P**ETITHDMNWW  CVVNHSIMCL**P**GLVVYNRYHF  HFGVFQWLSY**P**WDERFSINMS  CGYYILLERI**P**DDLWDQTQVY  YGCQCQHSMM**P**SGACHNQDCL  NQDCLAMHYE**P**HDLSTYRHQP  PHDLSTYRHQ**P**VYMPPRAWYY  STYRHQPVYM**P**PRAWYYIRRD  TYRHQPVYMP**P**RAWYYIRRDE  SHMMVIFECN**P**KGMTPALGVQ  IFECNPKGMT**P**ALGVQGPPGP  GMTPALGVQG**P**PGPQGPRLIY  PALGVQGPPG**P**QGPRLIYTVL  GVQGPPGPQG**P**RLIYTVLSRR  LQHMKMIFGN**P**RQERYDPDRQ  FGNPRQERYD**P**DRQYRCLPKT  YDPDRQYRCL**P**KTHHIVKHMI  QTVLDFGIHI**P**PYWTGKNGML  TVLDFGIHIP**P**YWTGKNGMLG  GKNGMLGYYD**P**DSRQQTHRSH  DSRQQTHRSH**P**PAHHCGCASA  SRQQTHRSHP**P**AHHCGCASAY  NGYFVGRYYL**P**GSAGPPGATG  GRYYLPGSAG**P**PGATGFPGAA  QWHKKHLLHR**P**CTTHSFLKSG  HSFLKSGYCM**P**DFIKEFITGV  CVDLMKMSQG**P**YPETTNMHNN  DLMKMSQGPY**P**ETTNMHNNIS  DEKSAGGISV**P**GPMGPSGPRG  KSAGGISVPG**P**MGPSGPRGLP  GGISVPGPMG**P**SGPRGLPGPP  GAVGAKGEAG**P**QGARGSEGPQ  GPQGARGSEG**P**QGVRGEPGPP  GAPGFPGARG**P**AGPQGPSGAP  GFPGARGPAG**P**QGPSGAPGPK  GARGPAGPQG**P**SGAPGPKGNS  APGPKGNSGE**P**GAPGSKGDAG  PKGNSGEPGA**P**GSKGDAGAKG  GEPGPIGIQG**P**PGPAGEEGKR  GPIGIQGPPG**P**AGEEGKRGAR  GKRGARGEPG**P**TGLPGPPGER  GEPGPTGLPG**P**PGERGGPGSR  GFPGADGVAG**P**KGPAGERGSP  GADGVAGPKG**P**AGERGSPGPA  PKGPAGERGS**P**GPAGPKGSPG  GPAGERGSPG**P**AGPKGSPGEA  GERGSPGPAG**P**KGSPGEAGRP  EPGKAGERGV**P**GPPGAVGAAG  GKAGERGVPG**P**PGAVGAAGKD  KAGERGVPGP**P**GAVGAAGKDG  GFPGERGVQG**P**PGPAGPRGSN  GERGVQGPPG**P**AGPRGSNGAP  GVQGPPGPAG**P**RGSNGAPGND  PAGPRGSNGA**P**GNDGAKGDAG  GPKGDRGDAG**P**KGADGSPGKD  DAGPKGADGS**P**GKDGPRGLTG  GADGSPGKDG**P**RGLTGPIGPP  GKDGPRGLTG**P**IGPPGPAGAP  GPRGLTGPIG**P**PGPAGAPGDK  DAGAKGDAGP**P**GPAGPTGAPG  GAKGDAGPPG**P**AGPTGAPGPI  GDAGPPGPAG**P**TGAPGPIGNV  PPGPAGPTGA**P**GPIGNVGAPG  GPAGPTGAPG**P**IGNVGAPGAK  APGPIGNVGA**P**GAKGARGSAG  GAKGARGSAG**P**PGATGFPGAA  GPSGNAGPPG**P**PGPAGKEGGK  PSGNAGPPGP**P**GPAGKEGGKG  GNAGPPGPPG**P**AGKEGGKGPR  GPAGKEGGKG**P**RGETGPAGRP  GGKGPRGETG**P**AGRPGEVGPP  GRPGEVGPPG**P**PGPAGEKGSP  GEVGPPGPPG**P**AGEKGSPGAD  PPGPAGEKGS**P**GADGPAGAPG  GEKGSPGADG**P**AGAPGTPGPQ  SPGADGPAGA**P**GTPGPQGIGG  ADGPAGAPGT**P**GPQGIGGQRG  GPAGAPGTPG**P**QGIGGQRGVV  IGGQRGVVGL**P**GQRGERGFPG  FPGLPGPSGE**P**GKQGPSGSSG  GPSGEPGKQG**P**SGSSGERGPP  GPSGSSGERG**P**PGPAGPPGLA  PSGSSGERGP**P**GPAGPPGLAG  GSSGERGPPG**P**AGPPGLAGPP  GERGPPGPAG**P**PGLAGPPGES  ERGPPGPAGP**P**GLAGPPGESG  GPAGPPGLAG**P**PGESGREGAP  PAGPPGLAGP**P**GESGREGAPG  GSPGRDGSPG**P**KGDRGETGPS  GPKGDRGETG**P**SGPPGAPGAP  GDRGETGPSG**P**PGAPGAPGAP  DRGETGPSGP**P**GAPGAPGAPG  GKSGDRGETG**P**AGPAGPAGPA  GDRGETGPAG**P**AGPAGPAGVR  GETGPAGPAG**P**AGPAGVRGPA  GPAGPAGPAG**P**AGVRGPAGPQ  GPAGPAGVRG**P**AGPQGPRGDK  GPAGVRGPAG**P**QGPRGDKGET  GVRGPAGPQG**P**RGDKGETGEQ  GPSGASGPAG**P**RGPPGSAGAP  GASGPAGPRG**P**PGSAGAPGKD  ASGPAGPRGP**P**GSAGAPGKDG  PRGPPGSAGA**P**GKDGLNGLPG  GKDGLNGLPG**P**PGPPGPRGRT  GLNGLPGPPG**P**PGPRGRTGDA  GLPGPPGPPG**P**RGRTGDAGPV  GPRGRTGDAG**P**VGPPGPPGPP  GRTGDAGPVG**P**PGPPGPPGPP  RTGDAGPVGP**P**GPPGPPGPPG  GDAGPVGPPG**P**PGPPGPPGPP  DAGPVGPPGP**P**GPPGPPGPPS  GPVGPPGPPG**P**PGPPGPPSGA  PVGPPGPPGP**P**GPPGPPSGAF  GPPGPPGPPG**P**PGPPSGAFDF  PPGPPGPPGP**P**GPPSGAFDFS  GPPGPPGPPG**P**PSGAFDFSFL  PPGPPGPPGP**P**SGAFDFSFLP  PSGAFDFSFL**P**QPPQEKAHDG  GAFDFSFLPQ**P**PQEKAHDGGR  AFDFSFLPQP**P**QEKAHDGGRY  SQQSPGCCWN**P**ACVKNRCCRN  CASPACCCCA**P**SACRLGCRPC  CPAGIIIIGA**P**CRRCYHSDGK  YTTYGIYDAK**P**PFSCAGLRGG  CAGLRGGCVL**P**PNLRPKFKEE  DSRYGLKDLF**P**KARHEMKNPE  FPKARHEMKN**P**EASKLNKRDE  DSRYGLKNLF**P**KARHEMKNPE  SKLNKRDECY**P**PGTFCGIKPG  PGLCCSERCF**P**FVCLSLEFFE  SPFSKICMNY**P**QNNQPYNMCI  SMMSTLGILL**P**IALLLPLANP  GILLPIALLL**P**LANPAENGDG  PIALLLPLAN**P**AENGDGQAMP  PAENGDGQAM**P**RTRNLRSLSF  ETDTGPCCCK**P**NFTCQISNSG  AVVLLVLLIL**P**LSYFDVAGGQ  DRRGNGLARY**P**QRGGRDNEAE  GKCCLTRMCG**P**MCCARSGCTC  SRHAMMAHRS**P**ALCLLLLHAA  ACLCLAQLRG**P**PGEPGPRGPP  CLCLAQLRGP**P**GEPGPRGPPG  LAQLRGPPGE**P**GPRGPPGPPG  QLRGPPGEPG**P**RGPPGPPGVP  GPPGEPGPRG**P**PGPPGVPGAD  PPGEPGPRGP**P**GPPGVPGADG  GEPGPRGPPG**P**PGVPGADGID  EPGPRGPPGP**P**GVPGADGIDG  PRGPPGPPGV**P**GADGIDGDKG  ADGIDGDKGS**P**GAPGSPGAKG  IDGDKGSPGA**P**GSPGAKGEPG  DKGSPGAPGS**P**GAKGEPGAPG  APGSPGAKGE**P**GAPGPDGPPG  SPGAKGEPGA**P**GPDGPPGKPG  GAKGEPGAPG**P**DGPPGKPGLD  GEPGAPGPDG**P**PGKPGLDGLT  EPGAPGPDGP**P**GKPGLDGLTG  APGPDGPPGK**P**GLDGLTGAKG  GLTGAKGSRG**P**WGGQGLKGQP  PWGGQGLKGQ**P**GLPGPPGLPG  GQGLKGQPGL**P**GPPGLPGPSL  GLKGQPGLPG**P**PGLPGPSLPG  LKGQPGLPGP**P**GLPGPSLPGP  QPGLPGPPGL**P**GPSLPGPPGL  GLPGPPGLPG**P**SLPGPPGLPG  GPPGLPGPSL**P**GPPGLPGQVG  PGLPGPSLPG**P**PGLPGQVGLP  GLPGPSLPGP**P**GLPGQVGLPG  GPSLPGPPGL**P**GQVGLPGEIG  PPGLPGQVGL**P**GEIGVPGPKG  QVGLPGEIGV**P**GPKGDPGPDG  GLPGEIGVPG**P**KGDPGPDGPR  EIGVPGPKGD**P**GPDGPRGPPG  GVPGPKGDPG**P**DGPRGPPGPP  GPKGDPGPDG**P**RGPPGPPGKP  GDPGPDGPRG**P**PGPPGKPGPP  DPGPDGPRGP**P**GPPGKPGPPG  GPDGPRGPPG**P**PGKPGPPGHI  PDGPRGPPGP**P**GKPGPPGHIQ  PRGPPGPPGK**P**GPPGHIQGVE  GPPGPPGKPG**P**PGHIQGVEGS  GVEGSADFLC**P**TNCPPGPKGP  SADFLCPTNC**P**PGPKGPQGLQ  FLCPTNCPPG**P**KGPQGLQGLK  PTNCPPGPKG**P**QGLQGLKGHR  LQGLKGHRGR**P**GALGEPGQQG  HRGRPGALGE**P**GQQGKQGPKG  GEPGQQGKQG**P**KGDVGVSGEQ  DVGVSGEQGV**P**GPPGPQGQRG  GVSGEQGVPG**P**PGPQGQRGYP  VSGEQGVPGP**P**GPQGQRGYPG  GEQGVPGPPG**P**QGQRGYPGMA  PPGPQGQRGY**P**GMAGPKGETG  GQRGYPGMAG**P**KGETGPAGYK  GMAGPKGETG**P**AGYKGMVGTI  MVGTIGAAGR**P**GREGPKGPPG  GAAGRPGREG**P**KGPPGDPGEK  GRPGREGPKG**P**PGDPGEKGEL  RPGREGPKGP**P**GDPGEKGELG  REGPKGPPGD**P**GEKGELGGRG  GELGGRGIRG**P**QGDIGPKGDM  GIRGPQGDIG**P**KGDMGLPGID  DIGPKGDMGL**P**GIDGKDGTPG  LPGIDGKDGT**P**GIPGVKGTAG  IDGKDGTPGI**P**GVKGTAGQPG  IPGVKGTAGQ**P**GRPGPPGHRG  VKGTAGQPGR**P**GPPGHRGQAG  GTAGQPGRPG**P**PGHRGQAGLP  TAGQPGRPGP**P**GHRGQAGLPG  PPGHRGQAGL**P**GQPGSKGGPG  HRGQAGLPGQ**P**GSKGGPGDKG  LPGQPGSKGG**P**GDKGEVGARG  ARGQQGITGT**P**GLDGEPGPPG  ITGTPGLDGE**P**GPPGDAGTAG  GTPGLDGEPG**P**PGDAGTAGVP  TPGLDGEPGP**P**GDAGTAGVPG  PPGDAGTAGV**P**GLKGDRGERG  GLKGDRGERG**P**VGAPGEAGQS  DRGERGPVGA**P**GEAGQSGPKG  GAPGEAGQSG**P**KGEQGPPGIP  GQSGPKGEQG**P**PGIPGPQGLP  QSGPKGEQGP**P**GIPGPQGLPG  PKGEQGPPGI**P**GPQGLPGVKG  GEQGPPGIPG**P**QGLPGVKGDK  PPGIPGPQGL**P**GVKGDKGSPG  LPGVKGDKGS**P**GKTGPKGSTG  GDKGSPGKTG**P**KGSTGDPGVH  KTGPKGSTGD**P**GVHGLAGVKG  VKGEKGESGE**P**GPKGQQGIQG  GEKGESGEPG**P**KGQQGIQGEL  QQGIQGELGF**P**GPSGDAGSPG  GIQGELGFPG**P**SGDAGSPGVR  FPGPSGDAGS**P**GVRGYPGPPG  DAGSPGVRGY**P**GPPGPRGLLG  GSPGVRGYPG**P**PGPRGLLGER  SPGVRGYPGP**P**GPRGLLGERG  GVRGYPGPPG**P**RGLLGERGVP  PRGLLGERGV**P**GMPGQRGVAG  LLGERGVPGM**P**GQRGVAGRDA  AALGGVGAMG**P**PGPPGPPGPP  ALGGVGAMGP**P**GPPGPPGPPG  GGVGAMGPPG**P**PGPPGPPGEQ  GVGAMGPPGP**P**GPPGPPGEQG  GAMGPPGPPG**P**PGPPGEQGLH  AMGPPGPPGP**P**GPPGEQGLHG  GPPGPPGPPG**P**PGEQGLHGPM  PPGPPGPPGP**P**GEQGLHGPMG  GPPGEQGLHG**P**MGPRGVPGLL  GEQGLHGPMG**P**RGVPGLLGAA  LHGPMGPRGV**P**GLLGAAGQIG  GAAGQIGNIG**P**KGKRGEKGER  GERGDTGRGH**P**GMPGPPGIPG  GDTGRGHPGM**P**GPPGIPGLPG  TGRGHPGMPG**P**PGIPGLPGIP  GRGHPGMPGP**P**GIPGLPGIPG  HPGMPGPPGI**P**GLPGIPGHAL  MPGPPGIPGL**P**GIPGHALAGK  PPGIPGLPGI**P**GHALAGKDGE  ALAGKDGERG**P**PGVPGDAGRP  LAGKDGERGP**P**GVPGDAGRPG  KDGERGPPGV**P**GDAGRPGSPG  PPGVPGDAGR**P**GSPGPAGLPG  VPGDAGRPGS**P**GPAGLPGFCE  GDAGRPGSPG**P**AGLPGFCEPA  RPGSPGPAGL**P**GFCEPAACLG  GPAGLPGFCE**P**AACLGALPTP  CEPAACLGAL**P**TPRHGGHRPT  PAACLGALPT**P**RHGGHRPTPL  KRMMRKHRHL**P**LVAVFCLFLS  AVFCLFLSGF**P**TTHAQQQQAD  HFALVQFNGN**P**HTEFLLNTYR  AAGSRAGDGV**P**QVIVVLTDGH  DGHSKDGLAL**P**SAELKSADVN  EGALKEIASE**P**LNMHMFNLEN  LVSCVHSSVS**P**ERAGDTETLK  DFLVNLLEKL**P**IGTQQIRVGV  RVGVVQFSDE**P**RTMFSLDTYS  AGGSRVEEGV**P**QVLVLISAGP  PQVLVLISAG**P**SSDEIRYGVV  ATDDNLVFTV**P**EFRSFGDLQE  SFGDLQEKLL**P**YIVGVAQRHI  GVAQRHIVLK**P**PTIVTQVIEV  VAQRHIVLKP**P**TIVTQVIEVN  AVAQYADTVR**P**EFYFNTHPTK  VRPEFYFNTH**P**TKREVITAVR  EVITAVRKMK**P**LDGSALYTGS  SAGYRAAEGI**P**KLLVLITGGK  GGKSLDEISQ**P**AQELKRSSIM  IAFDSSLVFI**P**AEFRAAPLQG  VFIPAEFRAA**P**LQGMLPGLLA  FRAAPLQGML**P**GLLAPLRTLS  LQGMLPGLLA**P**LRTLSGTPEV  LAPLRTLSGT**P**EVHSNKRDII  GSANVGKTNF**P**YVRDFVMNLV  RVGLVQFSDT**P**VTEFSLNTYQ  AGGSRIREHV**P**QLLLLLTAGQ  KAELEQIAFN**P**SLVYLMDDFS  VYLMDDFSSL**P**ALPQQLIQPL  MDDFSSLPAL**P**QQLIQPLTTY  LPALPQQLIQ**P**LTTYVSGGVE  TYVSGGVEEV**P**LAQPESKRDI  GGVEEVPLAQ**P**ESKRDILFLF  DGSANLVGQF**P**VVRDFLYKII  YKIIDELNVK**P**EGTRIAVAQY  ESRFDEHQSK**P**EILNLVKRMK  AGRSSDRVDG**P**ASNLKQSGVV  ASNLKQSGVV**P**FIFQAKNADP  PFIFQAKNAD**P**AELEQIVLSP  PAELEQIVLS**P**AFILAAESLP  PAFILAAESL**P**KIGDLHPQIV  ESLPKIGDLH**P**QIVNLLKSVH  NLLKSVHNGA**P**APVSGEKDVV  LKSVHNGAPA**P**VSGEKDVVFL  DGSEGVRSGF**P**LLKEFVQRVV  AVVQYSDRTR**P**EFYLNSYMNK  AVRQLTLLGG**P**TPNTGAALEF  RQLTLLGGPT**P**NTGAALEFVL  SAGSRITEGV**P**QLLIVLTADR  ADRSGDDVRN**P**SVVVKRGGAV  SVVVKRGGAV**P**IGIGIGNADI  ITEMQTISFI**P**DFAVAIPTFR  SFIPDFAVAI**P**TFRQLGTVQQ  LTREELSRLQ**P**VLQPLPSPGV  ELSRLQPVLQ**P**LPSPGVGGKR  SRLQPVLQPL**P**SPGVGGKRDV  LQPVLQPLPS**P**GVGGKRDVVF  FLIDGSQSAG**P**EFQYVRTLIE  RVAVIQFSDD**P**KVEFLLNAHS  EVQNAVQRLR**P**KGGRQINVGN  EYVSRNIFKR**P**LGSRIEEGVP  PLGSRIEEGV**P**QFLVLISSGK  SGKSDDEVDD**P**AVELKQFGVA  AVELKQFGVA**P**FTIARNADQE  QEELVKISLS**P**EYVFSVSTFR  VFSVSTFREL**P**SLEQKLLTPI  LPSLEQKLLT**P**ITTLTSEQIQ  IQKLLASTRY**P**PPAVESDAAD  QKLLASTRYP**P**PAVESDAADI  KLLASTRYPP**P**AVESDAADIV  FLIDSSEGVR**P**DGFAHIRDFV  SRIVRRLNIG**P**SKVRVGVVQF  GVVQFSNDVF**P**EFYLKTYRSQ  FYLKTYRSQA**P**VLDAIRRLRL  IRRLRLRGGS**P**LNTGKALEFV  SAGSRIEDGV**P**QHLVLVLGGK  RTELQTITND**P**RLVFTVREFR  VFTVREFREL**P**NIEERIMNSF  IEERIMNSFG**P**SAATPAPPGV  MNSFGPSAAT**P**APPGVDTPPP  SFGPSAATPA**P**PGVDTPPPSR  FGPSAATPAP**P**GVDTPPPSRP  ATPAPPGVDT**P**PPSRPEKKKA  TPAPPGVDTP**P**PSRPEKKKAD  PAPPGVDTPP**P**SRPEKKKADI  PGVDTPPPSR**P**EKKKADIVFL  QVGLVQYNSD**P**TDEFFLKDFS  LEHLRVNHFV**P**EAGSRLDQRV  EAGSRLDQRV**P**QIAFVITGGK  LHDAMHETLC**P**GVTDAAKACN  HRVSCSGGRS**P**TVRVSVVANT  TVRVSVVANT**P**SGPVEAFDFD  VSVVANTPSG**P**VEAFDFDEYQ  VEAFDFDEYQ**P**EMLEKFRNMR  EKFRNMRSQH**P**YVLTEDTLKV  VYLNKFRQSS**P**DSVKVVIHFT  EFGRGFMYDR**P**LRLNLLDLDY  NIAEKACCGV**P**CKCSGQRGDR  KCSGQRGDRG**P**IGSIGPKGIP  GDRGPIGSIG**P**KGIPGEDGYR  PIGSIGPKGI**P**GEDGYRGYPG  IPGEDGYRGY**P**GDEGGPGERG  YRGYPGDEGG**P**GERGPPGVNG  GDEGGPGERG**P**PGVNGTQGFQ  DEGGPGERGP**P**GVNGTQGFQG  VNGTQGFQGC**P**GQRGVKGSRG  LDGEDGDKGL**P**GSSGEKGNPG  LPGSSGEKGN**P**GRRGDKGPRG  GNPGRRGDKG**P**RGEKGERGDV  ERGDVGIRGD**P**GNPGQDSQER  DVGIRGDPGN**P**GQDSQERGPK  NPGQDSQERG**P**KGETGDLGPM  GPKGETGDLG**P**MGVPGRDGVP  ETGDLGPMGV**P**GRDGVPGGPG  PMGVPGRDGV**P**GGPGETGKNG  VPGRDGVPGG**P**GETGKNGGFG  GKNGGFGRRG**P**PGAKGNKGGP  PPGAKGNKGG**P**GQPGFEGEQG  AKGNKGGPGQ**P**GFEGEQGTRG  GEQGTRGAQG**P**AGPAGPPGLI  GTRGAQGPAG**P**AGPPGLIGEQ  GAQGPAGPAG**P**PGLIGEQGIS  GLIGEQGISG**P**RGSGGAAGAP  PRGSGGAAGA**P**GERGRTGPLG  GAPGERGRTG**P**LGRKGEPGEP  RTGPLGRKGE**P**GEPGPKGGIG  PLGRKGEPGE**P**GPKGGIGNRG  GRKGEPGEPG**P**KGGIGNRGPR  GPKGGIGNRG**P**RGETGDDGRD  GERGFPGYPG**P**KGNPGEPGLN  FPGYPGPKGN**P**GEPGLNGTTG  YPGPKGNPGE**P**GLNGTTGPKG  GEPGLNGTTG**P**KGIRGRRGNS  GIRGRRGNSG**P**PGIVGQKGDP  IRGRRGNSGP**P**GIVGQKGDPG  PPGIVGQKGD**P**GYPGPAGPKG  IVGQKGDPGY**P**GPAGPKGNRG  GQKGDPGYPG**P**AGPKGNRGDS  GDPGYPGPAG**P**KGNRGDSIDQ  ALIQSIKDKC**P**CCYGPLECPV  IKDKCPCCYG**P**LECPVFPTEL  CPCCYGPLEC**P**VFPTELAFAL  CYGPLECPVF**P**TELAFALDTS  NDLTIAESNC**P**RGARVAVVTY  RKVAVFFSNT**P**TRASPQLREA  FFSNTPTRAS**P**QLREAVLKLS  VLKLSDAGIT**P**LFLTRQEDRQ  NTAVGHALVL**P**AGRDLTDFLE  HVCLDICNID**P**SCGFGSWRPS  DPSCGFGSWR**P**SFRDRRAAGS  AYLVRQLDMS**P**DPKASQHFAR  LVRQLDMSPD**P**KASQHFARVA  FARVAVVQHA**P**SESVDNASMP  PSESVDNASM**P**PVKVEFSLTD  SESVDNASMP**P**VKVEFSLTDY  YTIENVFESA**P**NPRDLKIVVL  IENVFESAPN**P**RDLKIVVLML  IVVLMLTGEV**P**EQQLEEAQRV  IKEVYTFASE**P**NDVFFKLVDK  VDKSTELNEE**P**LMRFGRLLPS  EPLMRFGRLL**P**SFVSSENAFY  VSSENAFYLS**P**DIRKQCDWFQ  KQCDWFQGDQ**P**TKNLVKFGHK  VKFGHKQVNV**P**NNVTSSPTSN  VNVPNNVTSS**P**TSNPVTTTKP  NNVTSSPTSN**P**VTTTKPVTTT  PTSNPVTTTK**P**VTTTKPVTTT  TTTKPVTTTK**P**VTTTTKPVTT  TTKPVTTTTK**P**VTTTTKPVTI  TTKPVTTTTK**P**VTIINQPSVK  TTKPVTIINQ**P**SVKPAAAKPA  VTIINQPSVK**P**AAAKPAPAKP  QPSVKPAAAK**P**APAKPVAAKP  SVKPAAAKPA**P**AKPVAAKPVA  PAAAKPAPAK**P**VAAKPVATKM  PAPAKPVAAK**P**VATKMATVRP  PVATKMATVR**P**PVAVKPATAA  VATKMATVRP**P**VAVKPATAAK  ATVRPPVAVK**P**ATAAKPVAAK  VAVKPATAAK**P**VAAKPAAVRP  ATAAKPVAAK**P**AAVRPPAAAA  PVAAKPAAVR**P**PAAAAAKPVA  VAAKPAAVRP**P**AAAAAKPVAT  VRPPAAAAAK**P**VATKPEVPRP  AAAAKPVATK**P**EVPRPQAAKP  AKPVATKPEV**P**RPQAAKPAAT  PVATKPEVPR**P**QAAKPAATKP  PEVPRPQAAK**P**AATKPATTKP  PQAAKPAATK**P**ATTKPMVKMS  PAATKPATTK**P**MVKMSREVQV  SAKLHWERAE**P**PGPYFYDLTV  AKLHWERAEP**P**GPYFYDLTVT  LHWERAEPPG**P**YFYDLTVTSA  HGSFSTKKSQ**P**PPPQPARSAS  GSFSTKKSQP**P**PPQPARSASS  SFSTKKSQPP**P**PQPARSASSS  FSTKKSQPPP**P**QPARSASSST  TKKSQPPPPQ**P**ARSASSSTIN  STINLMVSTE**P**LALTETDICK  ALTETDICKL**P**KDEGTCRDFI  RDFILKWYYD**P**NTKSCARFWY  SQKECEKVCA**P**VLAKPGVISV  EKVCAPVLAK**P**GVISVMGTTG  ESHPKSMMSK**P**HSEAGTAFIQ  EHMCRLDIDS**P**PITARNTGII  HMCRLDIDSP**P**ITARNTGIIC  RNTGIICTIG**P**ASRSVETLKE  RTATESFASD**P**ILYRPVAVAL  SFASDPILYR**P**VAVALDTKGP  PVAVALDTKG**P**EIRTGLIKGS  SLGSKKGVNL**P**GAAVDLPAVS  VNLPGAAVDL**P**AVSEKDIQDL  VARGDLGIEI**P**AEKVFLAQKM  MIGRCNRAGK**P**VICATQMLES  TQMLESMIKK**P**RPTRAEGSDV  MLESMIKKPR**P**TRAEGSDVAN  LSGETAKGDY**P**LEAVRMQHLI  RSAHQVARYR**P**RAPIIAVTRN  HQVARYRPRA**P**IIAVTRNPQT  RAPIIAVTRN**P**QTARQAHLYR  RQAHLYRGIF**P**VLCKDPVQEA  RGIFPVLCKD**P**VQEAWAEDVD  DVVIVLTGWR**P**GSGFTNTMRV  SGFTNTMRVV**P**VPPVPVVRMT  FTNTMRVVPV**P**PVPVVRMTNT  CMGLRCGRGN**P**QKCIGAHEDV  AMMAGLTAAA**P**RPGVLLLLLS  MAGLTAAAPR**P**GVLLLLLSIL  VLLLLLSILH**P**SRPGGVPGAI  LLLSILHPSR**P**GGVPGAIPGG  ILHPSRPGGV**P**GAIPGGVPGG  GVPGAIPGGV**P**GGVFYPGAGL  PGGVPGGVFY**P**GAGLGALGGG  LGALGGGALG**P**GGKPLKPVPG  GGGALGPGGK**P**LKPVPGGLAG  AGLGAGLGAF**P**AVTFPGALVP  PAVTFPGALV**P**GGVADAAAAY  GLGVSAGAVV**P**QPGAGVKPGK  GVSAGAVVPQ**P**GAGVKPGKVP  VVPQPGAGVK**P**GKVPGVGLPG  PGAGVKPGKV**P**GVGLPGVYPG  KPGKVPGVGL**P**GVYPGGVLPG  VPGVGLPGVY**P**GGVLPGARFP  PGGVLPGARF**P**GVGVLPGVPT  PTGAGVKPKA**P**GVGGAFAGIP  GAFAGIPGVG**P**FGGPQPGVPL  GIPGVGPFGG**P**QPGVPLGYPI  PGVGPFGGPQ**P**GVPLGYPIKA  GPFGGPQPGV**P**LGYPIKAPKL  GPQPGVPLGY**P**IKAPKLPGGY  GVPLGYPIKA**P**KLPGGYGLPY  LGYPIKAPKL**P**GGYGLPYTTG  APKLPGGYGL**P**YTTGKLPYGY  YGLPYTTGKL**P**YGYGPGGVAG  TTGKLPYGYG**P**GGVAGAAGKA  VAGAAGKAGY**P**TGTGVGPQAA  AGYPTGTGVG**P**QAAAAAAAKA  KFGAGAAGVL**P**GVGGAGVPGV  GIGGIAGVGT**P**AAAAAAAAAA  AKYGAAAGLV**P**GGPGFGPGVV  GLVPGGPGFG**P**GVVGVPGAGV  PGFGPGVVGV**P**GAGVPGVGVP  PGAGIPGAAV**P**GVVSPEAAAK  PGAAVPGVVS**P**EAAAKAAAKA  AAKAAKYGAR**P**GVGVGGIPTY  ARPGVGVGGI**P**TYGVGAGGFP  PTYGVGAGGF**P**GFGVGVGGIP  VGGVPGVGGV**P**GVGISPEAQA  VGGVPGVGIS**P**EAQAAAAAKA  GLVPGPQAAV**P**GVPGTGGVPG  PGPQAAVPGV**P**GTGGVPGVGT  GTGGVPGVGT**P**AAAAAKAAAK  AAKAAQFGLV**P**GVGVAPGVGV  FGLVPGVGVA**P**GVGVAPGVGV  VGVAPGVGVA**P**GVGVAPGVGL  VGVAPGVGLA**P**GVGVAPGVGV  VGLAPGVGVA**P**GVGVAPGVGV  VGVAPGVGVA**P**GVGVAPGIGP  VGVAPGVGVA**P**GIGPGGVAAA  ADEGVRRSLS**P**ELREGDPSSS  SLSPELREGD**P**SSSQHLPSTP  EGDPSSSQHL**P**STPSSPRVPG  PSSSQHLPST**P**SSPRVPGALA  SQHLPSTPSS**P**RVPGALAAAK  AKAAKYGAAV**P**GVLGGLGALG  GIPGGVVGAG**P**AAAAAAAKAA  GGLGVGGLGV**P**GVGGLGGIPP  VPGVGGLGGI**P**PAAAAKAAKY  PGVGGLGGIP**P**AAAAKAAKYG  GGVLGGAGQF**P**LGGVAARPGF  QFPLGGVAAR**P**GFGLSPIFPG  LPLLLMMLLL**P**LLVLLCVVSV  RDGPKGEKGE**P**GQGLRGLQGP  PGQGLRGLQG**P**PGKLGPPGSV  GLQGPPGKLG**P**PGSVGAPGSQ  KFFVTNHERM**P**FSKVKALCSE  CSELRGTVAI**P**RNAEENKAIQ  LTYSNWKKDE**P**NDHGSGEDCV  QASHTAVCEF**P**AAPFECVATH  LPLLLMMLLL**P**LSVLLLLTQP  PLSVLLLLTQ**P**WRSLGAEMTT  ANACTLVMCS**P**LESGLPGHDG  VMCSPLESGL**P**GHDGQDGREC  GHDGQDGREC**P**HGEKGDPGSP  RECPHGEKGD**P**GSPGPAGRAG  PHGEKGDPGS**P**GPAGRAGRPG  GEKGDPGSPG**P**AGRAGRPGWV  GRAGRPGWVG**P**IGPKGDNGFV  GRPGWVGPIG**P**KGDNGFVGEP  GDNGFVGEPG**P**KGDTGPRGPP  GEPGPKGDTG**P**RGPPGMPGPA  GPKGDTGPRG**P**PGMPGPAGRE  GPRGPPGMPG**P**AGREGPSGKQ  GMPGPAGREG**P**SGKQGSMGPP  GPSGKQGSMG**P**PGTPGPKGET  GSMGPPGTPG**P**KGETGPKGGV  GTPGPKGETG**P**KGGVGAPGIQ  GAPGIQGFPG**P**SGLKGEKGAP  PSGLKGEKGA**P**GETGAPGRAG  GAPGRAGVTG**P**SGAIGPQGPS  GVTGPSGAIG**P**QGPSGARGPP  GPSGAIGPQG**P**SGARGPPGLK  GPQGPSGARG**P**PGLKGDRGDP  PPGLKGDRGD**P**GETGAKGESG  FSQYKKAVLF**P**DGQAVGEKIF  CREAKGQLAS**P**RSSAENEAVT  DISTEGRFTY**P**TGEILVYSNW  LVYSNWADGE**P**NNSDEGQPEN  GEPNNSDEGQ**P**ENCVEIFPDG  GQPENCVEIF**P**DGKWNDVPCS  IFPDGKWNDV**P**CSKQLLVICE  CGPGNCQSQC**P**GGPTPPGGGD  QSQCPGGPTP**P**GGGDLGSIIS  CHDVIIGRWQ**P**SSADRAANRL  SSADRAANRL**P**GFGVITNIIN  IVGDNTGWSV**P**SSPNFYSQWA  DNTGWSVPSS**P**NFYSQWAAGK  RVGDSLQFNF**P**ANAHNVHEME  NSDNDVERTS**P**VIERLDELGM  PSSVMPPPVM**P**PPSPSSPSPP  RPGARYYRAG**P**RYIQAQVGPI  GPRYIQAQVG**P**IGPRGPPGPP  YIQAQVGPIG**P**RGPPGPPGSP  AQVGPIGPRG**P**PGPPGSPGQQ  GPIGPRGPPG**P**PGSPGQQGYQ  GLRGEPGDSG**P**MGPIGKRGPP  GEPGDSGPMG**P**IGKRGPPGPA  GPIGKRGPPG**P**AGIAGKSGDD  GDDGRDGEPG**P**RGGIGPMGPR  GEPGPRGGIG**P**MGPRGAGGMP  GPRGGIGPMG**P**RGAGGMPGMP  GAGGMPGMPG**P**KGHRGFRGLS  GEQGKSGNQG**P**DGGPGPAGPS  GNQGPDGGPG**P**AGPSGPIGPR  GGPGPAGPSG**P**IGPRGQTGER  GPAGPSGPIG**P**RGQTGERGRD  GLAGPPGPPG**P**IGSTGSPGFP  GIKGAQGLQG**P**VGLSGQPGVA  GASGESGLPG**P**SGFPGPRGMP  GLPGPSGFPG**P**RGMPGTAGSP  GQAGAKGDGG**P**TGEQGRPGAP  GAPGVKGSSG**P**PGDVGAPGHA  GKRGSPGSPG**P**AGSPGPQGDR  GSPGPAGSPG**P**QGDRGLPGSR  GAMGIPGEKG**P**SGEPGAKGPT  GPSGEPGAKG**P**TGDTGRQGNQ  GKDGKQGEQG**P**QGPQGLAGLQ  GKQGEQGPQG**P**QGLAGLQGRA  GKDGETGAAG**P**PGAAGPTGAR  GAAGPPGAAG**P**TGARGPPGPR  GAAGPTGARG**P**PGPRGQQGFQ  GPTGARGPPG**P**RGQQGFQGLA  GAPGDRGNVG**P**RGMPGERGAT  GAKGQGGPPG**P**AGLVGLPGER  GLVGLPGERG**P**KGVGGSKGSR  GSKGSRGDIG**P**RGKAGERGKD  GERGENGLPG**P**SGLAASKGER  GSPGERGSPG**P**AGERGPAGSQ  GSPGPAGERG**P**AGSQGIQGQP  GQPGPPGDAG**P**AGTKGDIGFP  GATGKQGARG**P**RGLAGKRGLR  GSPGQPGLPG**P**SGQPGPSGPA  GLPGPSGQPG**P**SGPAGTAGKQ  GPSGQPGPSG**P**AGTAGKQGVK  GEDGPPGVSG**P**TGAPGQQGER  GMVGLRGETG**P**MGGQGMKGDG  GMKGDGGPPG**P**SGDRGERGNA  GDRGERGNAG**P**QGPTGPSGQA  GERGNAGPQG**P**TGPSGQAGAP  GNAGPQGPTG**P**SGQAGAPGQE  GQRGLPGAAG**P**PGDRGERGEP  GEPGGQGVQG**P**VGAPGSQGPA  GPVGAPGSQG**P**AGIMGMKGEA  GTPGHSGESG**P**PGAPGPRGAR  GESGPPGAPG**P**RGARGEAGGR  GEAGGRGSQG**P**PGKDGQPGPS  GPPGKDGQPG**P**SGRVGPRGPS  GQPGPSGRVG**P**RGPSGDDGRS  GPSGRVGPRG**P**SGDDGRSGPP  QLINGQGAPA**P**YPDPLEPKRE  INGQGAPAPY**P**DPLEPKREVC  GQGAPAPYPD**P**LEPKREVCEL  EPKREVCELN**P**DCDELADQVG  LQDAYQRFYG**P**VVPGYFRQYA  TARRDPCCYH**P**TCNMSNPQIC  CYHPTCNMSN**P**QICGGCIQPN  MIEDIGESDS**P**IPLPNVTSTI  EDIGESDSPI**P**LPNVTSTILE  IGESDSPIPL**P**NVTSTILEKV  LDYCRHHHQH**P**SPQGDDKKDE  YCRHHHQHPS**P**QGDDKKDEKR  KKDEKRLDDI**P**PYDRDFCKVD  KDEKRLDDIP**P**YDRDFCKVDQ  YDRDFCKVDQ**P**TLFELILAAN  ILAANYLDIK**P**LLDVTCKTVA  TVANMIRGKT**P**EEIRKIFNIK  ALLTICLLLF**P**ITALLMDGDQ  ITALLMDGDQ**P**ADRPAERMDY  LMDGDQPADR**P**AERMDYDISS  AKINFLSKRK**P**SAERWRRDCT  YPGCCGGCCG**P**YPNAACHPCG  QVMVQGDGDQ**P**ADRNAVPRDD  GDQPADRNAV**P**RDDNPGGASG  DRNAVPRDDN**P**GGASGKFMNV  VLRRSGCPWE**P**WCGGCWPEWP  QVMVQGDADQ**P**ADRDAVPRDD  ADQPADRDAV**P**RDDNPSGTDG  DRDAVPRDDN**P**SGTDGKFMNV  VLRRFGCPWQ**P**WCGGCWPQWP  PGDWQCVEAC**P**VVPCAEVCQW  VPLCRMMRCL**P**VLIILLLLTA  IILLLLTASA**P**GVVVLPKTED  TASAPGVVVL**P**KTEDDVPMSS  VVLPKTEDDV**P**MSSVYGNGKS  QDCLVCIPCC**P**NNPCCPICVL  CPWCGFTCCL**P**NYCQGLTCTV  CLLLFSLNAV**P**LDGDQPADQP  LNAVPLDGDQ**P**ADQPAERLLD  PLDGDQPADQ**P**AERLLDDISF  LLDDISFENN**P**FYDPAKRCCR  ISFENNPFYD**P**AKRCCRTCFG  PGLCCSEFCL**P**GVCFGGGGFC  CPSGPPKCCK**P**PGSPCRVSSY  SKCGCRYNVH**P**SGWGCGCACS  VLLTICLLLF**P**ITALPMDGDQ  CLLLFPITAL**P**MDGDQPADRL  ITALPMDGDQ**P**ADRLAERMQD  MQDNISSEEH**P**FEKRQRLCCG  VLWPAAPWLV**P**SQITTCCGYN  PPCCLYGSCR**P**FPGCYNALCC  RIIRDECCSN**P**ACRVNNPHVC  CIKPYGFCSL**P**ILKNGLCCSG  CSDPRCNYDH**P**EICGGCIEPH  VVILLLITST**P**SVDARLKAKD  DARLKAKDNM**P**LASFHDNAKR  QTRLINTRCC**P**GKPCCRIGGI  PPKDYNNYDK**P**PVEKPPVYKP  NNYDKPPVEK**P**PVYKPPVEKP  PPVEKPPVYK**P**PVEKPPVYKP  PPVYKPPVEK**P**PVYKPPKYVP  PPVEKPPVYK**P**PKYVPPKEVP  PPLYMNNMYL**P**PVPPPPVVPT  LPPVPPPPVV**P**TFFTPVVPPP  FDPPQNQQNQ**P**PDFANPFIIP  DPPQNQQNQP**P**DFANPFIIPQ  PPDFANPFII**P**QNAAAANQPI  VPFCRMMRCF**P**VFIILLLLIA  IILLLLIASA**P**CFDARTKTDD  DARTKTDDDV**P**LSSLRDNLKR  LCIAGQAGAK**P**SGAESSKGAA  KQEGSEVVKR**P**RRYLYQWLGA  YLYQWLGAPV**P**YPDPLEPRRE  YQWLGAPVPY**P**DPLEPRREVC  WLGAPVPYPD**P**LEPRREVCEL  APVPYPDPLE**P**RREVCELNPD  EPRREVCELN**P**DCDELADHIG  RFPSFGPPRR**P**PGFSPFRRFP  GPPRRPPGFS**P**FRRFPSFGPP  EESDEKKRES**P**DRPPGFSPFR  DEKKRESPDR**P**PGFSPFRIYY  ESPDRPPGFS**P**FRIYYIRFPS  FPTFGPPVVP**P**GFTPFRQSSQ  GPPVVPPGFT**P**FRQSSQRFPT  WRLLRWPPKK**P**PWRLLRWPPK  RMCCKKCCMR**P**ICTCPCCIGP  PICTCPCCIG**P**PGICCPCTCI  RMCCKKCCMR**P**ICMCPCCIGA  TVVTSWSLCT**P**GCTSPGGGSN  PEGGEPGGVG**P**IGPPGERGAP  GEPGGVGPIG**P**PGERGAPGNR  PIGPPGERGA**P**GNRNMPGLIH  ERGAPGNRNM**P**GLIHGRFQWA  GGTEWSVTNF**P**KPPNQMHWSS  TEWSVTNFPK**P**PNQMHWSSKL  EWSVTNFPKP**P**NQMHWSSKLH  SKLHGVEGCD**P**NLEHLCDMVE  EFWGHWEMID**P**LPYWQSDQEH  WGHWEMIDPL**P**YWQSDQEHHD  SDQEHHDYYR**P**NSAWVVHYDQ  QLHWMDYLDA**P**AFQVRVRVYY  LIFFCENQTF**P**EYNWWHINPL  FPEYNWWHIN**P**LKWVEGENER  MCNWMQLNRI**P**LDAANFRMRM  KIHRFTTTTR**P**YIVLQFAHDT  QFAHDTKMST**P**MTQLVAMEGN  MEGNVNDRQD**P**KCMENFEEEY  FEEEYWLYGA**P**GERGETGPPG  FAGPPGADGQ**P**GAKPKTRKNP  PGADGQPGAK**P**KTRKNPRGGW  PGAKPKTRKN**P**RGGWGWTAAP  PRGGWGWTAA**P**IGTQLVMIPK  APIGTQLVMI**P**KVHKDFWQKV  HKDFWQKVQC**P**DKIGFQKIFF  GPPGANGNPG**P**AGPPGPAGKI  GANGNPGPAG**P**PGPAGKIVPM  GNPGPAGPPG**P**AGKIVPMQFV  GPPGPAGKIV**P**MQFVNPGRET  GKIVPMQFVN**P**GRETMPPVLS  QFVNPGRETM**P**PVLSFSKNHT  FVNPGRETMP**P**VLSFSKNHTV  SLGVGTEQAD**P**PCQEAYEHWP  LGVGTEQADP**P**CQEAYEHWPY  PPCQEAYEHW**P**YRKGIMGHKQ  NQMYQKSMQT**P**HGDQRYFKDC  MTTKVSTEQN**P**IQKCLYRDSH  MWSRRKDYWS**P**HADYGYVAEQ  KICWISMKYH**P**VSDHNNKEHC  FTGLQGLPGP**P**GTSGDQGASG  GTSGDQGASG**P**SGPAGPRRPG  GDQGASGPSG**P**AGPRRPGAPG  GASGPSGPAG**P**RRPGAPGSPG  QYDAKGVGLG**P**GPMGLMGPRG  DAKGVGLGPG**P**MGLMGPRGPP  LGPGPMGLMG**P**RGPPGATGPP  GPMGLMGPRG**P**PGATGPPGSP  PMGLMGPRGP**P**GATGPPGSPG  GPRGPPGATG**P**PGSPGFQGPP  PRGPPGATGP**P**GSPGFQGPPG  PPGATGPPGS**P**GFQGPPGEPG  GPPGSPGFQG**P**PGEPGEPGQT  PPGSPGFQGP**P**GEPGEPGQTG  SPGFQGPPGE**P**GEPGQTGPAG  FQGPPGEPGE**P**GQTGPAGSRG  GEPGEPGQTG**P**AGSRGPAGPP  GQTGPAGSRG**P**AGPPGKAGED  GPAGSRGPAG**P**PGKAGEDGHP  PAGSRGPAGP**P**GKAGEDGHPG  KAGEDGHPGK**P**GRPGERGVVG  EDGHPGKPGR**P**GERGVVGPQG  GRPGERGVVG**P**QGARGFPGTP  VVGPQGARGF**P**GTPGLPGFKG  PQGARGFPGT**P**GLPGFKGIRG  ARGFPGTPGL**P**GFKGIRGHNG  HNGLDGLKGQ**P**GAPGVKGEPG  LDGLKGQPGA**P**GVKGEPGAPG  QPGAPGVKGE**P**GAPGENGTPG  APGVKGEPGA**P**GENGTPGQIG  EPGAPGENGT**P**GQIGARGLPG  TPGQIGARGL**P**GERGRVGGPG  LPGERGRVGG**P**GPAGARGSDG  GERGRVGGPG**P**AGARGSDGSV  GARGSDGSVG**P**VGPAGPIGSA  GSDGSVGPVG**P**AGPIGSAGPP  GSVGPVGPAG**P**IGSAGPPGFP  GPAGPIGSAG**P**PGFPGAPGPK  PIGSAGPPGF**P**GAPGPKGEIG  GPPGFPGAPG**P**KGEIGPVGNP  GAPGPKGEIG**P**VGNPGPSGPA  PKGEIGPVGN**P**GPSGPAGPRG  GEIGPVGNPG**P**SGPAGPRGEA  GPVGNPGPSG**P**AGPRGEAGLP  GNPGPSGPAG**P**RGEAGLPGVS  PAGPRGEAGL**P**GVSGPVGPPG  GEAGLPGVSG**P**VGPPGNPGAN  GLPGVSGPVG**P**PGNPGANGLA  LPGVSGPVGP**P**GNPGANGLAG  VSGPVGPPGN**P**GANGLAGAKG  LAGAKGAAGL**P**GVAGAPGLPG  LPGVAGAPGL**P**GPRGIPGPVG  GVAGAPGLPG**P**RGIPGPVGAA  APGLPGPRGI**P**GPVGAAGATG  GLPGPRGIPG**P**VGAAGATGAR  ATGARGIVGE**P**GPAGSKGESG  GARGIVGEPG**P**AGSKGESGSK  GSKGEPGSAG**P**QGPPGPSGEE  GEPGSAGPQG**P**PGPSGEEGKR  GSAGPQGPPG**P**SGEEGKRGPN  GPSGEEGKRG**P**NGEAGSAGPA  GPNGEAGSAG**P**AGPPGLRGGP  GEAGSAGPAG**P**PGLRGGPGSR  EAGSAGPAGP**P**GLRGGPGSRG  PAGPPGLRGG**P**GSRGLPGADG  LRGGPGSRGL**P**GADGRAGVMG  GADGRAGVMG**P**PGSRGASGPA  ADGRAGVMGP**P**GSRGASGPAG  GPPGSRGASG**P**AGVRGPSGDS  GASGPAGVRG**P**SGDSGRPGEP  VRGPSGDSGR**P**GEPGVMGPRG  PSGDSGRPGE**P**GVMGPRGLPG  GRPGEPGVMG**P**RGLPGSPGNV  EPGVMGPRGL**P**GSPGNVGPAG  VMGPRGLPGS**P**GNVGPAGKEG  GLPGSPGNVG**P**AGKEGPAGLP  GNVGPAGKEG**P**AGLPGIDGRP  PAGKEGPAGL**P**GIDGRPGPIG  PAGLPGIDGR**P**GPIGPAGARG  GLPGIDGRPG**P**IGPAGARGEP  GIDGRPGPIG**P**AGARGEPGNI  ARGEPGNIGF**P**GPKGPAGDPG  GEPGNIGFPG**P**KGPAGDPGKN  GNIGFPGPKG**P**AGDPGKNGDK  FPGPKGPAGD**P**GKNGDKGHAG  GDKGHAGLAG**P**RGAPGPDGNN  HAGLAGPRGA**P**GPDGNNGAQG  GLAGPRGAPG**P**DGNNGAQGPP  GPDGNNGAQG**P**PGLQGVQGGK  PDGNNGAQGP**P**GLQGVQGGKG  GVQGGKGEQG**P**AGPPGFQGLP  GGKGEQGPAG**P**PGFQGLPGPS  GPPGFQGLPG**P**SGTAGEAGKP  GIPGEFGLPG**P**AGPRGERGPP  GEFGLPGPAG**P**RGERGPPGQS  GPAGPRGERG**P**PGQSGAAGPT  PAGPRGERGP**P**GQSGAAGPTG  GPPGQSGAAG**P**TGPIGSRGPS  GQSGAAGPTG**P**IGSRGPSGPP  GPTGPIGSRG**P**SGPPGPDGNK  GSRGPSGPPG**P**DGNKGEPGVV  PPGPDGNKGE**P**GVVGAPGTAG  NKGEPGVVGA**P**GTAGPSGPVG  GVVGAPGTAG**P**SGPVGLPGER  GAPGTAGPSG**P**VGLPGERGAA  TAGPSGPVGL**P**GERGAAGIPG  LPGERGAAGI**P**GGKGEKGETG  GAPGAVGAPG**P**AGATGDRGEA  GATGDRGEAG**P**AGSAGPAGPR  GEAGPAGSAG**P**AGPRGSPGER  GPAGSAGPAG**P**RGSPGERGEV  SAGPAGPRGS**P**GERGEVGPAG  GSPGERGEVG**P**AGPNGFAGPA  GERGEVGPAG**P**NGFAGPAGAA  GPAGPNGFAG**P**AGAAGQAGAK  GAKGERGTKG**P**KGENGPVGPT  GTKGPKGENG**P**VGPTGPVGAA  GPKGENGPVG**P**TGPVGAAGPA  GENGPVGPTG**P**VGAAGPAGPN  GPTGPVGAAG**P**AGPNGPPGPA  GPVGAAGPAG**P**NGPPGPAGSR  GAAGPAGPNG**P**PGPAGSRGDG  AAGPAGPNGP**P**GPAGSRGDGG  GPAGPNGPPG**P**AGSRGDGGPP  GPAGSRGDGG**P**PGATGFPGAA  GFPGAAGRTG**P**PGPAGITGPP  FPGAAGRTGP**P**GPAGITGPPG  GAAGRTGPPG**P**AGITGPPGPP  GPPGPAGITG**P**PGPPGAAGKE  GPAGITGPPG**P**PGAAGKEGLR  GAAGKEGLRG**P**RGDQGPVGRT  GLRGPRGDQG**P**VGRTGETGAS  GRTGETGASG**P**PGFAGEKGSS  FAGEKGSSGE**P**GTAGPPGAPG  GSSGEPGTAG**P**PGAPGPQGIL  SSGEPGTAGP**P**GAPGPQGILG  EPGTAGPPGA**P**GPQGILGPPG  GTAGPPGAPG**P**QGILGPPGIL  GAPGPQGILG**P**PGILGLPGSR  APGPQGILGP**P**GILGLPGSRG  ILGPPGILGL**P**GSRGERGLPG  GVAGAVGEPG**P**LGIAGPPGAR  GEPGPLGIAG**P**PGARGPPGAV  GIAGPPGARG**P**PGAVGSPGVN  IAGPPGARGP**P**GAVGSPGVNG  ARGPPGAVGS**P**GVNGAPGEAG  AVGSPGVNGA**P**GEAGRDGNPG  APGEAGRDGN**P**GSDGPPGRDG  GRDGNPGSDG**P**PGRDGLPGHK  RDGNPGSDGP**P**GRDGLPGHKG  SDGPPGRDGL**P**GHKGERGYPG  LPGHKGERGY**P**GNAGPVGTAG  GERGYPGNAG**P**VGTAGAPGPQ  NAGPVGTAGA**P**GPQGPLGPAG  GPVGTAGAPG**P**QGPLGPAGKH  GTAGAPGPQG**P**LGPAGKHGNR  GAPGPQGPLG**P**AGKHGNRGEP  GKHGNRGEPG**P**AGSVGPVGAV  GEPGPAGSVG**P**VGAVGPRGPS  GSVGPVGAVG**P**RGPSGPQGAR  GPVGAVGPRG**P**SGPQGARGDK  GAVGPRGPSG**P**QGARGDKGEA  GDKGEAGDKG**P**RGLPGFKGHN  EAGDKGPRGL**P**GFKGHNGLQG  FKGHNGLQGL**P**GLAGQHGDQG  LAGQHGDQGS**P**GSVGPAGPRG  GDQGSPGSVG**P**AGPRGPAGPS  GSPGSVGPAG**P**RGPAGPSGPV  GSVGPAGPRG**P**AGPSGPVGKD  GPAGPRGPAG**P**SGPVGKDGRP  GPRGPAGPSG**P**VGKDGRPGHA  PSGPVGKDGR**P**GHAGAVGPAG  GRPGHAGAVG**P**AGVRGSQGSQ  GVRGSQGSQG**P**SGPPGPPGPP  GSQGSQGPSG**P**PGPPGPPGPP  SQGSQGPSGP**P**GPPGPPGPPG  GSQGPSGPPG**P**PGPPGPPGPS  SQGPSGPPGP**P**GPPGPPGPSG  GPSGPPGPPG**P**PGPPGPSGGG  PSGPPGPPGP**P**GPPGPSGGGY  GPPGPPGPPG**P**PGPSGGGYDF  PPGPPGPPGP**P**GPSGGGYDFG  GPPGPPGPPG**P**SGGGYDFGYD  CSRPTCRMEY**P**ELCGGRRRRG  CMRYGCCPDD**P**CCGYRMCVPC  GARGPSGPQG**P**SGAPGPKGVQ  GPQGPSGAPG**P**KGVQGPPGPQ  GAPGPKGVQG**P**PGPQGPRGLT  GPKGVQGPPG**P**QGPRGLTGPI  GVQGPPGPQG**P**RGLTGPIGPP  GPQGPRGLTG**P**IGPPGPAGAP  GAPGDKGEAG**P**SGPPGPTGAR  GDKGEAGPSG**P**PGPTGARGSA  DKGEAGPSGP**P**GPTGARGSAG  GEAGPSGPPG**P**TGARGSAGPP  GPTGARGSAG**P**PGATGFPGAA  PGAAGRGETG**P**AGPAGPPGPA  AGRGETGPAG**P**AGPPGPAGAR  GETGPAGPAG**P**PGPAGARRAG  GPAGPAGPPG**P**AGARRAGAPG  GSNGEPGSAG**P**PGPAGLRGLP  GEPGSAGPPG**P**AGLRGLPGES  PPGPAGLRGL**P**GESGAVGPAG  GLPGESGAVG**P**AGPPGSRRSG  GESGAVGPAG**P**PGSRRSGPPG  SPLLEFFELL**P**SQDRSCCIQK  CPDTYYFGCC**P**GYATCMSINA  DKCINRLCFD**P**GHHGPDFCLR  ALCAGILAEA**P**RVRAQHRERV  VRSFLEGLVL**P**FSGAASAQGV  RFATVQYSDD**P**RTEFGLDALG  ILHVADHVFL**P**QLARPGVPKV  DHVFLPQLAR**P**GVPKVCILIT  FLPQLARPGV**P**KVCILITDGK  LFAVGIKNAD**P**EELKRVASQP  PEELKRVASQ**P**TSDFFFFVND  NDFSILRTLL**P**LVSRRVCTTA  RRVCTTAGGV**P**VTRPPDDSTS  TTAGGVPVTR**P**PDDSTSAPRD  TAGGVPVTRP**P**DDSTSAPRDL  TRPPDDSTSA**P**RDLVLSEPSS  SAPRDLVLSE**P**SSQSLRVQWT  LRVQWTAASG**P**VTGYKVQYTP  PVTGYKVQYT**P**LTGLGQPLPS  QYTPLTGLGQ**P**LPSERQEVNV  TPLTGLGQPL**P**SERQEVNVPA  LPSERQEVNV**P**AGETSVRLRG  ETSVRLRGLR**P**LTEYQVTVIA  GTARTTALEG**P**ELTIQNTTAH  HSLLVAWRSV**P**GATGYRVTWR  RVTWRVLSGG**P**TQQQELGPGQ  GGPTQQQELG**P**GQGSVLLRDL  QGSVLLRDLE**P**GTDYEVTVST  VSTLFGRSVG**P**ATSLMARTDA  TDASVEQTLR**P**VILGPTSILL  EQTLRPVILG**P**TSILLSWNLV  TSILLSWNLV**P**EARGYRLEWR  LEWRRETGLE**P**PQKVVLPSDV  EWRRETGLEP**P**QKVVLPSDVT  GLEPPQKVVL**P**SDVTRYQLDG  VTRYQLDGLQ**P**GTEYRLTLYT  TLLEGHEVAT**P**ATVVPTGPEL  HEVATPATVV**P**TGPELPVSPV  ATPATVVPTG**P**ELPVSPVTDL  ATVVPTGPEL**P**VSPVTDLQAT  VPTGPELPVS**P**VTDLQATELP  PVTDLQATEL**P**GQRVRVSWSP  PGQRVRVSWS**P**VPGATQYRII  QRVRVSWSPV**P**GATQYRIIVR  TQGVERTLVL**P**GSQTAFDLDD  YTVRVSARVG**P**REGSASVLTV  SASVLTVRRE**P**ETPLAVPGLR  VLTVRREPET**P**LAVPGLRVVV  RREPETPLAV**P**GLRVVVSDAT  DATRVRVAWG**P**VPGASGFRIS  TRVRVAWGPV**P**GASGFRISWS  FRISWSTGSG**P**ESSQTLPPDS  GSGPESSQTL**P**PDSTATDITG  SGPESSQTLP**P**DSTATDITGL  STATDITGLQ**P**GTTYQVAVSV  VSVLRGREEG**P**AAVIVARTDP  PAAVIVARTD**P**LGPVRTVHVT  VIVARTDPLG**P**VRTVHVTQAS  SSVTITWTRV**P**GATGYRVSWH  YRVSWHSAHG**P**EKSQLVSGEA  ATVAELDGLE**P**DTEYTVHVRA  VRAHVAGVDG**P**PASVVVRTAP  RAHVAGVDGP**P**ASVVVRTAPE  PPASVVVRTA**P**EPVGRVSRLQ  ASVVVRTAPE**P**VGRVSRLQIL  RLAWGRSEGG**P**MRHQILPGNT  EGGPMRHQIL**P**GNTDSAEIRG  TALVGDREGT**P**VSIVVTTPPE  GTPVSIVVTT**P**PEAPPALGTL  TPVSIVVTTP**P**EAPPALGTLH  SIVVTTPPEA**P**PALGTLHVVQ  IVVTTPPEAP**P**ALGTLHVVQR  GEHSLRLRWE**P**VPRAQGFLLH  HSLRLRWEPV**P**RAQGFLLHWQ  RAQGFLLHWQ**P**EGGQEQSRVL  GGQEQSRVLG**P**ELSSYHLDGL  LSSYHLDGLE**P**ATQYRVRLSV  QYRVRLSVLG**P**AGEGPSAEVT  LSVLGPAGEG**P**SAEVTARTES  SAEVTARTES**P**RVPSIELRVV  VTARTESPRV**P**SIELRVVDTS  SIDSVTLAWT**P**VSRASSYILS  RASSYILSWR**P**LRGPGQEVPG  YILSWRPLRG**P**GQEVPGSPQT  RPLRGPGQEV**P**GSPQTLPGIS  RGPGQEVPGS**P**QTLPGISSSQ  QEVPGSPQTL**P**GISSSQRVTG  SSSQRVTGLE**P**GVSYIFSLTP  PGVSYIFSLT**P**VLDGVRGPEA  LTPVLDGVRG**P**EASVTQTPVC  RGPEASVTQT**P**VCPRGLADVV  EASVTQTPVC**P**RGLADVVFLP  PRGLADVVFL**P**HATQDNAHRA  VLERLVLALG**P**LGPQAVQVGL  RLVLALGPLG**P**QAVQVGLLSY  QVGLLSYSHR**P**SPLFPLNGSH  GLLSYSHRPS**P**LFPLNGSHDL  SYSHRPSPLF**P**LNGSHDLGII  GIILQRIRDM**P**YMDPSGNNLG  QRIRDMPYMD**P**SGNNLGTAVV  VVTAHRYMLA**P**DAPGRRQHVP  AHRYMLAPDA**P**GRRQHVPGVM  PDAPGRRQHV**P**GVMVLLVDEP  PGVMVLLVDE**P**LRGDIFSPIR  DEPLRGDIFS**P**IREAQASGLN  VVMLGMAGAD**P**EQLRRLAPGM  ADPEQLRRLA**P**GMDSVQTFFA  VQTFFAVDDG**P**SLDQAVSGLA  ALCQASFTTQ**P**RPEPCPVYCP  CQASFTTQPR**P**EPCPVYCPKG  ASFTTQPRPE**P**CPVYCPKGQK  FTTQPRPEPC**P**VYCPKGQKGE  PRPEPCPVYC**P**KGQKGEPGEM  VYCPKGQKGE**P**GEMGLRGQVG  GEMGLRGQVG**P**PGDPGLPGRT  EMGLRGQVGP**P**GDPGLPGRTG  LRGQVGPPGD**P**GLPGRTGAPG  QVGPPGDPGL**P**GRTGAPGPQG  DPGLPGRTGA**P**GPQGPPGSAT  GLPGRTGAPG**P**QGPPGSATAK  GRTGAPGPQG**P**PGSATAKGER  RTGAPGPQGP**P**GSATAKGERG  SATAKGERGF**P**GADGRPGSPG  ERGFPGADGR**P**GSPGRAGNPG  FPGADGRPGS**P**GRAGNPGTPG  RPGSPGRAGN**P**GTPGAPGLKG  SPGRAGNPGT**P**GAPGLKGSPG  RAGNPGTPGA**P**GLKGSPGLPG  TPGAPGLKGS**P**GLPGPRGDPG  APGLKGSPGL**P**GPRGDPGERG  GLKGSPGLPG**P**RGDPGERGPR  SPGLPGPRGD**P**GERGPRGPKG  GPRGDPGERG**P**RGPKGEPGAP  GDPGERGPRG**P**KGEPGAPGQV  ERGPRGPKGE**P**GAPGQVIGGE  PRGPKGEPGA**P**GQVIGGEGPG  APGQVIGGEG**P**GLPGRKGDPG  QVIGGEGPGL**P**GRKGDPGPSG  GPGLPGRKGD**P**GPSGPPGPRG  GLPGRKGDPG**P**SGPPGPRGPL  GRKGDPGPSG**P**PGPRGPLGDP  RKGDPGPSGP**P**GPRGPLGDPG  GDPGPSGPPG**P**RGPLGDPGPR  GPSGPPGPRG**P**LGDPGPRGPP  PPGPRGPLGD**P**GPRGPPGLPG  GPRGPLGDPG**P**RGPPGLPGTA  GPLGDPGPRG**P**PGLPGTAMKG  PLGDPGPRGP**P**GLPGTAMKGD  DPGPRGPPGL**P**GTAMKGDKGD  GDKGDRGERG**P**PGPGEGGIAP  DKGDRGERGP**P**GPGEGGIAPG  GDRGERGPPG**P**GEGGIAPGEP  PPGPGEGGIA**P**GEPGLPGLPG  PGEGGIAPGE**P**GLPGLPGSPG  GGIAPGEPGL**P**GLPGSPGPQG  APGEPGLPGL**P**GSPGPQGPVG  EPGLPGLPGS**P**GPQGPVGPPG  GLPGLPGSPG**P**QGPVGPPGKK  GLPGSPGPQG**P**VGPPGKKGEK  GSPGPQGPVG**P**PGKKGEKGDS  SPGPQGPVGP**P**GKKGEKGDSE  GEKGDSEDGA**P**GLPGQPGSPG  GDSEDGAPGL**P**GQPGSPGEQG  EDGAPGLPGQ**P**GSPGEQGPRG  APGLPGQPGS**P**GEQGPRGPPG  GQPGSPGEQG**P**RGPPGAIGPK  GSPGEQGPRG**P**PGAIGPKGDR  SPGEQGPRGP**P**GAIGPKGDRG  GPRGPPGAIG**P**KGDRGFPGPL  AIGPKGDRGF**P**GPLGEAGEKG  GPKGDRGFPG**P**LGEAGEKGER  GEAGEKGERG**P**PGPAGSRGLP  EAGEKGERGP**P**GPAGSRGLPG  GEKGERGPPG**P**AGSRGLPGVA  PPGPAGSRGL**P**GVAGRPGAKG  SRGLPGVAGR**P**GAKGPEGPPG  GVAGRPGAKG**P**EGPPGPTGRQ  GRPGAKGPEG**P**PGPTGRQGEK  RPGAKGPEGP**P**GPTGRQGEKG  GAKGPEGPPG**P**TGRQGEKGEP  PTGRQGEKGE**P**GRPGDPAVVG  RQGEKGEPGR**P**GDPAVVGPAV  EKGEPGRPGD**P**AVVGPAVAGP  GRPGDPAVVG**P**AVAGPKGEKG  PAVVGPAVAG**P**KGEKGDVGPA  GPKGEKGDVG**P**AGPRGATGVQ  GEKGDVGPAG**P**RGATGVQGER  GATGVQGERG**P**PGLVLPGDPG  ATGVQGERGP**P**GLVLPGDPGP  GERGPPGLVL**P**GDPGPKGDPG  GPPGLVLPGD**P**GPKGDPGDRG  PGLVLPGDPG**P**KGDPGDRGPI  LPGDPGPKGD**P**GDRGPIGLTG  GPKGDPGDRG**P**IGLTGRAGPP  GPIGLTGRAG**P**PGDSGPPGEK  PIGLTGRAGP**P**GDSGPPGEKG  GRAGPPGDSG**P**PGEKGDPGRP  RAGPPGDSGP**P**GEKGDPGRPG  DSGPPGEKGD**P**GRPGPPGPVG  PPGEKGDPGR**P**GPPGPVGPRG  GEKGDPGRPG**P**PGPVGPRGRD  EKGDPGRPGP**P**GPVGPRGRDG  GDPGRPGPPG**P**VGPRGRDGEV  GRPGPPGPVG**P**RGRDGEVGEK  GEVGEKGDEG**P**PGDPGLPGKA  EVGEKGDEGP**P**GDPGLPGKAG  EKGDEGPPGD**P**GLPGKAGERG  DEGPPGDPGL**P**GKAGERGLRG  KAGERGLRGA**P**GVRGPVGEKG  GLRGAPGVRG**P**VGEKGDQGDP  PVGEKGDQGD**P**GEDGRNGSPG  DPGEDGRNGS**P**GSSGPKGDRG  GRNGSPGSSG**P**KGDRGEPGPP  SSGPKGDRGE**P**GPPGPPGRLV  GPKGDRGEPG**P**PGPPGRLVDT  PKGDRGEPGP**P**GPPGRLVDTG  GDRGEPGPPG**P**PGRLVDTGPG  DRGEPGPPGP**P**GRLVDTGPGA  GPPGRLVDTG**P**GAREKGEPGD  TGPGAREKGE**P**GDRGQEGPRG  GEPGDRGQEG**P**RGPKGDPGLP  GDRGQEGPRG**P**KGDPGLPGAP  QEGPRGPKGD**P**GLPGAPGERG  PRGPKGDPGL**P**GAPGERGIEG  PKGDPGLPGA**P**GERGIEGFRG  GERGIEGFRG**P**PGPQGDPGVR  ERGIEGFRGP**P**GPQGDPGVRG  GIEGFRGPPG**P**QGDPGVRGPA  FRGPPGPQGD**P**GVRGPAGEKG  GPQGDPGVRG**P**AGEKGDRGPP  GPAGEKGDRG**P**PGLDGRSGLD  PAGEKGDRGP**P**GLDGRSGLDG  LDGRSGLDGK**P**GAAGPSGPNG  GLDGKPGAAG**P**SGPNGAAGKA  GKPGAAGPSG**P**NGAAGKAGDP  PNGAAGKAGD**P**GRDGLPGLRG  KAGDPGRDGL**P**GLRGEQGLPG  LPGLRGEQGL**P**GPSGPPGLPG  GLRGEQGLPG**P**SGPPGLPGKP  GEQGLPGPSG**P**PGLPGKPGED  EQGLPGPSGP**P**GLPGKPGEDG  LPGPSGPPGL**P**GKPGEDGKPG  PSGPPGLPGK**P**GEDGKPGLNG  LPGKPGEDGK**P**GLNGKNGEPG  KPGLNGKNGE**P**GDPGEDGRKG  LNGKNGEPGD**P**GEDGRKGEKG  GASGREGRDG**P**KGERGAPGIL  RDGPKGERGA**P**GILGPQGPPG  GERGAPGILG**P**QGPPGLPGPV  GAPGILGPQG**P**PGLPGPVGPP  APGILGPQGP**P**GLPGPVGPPG  ILGPQGPPGL**P**GPVGPPGQGF  GPQGPPGLPG**P**VGPPGQGFPG  GPPGLPGPVG**P**PGQGFPGVPG  PPGLPGPVGP**P**GQGFPGVPGG  GPVGPPGQGF**P**GVPGGTGPKG  GPPGQGFPGV**P**GGTGPKGDRG  GFPGVPGGTG**P**KGDRGETGSK  ETGSKGEQGL**P**GERGLRGEPG  LPGERGLRGE**P**GSVPNVDRLL  RGLRGEPGSV**P**NVDRLLETAG  TWDESSGSFL**P**VPERRRGPKG  DESSGSFLPV**P**ERRRGPKGDS  FLPVPERRRG**P**KGDSGEQGPP  GPKGDSGEQG**P**PGKEGPIGFP  PKGDSGEQGP**P**GKEGPIGFPG  GEQGPPGKEG**P**IGFPGERGLK  PPGKEGPIGF**P**GERGLKGDRG  ERGLKGDRGD**P**GPQGPPGLAL  GLKGDRGDPG**P**QGPPGLALGE  GDRGDPGPQG**P**PGLALGERGP  DRGDPGPQGP**P**GLALGERGPP  PPGLALGERG**P**PGPSGLAGEP  PGLALGERGP**P**GPSGLAGEPG  LALGERGPPG**P**SGLAGEPGKP  PPGPSGLAGE**P**GKPGIPGLPG  PSGLAGEPGK**P**GIPGLPGRAG  LAGEPGKPGI**P**GLPGRAGGVG  EPGKPGIPGL**P**GRAGGVGEAG  RAGGVGEAGR**P**GERGERGEKG  GERGEQGRDG**P**PGLPGTPGPP  ERGEQGRDGP**P**GLPGTPGPPG  EQGRDGPPGL**P**GTPGPPGPPG  RDGPPGLPGT**P**GPPGPPGPKV  GPPGLPGTPG**P**PGPPGPKVSV  PPGLPGTPGP**P**GPPGPKVSVD  GLPGTPGPPG**P**PGPKVSVDEP  LPGTPGPPGP**P**GPKVSVDEPG  GTPGPPGPPG**P**KVSVDEPGPG  PPGPKVSVDE**P**GPGLSGEQGP  GPKVSVDEPG**P**GLSGEQGPPG  PGPGLSGEQG**P**PGLKGAKGEP  GPGLSGEQGP**P**GLKGAKGEPG  PPGLKGAKGE**P**GSNGDQGPKG  GEPGSNGDQG**P**KGDRGVPGIK  DQGPKGDRGV**P**GIKGDRGEPG  VPGIKGDRGE**P**GPRGQDGNPG  GIKGDRGEPG**P**RGQDGNPGLP  EPGPRGQDGN**P**GLPGERGMAG  PRGQDGNPGL**P**GERGMAGPEG  GLPGERGMAG**P**EGKPGLQGPR  GPEGKPGLQG**P**RGPPGPVGGH  GKPGLQGPRG**P**PGPVGGHGDP  GLQGPRGPPG**P**VGGHGDPGPP  GPVGGHGDPG**P**PGAPGLAGPA  GHGDPGPPGA**P**GLAGPAGPQG  GPPGAPGLAG**P**AGPQGPSGLK  GAPGLAGPAG**P**QGPSGLKGEP  GLAGPAGPQG**P**SGLKGEPGET  PQGPSGLKGE**P**GETGPPGRGL  GLKGEPGETG**P**PGRGLTGPTG  LKGEPGETGP**P**GRGLTGPTGA  TGPPGRGLTG**P**TGAVGLPGPP  LTGPTGAVGL**P**GPPGPSGLVG  GPTGAVGLPG**P**PGPSGLVGPQ  PTGAVGLPGP**P**GPSGLVGPQG  GAVGLPGPPG**P**SGLVGPQGSP  GPPGPSGLVG**P**QGSPGLPGQV  PSGLVGPQGS**P**GLPGQVGETG  LVGPQGSPGL**P**GQVGETGKPG  LPGQVGETGK**P**GAPGRDGASG  QVGETGKPGA**P**GRDGASGKDG  ASGKDGDRGS**P**GVPGSPGLPG  KDGDRGSPGV**P**GSPGLPGPVG  DRGSPGVPGS**P**GLPGPVGPKG  SPGVPGSPGL**P**GPVGPKGEPG  GVPGSPGLPG**P**VGPKGEPGPT  GSPGLPGPVG**P**KGEPGPTGAP  LPGPVGPKGE**P**GPTGAPGQAV  GPVGPKGEPG**P**TGAPGQAVVG  PKGEPGPTGA**P**GQAVVGLPGA  GAPGQAVVGL**P**GAKGEKGAPG  LPGAKGEKGA**P**GGLAGDLVGE  GGLAGDLVGE**P**GAKGDRGLPG  EPGAKGDRGL**P**GPRGEKGEAG  GAKGDRGLPG**P**RGEKGEAGRA  EKGEAGRAGE**P**GDPGEDGQKG  EAGRAGEPGD**P**GEDGQKGAPG  DPGEDGQKGA**P**GPKGFKGDPG  GEDGQKGAPG**P**KGFKGDPGVG  APGPKGFKGD**P**GVGVPGSPGP  GFKGDPGVGV**P**GSPGPPGPPG  GDPGVGVPGS**P**GPPGPPGVKG  PGVGVPGSPG**P**PGPPGVKGDL  GVGVPGSPGP**P**GPPGVKGDLG  GVPGSPGPPG**P**PGVKGDLGLP  VPGSPGPPGP**P**GVKGDLGLPG  PPGVKGDLGL**P**GLPGAPGVVG  VKGDLGLPGL**P**GAPGVVGFPG  DLGLPGLPGA**P**GVVGFPGQTG  LPGAPGVVGF**P**GQTGPRGEMG  GVVGFPGQTG**P**RGEMGQPGPS  QTGPRGEMGQ**P**GPSGERGLAG  GPRGEMGQPG**P**SGERGLAGPP  GPSGERGLAG**P**PGREGIPGPL  PSGERGLAGP**P**GREGIPGPLG  LAGPPGREGI**P**GPLGPPGPPG  GPPGREGIPG**P**LGPPGPPGSV  GREGIPGPLG**P**PGPPGSVGPP  REGIPGPLGP**P**GPPGSVGPPG  GIPGPLGPPG**P**PGSVGPPGAS  IPGPLGPPGP**P**GSVGPPGASG  GPPGPPGSVG**P**PGASGLKGDK  PPGPPGSVGP**P**GASGLKGDKG  ASGLKGDKGD**P**GVGLPGPRGE  GDKGDPGVGL**P**GPRGERGEPG  KGDPGVGLPG**P**RGERGEPGIR  LPGPRGERGE**P**GIRGEDGRPG  EPGIRGEDGR**P**GQEGPRGLTG  GEDGRPGQEG**P**RGLTGPPGSR  GQEGPRGLTG**P**PGSRGERGEK  QEGPRGLTGP**P**GSRGERGEKG  DKGDSAVILG**P**PGPRGAKGDM  KGDSAVILGP**P**GPRGAKGDMG  DSAVILGPPG**P**RGAKGDMGER  GAKGDMGERG**P**RGLDGDKGPR  GPRGLDGDKG**P**RGDNGDPGDK  DKGPRGDNGD**P**GDKGSKGEPG  DPGDKGSKGE**P**GDKGSAGLPG  EPGDKGSAGL**P**GLRGLLGPQG  GLPGLRGLLG**P**QGQPGAAGIP  LRGLLGPQGQ**P**GAAGIPGDPG  PQGQPGAAGI**P**GDPGSPGKDG  QPGAAGIPGD**P**GSPGKDGVPG  AAGIPGDPGS**P**GKDGVPGIRG  DPGSPGKDGV**P**GIRGEKGDVG  GEKGDVGFMG**P**RGLKGERGVK  GEKGDKGEAG**P**PGRPGLAGHK  EKGDKGEAGP**P**GRPGLAGHKG  DKGEAGPPGR**P**GLAGHKGEMG  GAPGKEGLIG**P**KGDRGFDGQP  PKGDRGFDGQ**P**GPKGDQGEKG  GDRGFDGQPG**P**KGDQGEKGER  DQGEKGERGT**P**GIGGFPGPSG  ERGTPGIGGF**P**GPSGNDGSAG  GTPGIGGFPG**P**SGNDGSAGPP  GPSGNDGSAG**P**PGPPGSVGPR  PSGNDGSAGP**P**GPPGSVGPRG  GNDGSAGPPG**P**PGSVGPRGPE  NDGSAGPPGP**P**GSVGPRGPEG  GPPGPPGSVG**P**RGPEGLQGQK  GPPGSVGPRG**P**EGLQGQKGER  GLQGQKGERG**P**PGERVVGAPG  LQGQKGERGP**P**GERVVGAPGV  GPPGERVVGA**P**GVPGAPGERG  GERVVGAPGV**P**GAPGERGEQG  VVGAPGVPGA**P**GERGEQGRPG  APGERGEQGR**P**GPAGPRGEKG  GERGEQGRPG**P**AGPRGEKGEA  GEQGRPGPAG**P**RGEKGEAALT  QGQFIASGSR**P**LPSYAADTAG  QFIASGSRPL**P**SYAADTAGSQ  DTAGSQLHAV**P**VLRVSHAEEE  VSHAEEEERV**P**PEDDEYSEYS  SHAEEEERVP**P**EDDEYSEYSE  SEYSVEEYQD**P**EAPWDSDDPC  SVEEYQDPEA**P**WDSDDPCSLP  DPEAPWDSDD**P**CSLPLDEGSC  PWDSDDPCSL**P**LDEGSCTAYT  AVTGSTEACH**P**FVYGGCGGNA  GTREACERRC**P**PRVVQSQGTG  TREACERRCP**P**RVVQSQGTGT  FQRKMRYWLL**P**PFLAIVYFCT  QRKMRYWLLP**P**FLAIVYFCTI  FCTIVQGQVA**P**PTRLRYNVIS  CTIVQGQVAP**P**TRLRYNVISH  HDSIQISWKA**P**RGKFGGYKLL  KFGGYKLLVT**P**TSGGKTNQLN  ATKAIIQGLM**P**DQNYTVQIIA  IAYNKDKESK**P**AQGQFRIKDL  RIKDLEKRKD**P**KPRVKVVDRG  KDLEKRKDPK**P**RVKVVDRGNG  KVVDRGNGSR**P**SSPEEVKFVC  DRGNGSRPSS**P**EEVKFVCQTP  PEEVKFVCQT**P**AIADIVILVD  RIGLAQYSGD**P**RIEWHLNAFS  DEVIEAVRNL**P**YKGGNTLTGL  LNYIFENSFK**P**EAGSRTGVSK  TDGKSQDDII**P**PSRNLRESGV  DGKSQDDIIP**P**SRNLRESGVE  VNELQEIASE**P**DSTHVYNVAE  IKASAHAITG**P**PTELITSEVT  KASAHAITGP**P**TELITSEVTA  RSFMVNWTHA**P**GNVEKYRVVY  NVEKYRVVYY**P**TRGGKPDEVV  VVYYPTRGGK**P**DEVVVDGTVS  LRGTETTLAL**P**MASDLLLYDV  NSMRVKWDAV**P**GASGYLILYA  GASGYLILYA**P**LTEGLAGDEK  HTDIELSGLL**P**NTEYTVTVYA  YAMFGEEASD**P**VTGQETTLAL  TGQETTLALS**P**PRNLRISNVG  GQETTLALSP**P**RNLRISNVGS  GSNSARLTWD**P**TSRQINGYRI  GTEINEVEVD**P**ITTFPLKGLT  EVEVDPITTF**P**LKGLTPLTEY  ITTFPLKGLT**P**LTEYTIAIFS  FSIYDEGQSE**P**LTGVFTTEEV  LTGVFTTEEV**P**AQQYLEIDEV  TTDSFRVTWH**P**LSADEGLHKL  DEGLHKLMWI**P**VYGGKTEEVV  QDSHVIEGLE**P**GTEYEVSLLA  GTTLDSFWTE**P**ATTIVPTTSV  FWTEPATTIV**P**TTSVTSVFQT  RVTYMTAQGD**P**EEEVIGTVMV  EEEVIGTVMV**P**GSQNNLLLKP  PGSQNNLLLK**P**LLPDTEYKVT  QNNLLLKPLL**P**DTEYKVTVTP  PDTEYKVTVT**P**IYTDGEGVSV  TDGEGVSVSA**P**GKTLPSSGPQ  VSVSAPGKTL**P**SSGPQNLRVS  APGKTLPSSG**P**QNLRVSEEWY  WYNRLRITWD**P**PSSPVKGYRI  YNRLRITWDP**P**SSPVKGYRIV  LRITWDPPSS**P**VKGYRIVYKP  PVKGYRIVYK**P**VSVPGPTLET  YRIVYKPVSV**P**GPTLETFVGA  IVYKPVSVPG**P**TLETFVGADI  TTRHCFYGLQ**P**DSEYKISVYT  VYTKLQEIEG**P**SVSIMEKTQS  VSIMEKTQSL**P**TRPPTFPPTI  MEKTQSLPTR**P**PTFPPTIPPA  EKTQSLPTRP**P**TFPPTIPPAK  QSLPTRPPTF**P**PTIPPAKEVC  SLPTRPPTFP**P**TIPPAKEVCK  TRPPTFPPTI**P**PAKEVCKAAK  RPPTFPPTIP**P**AKEVCKAAKA  QVAMVQFTDD**P**RTEFKLNAYK  TAESGTRRGI**P**KVIVVITDGR  YSELVSIGSK**P**SARHVFFVDD  FVCETASATC**P**VVHKDGIDLA  FSSVEGVSME**P**GTFNVFPCYQ  SMEPGTFNVF**P**CYQLHKDALV  QLHKDALVSQ**P**TRYLHPEGLP  LVSQPTRYLH**P**EGLPSDYTIS  PTRYLHPEGL**P**SDYTISFLFR  YTISFLFRIL**P**DTPQEPFALW  SFLFRILPDT**P**QEPFALWEIL  FRILPDTPQE**P**FALWEILNKN  LWEILNKNSD**P**LVGVILDNGG  GDFQTVTFEG**P**EIRKIFYGSF  VLGKMVRSRG**P**GGNSAPFQLQ  RSRGPGGNSA**P**FQLQMFDIVC  WANTDKCCEL**P**GLRDDESCPD  LPGLRDDESC**P**DLPHSCSCSE  LRDDESCPDL**P**HSCSCSETNE  CSETNEVALG**P**AGPPGGPGLR  TNEVALGPAG**P**PGGPGLRGPK  GPPGGPGLRG**P**KGQQGEPGPK  GPKGQQGEPG**P**KGPDGPRGEI  GQQGEPGPKG**P**DGPRGEIGLP  GEPGPKGPDG**P**RGEIGLPGPQ  GPRGEIGLPG**P**QGPPGPQGPS  GEIGLPGPQG**P**PGPQGPSGLS  GLPGPQGPPG**P**QGPSGLSIQG  GPQGPPGPQG**P**SGLSIQGMPG  GEKGDTGLPG**P**QGIPGGVGSP  GLPGKDGSSG**P**PGPPGPIGIP  GKDGSSGPPG**P**PGPIGIPGTP  GSSGPPGPPG**P**IGIPGTPGVP  GVPGITGSMG**P**QGALGPPGVP  GSMGPQGALG**P**PGVPGAKGER  HSSSIRTVQG**P**PGEPGRPGSP  GSPGAPGEQG**P**PGTPGFPGNA  KGNPGVGTQG**P**RGPPGPAGPS  PGVGTQGPRG**P**PGPAGPSGES  GTQGPRGPPG**P**AGPSGESRPG  GPRGPPGPAG**P**SGESRPGSPG  SGESRPGSPG**P**PGSPGPRGPP  GSPGPPGSPG**P**RGPPGHLGVP  GPPGSPGPRG**P**PGHLGVPGPQ  GPPGHLGVPG**P**QGPSGQPGYC  GHLGVPGPQG**P**SGQPGYCDPS  GPSGQPGYCD**P**SSCSAYGVRA  SSCSAYGVRA**P**HPDQPEFTPV  CSAYGVRAPH**P**DQPEFTPVQD  YGVRAPHPDQ**P**EFTPVQDELE  APHPDQPEFT**P**VQDELEAMEL  DELEAMELWG**P**GVVGPGWLEM  EGNQEERRDR**P**PSWIPKKPIW  ERRDRPPSWI**P**KKPIWSPPRD  EQSEEKKRFE**P**VPPGFTPFRQ  EEKKRFEPVP**P**GFTPFRQTTQ  RFEPVPPGFT**P**FRQTTQRFPT  LGAVLLLLAL**P**GHDQETTTQG  GHDQETTTQG**P**GVLLPLPKGA  TTTQGPGVLL**P**LPKGACTGWM  TQGPGVLLPL**P**KGACTGWMAG  APGRDGRDGT**P**GEKGEKGDPG  PKGDIGETGV**P**GAEGPRGFPG  FPGIQGRKGE**P**GEGAYVYRSA  SVGLETYVTI**P**NMPIRFTKIF  LETYVTIPNM**P**IRFTKIFYNQ  GSTGKFHCNI**P**GLYYFAYHIT  EVFYELAHQL**P**LPHNVSSHLD  FYELAHQLPL**P**HNVSSHLDKA  TGHSVFDFTH**P**CDHEEMREML  IHVYDTNSNQ**P**QCGYKKPPMT  SNQPQCGYKK**P**PMTCLVLICE  NQPQCGYKKP**P**MTCLVLICEP  PMTCLVLICE**P**IPHPSNIEIP  TCLVLICEPI**P**HPSNIEIPLD  LVLICEPIPH**P**SNIEIPLDSK  PIPHPSNIEI**P**LDSKTFLSRH  ERITELMGYE**P**EELLGRSIYE  TVIYNTKNSQ**P**QCIVCVNYVV  SLQQTECVLK**P**VESSDMKMTQ  SSLFDKLKKE**P**DALTLLAPAA  ETDDQQLEEV**P**LYNDVMLPSP  EVPLYNDVML**P**SPNEKLQNIN  PLYNDVMLPS**P**NEKLQNINLA  KLQNINLAMS**P**LPTAETPKPL  QNINLAMSPL**P**TAETPKPLRS  AMSPLPTAET**P**KPLRSSADPA  SPLPTAETPK**P**LRSSADPALN  TPKPLRSSAD**P**ALNQEVALKL  LNQEVALKLE**P**NPESLELSFT  QEVALKLEPN**P**ESLELSFTMP  PESLELSFTM**P**QIQDQTPSPS  FTMPQIQDQT**P**SPSDGSTRQS  MPQIQDQTPS**P**SDGSTRQSSP  PSDGSTRQSS**P**EPNSPSEYCF  DGSTRQSSPE**P**NSPSEYCFYV  TRQSSPEPNS**P**SEYCFYVDSD  LFAEDTEAKN**P**FSTQDTDLDL  LDLEMLAPYI**P**MDDDFQLRSF  FQLRSFDQLS**P**LESSSASPES  LSPLESSSAS**P**ESASPQSTVT  SSSASPESAS**P**QSTVTVFQQT  TVFQQTQIQE**P**TANATTTTAT  MEDIKILIAS**P**SPTHIHKETT  DIKILIASPS**P**THIHKETTSA  HKETTSATSS**P**YRDTQSRTAS  YRDTQSRTAS**P**NRAGKGVIEQ  GVIEQTEKSH**P**RSPNVLSVAL  EQTEKSHPRS**P**NVLSVALSQR  SVALSQRTTV**P**EEELNPKILA  RTTVPEEELN**P**KILALQNAQR  AVGIGTLLQQ**P**DDHAATTSLS  GMEQKTIILI**P**SDLACRLLGQ  LGQSMDESGL**P**QLTSYDCEVN  LTSYDCEVNA**P**IQGSRNLLQG  RASDILSHGV**P**YEANNRAVPD  SEQQLVDCSG**P**WGNNGCSGGL  ELKNLVGARR**P**AAVAVDVESD  SGIYQSQTCS**P**LRVNHAVLAV  MCGIASLASL**P**MVARFPPFRA  LASLPMVARF**P**PFRAVMPLSA  VPEHKTTKHE**P**VYKPVKTSYS  KTTKHEPVYK**P**VKTSYSAPYK  YKPVKTSYSA**P**YKPPTYQPLK  VKTSYSAPYK**P**PTYQPLKKKV  KTSYSAPYKP**P**TYQPLKKKVD  SAPYKPPTYQ**P**LKKKVDYRPT  QPLKKKVDYR**P**TKSYPPTYGS  KVDYRPTKSY**P**PTYGSKTNYL  VDYRPTKSYP**P**TYGSKTNYLP  PTYGSKTNYL**P**LAKKLSSYKP  PLAKKLSSYK**P**IKTTYNAKTN  KTTYNAKTNY**P**PVYKPKMTYP  TTYNAKTNYP**P**VYKPKMTYPP  AKTNYPPVYK**P**KMTYPPTYKP  PPVYKPKMTY**P**PTYKPKPSYP  PVYKPKMTYP**P**TYKPKPSYPP  PKMTYPPTYK**P**KPSYPPTYKS  PPTYKPKPSY**P**PTYKSKPTYK  PSYPPTYKSK**P**TYKPKITYPP  PTYKSKPTYK**P**KITYPPTYKA  KPTYKPKITY**P**PTYKAKPSYP  PTYKPKITYP**P**TYKAKPSYPS  PPTYKAKPSY**P**SSYKPKKTYP  AKPSYPSSYK**P**KKTYPPTYKP  PSSYKPKKTY**P**PTYKPKLTYP  SSYKPKKTYP**P**TYKPKLTYPP  PKKTYPPTYK**P**KLTYPPTYKP  PPTYKPKLTY**P**PTYKPKPSYP  PTYKPKLTYP**P**TYKPKPSYPP  PKLTYPPTYK**P**KPSYPPTYKP  PPTYKPKPSY**P**PTYKPKPSYP  PKPSYPPTYK**P**KPSYPPSYKT  PPTYKPKPSY**P**PSYKTKKTYP  PPSYKTKKTY**P**SSYKAKPSYP  PSSYKAKPSY**P**PTYKAKPSYP  PPTYKAKPSY**P**PTYKAKPSYP  PPTYKAKPSY**P**PTYKAKPTYK  PSYPPTYKAK**P**TYKAKPTYPS  KPTYKAKPTY**P**STYKAKPSYP  PSTYKAKPSY**P**PTYKAKPTYK  PSYPPTYKAK**P**TYKAKPSYPP  KPTYKAKPSY**P**PTYKAKPSYP  PSYPPTYKAK**P**TYKAKPTYKA  YKAKPTYKAK**P**TYKAKPTYKA  YKAKPTYKAK**P**TYKAKPSYPP  PSTYKAKPSY**P**PTYKAKPSYP  PSTYKAKPSY**P**PSYKAKPSYP  PPSYKAKPSY**P**PTYKAKPTYK  PSTYKAKPSY**P**ASYKAKPSYP  PASYKAKPSY**P**PTYKSKSSYP  PPTYKSKSSY**P**SSYKPKKTYP  SKSSYPSSYK**P**KKTYPPTYKP  PSSYKPKKTY**P**PTYKPKLTYK  SSYKPKKTYP**P**TYKPKLTYKP  PKKTYPPTYK**P**KLTYKPTYKP  PTYKPKLTYK**P**TYKPKPSYPP  PKLTYKPTYK**P**KPSYPPSYKP  KPTYKPKPSY**P**PSYKPKTTYP  PKPSYPPSYK**P**KTTYPPTYKP  PPSYKPKTTY**P**PTYKPKISYP  PSYKPKTTYP**P**TYKPKISYPP  PKTTYPPTYK**P**KISYPPTYKA  PPTYKPKISY**P**PTYKAKPSYP  PPTYKAKPSY**P**ATYKAKPSYP  PATYKAKPSY**P**PTYKAKPSYP  PPTYKAKPSY**P**PTYKAKPSYK  PSYPPTYKAK**P**SYKAKPTYPS  KPSYKAKPTY**P**STYKAKPSYP  PPTYKAKPSY**P**PTYKAKPTYP  PPTYKAKPTY**P**STYKAKPSYP  PSTYKAKPSY**P**PTYKPKISYP  AKPSYPPTYK**P**KISYPPTYKA  PSYPPTYKAK**P**TYKAKPTNPS  YKAKPTYKAK**P**TNPSTYKAKP  KPTYKAKPTN**P**STYKAKPSYP  KPTYKAKPTY**P**STYKAKPTYK  PTYPSTYKAK**P**TYKAKPTYPP  KPTYKAKPTY**P**PTYKAKPSYP  PPTYKAKPSY**P**PTYKPKPSYP  AKPSYPPTYK**P**KPSYPPTYKS  PPTYKPKPSY**P**PTYKSKSIYP  PPTYKSKSIY**P**SSYKPKKTYP  SKSIYPSSYK**P**KKTYPPTYKP  PKLTYPPTYK**P**KPSYPPSYKP  PPTYKPKPSY**P**PSYKPKITYP  PKPSYPPSYK**P**KITYPSTYKL  PPSYKPKITY**P**STYKLKPSYP  PSTYKLKPSY**P**PTYKSKTSYP  PPTYKSKTSY**P**PTYNKKISYP  PTYKSKTSYP**P**TYNKKISYPS  PPTYNKKISY**P**SSYKAKTSYP  PSSYKAKTSY**P**PAYKPTNRYY  AKTSYPPAYK**P**TNRYYRNTPK  VLLFTFLVLF**P**LTTLELDTDR  LTTLELDTDR**P**VERHAAIKQD  RHAAIKQDLK**P**QERRGIRLHA  QERRGIRLHA**P**RDECCEPQWC  QGALLLLLAL**P**SHGEDNMEDP  PSHGEDNMED**P**PLPKGACAGW  SHGEDNMEDP**P**LPKGACAGWM  GEDNMEDPPL**P**KGACAGWMAG  TPGRDGRDGT**P**GEKGEKGDPG  TPGEKGEKGD**P**GLVGPKGDTG  GEKGDPGLVG**P**KGDTGETGIT  ITGIEGPRGF**P**GTPGRKGEPG  IEGPRGFPGT**P**GRKGEPGESA  FPGTPGRKGE**P**GESAYVYRSA  SVGLERQVTV**P**NVPIRFTKIF  LERQVTVPNV**P**IRFTKIFYNQ  GTTGKFLCNI**P**GLYYFSYHIT  RVEAKGIKGD**P**GSRGSPGKHG  IKGDPGSRGS**P**GKHGPKGSIG  GSRGSPGKHG**P**KGSIGPTGEQ  GKHGPKGSIG**P**TGEQGLPGET  GEQGLPGETG**P**QGQKGDKGEV  GQKGDKGEVG**P**TGPEGLMGST  GDKGEVGPTG**P**EGLMGSTGPL  GPEGLMGSTG**P**LGPKGLPGPM  GLMGSTGPLG**P**KGLPGPMGPI  GPLGPKGLPG**P**MGPIGKPGPR  GPKGLPGPMG**P**IGKPGPRGEA  GPMGPIGKPG**P**RGEAGPMGPQ  GKPGPRGEAG**P**MGPQGEPGVR  GPRGEAGPMG**P**QGEPGVRGMR  RGEKGKVGEA**P**LVPKSAFTVG  KGKVGEAPLV**P**KSAFTVGLTV  TVGLTVISKF**P**PPDAPIKFDK  VGLTVISKFP**P**PDAPIKFDKI  GLTVISKFPP**P**DAPIKFDKIL  VISKFPPPDA**P**IKFDKILYNE  LQALLFLLIL**P**SHAEDDVTTT  DDVTTTEELA**P**ALVPPPKGTC  TTEELAPALV**P**PPKGTCAGWM  TEELAPALVP**P**PKGTCAGWMA  EELAPALVPP**P**KGTCAGWMAG  TPGRDGRDGT**P**GEKGEKGDAG  GEKGDAGLLG**P**KGETGDVGMT  MTGAEGPRGF**P**GTPGRKGEPG  AEGPRGFPGT**P**GRKGEPGEAA  FPGTPGRKGE**P**GEAAYVYRSA  SVGLETRVTV**P**NVPIRFTKIF  LETRVTVPNV**P**IRFTKIFYNQ  GSTGKFYCNI**P**GLYYFSYHIT  QSQLERANLR**P**CEQHLMQKIQ  QRDEDSYGRD**P**YSPSQDPYSP  GRDPYSPSQD**P**YSPSQDPDRR  SQDPYSPSQD**P**DRRDPYSPSP  SPSQDPDRRD**P**YSPSPYDRRG  PDRRDPYSPS**P**YDRRGAGSSQ  QQFKRELRNL**P**QQCGLRAPQR  NLPQQCGLRA**P**QRCDLEVESG  IVNGRHDSVL**P**PPSPKTDPII  VLPPPSPKTD**P**IIGQLTTITT  IGQLTTITTT**P**HHDDTVAAPP  TPHHDDTVAA**P**PVGGRHDYVA  PHHDDTVAAP**P**VGGRHDYVAS  DYVASPPPPK**P**QDEQRQIIIT  IIITSSSSTL**P**LQASYYSAQL  ICAPSTNWIL**P**GCSTSTFTTF  ICKPSTNVIL**P**GCSTSSFFRI  ICAPSTNWIL**P**GCSTSSFFKI  EESEEKKREA**P**ERPPGFTPFR  EEKKREAPER**P**PGFTPFRIYY  EAPERPPGFT**P**FRIYYIRFPT  PAPSPTTTVT**P**PPVATPPPAA  TTVTPPPVAT**P**PPAATPAPTT  TVTPPPVATP**P**PAATPAPTTT  VTPPPVATPP**P**AATPAPTTTP  PVATPPPAAT**P**APTTTPPPAV  ATPPPAATPA**P**TTTPPPAVSP  PAATPAPTTT**P**PPAVSPAPTS  AATPAPTTTP**P**PAVSPAPTSS  ATPAPTTTPP**P**AVSPAPTSSP  PTTTPPPAVS**P**APTSSPPSSA  TTPPPAVSPA**P**TSSPPSSAPS  PAVSPAPTSS**P**PSSAPSPSSD  AVSPAPTSSP**P**SSAPSPSSDA  APTSSPPSSA**P**SPSSDAPTAS  TSSPPSSAPS**P**SSDAPTASPP  SSAPSPSSDA**P**TASPPAPEGP  SPSSDAPTAS**P**PAPEGPGVSP  PSSDAPTASP**P**APEGPGVSPG  SDAPTASPPA**P**EGPGVSPGEL  PTASPPAPEG**P**GVSPGELAPT  PPAPEGPGVS**P**GELAPTPSDA  GPGVSPGELA**P**TPSDASAPPP  GVSPGELAPT**P**SDASAPPPNA  LAPTPSDASA**P**PPNAALTNKA  APTPSDASAP**P**PNAALTNKAF  PTPSDASAPP**P**NAALTNKAFV  ALFLIYLILS**P**FRAVSRTLLE  GSGYGRGTNL**P**PPSPASSPPS  PSKEVSNSVS**P**TRTDEKTSEN  AQGENSNQLF**P**FLTSSDNYQL  FKKLSISYLL**P**VSYVWKLISS  SSDEKYWNRK**P**LSPPSPKPAD  RKPLSPPSPK**P**ADGHRPLQSY  PSPKPADGHR**P**LQSYYSQLPR  VLFLIYLILS**P**FGAEARTLLE  GSGYGRGANL**P**PPSPASSPPS  GENINQLFSF**P**TSADNYYQLA  FKKLFISYLL**P**VSYVWNLIGS  KSDERYWNRK**P**LSPPSPKPAD  RKPLSPPSPK**P**ADGQRPLHSY  PSPKPADGQR**P**LHSYYSHLPR  LTITSSLARN**P**VSVSGGFENS  LMVNVEDYGD**P**SANPKHDPGV  VEDYGDPSAN**P**KHDPGVPPSA  GDPSANPKHD**P**GVPPSATGQR  STQALFQEKR**P**MKKIDFLSKG  STQAVLQEKR**P**KEKIKFLSKR  DARTKTDDDV**P**LSPLRDNLKR  TKTDDDVPLS**P**LRDNLKRTIR  ALLIICLLLF**P**LTAVPMDGDQ  CLLLFPLTAV**P**MDGDQPADRP  LTAVPMDGDQ**P**ADRPAERMQD  PMDGDQPADR**P**AERMQDDISF  MQDDISFEQH**P**MFDATRRCCN  TSPPTATPAP**P**TPTTPPPAAT  PPTATPAPPT**P**TTPPPAATPP  ATPAPPTPTT**P**PPAATPPPVS  TPAPPTPTTP**P**PAATPPPVSA  PAPPTPTTPP**P**AATPPPVSAP  TPTTPPPAAT**P**PPVSAPPPVT  PTTPPPAATP**P**PVSAPPPVTT  TTPPPAATPP**P**VSAPPPVTTS  PAATPPPVSA**P**PPVTTSPPPV  AATPPPVSAP**P**PVTTSPPPVT  ATPPPVSAPP**P**VTTSPPPVTT  VSAPPPVTTS**P**PPVTTAPPPA  SAPPPVTTSP**P**PVTTAPPPAN  APPPVTTSPP**P**VTTAPPPANP  TTSPPPVTTA**P**PPANPPPPVS  TSPPPVTTAP**P**PANPPPPVSS  SPPPVTTAPP**P**ANPPPPVSSP  PVTTAPPPAN**P**PPPVSSPPPA  VTTAPPPANP**P**PPVSSPPPAS  TTAPPPANPP**P**PVSSPPPASP  TAPPPANPPP**P**VSSPPPASPP  PANPPPPVSS**P**PPASPPPATP  ANPPPPVSSP**P**PASPPPATPP  NPPPPVSSPP**P**ASPPPATPPP  PPVSSPPPAS**P**PPATPPPVAS  PVSSPPPASP**P**PATPPPVASP  VSSPPPASPP**P**ATPPPVASPP  PPPASPPPAT**P**PPVASPPPPV  PPASPPPATP**P**PVASPPPPVA  PASPPPATPP**P**VASPPPPVAS  PPATPPPVAS**P**PPPVASPPPA  PATPPPVASP**P**PPVASPPPAT  ATPPPVASPP**P**PVASPPPATP  TPPPVASPPP**P**VASPPPATPP  VASPPPPVAS**P**PPATPPPVAT  ASPPPPVASP**P**PATPPPVATP  SPPPPVASPP**P**ATPPPVATPP  PPVASPPPAT**P**PPVATPPPAP  PVASPPPATP**P**PVATPPPAPL  VASPPPATPP**P**VATPPPAPLA  PPATPPPVAT**P**PPAPLASPPA  PATPPPVATP**P**PAPLASPPAQ  ATPPPVATPP**P**APLASPPAQV  PPPVATPPPA**P**LASPPAQVPA  ATPPPAPLAS**P**PAQVPAPAPT  TPPPAPLASP**P**AQVPAPAPTT  APLASPPAQV**P**APAPTTKPDS  LASPPAQVPA**P**APTTKPDSPS  SPPAQVPAPA**P**TTKPDSPSPS  QVPAPAPTTK**P**DSPSPSPSSS  APAPTTKPDS**P**SPSPSSSPPL  APTTKPDSPS**P**SPSSSPPLPS  TTKPDSPSPS**P**SSSPPLPSSD  DSPSPSPSSS**P**PLPSSDAPGP  SPSPSPSSSP**P**LPSSDAPGPS  SPSPSSSPPL**P**SSDAPGPSTD  SSPPLPSSDA**P**GPSTDSISPA  PPLPSSDAPG**P**STDSISPAPS  APGPSTDSIS**P**APSPTDVNDQ  GPSTDSISPA**P**SPTDVNDQNG  STDSISPAPS**P**TDVNDQNGAS  SPTSDAAMFV**P**ALFASVAALA  SPTSDASSFI**P**TFFASVAVMA  PGPAPTRSPL**P**SPAQPPRTAA  TRSPLPSPAQ**P**PRTAAPTPSI  RSPLPSPAQP**P**RTAAPTPSIT  SPAQPPRTAA**P**TPSITPTPTP  AQPPRTAAPT**P**SITPTPTPTP  RTAAPTPSIT**P**TPTPTPSATP  AAPTPSITPT**P**TPTPSATPTA  PTPSITPTPT**P**TPSATPTAAP  PSITPTPTPT**P**SATPTAAPVS  PTPTPTPSAT**P**TAAPVSPPAG  PTPSATPTAA**P**VSPPAGSPLP  SATPTAAPVS**P**PAGSPLPSSA  ATPTAAPVSP**P**AGSPLPSSAS  AAPVSPPAGS**P**LPSSASPPAP  PVSPPAGSPL**P**SSASPPAPPT  AGSPLPSSAS**P**PAPPTSLTPD  GSPLPSSASP**P**APPTSLTPDG  PLPSSASPPA**P**PTSLTPDGAP  LPSSASPPAP**P**TSLTPDGAPV  SPPAPPTSLT**P**DGAPVAGPTG  PPTSLTPDGA**P**VAGPTGSTPV  LTPDGAPVAG**P**TGSTPVDNNN  APVAGPTGST**P**VDNNNAATLA  APAPAPTTVT**P**PPTALPPVTA  TTVTPPPTAL**P**PVTAETPSPI  TVTPPPTALP**P**VTAETPSPIA  TALPPVTAET**P**SPIASPPVPV  LPPVTAETPS**P**IASPPVPVNE  TAETPSPIAS**P**PVPVNEPTPA  AETPSPIASP**P**VPVNEPTPAP  TPSPIASPPV**P**VNEPTPAPTT  IASPPVPVNE**P**TPAPTTSPTT  SPPVPVNEPT**P**APTTSPTTSP  PVPVNEPTPA**P**TTSPTTSPVA  NEPTPAPTTS**P**TTSPVASPPQ  PAPTTSPTTS**P**VASPPQTDAP  TSPTTSPVAS**P**PQTDAPAPGP  SPTTSPVASP**P**QTDAPAPGPS  PVASPPQTDA**P**APGPSAGLTP  ASPPQTDAPA**P**GPSAGLTPTS  PPQTDAPAPG**P**SAGLTPTSSP  PAPGPSAGLT**P**TSSPAPGPDG  PSAGLTPTSS**P**APGPDGAADA  AGLTPTSSPA**P**GPDGAADAPS  LTPTSSPAPG**P**DGAADAPSAA  APGPDGAADA**P**SAAWANKAFL  SPTSDASLAI**P**AFFASVATLA  LVIVFLNLVV**P**TSACRAEGTY  WGGCGHPCRH**P**GKRSKLQEFF  GSYMMYSGAG**P**ALAPPAPPPP  MYSGAGPALA**P**PAPPPPIQGY  YSGAGPALAP**P**APPPPIQGYA  GAGPALAPPA**P**PPPIQGYAFK  AGPALAPPAP**P**PPIQGYAFKP  GPALAPPAPP**P**PIQGYAFKPP  PALAPPAPPP**P**IQGYAFKPPP  PPPIQGYAFK**P**PPRPDFGTSG  PPIQGYAFKP**P**PRPDFGTSGR  PIQGYAFKPP**P**RPDFGTSGRT  QGYAFKPPPR**P**DFGTSGRTIK  LQANFFEMDI**P**KIDIYHYELD  DIYHYELDIK**P**EKCPRRVNRE  YELDIKPEKC**P**RRVNREIVEH  FKTQIFGDRK**P**VFDGRKNLYT  DGRKNLYTAM**P**LPIGRDKVEL  RKNLYTAMPL**P**IGRDKVELEV  RDKVELEVTL**P**GEGKDRIFKV  ALHDALSGRL**P**SVPFETIQAL  DALSGRLPSV**P**FETIQALDVV  QALDVVMRHL**P**SMRYTPVGRS  MRHLPSMRYT**P**VGRSFFTASE  FFTASEGCSN**P**LGGGREVWFG  VWFGFHQSVR**P**SLWKMMLNID  VSATAFYKAQ**P**VIEFVCEVLD  DFKSIEEQQK**P**LTDSQRVKFT  KYRVCNVTRR**P**ASHQTFPLQQ  TRRPASHQTF**P**LQQESGQTVE  KDRHKLVLRY**P**HLPCLQVGQE  HKLVLRYPHL**P**CLQVGQEQKH  VGQEQKHTYL**P**LEVCNIVAGQ  TMIRATARSA**P**DRQEEISKLM  LMRSASFNTD**P**YVREFGIMVK  MTDVTGRVLQ**P**PSILYGGRNK  TDVTGRVLQP**P**SILYGGRNKA  YGGRNKAIAT**P**VQGVWDMRNK  IKVWAIACFA**P**QRQCTEVHLK  LRKISRDAGM**P**IQGQPCFCKY  RDAGMPIQGQ**P**CFCKYAQGAD  KYAQGADSVE**P**MFRHLKNTYA  AGLQLVVVIL**P**GKTPVYAEVK  LVVVILPGKT**P**VYAEVKRVGD  VQMKNVQRTT**P**QTLSNLCLKI  KLGGVNNILL**P**QGRPPVFQQP  VNNILLPQGR**P**PVFQQPVIFL  NNILLPQGRP**P**VFQQPVIFLG  PQGRPPVFQQ**P**VIFLGADVTH  VIFLGADVTH**P**PAGDGKKPSI  IFLGADVTHP**P**AGDGKKPSIA  THPPAGDGKK**P**SIAAVVGSMD  AAVVGSMDAH**P**NRYCATVRVQ  IQFYKSTRFK**P**TRIIFYRDGV  NERVGKSGNI**P**AGTTVDTKIT  GTTVDTKITH**P**TEFDFYLCSH  SHAGIQGTSR**P**SHYHVLWDDN  YVRCTRSVSI**P**APAYYAHLVA  RCTRSVSIPA**P**AYYAHLVAFR  EVLYQLAHTL**P**FARGVSAHLD  EWNQVGAGGE**P**LDACYLKALE  IGHSIFDFIH**P**CDQEELQDAL  DQEELQDALT**P**QQTLSRRKVE  QTLSRRKVEA**P**TERCFSLRMK  NCSGHMRAYK**P**PAQTSPAGSP  CSGHMRAYKP**P**AQTSPAGSPD  RAYKPPAQTS**P**AGSPDSEPPL  PPAQTSPAGS**P**DSEPPLQCLV  TSPAGSPDSE**P**PLQCLVLICE  SPAGSPDSEP**P**LQCLVLICEA  QCLVLICEAI**P**HPGSLEPPLG  LVLICEAIPH**P**GSLEPPLGRG  EAIPHPGSLE**P**PLGRGAFLSR  AIPHPGSLEP**P**LGRGAFLSRH  DRIAEVAGYS**P**DDLIGCSAYE  QATVVSGGRG**P**QSESIVCVHF  LEQTEQHSRR**P**IQRGAPSQKD  HSRRPIQRGA**P**SQKDTPNPGD  QRGAPSQKDT**P**NPGDSLDTPG  GAPSQKDTPN**P**GDSLDTPGPR  TPNPGDSLDT**P**GPRILAFLHP  NPGDSLDTPG**P**RILAFLHPPS  PGPRILAFLH**P**PSLSEAALAA  GPRILAFLHP**P**SLSEAALAAD  SLSEAALAAD**P**RRFCSPDLRR  LAADPRRFCS**P**DLRRLLGPIL  CSPDLRRLLG**P**ILDGASVAAT  ILDGASVAAT**P**STPLATRHPQ  GASVAATPST**P**LATRHPQSPL  TPSTPLATRH**P**QSPLSADLPD  TPLATRHPQS**P**LSADLPDELP  HPQSPLSADL**P**DELPVGTENV  PLSADLPDEL**P**VGTENVHRLF  DFQLNASEQL**P**RAYHRPLGAV  SEQLPRAYHR**P**LGAVPRPRAR  RAYHRPLGAV**P**RPRARSFHGL  YHRPLGAVPR**P**RARSFHGLSP  PRARSFHGLS**P**PALEPSLLPR  RARSFHGLSP**P**ALEPSLLPRW  FHGLSPPALE**P**SLLPRWGSDP  SPPALEPSLL**P**RWGSDPRLSC  PSLLPRWGSD**P**RLSCSSPSRG  GSDPRLSCSS**P**SRGDPSASSP  LSCSSPSRGD**P**SASSPMAGAR  PSRGDPSASS**P**MAGARKRTLA  DEGVELLGVR**P**PKRSPSPEHE  EGVELLGVRP**P**KRSPSPEHEN  LLGVRPPKRS**P**SPEHENFLLF  GVRPPKRSPS**P**EHENFLLFPL  SPEHENFLLF**P**LSLSFLLTGG  LSLSFLLTGG**P**APGSLQDPST  LSFLLTGGPA**P**GSLQDPSTPL  GGPAPGSLQD**P**STPLLNLNEP  APGSLQDPST**P**LLNLNEPLGL  PSTPLLNLNE**P**LGLGPSLLSP  LNLNEPLGLG**P**SLLSPYSDED  PLGLGPSLLS**P**YSDEDTTQPG  SPYSDEDTTQ**P**GGPFQPRAGS  SDEDTTQPGG**P**FQPRAGSAQA  DTTQPGGPFQ**P**RAGSAQADDA  PPATPPPVAT**P**PPVATPPPAA  PATPPPVATP**P**PVATPPPAAT  ATPPPVATPP**P**VATPPPAATP  PVATPPPVAT**P**PPAATPAPAT  VATPPPVATP**P**PAATPAPATP  ATPPPVATPP**P**AATPAPATPP  PVATPPPAAT**P**APATPPPAAT  ATPPPAATPA**P**ATPPPAATPA  PPAATPAPAT**P**PPAATPAPAT  PAATPAPATP**P**PAATPAPATT  AATPAPATPP**P**AATPAPATTP  APATPPPAAT**P**APATTPPSVA  ATPPPAATPA**P**ATTPPSVAPS  PAATPAPATT**P**PSVAPSPADV  AATPAPATTP**P**SVAPSPADVP  APATTPPSVA**P**SPADVPTASP  ATTPPSVAPS**P**ADVPTASPPA  PSVAPSPADV**P**TASPPAPEGP  PSPADVPTAS**P**PAPEGPTVSP  SPADVPTASP**P**APEGPTVSPS  ADVPTASPPA**P**EGPTVSPSSA  PTASPPAPEG**P**TVSPSSAPGP  PPAPEGPTVS**P**SSAPGPSDAS  EGPTVSPSSA**P**GPSDASPAPS  PTVSPSSAPG**P**SDASPAPSAA  SSAPGPSDAS**P**APSAAFSNKA  APGPSDASPA**P**SAAFSNKAFF |
