# Supplementary material for: iHyd-PseCp: Identify hydroxyproline and hydroxylysine in proteins by incorporating sequence-coupled effects into general PseAAC
Source: Oncotarget. 2016 Jun 14;7(28):44310–21. doi: 10.18632/oncotarget.10027 (PMC5190098; doi:10.18632/oncotarget.10027)
Supplement: Supplementary file 3 [file oncotarget-07-44310-s003.docx]

**Supporting Information S2.** The benchmark dataset $\mathbb{S}(K)$used to train and test the model for predicting the possibility of hydroxylation at Lys site. It contains 142 positive samples and 980 negative samples, which were extracted from the 33 hydroxylysine proteins. See the main text for further explanation.

(1) List of the 142 peptide samples in the positive subset $\mathbb{S}^{+}$(K)

| Sample # | Protein ID | Site | Sequences |
| --- | --- | --- | --- |
| 1  2  3  4  5  6  7  8  9  10  11  12  13  14  15  16  17  18  19  20  21  22  23  24  25  26  27  28  29  30  31  32  33  34  35  36  37  38  39  40  41  42  43  44  45  46  47  48  49  50  51  52  53  54  55  56  57  58  59  60  61  62  63  64  65  66  67  68  69  70  71  72  73  74  75  76  77  78  79  80  81  82  83  84  85  86  87  88  89  90  91  92  93  94  95  96  97  98  99  100  101  102  103  104  105  106  107  108  109  110  111  112  113  114  115  116  117  118  119  120  121  122  123  124  125  126  127  128  129  130  131  132  133  134  135  136  137  138  139  140  141  142 | O18495  O18495  O18495  O18495  O18495  O18495  P01170  P02452  P02452  P02453  P02453  P02453  P02453  P02453  P02453  P02453  P02454  P02454  P02457  P02457  P02457  P02459  P02459  P02459  P02459  P02459  P02459  P02459  P02459  P02459  P02459  P02459  P02459  P02459  P02459  P02461  P02461  P02461  P02461  P02461  P02461  P02745  P02745  P02745  P02745  P02747  P04142  P04258  P04258  P04258  P04258  P04258  P12108  P12111  P12111  P12111  P12111  P12111  P19999  P19999  P19999  P19999  P20908  P20908  P20908  P20908  P20908  P20908  P20908  P20908  P20908  P20908  P20908  P20908  P20908  P20908  P23805  P23805  P23805  P23805  P23805  P23805  P23805  P23805  P23805  P26368  P26368  P30754  P30754  P30754  P30754  P30754  P30754  P30754  P30754  P30754  P30754  P30754  P30754  P30754  P30754  P30754  P30754  P30754  P30754  P30754  P30754  P30943  P35248  P35248  P83414  P83415  P84698  P84698  Q00433  Q02388  Q02388  Q05707  Q05707  Q05707  Q05707  Q05707  Q05707  Q05707  Q15848  Q15848  Q15848  Q15848  Q15848  Q3Y5Z3  Q3Y5Z3  Q3Y5Z3  Q3Y5Z3  Q3Y5Z3  Q4ZJN1  Q4ZJN1  Q60994  Q60994  Q60994  Q60994  Q9Y383  Q9Y383 | 40  44  27  30  34  38  120  265  1108  276  285  708  780  861  933  1095  575  698  419  494  740  190  287  299  308  374  419  452  464  470  527  542  608  620  1130  263  284  860  977  1094  1106  33  48  67  100  75  47  95  107  119  938  950  181  2103  2209  2212  2322  2337  44  47  79  82  627  642  708  744  774  795  804  807  810  819  846  864  882  897  63  87  99  135  141  159  162  198  210  15  276  96  108  183  192  261  279  342  351  546  567  573  612  657  738  765  810  927  933  936  939  41  86  98  21  21  18  25  56  2625  2631  1476  1485  1523  1526  1601  1698  1701  33  65  68  77  101  28  60  63  72  96  73  127  68  71  80  104  266  269 | KSVGKFYYKH**K**YYIKAAWQIG  KFYYKHKYYI**K**AAWQIGKHAL  IQQSEAGWLR**K**AAKSVGKFYY  SEAGWLRKAA**K**SVGKFYYKHK  WLRKAAKSVG**K**FYYKHKYYIK  AAKSVGKFYY**K**HKYYIKAAWQ  RKAGCKNFYW**K**GFTSCCSTFG  LPGTAGLPGM**K**GHRGFSGLDG  ETGEQGDRGI**K**GHRGFSGLQG  HRGFSGLDGA**K**GDAGPAGPKG  AKGDAGPAGP**K**GEPGSPGENG  ANGAPGNDGA**K**GDAGAPGAPG  PPGPAGAPGD**K**GEAGPSGPAG  PIGNVGAPGP**K**GARGSAGPPG  PPGPPGPAGE**K**GAPGADGPAG  PAGPQGPRGD**K**GETGEQGDRG  QAGVMGFPGP**K**GTAGEPGKAG  NNGAPGNDGA**K**GDTGAPGAPG  PQGPSGAPGP**K**GNSGEPGAPG  FPGADGIAGP**K**GPPGERGSPG  AKGDRGDPGP**K**GADGAPGKDG  AAQMAGGFDE**K**AGGAQMGVMQ  FPGTPGLPGV**K**GHRGYPGLDG  HRGYPGLDGA**K**GEAGAPGVKG  AKGEAGAPGV**K**GESGSPGENG  GPGFPGAPGA**K**GEAGPTGARG  NPGTDGIPGA**K**GSAGAPGIAG  PQGATGPLGP**K**GQTGEPGIAG  QTGEPGIAGF**K**GEQGPKGEPG  IAGFKGEQGP**K**GEPGPAGPQG  FPGQDGLAGP**K**GAPGERGPSG  ERGPSGLAGP**K**GANGDPGRPG  QPGVMGFPGP**K**GANGEPGKAG  ANGEPGKAGE**K**GLPGAPGLRG  ETGEAGERGL**K**GHRGFTGLQG  PAGIPGFPGM**K**GHRGFDGRNG  EKGETGAPGL**K**GENGLPGENG  PAGPPGPQGV**K**GERGSPGGPG  PRGSPGPQGV**K**GESGKPGANG  APGPQGPRGD**K**GETGERGAAG  ETGERGAAGI**K**GHRGFPGNPG  EDLCRAPDGK**K**GEAGRPGRRG  RPGRRGRPGL**K**GEQGEPGAPG  PGIRTGIQGL**K**GDQGEPGPSG  PLGARGIPGI**K**GTKGSPGNIK  IPGIRGPKGQ**K**GEPGLPGHPG  MGRNIRDGIV**K**AGPAIEVLGS  PRGFPGPPGM**K**GPAGMPGFPG  PAGMPGFPGM**K**GHRGFDGRNG  HRGFDGRNGE**K**GEPGAPGLKG  PPGPQGPRGD**K**GETGERGAMG  ETGERGAMGI**K**GHRGFPGNPG  LCPTNCPPGP**K**GPQGLQGLKG  VKGSRGFPGE**K**GEVGEIGLDG  GFGRRGPPGA**K**GNKGGPGQPG  RRGPPGAKGN**K**GGPGQPGFEG  ERGFPGYPGP**K**GNPGEPGLNG  EPGLNGTTGP**K**GIRGRRGNSG  ACGRDGRDGP**K**GEKGEPGQGL  RDGRDGPKGE**K**GEPGQGLRGL  SVGAPGSQGP**K**GQKGDRGDSR  APGSQGPKGQ**K**GDRGDSRAIE  ARGMPGQTGP**K**GDRGFDGLAG  FDGLAGLPGE**K**GHRGDPGPSG  VTGMDGQPGP**K**GNVGPQGEPG  PQGAIGPPGE**K**GPLGKPGLPG  HPGKEGPPGE**K**GGQGPPGPQG  PIGYPGPRGV**K**GADGIRGLKG  VKGADGIRGL**K**GTKGEKGEDG  ADGIRGLKGT**K**GEKGEDGFPG  IRGLKGTKGE**K**GEDGFPGFKG  EKGEDGFPGF**K**GDMGIKGDRG  PRGEDGPEGP**K**GRGGPNGDPG  DPGPLGPPGE**K**GKLGVPGLPG  LPGYPGRQGP**K**GSIGFPGFPG  FPGFPGANGE**K**GGRGTPGKPG  QDGRECPHGE**K**GDPGSPGPAG  RPGWVGPIGP**K**GDNGFVGEPG  DNGFVGEPGP**K**GDTGPRGPPG  SMGPPGTPGP**K**GETGPKGGVG  TPGPKGETGP**K**GGVGAPGIQG  IQGFPGPSGL**K**GEKGAPGETG  FPGPSGLKGE**K**GAPGETGAPG  PSGARGPPGL**K**GDRGDPGETG  DRGDPGETGA**K**GESGLAEVNA  DEFERQLNEN**K**QERDKENRHR  LPNYLNDDQV**K**ELLTSFGPLK  AGGMPGMPGP**K**GHRGFRGLSG  HRGFRGLSGS**K**GEQGKSGNQG  SPGFPGTPGS**K**GDRGQSGIKG  SKGDRGQSGI**K**GAQGLQGPVG  TAGSPGQAGA**K**GDGGPTGEQG  EQGRPGAPGV**K**GSSGPPGDVG  ASGAMGIPGE**K**GPSGEPGAKG  EKGPSGEPGA**K**GPTGDTGRQG  PTGSPGVAGA**K**GQGGPPGPAG  LVGLPGERGP**K**GVGGSKGSRG  ERGPKGVGGS**K**GSRGDIGPRG  LPGPSGLAAS**K**GERGDMGSPG  PPGDAGPAGT**K**GDIGFPGERG  PAGTAGKQGV**K**GARGSPGLVG  SDGEPGRDGT**K**GERGEDGPPG  ETGPMGGQGM**K**GDGGPPGPSG  SQGPAGIMGM**K**GEAGGKGAKG  IMGMKGEAGG**K**GAKGDKGWTG  MKGEAGGKGA**K**GDKGWTGLPG  EAGGKGAKGD**K**GWTGLPGLQG  GATSVNMAMD**K**SAKAPVITIF  LPGPRGPVGP**K**GENGSAGEPG  ENGSAGEPGP**K**GERGLVGPPG  VGRNIRDGIV**K**AGPAIAVLGQ  MGRNIRDGVI**K**AAPAIEVLGQ  TTCANGWECC**K**GYPCVNKACS  ECCKGYPCVN**K**ACSGCTHGKK  SGAPAGAAMD**K**SAKAPQITIF  DVGFMGPRGL**K**GERGVKGACG  PRGLKGERGV**K**GACGLDGEKG  PPGGPGLRGP**K**GQQGEPGPKG  PKGQQGEPGP**K**GPDGPRGEIG  IQGMPGMPGE**K**GEKGDTGLPG  MPGMPGEKGE**K**GDTGLPGPQG  ALGPPGVPGA**K**GERGERGDLQ  TPGERGLTGI**K**GEKGNPGVGT  ERGLTGIKGE**K**GNPGVGTQGP  QGPGVLLPLP**K**GACTGWMAGI  RDGRDGTPGE**K**GEKGDPGLIG  RDGTPGEKGE**K**GDPGLIGPKG  EKGDPGLIGP**K**GDIGETGVPG  PRGFPGIQGR**K**GEPGEGAYVY  EDNMEDPPLP**K**GACAGWMAGI  RDGRDGTPGE**K**GEKGDPGLVG  RDGTPGEKGE**K**GDPGLVGPKG  EKGDPGLVGP**K**GDTGETGITG  PRGFPGTPGR**K**GEPGESAYVY  GPGKDGIRGE**K**GEPGADGRVE  ETGPQGQKGD**K**GEVGPTGPEG  RDGRDGTPGE**K**GEKGDAGLLG  RDGTPGEKGE**K**GDAGLLGPKG  EKGDAGLLGP**K**GETGDVGMTG  PRGFPGTPGR**K**GEPGEAAYVY  EKLRRSRSHS**K**NPKRSRSREH  RRSRSHSKNP**K**RSRSREHRRH |

(2) List of the 980 peptide samples in the negative subset $\mathbb{S}^{-}$(K)

| Sample # | Protein ID | Site | Sequences |
| --- | --- | --- | --- |
| 1  2  3  4  5  6  7  8  9  10  11  12  13  14  15  16  17  18  19  20  21  22  23  24  25  26  27  28  29  30  31  32  33  34  35  36  37  38  39  40  41  42  43  44  45  46  47  48  49  50  51  52  53  54  55  56  57  58  59  60  61  62  63  64  65  66  67  68  69  70  71  72  73  74  75  76  77  78  79  80  81  82  83  84  85  86  87  88  89  90  91  92  93  94  95  96  97  98  99  100  101  102  103  104  105  106  107  108  109  110  111  112  113  114  115  116  117  118  119  120  121  122  123  124  125  126  127  128  129  130  131  132  133  134  135  136  137  138  139  140  141  142  143  144  145  146  147  148  149  150  151  152  153  154  155  156  157  158  159  160  161  162  163  164  165  166  167  168  169  170  171  172  173  174  175  176  177  178  179  180  181  182  183  184  185  186  187  188  189  190  191  192  193  194  195  196  197  198  199  200  201  202  203  204  205  206  207  208  209  210  211  212  213  214  215  216  217  218  219  220  221  222  223  224  225  226  227  228  229  230  231  232  233  234  235  236  237  238  239  240  241  242  243  244  245  246  247  248  249  250  251  252  253  254  255  256  257  258  259  260  261  262  263  264  265  266  267  268  269  270  271  272  273  274  275  276  277  278  279  280  281  282  283  284  285  286  287  288  289  290  291  292  293  294  295  296  297  298  299  300  301  302  303  304  305  306  307  308  309  310  311  312  313  314  315  316  317  318  319  320  321  322  323  324  325  326  327  328  329  330  331  332  333  334  335  336  337  338  339  340  341  342  343  344  345  346  347  348  349  350  351  352  353  354  355  356  357  358  359  360  361  362  363  364  365  366  367  368  369  370  371  372  373  374  375  376  377  378  379  380  381  382  383  384  385  386  387  388  389  390  391  392  393  394  395  396  397  398  399  400  401  402  403  404  405  406  407  408  409  410  411  412  413  414  415  416  417  418  419  420  421  422  423  424  425  426  427  428  429  430  431  432  433  434  435  436  437  438  439  440  441  442  443  444  445  446  447  448  449  450  451  452  453  454  455  456  457  458  459  460  461  462  463  464  465  466  467  468  469  470  471  472  473  474  475  476  477  478  479  480  481  482  483  484  485  486  487  488  489  490  491  492  493  494  495  496  497  498  499  500  501  502  503  504  505  506  507  508  509  510  511  512  513  514  515  516  517  518  519  520  521  522  523  524  525  526  527  528  529  530  531  532  533  534  535  536  537  538  539  540  541  542  543  544  545  546  547  548  549  550  551  552  553  554  555  556  557  558  559  560  561  562  563  564  565  566  567  568  569  570  571  572  573  574  575  576  577  578  579  580  581  582  583  584  585  586  587  588  589  590  591  592  593  594  595  596  597  598  599  600  601  602  603  604  605  606  607  608  609  610  611  612  613  614  615  616  617  618  619  620  621  622  623  624  625  626  627  628  629  630  631  632  633  634  635  636  637  638  639  640  641  642  643  644  645  646  647  648  649  650  651  652  653  654  655  656  657  658  659  660  661  662  663  664  665  666  667  668  669  670  671  672  673  674  675  676  677  678  679  680  681  682  683  684  685  686  687  688  689  690  691  692  693  694  695  696  697  698  699  700  701  702  703  704  705  706  707  708  709  710  711  712  713  714  715  716  717  718  719  720  721  722  723  724  725  726  727  728  729  730  731  732  733  734  735  736  737  738  739  740  741  742  743  744  745  746  747  748  749  750  751  752  753  754  755  756  757  758  759  760  761  762  763  764  765  766  767  768  769  770  771  772  773  774  775  776  777  778  779  780  781  782  783  784  785  786  787  788  789  790  791  792  793  794  795  796  797  798  799  800  801  802  803  804  805  806  807  808  809  810  811  812  813  814  815  816  817  818  819  820  821  822  823  824  825  826  827  828  829  830  831  832  833  834  835  836  837  838  839  840  841  842  843  844  845  846  847  848  849  850  851  852  853  854  855  856  857  858  859  860  861  862  863  864  865  866  867  868  869  870  871  872  873  874  875  876  877  878  879  880  881  882  883  884  885  886  887  888  889  890  891  892  893  894  895  896  897  898  899  900  901  902  903  904  905  906  907  908  909  910  911  912  913  914  915  916  917  918  919  920  921  922  923  924  925  926  927  928  929  930  931  932  933  934  935  936  937  938  939  940  941  942  943  944  945  946  947  948  949  950  951  952  953  954  955  956  957  958  959  960  961  962  963  964  965  966  967  968  969  970  971  972  973  974  975  976  977  978  979  980 | O18495  O18495  O18495  P01170  P01170  P01170  P02452  P02452  P02452  P02452  P02452  P02452  P02452  P02452  P02452  P02452  P02452  P02452  P02452  P02452  P02452  P02452  P02452  P02452  P02452  P02452  P02452  P02452  P02452  P02452  P02452  P02452  P02452  P02452  P02452  P02452  P02452  P02452  P02452  P02452  P02452  P02452  P02452  P02452  P02452  P02452  P02452  P02452  P02452  P02452  P02452  P02452  P02452  P02452  P02452  P02452  P02452  P02452  P02452  P02452  P02452  P02453  P02453  P02453  P02453  P02453  P02453  P02453  P02453  P02453  P02453  P02453  P02453  P02453  P02453  P02453  P02453  P02453  P02453  P02453  P02453  P02454  P02454  P02454  P02454  P02454  P02454  P02454  P02454  P02454  P02454  P02454  P02454  P02454  P02454  P02454  P02454  P02454  P02454  P02454  P02454  P02454  P02454  P02454  P02454  P02454  P02454  P02454  P02454  P02454  P02454  P02454  P02454  P02454  P02457  P02457  P02457  P02457  P02457  P02457  P02457  P02457  P02457  P02457  P02457  P02457  P02457  P02457  P02457  P02457  P02457  P02457  P02457  P02457  P02457  P02457  P02457  P02457  P02457  P02457  P02457  P02457  P02457  P02457  P02457  P02457  P02457  P02457  P02457  P02457  P02457  P02457  P02457  P02457  P02459  P02459  P02459  P02459  P02459  P02459  P02459  P02459  P02459  P02459  P02459  P02459  P02459  P02459  P02459  P02459  P02459  P02459  P02459  P02459  P02459  P02459  P02459  P02459  P02459  P02459  P02459  P02459  P02459  P02459  P02459  P02459  P02459  P02459  P02459  P02459  P02459  P02459  P02459  P02459  P02459  P02459  P02459  P02459  P02459  P02459  P02459  P02459  P02459  P02459  P02459  P02459  P02459  P02459  P02461  P02461  P02461  P02461  P02461  P02461  P02461  P02461  P02461  P02461  P02461  P02461  P02461  P02461  P02461  P02461  P02461  P02461  P02461  P02461  P02461  P02461  P02461  P02461  P02461  P02461  P02461  P02461  P02461  P02461  P02461  P02461  P02461  P02461  P02461  P02461  P02461  P02461  P02461  P02461  P02461  P02461  P02461  P02461  P02461  P02461  P02461  P02461  P02461  P02461  P02461  P02461  P02461  P02461  P02461  P02461  P02745  P02745  P02745  P02745  P02745  P02745  P02745  P02745  P02747  P02747  P02747  P02747  P02747  P02747  P02747  P02747  P02747  P02747  P02747  P04142  P04142  P04142  P04142  P04142  P04142  P04142  P04258  P04258  P04258  P04258  P04258  P04258  P04258  P04258  P04258  P04258  P04258  P04258  P04258  P04258  P04258  P04258  P04258  P04258  P04258  P04258  P04258  P04258  P04258  P04258  P04258  P04258  P04258  P04258  P04258  P12108  P12108  P12108  P12108  P12108  P12108  P12108  P12108  P12108  P12108  P12108  P12108  P12108  P12108  P12108  P12108  P12108  P12108  P12108  P12108  P12108  P12108  P12108  P12108  P12108  P12108  P12108  P12108  P12108  P12108  P12108  P12108  P12108  P12108  P12111  P12111  P12111  P12111  P12111  P12111  P12111  P12111  P12111  P12111  P12111  P12111  P12111  P12111  P12111  P12111  P12111  P12111  P12111  P12111  P12111  P12111  P12111  P12111  P12111  P12111  P12111  P12111  P12111  P12111  P12111  P12111  P12111  P12111  P12111  P12111  P12111  P12111  P12111  P12111  P12111  P12111  P12111  P12111  P12111  P12111  P12111  P12111  P12111  P12111  P12111  P12111  P12111  P12111  P12111  P12111  P12111  P12111  P12111  P12111  P12111  P12111  P12111  P12111  P12111  P12111  P12111  P12111  P12111  P12111  P12111  P12111  P12111  P12111  P12111  P12111  P12111  P12111  P12111  P12111  P12111  P12111  P12111  P12111  P12111  P12111  P12111  P12111  P12111  P12111  P12111  P12111  P12111  P12111  P12111  P12111  P12111  P12111  P12111  P12111  P12111  P12111  P12111  P12111  P12111  P12111  P12111  P12111  P12111  P12111  P12111  P12111  P12111  P12111  P12111  P12111  P12111  P12111  P12111  P12111  P12111  P12111  P12111  P12111  P12111  P12111  P12111  P12111  P12111  P12111  P12111  P12111  P12111  P12111  P12111  P12111  P12111  P12111  P12111  P12111  P12111  P12111  P12111  P12111  P12111  P12111  P12111  P12111  P12111  P12111  P12111  P12111  P12111  P19999  P19999  P19999  P19999  P19999  P19999  P19999  P19999  P19999  P19999  P19999  P19999  P19999  P19999  P19999  P19999  P20908  P20908  P20908  P20908  P20908  P20908  P20908  P20908  P20908  P20908  P20908  P20908  P20908  P20908  P20908  P20908  P20908  P20908  P20908  P20908  P20908  P20908  P20908  P20908  P20908  P20908  P20908  P20908  P20908  P20908  P20908  P20908  P20908  P20908  P20908  P20908  P20908  P20908  P20908  P20908  P20908  P20908  P20908  P20908  P20908  P20908  P20908  P20908  P20908  P20908  P20908  P20908  P20908  P20908  P20908  P20908  P20908  P20908  P20908  P20908  P20908  P20908  P20908  P20908  P20908  P20908  P20908  P20908  P20908  P20908  P20908  P20908  P20908  P20908  P20908  P20908  P20908  P20908  P20908  P20908  P20908  P20908  P20908  P20908  P23805  P23805  P23805  P23805  P23805  P23805  P23805  P23805  P23805  P23805  P23805  P23805  P26368  P26368  P26368  P26368  P26368  P26368  P26368  P26368  P26368  P26368  P26368  P26368  P26368  P26368  P26368  P26368  P26368  P26368  P26368  P26368  P26368  P26368  P26368  P26368  P26368  P26368  P30754  P30754  P30754  P30754  P30754  P30754  P30754  P30754  P30754  P30754  P30754  P30754  P30754  P30754  P30754  P30754  P30754  P30754  P30754  P30943  P30943  P30943  P30943  P30943  P30943  P30943  P30943  P30943  P35248  P35248  P35248  P35248  P35248  P35248  P35248  P35248  P35248  P35248  P35248  P35248  P35248  P35248  P35248  P35248  P35248  P35248  P35248  P83414  P83414  P83414  P83414  P83414  P83415  P83415  P83415  P83415  P83415  P84698  Q00433  Q00433  Q00433  Q00433  Q00433  Q00433  Q00433  Q00433  Q00433  Q00433  Q02388  Q02388  Q02388  Q02388  Q02388  Q02388  Q02388  Q02388  Q02388  Q02388  Q02388  Q02388  Q02388  Q02388  Q02388  Q02388  Q02388  Q02388  Q02388  Q02388  Q02388  Q02388  Q02388  Q02388  Q02388  Q02388  Q02388  Q02388  Q02388  Q02388  Q02388  Q02388  Q02388  Q02388  Q02388  Q02388  Q02388  Q02388  Q02388  Q02388  Q02388  Q02388  Q02388  Q02388  Q02388  Q02388  Q02388  Q02388  Q02388  Q02388  Q02388  Q02388  Q02388  Q02388  Q02388  Q02388  Q02388  Q02388  Q02388  Q02388  Q02388  Q02388  Q02388  Q02388  Q02388  Q02388  Q02388  Q02388  Q02388  Q02388  Q02388  Q02388  Q02388  Q02388  Q02388  Q02388  Q02388  Q02388  Q02388  Q02388  Q02388  Q02388  Q02388  Q02388  Q02388  Q02388  Q02388  Q02388  Q02388  Q02388  Q02388  Q05707  Q05707  Q05707  Q05707  Q05707  Q05707  Q05707  Q05707  Q05707  Q05707  Q05707  Q05707  Q05707  Q05707  Q05707  Q05707  Q05707  Q05707  Q05707  Q05707  Q05707  Q05707  Q05707  Q05707  Q05707  Q05707  Q05707  Q05707  Q05707  Q05707  Q05707  Q05707  Q05707  Q05707  Q05707  Q05707  Q05707  Q05707  Q05707  Q05707  Q05707  Q05707  Q05707  Q05707  Q05707  Q05707  Q05707  Q05707  Q05707  Q05707  Q05707  Q05707  Q05707  Q05707  Q05707  Q05707  Q05707  Q05707  Q05707  Q05707  Q05707  Q05707  Q05707  Q05707  Q05707  Q05707  Q05707  Q05707  Q05707  Q05707  Q05707  Q05707  Q05707  Q05707  Q05707  Q05707  Q05707  Q05707  Q05707  Q15848  Q15848  Q15848  Q15848  Q15848  Q15848  Q15848  Q3Y5Z3  Q3Y5Z3  Q3Y5Z3  Q3Y5Z3  Q3Y5Z3  Q3Y5Z3  Q3Y5Z3  Q3Y5Z3  Q4ZJN1  Q4ZJN1  Q4ZJN1  Q4ZJN1  Q4ZJN1  Q4ZJN1  Q4ZJN1  Q4ZJN1  Q4ZJN1  Q4ZJN1  Q4ZJN1  Q4ZJN1  Q4ZJN1  Q4ZJN1  Q4ZJN1  Q4ZJN1  Q4ZJN1  Q4ZJN1  Q4ZJN1  Q4ZJN1  Q4ZJN1  Q4ZJN1  Q60994  Q60994  Q60994  Q60994  Q60994  Q60994  Q60994  Q60994  Q9SE35  Q9SE35  Q9SE35  Q9SE35  Q9SE35  Q9SE35  Q9SE35  Q9SE35  Q9SE35  Q9SE35  Q9SE35  Q9SE35  Q9SE35  Q9SE35  Q9SE35  Q9SE35  Q9SE35  Q9SE35  Q9SE35  Q9SE35  Q9SE35  Q9SE35  Q9SE35  Q9SE35  Q9Y383  Q9Y383  Q9Y383  Q9Y383  Q9Y383  Q9Y383  Q9Y383  Q9Y383  Q9Y383  Q9Y383  Q9Y383  Q9Y383  Q9Y383  Q9Y383  Q9Y383  Q9Y383  Q9Y383  Q9Y383  Q9Y383  Q9Y383  Q9Y383  Q9Y383  Q9Y383  Q9Y383  Q9Y383  Q9Y383  Q9Y383  Q9Y383  Q9Y383 | 4  51  67  61  111  115  54  67  79  114  170  228  237  277  286  352  397  430  442  448  468  505  520  538  552  586  594  612  657  709  742  751  759  781  826  835  862  903  907  934  984  1033  1062  1096  1152  1208  1236  1253  1263  1270  1288  1310  1316  1319  1321  1371  1385  1386  1424  1430  1433  54  79  85  170  351  429  447  467  504  519  750  758  902  906  1151  1207  1309  1315  1318  1320  45  67  77  104  160  266  275  341  419  431  437  457  583  601  770  815  824  851  892  896  1022  1051  1097  1197  1277  1299  1308  1310  1311  1374  1375  1413  1419  43  47  107  160  217  226  266  275  341  386  509  575  583  601  646  731  748  770  815  824  850  891  894  921  1020  1095  1139  1240  1250  1257  1275  1297  1303  1306  1308  1309  1358  1372  1373  1420  30  44  48  73  103  106  113  119  140  143  250  259  262  490  574  616  634  679  731  764  773  781  803  848  857  884  925  929  956  1006  1055  1084  1118  1174  1231  1260  1277  1287  1294  1312  1333  1334  1340  1342  1344  1345  1394  1408  1409  1435  1440  1444  1447  1456  7  46  108  161  229  251  275  350  395  436  440  461  466  503  550  584  592  610  629  655  662  674  707  740  743  757  779  824  833  901  923  932  982  1031  1060  1150  1212  1228  1239  1256  1266  1273  1286  1291  1313  1323  1324  1373  1387  1388  1391  1401  1407  1419  1426  1435  32  81  103  110  195  219  222  223  14  47  57  72  86  115  117  157  161  188  198  5  29  32  33  36  59  63  8  73  128  239  280  284  305  310  347  394  454  473  499  506  518  584  587  601  623  668  677  704  745  767  821  826  875  904  994  51  63  77  87  99  137  154  190  207  211  241  250  268  277  295  306  316  343  349  385  406  424  427  432  436  451  454  463  529  543  595  597  601  634  3  33  67  99  120  132  155  166  186  233  271  302  310  370  414  430  443  470  505  514  516  551  560  573  585  637  652  699  783  835  861  869  885  896  904  907  909  912  930  964  976  1001  1014  1028  1048  1088  1156  1231  1281  1292  1305  1325  1347  1360  1380  1403  1418  1472  1491  1518  1530  1551  1635  1636  1637  1694  1699  1708  1712  1720  1754  1772  1788  1833  1856  1861  1913  1931  1936  1946  2027  2035  2052  2094  2121  2130  2139  2145  2170  2196  2272  2281  2309  2310  2355  2367  2384  2386  2464  2466  2472  2474  2483  2500  2510  2532  2639  2640  2656  2684  2695  2697  2737  2763  2765  2777  2781  2796  2800  2834  2846  2850  2854  2875  2881  2888  2895  2906  2911  2916  2921  2926  2937  2943  2948  2961  2966  2976  2981  2986  2990  3006  3035  3077  3078  3113  3116  3127  3135  3151  3156  3160  3168  28  63  94  106  108  114  122  123  126  127  140  142  162  169  199  200  8  44  57  69  79  88  107  109  110  146  164  174  175  184  185  186  189  288  294  295  301  329  452  455  535  582  605  687  749  767  825  866  905  929  933  963  971  989  1034  1038  1049  1074  1119  1125  1158  1167  1170  1173  1212  1239  1284  1305  1311  1326  1335  1365  1391  1407  1410  1421  1433  1473  1479  1482  1500  1515  1542  1545  1554  1619  1626  1640  1668  1685  1686  1697  1708  1711  1762  1792  1793  1797  1806  1826  29  122  222  243  244  257  260  266  279  303  355  363  20  26  38  63  70  84  85  86  87  90  108  195  225  260  286  292  300  328  329  340  408  413  431  444  453  462  50  62  113  146  299  383  392  395  425  443  488  584  590  674  686  734  749  851  986  4  44  61  67  71  78  83  101  104  23  62  118  124  133  137  143  158  164  203  215  236  248  249  262  277  308  322  362  1  3  6  7  10  3  6  7  10  33  34  4  59  76  82  93  98  108  109  118  127  113  142  151  164  169  176  184  263  459  727  1253  1256  1295  1325  1346  1367  1399  1402  1443  1444  1447  1479  1494  1518  1533  1553  1556  1588  1615  1639  1653  1672  1693  1718  1733  1769  1786  1801  1831  1837  1843  1856  1859  1874  1912  1921  1952  1981  1992  2005  2038  2066  2093  2113  2116  2128  2137  2166  2206  2255  2267  2289  2309  2312  2329  2341  2359  2365  2368  2388  2457  2460  2507  2516  2519  2535  2550  2562  2565  2571  2603  2613  2640  2643  2658  2675  2682  2694  2700  2742  2780  2  7  51  56  61  71  83  104  106  109  118  122  124  127  131  148  194  218  231  250  261  270  295  346  382  394  410  462  488  491  591  655  665  672  754  796  805  825  857  864  898  916  928  960  984  990  1004  1023  1027  1030  1050  1064  1086  1091  1093  1101  1106  1111  1114  1117  1136  1152  1183  1199  1200  1222  1231  1241  1266  1311  1326  1351  1359  1370  1376  1381  1399  1431  1558  134  149  169  172  177  178  180  129  144  164  167  172  175  187  202  49  52  66  85  88  99  103  124  148  159  184  190  192  201  213  222  225  240  268  272  277  296  36  152  172  175  180  181  183  195  2  36  110  111  121  122  132  133  143  144  154  155  165  166  174  177  184  185  193  196  203  204  250  253  29  37  61  76  105  106  122  135  139  154  161  165  166  186  213  224  229  235  238  245  257  289  295  299  332  337  365  367  368 | ITAKMQMMQM**K**ATILIVLVAL  YYIKAAWQIG**K**HALGDMTDEE  MTDEEFQDFM**K**EVEQAREEEL  SAGLLSQEWS**K**RAVEELLAQM  STNNLPPRER**K**AGCKNFYWKG  LPPRERKAGC**K**NFYWKGFTSC  GLRYHDRDVW**K**PEPCRICVCD  PCRICVCDNG**K**VLCDDVICDE  LCDDVICDET**K**NCPGAEVPEG  DQETTGVEGP**K**GDTGPRGPRG  APQLSYGYDE**K**STGGISVPGP  GPRGPPGPPG**K**NGDDGEAGKP  GKNGDDGEAG**K**PGRPGERGPP  HRGFSGLDGA**K**GDAGPAGPKG  AKGDAGPAGP**K**GEPGSPGENG  PPGFPGAVGA**K**GEAGPQGPRG  NPGADGQPGA**K**GANGAPGIAG  PQGPGGPPGP**K**GNSGEPGAPG  NSGEPGAPGS**K**GDTGAKGEPG  APGSKGDTGA**K**GEPGPVGVQG  GPPGPAGEEG**K**RGARGEPGPT  FPGADGVAGP**K**GPAGERGSPG  ERGSPGPAGP**K**GSPGEAGRPG  RPGEAGLPGA**K**GLTGSPGSPG  GSPGSPGPDG**K**TGPPGPAGQD  QAGVMGFPGP**K**GAAGEPGKAG  GPKGAAGEPG**K**AGERGVPGPP  GPPGAVGPAG**K**DGEAGAQGPP  GPAGPPGEAG**K**PGEQGVPGDL  ANGAPGNDGA**K**GDAGAPGAPG  ERGAAGLPGP**K**GDRGDAGPKG  PKGDRGDAGP**K**GADGSPGKDG  GPKGADGSPG**K**DGVRGLTGPI  PPGPAGAPGD**K**GESGPSGPAG  PPGADGQPGA**K**GEPGDAGAKG  AKGEPGDAGA**K**GDAGPPGPAG  PIGNVGAPGA**K**GARGSAGPPG  GPPGPPGPAG**K**EGGKGPRGET  PPGPAGKEGG**K**GPRGETGPAG  PPGPPGPAGE**K**GSPGADGPAG  GLPGPSGEPG**K**QGPSGASGER  SPGRDGSPGA**K**GDRGETGPAG  GAPGPVGPAG**K**SGDRGETGPA  PAGPQGPRGD**K**GETGEQGDRG  GPPGSAGAPG**K**DGLNGLPGPI  FSFLPQPPQE**K**AHDGGRYYRA  DRDLEVDTTL**K**SLSQQIENIR  ENIRSPEGSR**K**NPARTCRDLK  KNPARTCRDL**K**MCHSDWKSGE  RDLKMCHSDW**K**SGEYWIDPNQ  PNQGCNLDAI**K**VFCNMETGET  VYPTQPSVAQ**K**NWYISKNPKD  SVAQKNWYIS**K**NPKDKRHVWF  QKNWYISKNP**K**DKRHVWFGES  NWYISKNPKD**K**RHVWFGESMT  EASQNITYHC**K**NSVAYMDQQT  AYMDQQTGNL**K**KALLLQGSNE  YMDQQTGNLK**K**ALLLQGSNEI  GCTSHTGAWG**K**TVIEYKTTKT  GAWGKTVIEY**K**TTKTSRLPII  GKTVIEYKTT**K**TSRLPIIDVA  GLRYHDRDVW**K**PVPCQICVCD  LCDDVICDEL**K**DCPNAKVPTD  CDELKDCPNA**K**VPTDECCPVC  APQLSYGYDE**K**STGISVPGPM  PPGFPGAVGA**K**GEGGPQGPRG  PQGPSGPPGP**K**GNSGEPGAPG  APGSKGDTGA**K**GEPGPTGIQG  GPPGPAGEEG**K**RGARGEPGPA  FPGADGVAGP**K**GPAGERGAPG  ERGAPGPAGP**K**GSPGEAGRPG  PKGDRGDAGP**K**GADGAPGKDG  GPKGADGAPG**K**DGVRGLTGPI  GPPGPPGPAG**K**EGSKGPRGET  PPGPAGKEGS**K**GPRGETGPAG  GPPGSAGSPG**K**DGLNGLPGPI  LSFLPQPPQE**K**AHDGGRYYRA  VYPTQPSVAQ**K**NWYISKNPKE  SVAQKNWYIS**K**NPKEKRHVWY  QKNWYISKNP**K**EKRHVWYGES  NWYISKNPKE**K**RHVWYGESMT  GLRVPNGETW**K**PDVCLICICH  GTAVCDGVLC**K**EDLDCPNPQK  KEDLDCPNPQ**K**REGECCPFCP  DAEVIGVEGP**K**GDPGPQGPRG  ASQMSYGYDE**K**SAGVSVPGPM  HRGFSGLDGA**K**GDTGPAGPKG  AKGDTGPAGP**K**GEPGSPGENG  PPGFPGAAGA**K**GEAGPQGARG  PQGPSGAPGP**K**GNSGEPGAPG  NSGEPGAPGN**K**GDTGAKGEPG  APGNKGDTGA**K**GEPGPAGVQG  GPPGPAGEEG**K**RGARGEPGPS  GPKGTAGEPG**K**AGERGVPGPP  GPPGAVGPAG**K**DGEAGAQGAP  PPGPAGAPGD**K**GETGPSGPAG  PPGADGQPGA**K**GEPGDTGVKG  AKGEPGDTGV**K**GDAGPPGPAG  PIGNVGAPGP**K**GSRGAAGPPG  GPPGPPGPVG**K**EGGKGPRGET  PPGPVGKEGG**K**GPRGETGPAG  SPGRDGAPGA**K**GDRGETGPAG  GAPGPVGPAG**K**NGDRGETGPA  ETGEQGDRGI**K**GHRGFSGLQG  FSFLPQPPQE**K**SQDGGRYYRA  PNQGCNLDAI**K**VYCNMETGQT  VFPTQPSVPQ**K**NWYISPNPKE  QKNWYISPNP**K**EKKHVWFGES  NWYISPNPKE**K**KHVWFGESMT  WYISPNPKEK**K**HVWFGESMTD  AYMDQQTGNL**K**KSLLLQGSNE  YMDQQTGNLK**K**SLLLQGSNEI  GCTSHTGTWG**K**TVIEYKTTKT  GTWGKTVIEY**K**TTKTSRLPII  CVQDGLTYND**K**DVWKPEPCQI  GLTYNDKDVW**K**PEPCQICVCD  YPESAGVEGP**K**GDTGPRGDRG  APQMSYGYDE**K**SAGVAVPGPM  GPRGPAGPPG**K**NGDDGEAGKP  GKNGDDGEAG**K**PGRPGQRGPP  HRGFSGLDGA**K**GQPGPAGPKG  AKGQPGPAGP**K**GEPGSPGENG  PPGFPGAAGA**K**GETGPQGARG  NPGADGQPGA**K**GATGAPGIAG  ERGSPGAVGP**K**GSPGEAGRPG  QAGVMGFPGP**K**GAAGEPGKPG  GPKGAAGEPG**K**PGERGAPGPP  GPPGAVGAAG**K**DGEAGAQGPP  GPAGPPGEAG**K**PGEQGVPGNA  ERGAAGLPGA**K**GDRGDPGPKG  GPKGADGAPG**K**DGLRGLTGPI  PPGPAGAPGD**K**GEAGPPGPAG  PPGADGQPGA**K**GETGDAGAKG  AKGETGDAGA**K**GDAGPPGPAG  GPAGVGAPGP**K**GARGSAGPPG  GLPGPPGPAG**K**GSKGPRGETG  GPPGPAGKGS**K**GPRGETGPAG  PAGPPGPPGE**K**GSPGADGPIG  APGRDGAAGP**K**GDRGETGPAG  ETGEQGDRGM**K**GHRGFSGLQG  GPPGSAGAAG**K**DGLNGLPGPI  ENIRSPEGTR**K**NPARTCRDLK  KNPARTCRDL**K**MCHGDWKSGE  RDLKMCHGDW**K**SGEYWIDPNQ  PNQGCNLDAI**K**VYCNMETGET  VYPTQATIAQ**K**NWYLSKNPKE  TIAQKNWYLS**K**NPKEKKHVWF  QKNWYLSKNP**K**EKKHVWFGET  NWYLSKNPKE**K**KHVWFGETMS  WYLSKNPKEK**K**HVWFGETMSD  EATQNVTYHC**K**NSVAYMDHDT  AYMDHDTGNL**K**KALLLQGANE  YMDHDTGNLK**K**ALLLQGANEI  GKTVIEYKTT**K**TSRLPIIDLA  VLRCHGQDVQ**K**AGSCVQDGQR  CVQDGQRYND**K**DVWKPEPCRI  GQRYNDKDVW**K**PEPCRICVCD  LCDDIICEDM**K**DCLSPETPFG  LPTASGQPGP**K**GQKGEPGDIK  ASGQPGPKGQ**K**GEPGDIKDIV  KGQKGEPGDI**K**DIVGPKGPPG  PGDIKDIVGP**K**GPPGPQGPAG  EQGPRGDRGD**K**GEKGAPGPRG  PRGDRGDKGE**K**GAPGPRGRDG  GPRGPPGPPG**K**PGDDGEAGKP  GKPGDDGEAG**K**PGKSGERGPP  GDDGEAGKPG**K**SGERGPPGPQ  GAPGPAGEEG**K**RGARGEPGGA  GRPGDAGPQG**K**VGPSGAPGED  GPKGANGEPG**K**AGEKGLPGAP  GAPGLRGLPG**K**DGETGAAGPP  GPPGPPGEGG**K**PGDQGVPGEA  LPGTPGTDGP**K**GAAGPAGPPG  ERGAAGIAGP**K**GDRGDVGEKG  PKGDRGDVGE**K**GPEGAPGKDG  GEKGPEGAPG**K**DGGRGLTGPI  PPGPAGANGE**K**GEVGPPGPAG  PPGADGQPGA**K**GEQGEAGQKG  AKGEQGEAGQ**K**GDAGAPGPQG  PQGPTGVTGP**K**GARGAQGPPG  GPPGPPGPSG**K**DGPKGARGDS  PPGPSGKDGP**K**GARGDSGPPG  LQGPAGPPGE**K**GEPGDDGPSG  GLPGPSGEPG**K**QGAPGASGDR  PPGRDGAAGV**K**GDRGETGAVG  GSPGPAGPIG**K**QGDRGEAGAQ  MPGPQGPRGD**K**GETGEAGERG  GPPGPVGPSG**K**DGANGIPGPI  SAFAGLGQRE**K**GPDPLQYMRA  QHDAEVDATL**K**SLNNQIESLR  ESLRSPEGSR**K**NPARTCRDLK  KNPARTCRDL**K**LCHPEWKSGD  RDLKLCHPEW**K**SGDYWIDPNQ  PNQGCTLDAM**K**VFCNMETGET  CVYPNPASVP**K**KNWWSSKSKD  VYPNPASVPK**K**NWWSSKSKDK  SVPKKNWWSS**K**SKDKKHIWFG  PKKNWWSSKS**K**DKKHIWFGET  KNWWSSKSKD**K**KHIWFGETIN  NWWSSKSKDK**K**HIWFGETING  EGSQNITYHC**K**NSIAYLDEAA  AYLDEAAGNL**K**KALLIQGSND  YLDEAAGNLK**K**ALLIQGSNDV  GNSRFTYTVL**K**DGCTKHTGKW  TYTVLKDGCT**K**HTGKWGKTMI  LKDGCTKHTG**K**WGKTMIEYRS  GCTKHTGKWG**K**TMIEYRSQKT  GKTMIEYRSQ**K**TSRLPIIDIA  FSMMMMSFVQ**K**GSWLLLALLH  GQSYADRDVW**K**PEPCQICVCD  PPNGQGPQGP**K**GDPGPPGIPG  YSPQYDSYDV**K**SGVAVGGLAG  GAIGPSGPAG**K**DGESGRPGRP  ERGLPGPPGI**K**GPAGIPGFPG  HRGFDGRNGE**K**GETGAPGLKG  TAGFPGSPGA**K**GEVGPAGSPG  PPGINGSPGG**K**GEMGPAGIPG  GLRGGAGEPG**K**NGAKGEPGPR  GAGEPGKNGA**K**GEPGPRGERG  EAGIPGVPGA**K**GEDGKDGSPG  GVPGAKGEDG**K**DGSPGEPGAN  PAGPNGIPGE**K**GPAGERGAPG  GSPGGPGSDG**K**PGPPGSQGES  QPGVMGFPGP**K**GNDGAPGKNG  GPKGNDGAPG**K**NGERGGPGGP  GGPGPQGPPG**K**NGETGPQGPP  PPGPTGPGGD**K**GDTGPPGPQG  GTGGPPGENG**K**PGEPGPKGDA  ENGKPGEPGP**K**GDAGAPGAPG  DAGAPGAPGG**K**GDAGAPGERG  GAGPPGPEGG**K**GAAGPPGPPG  ERGGLGSPGP**K**GDKGEPGGPG  GLGSPGPKGD**K**GEPGGPGADG  GGPGADGVPG**K**DGPRGPTGPI  PPGPAGQPGD**K**GEGGAPGLPG  APGQNGEPGG**K**GERGAPGEKG  GKGERGAPGE**K**GEGGPPGVAG  GPPGPSGSPG**K**DGPPGPAGNT  APGSPGVSGP**K**GDAGQPGEKG  PKGDAGQPGE**K**GSPGAQGPPG  GPQGVKGESG**K**PGANGLSGER  LPGRDGSPGG**K**GDRGENGSPG  GPPGPVGPAG**K**SGDRGESGPA  GPVGPSGPPG**K**DGTSGHPGPI  AAAIAGIGGE**K**AGGFAPYYGD  PYYGDEPMDF**K**INTDEIMTSL  INTDEIMTSL**K**SVNGQIESLI  ESLISPDGSR**K**NPARNCRDLK  KNPARNCRDL**K**FCHPELKSGE  RDLKFCHPEL**K**SGEYWVDPNQ  EYWVDPNQGC**K**LDAIKVFCNM  PNQGCKLDAI**K**VFCNMETGET  ISANPLNVPR**K**HWWTDSSAEK  KHWWTDSSAE**K**KHVWFGESMD  HWWTDSSAEK**K**HVWFGESMDG  RASQNITYHC**K**NSIAYMDQAS  AYMDQASGNV**K**KALKLMGSNE  YMDQASGNVK**K**ALKLMGSNEG  QASGNVKKAL**K**LMGSNEGEFK  KLMGSNEGEF**K**AEGNSKFTYT  EGEFKAEGNS**K**FTYTVLEDGC  TYTVLEDGCT**K**HTGEWSKTVF  GCTKHTGEWS**K**TVFEYRTRKA  SKTVFEYRTR**K**AVRLPIVDIA  TEDLCRAPDG**K**KGEAGRPGRR  GEPGPSGNPG**K**VGYPGPSGPL  ARGIPGIKGT**K**GSPGNIKDQP  KGTKGSPGNI**K**DQPRPAFSAI  RSLGFCDTTN**K**GLFQVVSGGM  LQQGDQVWVE**K**DPKKGHIYQG  GDQVWVEKDP**K**KGHIYQGSEA  DQVWVEKDPK**K**GHIYQGSEAD  GPSSLPHLGL**K**LLLLLLLLPL  GMPGLPGAPG**K**DGYDGLPGPK  KDGYDGLPGP**K**GEPGIPAIPG  IPAIPGIRGP**K**GQKGEPGLPG  GEPGLPGHPG**K**NGPMGPPGMP  PGEPGEEGRY**K**QKFQSVFTVT  EPGEEGRYKQ**K**FQSVFTVTRQ  PQGDYDTSTG**K**FTCKVPGLYY  YDTSTGKFTC**K**VPGLYYFVYH  LCVLLYRSGV**K**VVTFCGHTSK  KVVTFCGHTS**K**TNQVNSGGVL  IKAFNMMNFA**K**ILSFVFALVL  MTSAAPEPRW**K**IFKKIEKMGR  AAPEPRWKIF**K**KIEKMGRNIR  APEPRWKIFK**K**IEKMGRNIRD  PRWKIFKKIE**K**MGRNIRDGIV  GPAIEVLGSA**K**AIGKKGIAKA  EVLGSAKAIG**K**KGIAKASGLV  EYEEYEAYDV**K**SGVAGGGIAG  GPPGAIGPSG**K**DGESGRPGRP  EKGEPGAPGL**K**GENGVPGEDG  PPGSDGSPGG**K**GEMGPAGIPG  GQRGAAGEPG**K**NGAKGDPGPR  AAGEPGKNGA**K**GDPGPRGERG  EAGSPGIAGP**K**GEDGKDGSPG  GIAGPKGEDG**K**DGSPGEPGAN  PAGANGLPGE**K**GPPGDRGGPG  GSPGGPGSNG**K**PGPPGSQGET  GGPGPQGPAG**K**NGETGPQGPP  PPGPTGPSGD**K**GDTGPPGPQG  GTSGPPGENG**K**PGEPGPKGEA  ENGKPGEPGP**K**GEAGAPGIPG  EAGAPGIPGG**K**GDSGAPGERG  ERGGPGGPGP**K**GDKGEPGSSG  GPGGPGPKGD**K**GEPGSSGVDG  GSSGVDGAPG**K**DGPRGPTGPI  PPGPAGQPGD**K**GESGAPGVPG  APGQNGEPGA**K**GERGAPGEKG  AKGERGAPGE**K**GEGGPPGAAG  PAGPPGPQGV**K**GERGSPGGPG  GPPGSSGAPG**K**DGPPGPPGSN  APGSPGISGP**K**GDSGPPGERG  ARGSPGPQGI**K**GENGKPGPSG  GPQGIKGENG**K**PGPSGQNGER  LPGRDGAPGA**K**GDRGENGSPG  GPPGPVGPAG**K**SGDRGETGPA  GPVGPSGPPG**K**DGASGHPGPI  VPGADGIDGD**K**GSPGAPGSPG  SPGAPGSPGA**K**GEPGAPGPDG  GAPGPDGPPG**K**PGLDGLTGAK  KPGLDGLTGA**K**GSRGPWGGQG  SRGPWGGQGL**K**GQPGLPGPPG  LPGEIGVPGP**K**GDPGPDGPRG  GPRGPPGPPG**K**PGPPGHIQGV  PKGPQGLQGL**K**GHRGRPGALG  GALGEPGQQG**K**QGPKGDVGVS  EPGQQGKQGP**K**GDVGVSGEQG  QRGYPGMAGP**K**GETGPAGYKG  PKGETGPAGY**K**GMVGTIGAAG  AAGRPGREGP**K**GPPGDPGEKG  PKGPPGDPGE**K**GELGGRGIRG  IRGPQGDIGP**K**GDMGLPGIDG  GDMGLPGIDG**K**DGTPGIPGVK  KDGTPGIPGV**K**GTAGQPGRPG  QAGLPGQPGS**K**GGPGDKGEVG  QPGSKGGPGD**K**GEVGARGQQG  DAGTAGVPGL**K**GDRGERGPVG  APGEAGQSGP**K**GEQGPPGIPG  IPGPQGLPGV**K**GDKGSPGKTG  PQGLPGVKGD**K**GSPGKTGPKG  GVKGDKGSPG**K**TGPKGSTGDP  DKGSPGKTGP**K**GSTGDPGVHG  DPGVHGLAGV**K**GEKGESGEPG  VHGLAGVKGE**K**GESGEPGPKG  EKGESGEPGP**K**GQQGIQGELG  GDQHIIDVVL**K**MMQEQLAEVA  EQLAEVAVSA**K**RAALGGVGAM  AAGQIGNIGP**K**GKRGEKGERG  GQIGNIGPKG**K**RGEKGERGDT  NIGPKGKRGE**K**GERGDTGRGH  PGIPGHALAG**K**DGERGPPGVP  PLHRHKRMMR**K**HRHLPLVAVF  THAQQQQADV**K**NGAAADIIFL  LVREFLYDVV**K**SLAVGENDFH  TEFLLNTYRT**K**QEVLSHISNM  SYIGGTNQTG**K**GLEYIMQSHL  LEYIMQSHLT**K**AAGSRAGDGV  VIVVLTDGHS**K**DGLALPSAEL  DGLALPSAEL**K**SADVNVFAIG  GVEDADEGAL**K**EIASEPLNMH  PERAGDTETL**K**DITAQDSADI  ILDFLVNLLE**K**LPIGTQQIRV  TMFSLDTYST**K**AQVLGAVKAL  STKAQVLGAV**K**ALGFAGGELA  DEIRYGVVAL**K**QASVFSFGLG  EFRSFGDLQE**K**LLPYIVGVAQ  VGVAQRHIVL**K**PPTIVTQVIE  TIVTQVIEVN**K**RDIVFLVDGS  NFNAIRDFIA**K**VIQRLEIGQD  PEFYFNTHPT**K**REVITAVRKM  TKREVITAVR**K**MKPLDGSALY  REVITAVRKM**K**PLDGSALYTG  AGYRAAEGIP**K**LLVLITGGKS  PKLLVLITGG**K**SLDEISQPAQ  DEISQPAQEL**K**RSSIMAFAIG  SSIMAFAIGN**K**GADQAELEEI  LSGTPEVHSN**K**RDIIFLLDGS  FLLDGSANVG**K**TNFPYVRDFV  TEFSLNTYQT**K**SDILGHLRQL  TFCVGASQAN**K**AELEQIAFNP  EEVPLAQPES**K**RDILFLFDGS  QFPVVRDFLY**K**IIDELNVKPE  LYKIIDELNV**K**PEGTRIAVAQ  IAVAQYSDDV**K**VESRFDEHQS  VESRFDEHQS**K**PEILNLVKRM  QSKPEILNLV**K**RMKIKTGKAL  PEILNLVKRM**K**IKTGKALNLG  ILNLVKRMKI**K**TGKALNLGYA  LVKRMKIKTG**K**ALNLGYALDY  LDYAQRYIFV**K**SAGSRIEDGV  DRVDGPASNL**K**QSGVVPFIFQ  SGVVPFIFQA**K**NADPAELEQI  AFILAAESLP**K**IGDLHPQIVN  DLHPQIVNLL**K**SVHNGAPAPV  NGAPAPVSGE**K**DVVFLLDGSE  EGVRSGFPLL**K**EFVQRVVESL  PEFYLNSYMN**K**QDVVNAVRQL  DDVRNPSVVV**K**RGGAVPIGIG  QPLPSPGVGG**K**RDVVFLIDGS  VAVIQFSDDP**K**VEFLLNAHSS  VEFLLNAHSS**K**DEVQNAVQRL  VQNAVQRLRP**K**GGRQINVGNA  ALEYVSRNIF**K**RPLGSRIEEG  PQFLVLISSG**K**SDDEVDDPAV  DEVDDPAVEL**K**QFGVAPFTIA  ARNADQEELV**K**ISLSPEYVFS  TFRELPSLEQ**K**LLTPITTLTS  ITTLTSEQIQ**K**LLASTRYPPP  IVRRLNIGPS**K**VRVGVVQFSN  SNDVFPEFYL**K**TYRSQAPVLD  LRGGSPLNTG**K**ALEFVARNLF  LEFVARNLFV**K**SAGSRIEDGV  PQHLVLVLGG**K**SQDDVSRFAQ  VDTPPPSRPE**K**KKADIVFLLD  DTPPPSRPEK**K**KADIVFLLDG  TPPPSRPEKK**K**ADIVFLLDGS  NSDPTDEFFL**K**DFSTKRQIID  DEFFLKDFST**K**RQIIDAINKV  TKRQIIDAIN**K**VVYKGGRHAN  IIDAINKVVY**K**GGRHANTKVG  VYKGGRHANT**K**VGLEHLRVNH  PQIAFVITGG**K**SVEDAQDVSL  VSLALTQRGV**K**VFAVGVRNID  VRNIDSEEVG**K**IASNSATAFR  TLCPGVTDAA**K**ACNLDVILGF  SRDQNVFVAQ**K**GFESKVDAIL  VFVAQKGFES**K**VDAILNRISQ  FDEYQPEMLE**K**FRNMRSQHPY  HPYVLTEDTL**K**VYLNKFRQSS  TEDTLKVYLN**K**FRQSSPDSVK  KFRQSSPDSV**K**VVIHFTDGAD  LAEQLDNIAE**K**ACCGVPCKCS  AEKACCGVPC**K**CSGQRGDRGP  DRGPIGSIGP**K**GIPGEDGYRG  FQGCPGQRGV**K**GSRGFPGEKG  LDGLDGEDGD**K**GLPGSSGEKG  DKGLPGSSGE**K**GNPGRRGDKG  EKGNPGRRGD**K**GPRGEKGERG  RRGDKGPRGE**K**GERGDVGIRG  PGQDSQERGP**K**GETGDLGPMG  GVPGGPGETG**K**NGGFGRRGPP  ERGRTGPLGR**K**GEPGEPGPKG  RKGEPGEPGP**K**GGIGNRGPRG  DGVGSEGRRG**K**KGERGFPGYP  GVGSEGRRGK**K**GERGFPGYPG  NSGPPGIVGQ**K**GDPGYPGPAG  DPGYPGPAGP**K**GNRGDSIDQC  IDQCALIQSI**K**DKCPCCYGPL  QCALIQSIKD**K**CPCCYGPLEC  VTTEIRFADS**K**RKSVLLDKIK  TEIRFADSKR**K**SVLLDKIKNL  DSKRKSVLLD**K**IKNLQVALTS  KRKSVLLDKI**K**NLQVALTSKQ  IKNLQVALTS**K**QQSLETAMSF  AMSFVARNTF**K**RVRNGFLMRK  KRVRNGFLMR**K**VAVFFSNTPT  ASPQLREAVL**K**LSDAGITPLF  TTTLFQFNEM**K**KYIAYLVRQL  TTLFQFNEMK**K**YIAYLVRQLD  VRQLDMSPDP**K**ASQHFARVAV  SVDNASMPPV**K**VEFSLTDYGS  VEFSLTDYGS**K**EKLVDFLSRG  FSLTDYGSKE**K**LVDFLSRGMT  FESAPNPRDL**K**IVVLMLTGEV  EEAQRVILQA**K**CKGYFFVVLG  AQRVILQAKC**K**GYFFVVLGIG  YFFVVLGIGR**K**VNIKEVYTFA  VLGIGRKVNI**K**EVYTFASEPN  FASEPNDVFF**K**LVDKSTELNE  PNDVFFKLVD**K**STELNEEPLM  NAFYLSPDIR**K**QCDWFQGDQP  CDWFQGDQPT**K**NLVKFGHKQV  QGDQPTKNLV**K**FGHKQVNVPN  PTKNLVKFGH**K**QVNVPNNVTS  SPTSNPVTTT**K**PVTTTKPVTT  VTTTKPVTTT**K**PVTTTTKPVT  TTTKPVTTTT**K**PVTTTTKPVT  TTTKPVTTTT**K**PVTIINQPSV  PVTIINQPSV**K**PAAAKPAPAK  NQPSVKPAAA**K**PAPAKPVAAK  KPAAAKPAPA**K**PVAAKPVATK  KPAPAKPVAA**K**PVATKMATVR  KPVAAKPVAT**K**MATVRPPVAV  MATVRPPVAV**K**PATAAKPVAA  PVAVKPATAA**K**PVAAKPAAVR  PATAAKPVAA**K**PAAVRPPAAA  AVRPPAAAAA**K**PVATKPEVPR  AAAAAKPVAT**K**PEVPRPQAAK  KPEVPRPQAA**K**PAATKPATTK  RPQAAKPAAT**K**PATTKPMVKM  KPAATKPATT**K**PMVKMSREVQ  TKPATTKPMV**K**MSREVQVFEI  QVFEITENSA**K**LHWERAEPPG  TSAHDQSLVL**K**QNLTVTDRVI  RATYHGSFST**K**KSQPPPPQPA  ATYHGSFSTK**K**SQPPPPQPAR  PLALTETDIC**K**LPKDEGTCRD  LTETDICKLP**K**DEGTCRDFIL  DEGTCRDFIL**K**WYYDPNTKSC  ILKWYYDPNT**K**SCARFWYGGC  WYGGCGGNEN**K**FGSQKECEKV  GGNENKFGSQ**K**ECEKVCAPVL  NKFGSQKECE**K**VCAPVLAKPG  CEKVCAPVLA**K**PGVISVMGTT  SGSQTCEETL**K**TCSVIACGRD  GLRGLQGPPG**K**LGPPGSVGAP  DRGDSRAIEV**K**LANMEAEINT  ANMEAEINTL**K**SKLELTNKLH  MEAEINTLKS**K**LELTNKLHAF  TLKSKLELTN**K**LHAFSMGKKS  TNKLHAFSMG**K**KSGKKFFVTN  NKLHAFSMGK**K**SGKKFFVTNH  HAFSMGKKSG**K**KFFVTNHERM  AFSMGKKSGK**K**FFVTNHERMP  VTNHERMPFS**K**VKALCSELRG  NHERMPFSKV**K**ALCSELRGTV  VAIPRNAEEN**K**AIQEVAKTSA  EENKAIQEVA**K**TSAFLGITDE  TGGRLTYSNW**K**KDEPNDHGSG  GGRLTYSNWK**K**DEPNDHGSGE  VDMMDVHTRW**K**ARSALRPGAP  SRAAQPADLL**K**VLDFHNLPDG  DFHNLPDGIT**K**TTGFCATRRS  TGFCATRRSS**K**GPDVAYRVTK  KGPDVAYRVT**K**DAQLSAPTKQ  TKDAQLSAPT**K**QLYPASAFPE  PEDFSILTTV**K**AKKGSQAFLV  DFSILTTVKA**K**KGSQAFLVSI  FSILTTVKAK**K**GSQAFLVSIY  PVFLYEDHTG**K**PGPEDYPLFR  LFRGINLSDG**K**WHRIALSVHK  KWHRIALSVH**K**KNVTLILDCK  WHRIALSVHK**K**NVTLILDCKK  KKNVTLILDC**K**KKTTKFLDRS  KNVTLILDCK**K**KTTKFLDRSD  NVTLILDCKK**K**TTKFLDRSDH  LILDCKKKTT**K**FLDRSDHPMI  PYYEDPEDLG**K**EPTPSKKPVE  EDLGKEPTPS**K**KPVEAAKETT  DLGKEPTPSK**K**PVEAAKETTE  TPSKKPVEAA**K**ETTEVPEELT  PMPETSEGAG**K**EEDVGIGDYD  YEGIGGPRGE**K**GQKGEPAIIE  IGGPRGEKGQ**K**GEPAIIEPGM  RFGGGGDAGS**K**GPMVSAQESQ  PVGPPGSGGL**K**GEPGDVGPQG  GVQGPPGPAG**K**PGRRGRAGSD  EPGPRGLLGP**K**GPPGPPGPPG  GPPGEKGPLG**K**PGLPGMPGAD  GADGPPGHPG**K**EGPPGEKGGQ  FPGFKGDMGI**K**GDRGEIGPPG  GPLGPPGEKG**K**LGVPGLPGYP  GEKGGRGTPG**K**PGPRGQRGPT  GERGPRGITG**K**PGPKGNSGGD  PRGITGKPGP**K**GNSGGDGPAG  PQGPTGFPGP**K**GPPGPPGKDG  GPKGPPGPPG**K**DGLPGHPGQR  GQRGETGFQG**K**TGPPGPPGVV  GEQGLPGLAG**K**EGTKGDPGPA  LPGLAGKEGT**K**GDPGPAGLPG  GDPGPAGLPG**K**DGPPGLRGFP  LPGPVGALGL**K**GNEGPPGPPG  PQGPPGPAGE**K**GAPGEKGPQG  PAGEKGAPGE**K**GPQGPAGRDG  PVGPPGEDGD**K**GEIGEPGQKG  DKGEIGEPGQ**K**GSKGDKGEQG  EIGEPGQKGS**K**GDKGEQGPPG  EPGQKGSKGD**K**GEQGPPGPTG  PRGQQGLFGQ**K**GDEGPRGFPG  LQGLPGPPGE**K**GETGDVGQMG  GIGNPGAVGE**K**GEPGEAGEPG  LPGEGGPPGP**K**GERGEKGESG  PPGPKGERGE**K**GESGPSGAAG  PSGAAGPPGP**K**GPPGDDGPKG  PKGPPGDDGP**K**GSPGPVGFPG  PAGQDGPPGD**K**GDDGEPGQTG  GEPGPSGPPG**K**RGPPGPAGPE  PAGPEGRQGE**K**GAKGEAGLEG  PEGRQGEKGA**K**GEAGLEGPPG  GEAGLEGPPG**K**TGPIGPQGAP  GPIGPQGAPG**K**PGPDGLRGIP  PMGPPGLPGL**K**GDSGPKGEKG  LPGLKGDSGP**K**GEKGHPGLIG  LKGDSGPKGE**K**GHPGLIGLIG  LIGPPGEQGE**K**GDRGLPGPQG  LPGPQGSSGP**K**GEQGITGPSG  PPGLPGPPGP**K**GAKGSSGPTG  LPGPPGPKGA**K**GSSGPTGPKG  AKGSSGPTGP**K**GEAGHPGPPG  EEIFGSLNSL**K**LEIEQMKRPL  NSLKLEIEQM**K**RPLGTQQNPA  GTQQNPARTC**K**DLQLCHPDFP  PNQGCSRDSF**K**VYCNFTAGGS  AGGSTCVFPD**K**KSEGARITSW  GGSTCVFPDK**K**SEGARITSWP  SEGARITSWP**K**ENPGSWFSEF  ENPGSWFSEF**K**RGKLLSYVDA  GSWFSEFKRG**K**LLSYVDAEGN  WQDAATGSYD**K**ALRFLGSNDE  IRALVDGCAT**K**KGYQKTVLEI  RALVDGCATK**K**GYQKTVLEID  DGCATKKGYQ**K**TVLEIDTPKV  QKTVLEIDTP**K**VEQVPIVDIM  MFNDFGEASQ**K**FGFEVGPACF  LGAEMTTFSQ**K**ILANACTLVM  GPAGREGPSG**K**QGSMGPPGTP  ESGLAEVNAL**K**QRVTILDGHL  RRFQNAFSQY**K**KAVLFPDGQA  RFQNAFSQYK**K**AVLFPDGQAV  LFPDGQAVGE**K**IFKTAGAVKS  DGQAVGEKIF**K**TAGAVKSYSD  EKIFKTAGAV**K**SYSDAEQLCR  SDAEQLCREA**K**GQLASPRSSA  AVTQMVRAQE**K**NAYLSMNDIS  ENCVEIFPDG**K**WNDVPCSKQL  DGKWNDVPCS**K**QLLVICEFFE  QLNENKQERD**K**ENRHRKRSHS  QERDKENRHR**K**RSHSRSRSRD  SHSRSRSRDR**K**RRSRSRDRRN  SASRDRRRRS**K**PLTRGAKEEH  RRSKPLTRGA**K**EEHGGLIRSP  GGLIRSPRHE**K**KKKVRKYWDV  GLIRSPRHEK**K**KKVRKYWDVP  LIRSPRHEKK**K**KVRKYWDVPP  IRSPRHEKKK**K**VRKYWDVPPP  PRHEKKKKVR**K**YWDVPPPGFE  GFEHITPMQY**K**AMQAAGQIPA  PVLAVQINQD**K**NFAFLEFRSV  DGIIFQGQSL**K**IRRPHDYQPL  VSTVVPDSAH**K**LFIGGLPNYL  KELLTSFGPL**K**AFNLVKDSAT  FGPLKAFNLV**K**DSATGLSKGY  LVKDSATGLS**K**GYAFCEYVDI  AGLNGMQLGD**K**KLLVQRASVG  GLNGMQLGDK**K**LLVQRASVGA  LLVQRASVGA**K**NATLVSPPST  IVEDVRDECS**K**YGLVKSIEIP  RDECSKYGLV**K**SIEIPRPVDG  VDGVEVPGCG**K**IFVEFTSVFD  VEFTSVFDCQ**K**AMQGLTGRKF  QKAMQGLTGR**K**FANRVVVTKY  RKFANRVVVT**K**YCDPDSYHRR  GDSGPMGPIG**K**RGPPGPAGIA  GPPGPAGIAG**K**SGDDGRDGEP  GLSGSKGEQG**K**SGNQGPDGGP  GQTGERGRDG**K**SGLPGLRGVD  GAPGHAGEAG**K**RGSPGSPGPA  GNPGSDGRPG**K**DGRPGIRGKD  GKDGRPGIRG**K**DGKQGEQGPQ  GRPGIRGKDG**K**QGEQGPQGPQ  GPPGARGEPG**K**NGAPGEPGAH  GAHGEQGDAG**K**DGETGAAGPP  GAQGTPGEAG**K**TGERGAVGAT  GSRGDIGPRG**K**AGERGKDGER  GPRGKAGERG**K**DGERGERGEN  GERGTRGATG**K**QGARGPRGLA  GARGPRGLAG**K**RGLRGAGGSR  GPSGPAGTAG**K**QGVKGARGSP  GARGSPGLVG**K**QGDRGSDGEP  GAPGQEGAPG**K**DGLPGLAGRP  GGRGSQGPPG**K**DGQPGPSGRV  AIIKASMMSA**K**IIAFSAVVAT  SVNMAMDKSA**K**APVITIFDHR  FDHRGCSRAP**K**EYTGAKAGGK  SRAPKEYTGA**K**AGGKDDEMMV  KEYTGAKAGG**K**DDEMMVKAQS  AGGKDDEMMV**K**AQSVKIEVST  DEMMVKAQSV**K**IEVSTGTAEG  AEGVLATSLA**K**MTKKTMKALS  VLATSLAKMT**K**KTMKALSTAL  QPLGDLGAEM**K**TLSQRSITNT  RDGREGPRGE**K**GDPGLPGPMG  GSPGISGPAG**K**EGPSGKQGNI  GPAGKEGPSG**K**QGNIGPQGKP  GKQGNIGPQG**K**PGPKGEAGPK  NIGPQGKPGP**K**GEAGPKGEVG  KPGPKGEAGP**K**GEVGAPGMQG  APGMQGSAGA**K**GPAGPKGERG  SAGAKGPAGP**K**GERGAPGEQG  APGSRGPPGL**K**GDRGAPGDRG  DRGAPGDRGI**K**GESGLPDSAA  LRQQMEALNG**K**LQRLEAAFSR  QRLEAAFSRY**K**KAALFPDGQS  RLEAAFSRYK**K**AALFPDGQSV  LFPDGQSVGD**K**IFRAANSEEP  ANSEEPFEDA**K**EMCRQAGGQL  AVQQLVTAHS**K**AAFLSMTDVG  LSMTDVGTEG**K**FTYPTGEALV  EIFTNGQWND**K**ACGEQRLVIC  KEIKKFVKWK**K**WKVFKKIEKV  IKKFVKWKKW**K**VFKKIEKVGR  FVKWKKWKVF**K**KIEKVGRNIR  VKWKKWKVFK**K**IEKVGRNIRD  KKWKVFKKIE**K**VGRNIRDGIV  IKKFVKWRRW**K**VFKKIEKMGR  FVKWRRWKVF**K**KIEKMGRNIR  VKWRRWKVFK**K**IEKMGRNIRD  RRWKVFKKIE**K**MGRNIRDGVI  APAIEVLGQA**K**KAQGLVEIAP  NKACSGCTHG**K**KGHTCGSCAK  ALTKAFMMFA**K**TLASLAVIGS  PAGAAMDKSA**K**APQITIFDHR  FDHRGCSRAP**K**ESTGGKAGGQ  SRAPKESTGG**K**AGGQDDEMMV  AGGQDDEMMV**K**VASTKVTVSE  DEMMVKVAST**K**VTVSESDAAK  KVTVSESDAA**K**KLQEFITFEK  VTVSESDAAK**K**LQEFITFEKG  KKLQEFITFE**K**GIDGPFTSKN  EKGIDGPFTS**K**NNKSTFPGDI  VIRAIRELSY**K**GGNTRTGAAI  LPQLARPGVP**K**VCILITDGKS  PKVCILITDG**K**SQDLVDTAAQ  DLVDTAAQRL**K**GQGVKLFAVG  AAQRLKGQGV**K**LFAVGIKNAD  QGVKLFAVGI**K**NADPEELKRV  GIKNADPEEL**K**RVASQPTSDF  TAASGPVTGY**K**VQYTPLTGLG  RRETGLEPPQ**K**VVLPSDVTRY  VSWHSAHGPE**K**SQLVSGEATV  RPEPCPVYCP**K**GQKGEPGEMG  PCPVYCPKGQ**K**GEPGEMGLRG  PQGPPGSATA**K**GERGFPGADG  NPGTPGAPGL**K**GSPGLPGPRG  DPGERGPRGP**K**GEPGAPGQVI  GGEGPGLPGR**K**GDPGPSGPPG  GPPGLPGTAM**K**GDKGDRGERG  GLPGTAMKGD**K**GDRGERGPPG  GPQGPVGPPG**K**KGEKGDSEDG  PQGPVGPPGK**K**GEKGDSEDGA  PVGPPGKKGE**K**GDSEDGAPGL  PRGPPGAIGP**K**GDRGFPGPLG  FPGPLGEAGE**K**GERGPPGPAG  LPGVAGRPGA**K**GPEGPPGPTG  PPGPTGRQGE**K**GEPGRPGDPA  AVVGPAVAGP**K**GEKGDVGPAG  GPAVAGPKGE**K**GDVGPAGPRG  GLVLPGDPGP**K**GDPGDRGPIG  PPGDSGPPGE**K**GDPGRPGPPG  PRGRDGEVGE**K**GDEGPPGDPG  GPPGDPGLPG**K**AGERGLRGAP  APGVRGPVGE**K**GDQGDPGEDG  RNGSPGSSGP**K**GDRGEPGPPG  LVDTGPGARE**K**GEPGDRGQEG  DRGQEGPRGP**K**GDPGLPGAPG  DPGVRGPAGE**K**GDRGPPGLDG  GLDGRSGLDG**K**PGAAGPSGPN  GPSGPNGAAG**K**AGDPGRDGLP  GPSGPPGLPG**K**PGEDGKPGLN  GLPGKPGEDG**K**PGLNGKNGEP  GEDGKPGLNG**K**NGEPGDPGED  EPGDPGEDGR**K**GEKGDSGASG  DPGEDGRKGE**K**GDSGASGREG  ASGREGRDGP**K**GERGAPGILG  FPGVPGGTGP**K**GDRGETGSKG  PKGDRGETGS**K**GEQGLPGERG  VDRLLETAGI**K**ASALREIVET  LPVPERRRGP**K**GDSGEQGPPG  GDSGEQGPPG**K**EGPIGFPGER  PIGFPGERGL**K**GDRGDPGPQG  GPSGLAGEPG**K**PGIPGLPGRA  RPGERGERGE**K**GERGEQGRDG  TPGPPGPPGP**K**VSVDEPGPGL  LSGEQGPPGL**K**GAKGEPGSNG  EQGPPGLKGA**K**GEPGSNGDQG  EPGSNGDQGP**K**GDRGVPGIKG  PKGDRGVPGI**K**GDRGEPGPRG  GERGMAGPEG**K**PGLQGPRGPP  PAGPQGPSGL**K**GEPGETGPPG  GLPGQVGETG**K**PGAPGRDGAS  GAPGRDGASG**K**DGDRGSPGVP  SPGLPGPVGP**K**GEPGPTGAPG  GQAVVGLPGA**K**GEKGAPGGLA  VVGLPGAKGE**K**GAPGGLAGDL  AGDLVGEPGA**K**GDRGLPGPRG  DRGLPGPRGE**K**GEAGRAGEPG  EPGDPGEDGQ**K**GAPGPKGFKG  EDGQKGAPGP**K**GFKGDPGVGV  QKGAPGPKGF**K**GDPGVGVPGS  SPGPPGPPGV**K**GDLGLPGLPG  SVGPPGASGL**K**GDKGDPGVGL  PPGASGLKGD**K**GDPGVGLPGP  PPGSRGERGE**K**GDVGSAGLKG  EKGDVGSAGL**K**GDKGDSAVIL  DVGSAGLKGD**K**GDSAVILGPP  ILGPPGPRGA**K**GDMGERGPRG  ERGPRGLDGD**K**GPRGDNGDPG  PRGDNGDPGD**K**GSKGEPGDKG  DNGDPGDKGS**K**GEPGDKGSAG  DKGSKGEPGD**K**GSAGLPGLRG  GIPGDPGSPG**K**DGVPGIRGEK  KDGVPGIRGE**K**GDVGFMGPRG  VKGACGLDGE**K**GDKGEAGPPG  ACGLDGEKGD**K**GEAGPPGRPG  PPGRPGLAGH**K**GEMGEPGVPG  GVPGQSGAPG**K**EGLIGPKGDR  APGKEGLIGP**K**GDRGFDGQPG  DRGFDGQPGP**K**GDQGEKGERG  QPGPKGDQGE**K**GERGTPGIGG  PRGPEGLQGQ**K**GERGPPGERV  RPGPAGPRGE**K**GEAALTEDDI  RMKRQFIKMM**K**IFQRKMRYWL  FIKMMKIFQR**K**MRYWLLPPFL  ISHDSIQISW**K**APRGKFGGYK  IQISWKAPRG**K**FGGYKLLVTP  KAPRGKFGGY**K**LLVTPTSGGK  KLLVTPTSGG**K**TNQLNLQNTA  NQLNLQNTAT**K**AIIQGLMPDQ  NYTVQIIAYN**K**DKESKPAQGQ  TVQIIAYNKD**K**ESKPAQGQFR  IIAYNKDKES**K**PAQGQFRIKD  SKPAQGQFRI**K**DLEKRKDPKP  QGQFRIKDLE**K**RKDPKPRVKV  QFRIKDLEKR**K**DPKPRVKVVD  IKDLEKRKDP**K**PRVKVVDRGN  EKRKDPKPRV**K**VVDRGNGSRP  GSRPSSPEEV**K**FVCQTPAIAD  LVTAFDVGSE**K**TRIGLAQYSG  IEWHLNAFST**K**DEVIEAVRNL  VIEAVRNLPY**K**GGNTLTGLAL  ALNYIFENSF**K**PEAGSRTGVS  PEAGSRTGVS**K**IGILITDGKS  SKIGILITDG**K**SQDDIIPPSR  SGVELFAIGV**K**NADVNELQEI  SRVEEQDREI**K**ASAHAITGPP  NWTHAPGNVE**K**YRVVYYPTRG  RVVYYPTRGG**K**PDEVVVDGTV  VDGTVSSTVL**K**NLMSLTEYQI  YDVTENSMRV**K**WDAVPGASGY  PLTEGLAGDE**K**EMKIGETHTD  EGLAGDEKEM**K**IGETHTDIEL  EVDPITTFPL**K**GLTPLTEYTI  HPLSADEGLH**K**LMWIPVYGGK  KLMWIPVYGG**K**TEEVVLKEEQ  YGGKTEEVVL**K**EEQDSHVIEG  GDETTSSLRV**K**WDISDSDVQQ  VPGSQNNLLL**K**PLLPDTEYKV  LKPLLPDTEY**K**VTVTPIYTDG  GEGVSVSAPG**K**TLPSSGPQNL  ITWDPPSSPV**K**GYRIVYKPVS  SPVKGYRIVY**K**PVSVPGPTLE  NLLSGMDYNV**K**IFASQASGFS  GFSDALTGMV**K**TLFLGVTNLQ  LFLGVTNLQA**K**HVEMTSLCAH  VVIESLQDRQ**K**QESTVGGGTT  FYGLQPDSEY**K**ISVYTKLQEI  DSEYKISVYT**K**LQEIEGPSVS  IEGPSVSIME**K**TQSLPTRPPT  PTFPPTIPPA**K**EVCKAAKADL  PTIPPAKEVC**K**AAKADLVFMV  PPAKEVCKAA**K**ADLVFMVDGS  SWSIGDENFN**K**IISFLYSTVG  FLYSTVGALN**K**IGTDGTQVAM  QFTDDPRTEF**K**LNAYKTKETL  PRTEFKLNAY**K**TKETLLDAIK  TEFKLNAYKT**K**ETLLDAIKHI  KTKETLLDAI**K**HISYKGGNTK  LLDAIKHISY**K**GGNTKTGKAI  KHISYKGGNT**K**TGKAIKYVRD  SYKGGNTKTG**K**AIKYVRDTLF  GGNTKTGKAI**K**YVRDTLFTAE  AESGTRRGIP**K**VIVVITDGRS  TDGRSQDDVN**K**ISREMQLDGY  DYSELVSIGS**K**PSARHVFFVD  VFFVDDFDAF**K**KIEDELITFV  FFVDDFDAFK**K**IEDELITFVC  TASATCPVVH**K**DGIDLAGFKM  HKDGIDLAGF**K**MMEMFGLVEK  KMMEMFGLVE**K**DFSSVEGVSM  FNVFPCYQLH**K**DALVSQPTRY  EPFALWEILN**K**NSDPLVGVIL  LVGVILDNGG**K**TLTYFNYDQS  TVTFEGPEIR**K**IFYGSFHKLH  IRKIFYGSFH**K**LHIVVSETLV  LHIVVSETLV**K**VVIDCKQVGE  ETLVKVVIDC**K**QVGEKAMNAS  VVIDCKQVGE**K**AMNASANITS  ITSDGVEVLG**K**MVRSRGPGGN  VCSTSWANTD**K**CCELPGLRDD  GSPGQRGLPG**K**DGSSGPPGPP  TIPNMPIRFT**K**IFYNQQNHYD  QQNHYDGSTG**K**FHCNIPGLYY  YFAYHITVYM**K**DVKVSLFKKD  YHITVYMKDV**K**VSLFKKDKAM  YMKDVKVSLF**K**KDKAMLFTYD  MKDVKVSLFK**K**DKAMLFTYDQ  DVKVSLFKKD**K**AMLFTYDQYQ  TVPNVPIRFT**K**IFYNQQNHYD  QQNHYDGTTG**K**FLCNIPGLYY  YFSYHITVYL**K**DVKVSLYKND  YHITVYLKDV**K**VSLYKNDKAL  YLKDVKVSLY**K**NDKALLFTHD  DVKVSLYKND**K**ALLFTHDQFQ  LLFTHDQFQD**K**NVDQASGSVL  ASGSVLLYLE**K**GDQVWLQVYE  LPGRDGRDGA**K**GDKGDAGEPG  RDGRDGAKGD**K**GDAGEPGHPG  GEPGHPGGPG**K**DGIRGEKGEP  EPGADGRVEA**K**GIKGDPGSRG  ADGRVEAKGI**K**GDPGSRGSPG  GDPGSRGSPG**K**HGPKGSIGPT  SRGSPGKHGP**K**GSIGPTGEQG  LPGETGPQGQ**K**GDKGEVGPTG  LMGSTGPLGP**K**GLPGPMGPIG  GLPGPMGPIG**K**PGPRGEAGPM  EPGVRGMRGW**K**GDRGEKGKVG  MRGWKGDRGE**K**GKVGEAPLVP  GWKGDRGEKG**K**VGEAPLVPKS  GKVGEAPLVP**K**SAFTVGLTVI  AFTVGLTVIS**K**FPPPDAPIKF  SKFPPPDAPI**K**FDKILYNELN  PPPDAPIKFD**K**ILYNELNHYN  ELNHYNVATG**K**FTCHVAGVYY  FSRNVQVSLV**K**NGVKVLHTKD  VQVSLVKNGV**K**VLHTKDSYMS  VKNGVKVLHT**K**DSYMSSEDQA  QASGGIVQEL**K**LGDEVWMQVT  ELAPALVPPP**K**GTCAGWMAGI  QQNHYDGSTG**K**FYCNIPGLYY  YFSYHITVYM**K**DVKVSLFKKD  YHITVYMKDV**K**VSLFKKDKAV  YMKDVKVSLF**K**KDKAVLFTYD  MKDVKVSLFK**K**DKAVLFTYDQ  DVKVSLFKKD**K**AVLFTYDQYQ  VLFTYDQYQE**K**NVDQASGSVL  LPFIATLKMM**K**LTAIFPLLFT  AAANLSTEDS**K**SAQLISADSS  EEEELRILSS**K**KSGSYYSYGT  EEELRILSSK**K**SGSYYSYGTK  KSGSYYSYGT**K**KSGSYSGYST  SGSYYSYGTK**K**SGSYSGYSTK  KSGSYSGYST**K**KSASRRILSS  SGSYSGYSTK**K**SASRRILSSK  KSASRRILSS**K**KSGSYSGYST  SASRRILSSK**K**SGSYSGYSTK  KSGSYSGYST**K**KSGSRRILSS  SGSYSGYSTK**K**SGSRRILSSK  KSGSRRILSS**K**KSGSYSGSKG  SGSRRILSSK**K**SGSYSGSKGS  SKKSGSYSGS**K**GSKRRILSSK  SGSYSGSKGS**K**RRILSSKKSG  KGSKRRILSS**K**KSGSYSGSKG  GSKRRILSSK**K**SGSYSGSKGS  SKKSGSYSGS**K**GSKRRNLSSK  SGSYSGSKGS**K**RRNLSSKKSG  KGSKRRNLSS**K**KSGSYSGSKG  GSKRRNLSSK**K**SGSYSGSKGS  SKKSGSYSGS**K**GSKRRILSGG  SGSYSGSKGS**K**RRILSGGLRG  RDGDTTRQRI**K**FSDDRVCKSH  RIKFSDDRVC**K**SHLLNCCPHD  GTRMDLGECL**K**VHDLALRADY  ALRADYEIAS**K**EQDFFFELDA  ADCDRRTEVA**K**KRLAETQEEI  DCDRRTEVAK**K**RLAETQEEIS  QEEISAEVAA**K**AERVHELNEE  RVHELNEEIG**K**LLAKVEQLGA  LNEEIGKLLA**K**VEQLGAEGNV  GAEGNVEESQ**K**VMDEVEKARA  ESQKVMDEVE**K**ARAKKREAEE  VMDEVEKARA**K**KREAEEVYRN  MDEVEKARAK**K**REAEEVYRNS  SMPASSFQQQ**K**LRVCEVCSAY  DRRLADHFGG**K**LHLGFIEIRE  LHLGFIEIRE**K**LEELKRVVAE  IEIREKLEEL**K**RVVAEKQEKR  LEELKRVVAE**K**QEKRNQERLK  LKRVVAEKQE**K**RNQERLKRRE  KQEKRNQERL**K**RREEREREER  REEREREERE**K**LRRSRSHSKN  HRSRSMSRER**K**RRTRSKSREK  SRERKRRTRS**K**SREKRHRHRS  KRRTRSKSRE**K**RHRHRSRSSS  SSRDRSRERS**K**RRSSKERFRD  SRERSKRRSS**K**ERFRDQDLAS  RDRSPRDRDR**K**DKKRSYESAN  RSPRDRDRKD**K**KRSYESANGR  SPRDRDRKDK**K**RSYESANGRS |
